# Sclerofish A and B: Two Pairs of Enantiomeric Diterpenoids Featuring a 4/7/6-Fused Tricyclic Scaffold from the Soft Coral *Sclerophytum humesi*

**DOI:** 10.1021/acs.joc.6c00512

**Published:** 2026-06-01

**Authors:** Phuong Vu Luu, Thuy-Tien Thi Phan, Quoc-Dung Tran Huynh, Ngoc-Thac Pham, Huong-Giang Le, Lo-Yun Chen, Huong Lien Ton-Nu, Cuong-Quoc Nguyen, Yao-An Shen, Yu-Jui Fan, Jui-Hsin Su, Bo-Rong Peng, Kuei-Hung Lai

**Affiliations:** † Institute of Pharmaceutical Education and Research, Binh Duong University, Thu Dau Mot, Binh Duong 820000, Vietnam; ‡ Institute of Biological Chemistry, Academia Sinica, Taipei 115024, Taiwan; § Department of Pharmacognosy and Traditional Pharmacy, School of Pharmacy, University of Medicine and Pharmacy at Ho Chi Minh City, Ho Chi Minh City 700000, Vietnam; ∥ PhD Program in Clinical Drug Development of Herbal Medicine, College of Pharmacy, Taipei Medical University, Taipei 110301, Taiwan; ⊥ Department of Chemistry, College of Natural Sciences, Can Tho University, Can Tho 94000, Vietnam; # Department of Health Sciences, College of Natural Sciences, Can Tho University, Can Tho 94000, Vietnam; ¶ Department of Pathology, School of Medicine, College of Medicine, Taipei Medical University, Taipei 110301, Taiwan; ○ International Master/Ph.D. Program in Medicine, College of Medicine, Taipei Medical University, Taipei 110301, Taiwan; ⧫ National Yang Ming Chiao Tung University, Department of Mechanical Engineering, Hsinchu 300093, Taiwan; †† National Museum of Marine Biology and Aquarium, Pingtung 94450, Taiwan; ‡‡ Department of Marine Biotechnology and Resources, National Sun Yat-sen University, Kaohsiung 804, Taiwan; §§ Graduate Institute of Marine Biology, National Dong Hwa University, Pingtung 94450, Taiwan; ∥∥ Graduate Institute of Pharmacognosy, College of Pharmacy, Taipei Medical University, Taipei 110301, Taiwan; ⊥⊥ Graduate Institute of Biomedical Materials and Tissue Engineering, 397 College of Biomedical Engineering, Taipei Medical University, Taipei 110301, Taiwan; ( Department of Biochemistry and Molecular Cell Biology, School of Medicine, College of Medicine, Taipei Medical University, Taipei 110301, Taiwan; _ Graduate Institute of Healthy Industry Technology, Center for Drug Research and Development, College of Human Ecology, Chang Gung University of Science and Technology, Taoyuan 333324, Taiwan; ) Traditional Herbal Medicine Research Center, Taipei Medical University Hospital, Taipei 110301, Taiwan; + PhD Program in Drug Discovery and Development Industry, College of Pharmacy, Taipei Medical University, Taipei 110301, Taiwan

## Abstract

Two pairs of enantiomeric diterpenoids, (±)-sclerofish A (**1**) and (±)-sclerofish B (**2**), featuring a rare 4/7/6-fused tricyclic framework, were isolated from the soft coral *Sclerophytum humesi* by molecular networking-guided isolation. Their structures were elucidated by comprehensive spectroscopic analyses, including NMR, HRESIMS, TDDFT-ECD, and DP4+ analysis. A plausible biogenetic pathway, originating from geranylgeranyl pyrophosphate (GGPP) was proposed to rationalize the formation of the unusual bicyclo­[4.3.1^4,8^]­decane subunit and the resulting 4/7/6-fused skeleton. Compound **1a/1b** exhibited inhibition of Huh-7 cells with IC_50_ values of 5.1 ± 0.9 and 4.9 ± 0.3 μM, respectively, and showed selective cytotoxicity toward cancer cells (SI > 9.7), whereas compound **2a/2b** displayed comparatively weaker activity. These findings expand the structural diversity of xeniaphyllane-type diterpenoids and highlight the soft coral *S. humesi* as a valuable source of structurally unique and biologically relevant marine natural products.

## Graphical abstract

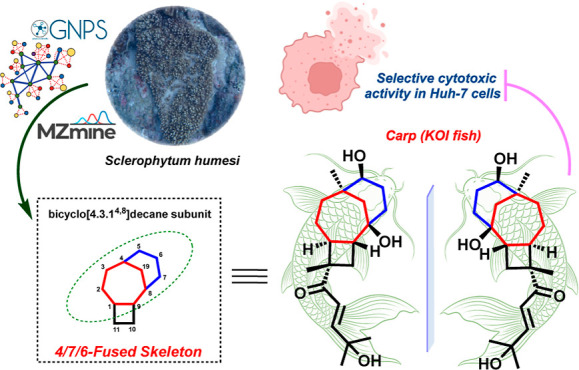

## Introduction

Soft corals of the genus *Sclerophytum* are recognized as prolific sources of structurally diverse terpenoids.^1^ Among them, *S. humesi* has attracted particular attention owing to its remarkable capacity to produce bioactive norsesquiterpenoids and diterpenoids.^1^ Early chemical investigations by our group revealed a characteristic dominance of xeniaphyllane-type diterpenoids, distinguished by a bicyclo[7.2.0]­undecane ring system bearing a 4-methylpentyl side chain, providing important insights into the chemotaxonomy and biosynthetic potential of this genus.^1a,1c^ The diterpenoid biosynthetic pathway in soft corals is thought to originate from geranylgeranyl pyrophosphate (GGPP), which undergoes elaborate cyclization cascades mediated by specialized terpene cyclases.^2^ Environmental factors in benthic habitats may influence the metabolism of symbiotic microorganisms. Their enzymatic systems could promote the formation of structurally rare diterpenoids with distinctive and potentially enhanced biological activities.^3^ As a result, the soft coral *S. humesi* is capable of producing not only the commonly observed 4/9 frameworks but also structurally rare fused-ring systems.

As part of our investigation of xeniaphyllane-type diterpenoids from the soft coral *S. humesi*, two diterpenoids featuring a 4/7/6-fused tricyclic framework were isolated using molecular networking (MN)-guided isolation. Visualization of the MN revealed two unannotated nodes at *m*/*z* 337.237 and 359.221 associated with known diterpenoids, including sclerohumins G and H, as well as gibberosins G and H (Figure 1A). These nodes were prioritized based on their spectral connectivity, relative abundance, and distinctive MS/MS fragmentation patterns within the xeniaphyllane cluster. Although the *m*/*z* 337.237 node was only indirectly connected to annotated compounds, its similar fragmentation features and close proximity within the network suggested a shared biosynthetic origin. In contrast, other nodes (e.g., *m*/*z* 361.199) were not selected due to their lower abundance and less informative fragmentation patterns. Targeted isolation subsequently led to the identification of compounds **1** and **2**, both of which exhibited characteristic MS/MS fragmentation patterns consistent with biosynthetically related xeniaphyllane-type derivatives (Figure 1B, Table 1).^1c^ Notably, both compounds were isolated as racemic mixtures and were resolved into enantiomeric pairs, indicating the absence of strict stereochemical control during their biosynthesis.^4^ Such enantiomeric natural products are increasingly recognized in marine secondary metabolism and are often attributed to either nonenantioselective enzyme catalysis or parallel biosynthetic pathways operating within the coral holobiont.^4a^ This discovery expands the diterpenoid chemical space of the soft coral *S. humesi* and strongly implies the involvement of alternative cyclization and/or rearrangement processes beyond those associated with the canonical 4/9-fused framework.

**Figure 1:**
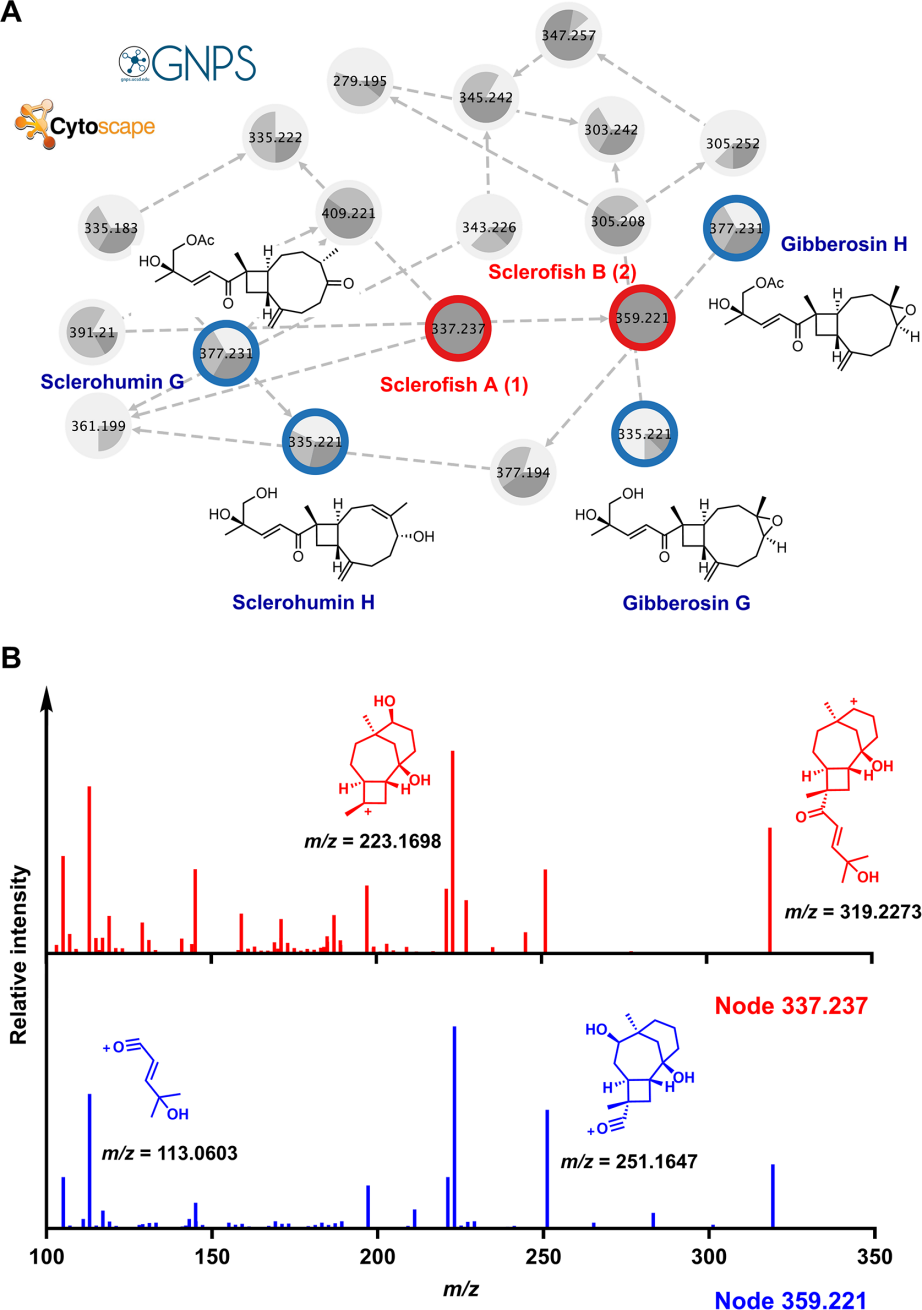
Molecular networking (MN) illustrates the chemical diversity of diterpenoids obtained from the soft coral *S. humesi*. (**A**) MN visualization colored according to precursor *m*/*z* values. (**B**) Alignment and comparison of the MS/MS fragmentation spectra of sclerofish A (**1**) and sclerofish B (**2**).

**Table 1:** Annotated Terpenoids Originating From the Fractions of the Soft Coral *S. humesi*

no	*t*_R_ (min)	compound name	ion adduct	precursor ion (*m*/*z*)	product ion (*m*/*z*)	molecular formula (error in ppm)	reference
1	5.92	sclerohumin H	[M + H]^+^	335.221	105, 115, 129, 145, 169, 197, 275, 317	C_20_H_30_O_4_ (−2.98)	^1c^
2	6.54	sclerohumin G	[M + H]^+^	377.231	105, 143, 169, 187, 211, 281, 317	C_22_H_32_O_5_ (−5.32)	^1c^
3	6.34	gibberosin H	[M + H]^+^	377.231	105, 131, 187, 211, 235, 281, 317	C_22_H_32_O_5_ (6.34)	GNPS libraries
4	8.07	gibberosin G	[M + H]^+^	335.221	105, 117, 145, 187, 209, 227, 275	C_20_H_30_O_4_ (−2.98)	GNPS libraries
5	7.41	sclerofish A	[M + H]^+^	337.237	105, 113, 145, 197, 221, 223, 251, 319	C_20_H_32_O_4_ (−2.37)	New
6	7.72	sclerofish B	[M + Na]^+^	359.221	105, 113, 145, 197, 221, 223, 251, 319	C_20_H_32_O_4_ (3.62)	New

## Results and Discussion

Sclerofish A (**1**) was obtained as a colorless oil. Its molecular formula C_20_H_32_O_4_, as established from the HRESIMS peak at *m*/*z* 337.2362 ([M + H]^+^, calcd for C_20_H_33_O_4_, 337.2373), with five degrees of unsaturation. The IR spectrum displayed characteristic absorption bands attributable to hydroxy groups (3356 cm^–1^) and conjugated carbonyl functionalities (1627 cm^–1^) (Figure S10). The ^1^H NMR and HSQC spectroscopic of compound **1** (Table 2) revealed signals corresponding to four tertiary methyl groups at δ_H_ 1.00 (3H, s), 1.29 (3H, s), 1.32 (3H, s), 1.32 (3H, s); six methylene groups at δ_H_ [1.66 (1H, overlapped), 1.52 (1H, m)], [1.34 (1H, m), 1.64 (1H, m)], [1.69 (1H, m), 2.08 (1H, m)], [1.66 (1H, overlapped), 1.75 (1H, m)], [1.70 (1H, m), 1.96 (1H, dd, *J* = 11.1, 10.0 Hz)], [1.83 (1H, d, *J* = 13.4 Hz), and 1.43 (1H, dt, *J* = 13.4, 1.4 Hz)]; three methine protons at δ_H_ 2.03 (1H, m), 2.21 (1H, m), and 3.31 (1H, overlapped), as well as two olefinic protons at δ_H_ 6.42 (1H, d, *J* = 15.5 Hz), and 6.94 (1H, d, *J* = 15.5 Hz). Analysis of the ^13^C NMR, DEPT, and HSQC data of compound **1** (Table 2) established 20 carbon resonances, including four methyl (δ_C_ 15.4, 29.3, 29.3, and 31.8), six sp^3^ methylenes (δ_C_ 27.5, 27.7, 29.5, 32.1, 40.0, and 47.3), three sp^3^ methines (δ_C_ 44.3, 50.5, and 75.1), four sp^3^ quaternary (δ_C_ 39.3, 51.1, 71.4, and 71.8), two olefinic (δ_C_ 121.9 and 155.5), and one conjugated carbonyl carbon (δ_C_ 206.4). The occurrence of one conjugated carbonyl and one double bond accounted for two of the five degrees of unsaturation, indicating that compound **1** possesses a tricyclic fused-ring system. Furthermore, the NMR spectroscopic features of compound **1** show close similarity to those of the side chain commonly observed in xeniaphyllane-type diterpenoids. Specifically, two olefinic protons at [δ_H_ 6.42 (d, *J* = 15.5 Hz), H-13; 6.94 (d, *J* = 15.5 Hz), H-14], two olefinic carbons at [δ_C_ 121.9, C-13; 155.5, C-14], and two tertiary methyl groups at [δ_H_ 1.32 (3H, s), H-16/17; δ_C_ 29.3, C-16/17)] were identified.^1a,1c,1d^ The major structural difference between compound **1** and xeniaphyllanes is the presence of a bicyclo­[4.3.1^4,8^]­decane moiety comprising rings B and C (Figure 2). The existence of this subunit was substantiated by key HMBC correlations from H_3_-20 (δ_H_ 1.00) to C-3 (δ_C_ 40.0)/C-4 (δ_C_ 39.3)/C-5 (δ_C_ 75.1)/C-19 (δ_C_ 47.3), from H-9 to C-1 (δ_C_ 44.3)/C-7 (δ_C_ 27.7)/C-8 (δ_C_ 71.8)/C-10 (δ_C_ 32.1)/C-19 (δ_C_ 47.3), and from H_2_-19 (δ_H_ 1.83, 1.43) to C-4/C-5/C-8 (Part A). Moreover, COSY cross-peaks further confirmed the tricyclic planar structure of compound **1** by establishing three distinct spin systems: H_2_-10/H-9/H-1/H_2_-2/H_2_-3 (Part A) (**a**), H-5/H_2_-6/H_2_-7 (Part A) (**b**), and H-13/H-14 (Part B) (**c**) (Figure 3). Collectively, these data confirm that **1** is a xeniaphyllane-type diterpenoid featuring a rare 4/7/6-fused tricyclic framework.

**Table 2:** ^1^H and ^13^C NMR Data for Compounds **1** and **2** ^a^

no.	1^a^		2^a^	
	δ_H_ (*J* in Hz)	δ_C_	δ_H_ (*J* in Hz)	δ_C_
1	2.21, m	44.3, CH	2.39, overlapped	40.7, CH
2α	1.66, overlapped	27.5, CH_2_	1.74, m	28.8, CH_2_
2β	1.52, m		2.09, m	
3α	1.34, m	40.0, CH_2_	3.38, m	72.8, CH
3β	1.64, m			
4	-	39.3, qC	-	40.2, qC
5α	3.31, overlapped	75.1, CH	1.19, m	36.7, CH_2_
5β			1.55, m	
6	1.69, m	29.5, CH_2_	1.60, m	20.0, CH_2_
	2.08, m		1.72, m	
7	1.66, overlapped	27.7, CH_2_	1.65, m	33.9, CH_2_
	1.75, m		1.51, m	
8	-	71.8, qC	-	71.3, qC
9	2.03, m	50.5, CH	2.39, overlapped	38.2, CH
10α	1.70, m	32.1, CH_2_	1.68, m	31.2, CH_2_
10β	1.96, dd (11.1, 10.0)		2.08, m	
11	-	51.1, qC	-	50.2, qC
12	-	206.4, qC	-	207.0, qC
13	6.42, d (15.5)	121.9, CH	6.41, d (15.5)	122.0, CH
14	6.94, d (15.5)	155.5, CH	6.93, d (15.5)	155.3, CH
15	-	71.4, qC	-	71.4, qC
16	1.32, s	29.3, CH_3_	1.32, s	29.3, CH_3_
17	1.32, s	29.3, CH_3_	1.32, s	29.3, CH_3_
18	1.29, s	15.4, CH_3_	1.27, s	16.4, CH_3_
19α	1.83, d (13.4)	47.3, CH_2_	1.50, d (13.0)	43.1, CH_2_
19β	1.43, dt (13.4, 1.4)		1.36, ddd (13.0, 2.8, 1.1)	
20	1.00, s	31.8, CH_3_	0.91, s	27.4, CH_3_

a Spectra recorded in CD_3_OD at 600 MHz (^1^H NMR) and 150 MHz (^13^C NMR).

**Figure 2:**
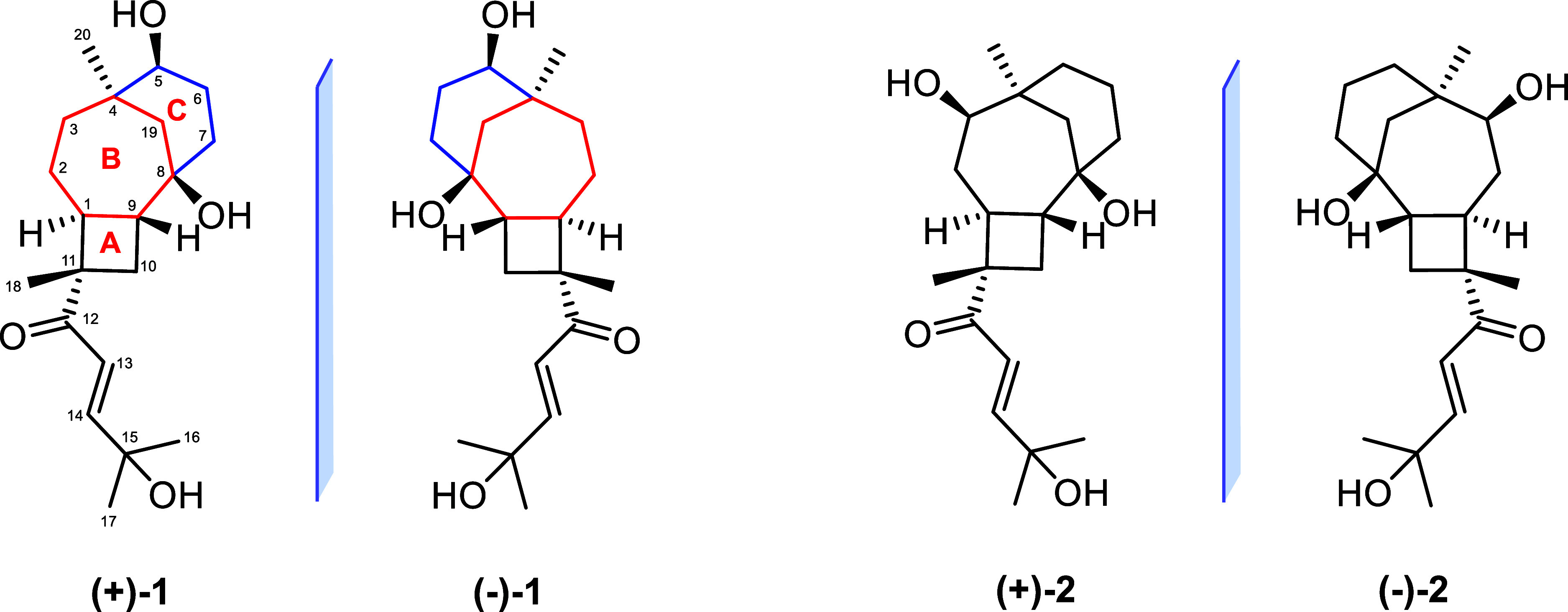
Chemical structures of compounds **1** and **2**.

**Figure 3:**
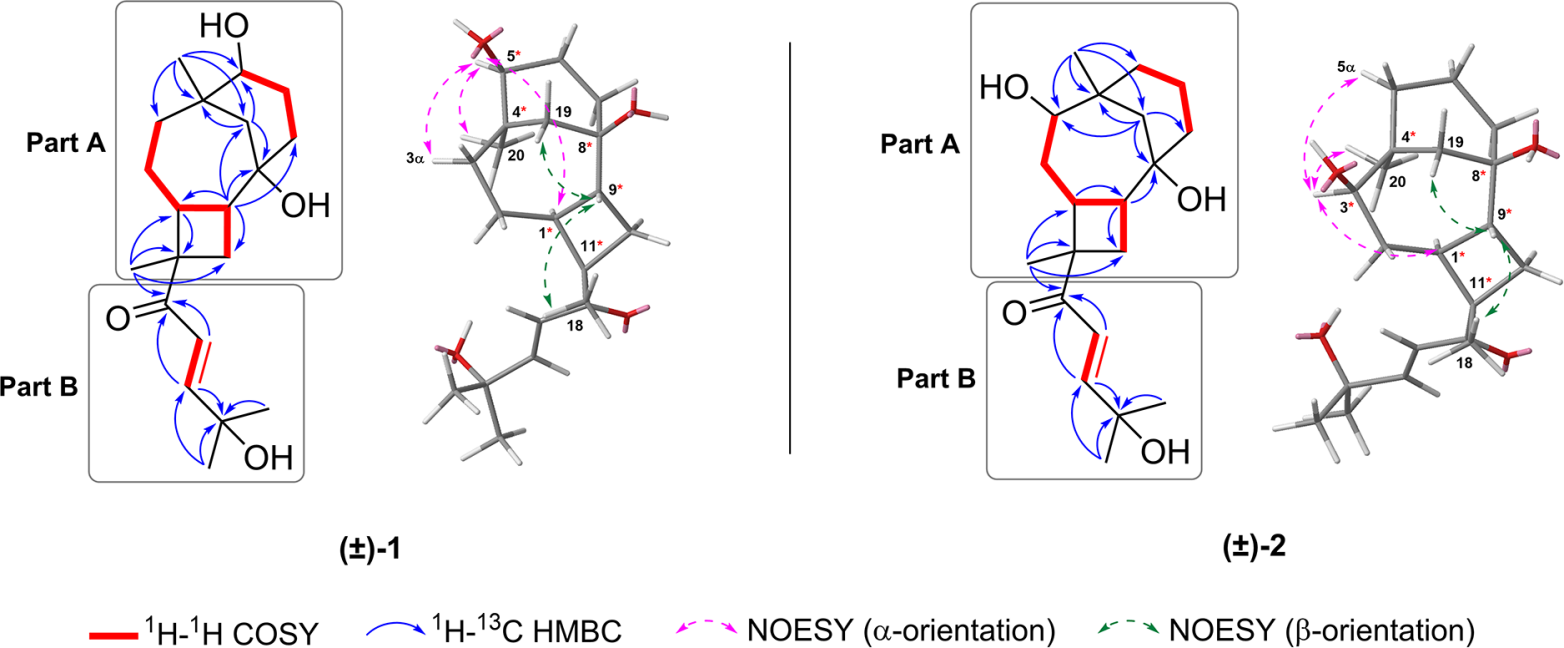
Key ^1^H–^1^H COSY (bold), HMBC (blue arrows), and NOESY correlations (dashed arrows) for compounds **1** and **2**.

The relative configuration of compound **1** was established on the basis of NOESY experiments and coupling constant analysis. Key NOE correlations between H-1 (δ_H_ 2.21)/H-5 (δ_H_ 3.31), H-5/H_3_-20 (δ_H_ 1.00), and H-5/H-3α (δ_H_ 1.34), indicated that H-1, H-5, and H_3_-20 are α-oriented. In contrast, H_3_-18 (δ_H_ 1.29)/H-9 (δ_H_ 2.03), H-9/H-19β (δ_H_ 1.43), suggested that H_3_-18 and H-9 are β-oriented, thereby supporting an R* configuration at C-8. Additionally, the *trans* geometry of the C-13/C-14 double bond was confirmed by the large vicinal coupling constant *J*_13,14_ (15.5 Hz) (Figure 3). However, the relative configuration at C-5 was difficult to establish unambiguously due to conformational effects within the 4/7/6-fused ring system. To address this issue, the stereochemical configuration was investigated using (GIAO) DFT-based NMR calculations.^1a,1c,5^ Accordingly, two candidate diastereomers, (1S*,4R*,5S*,8R*,9R*,11S*)-**1a**, (1S*,4R*,5R*,8R*,9R*,11S*)-**1b**, were generated for computational evaluation at the *m*PW1PW91/6-311+G­(d,p)//B3LYP/6-31G­(d,p) level using the IEFPCM solvation model for methanol. Subsequent DP4+ statistical analysis of the calculated and experimental NMR data favored the (1S*, 4R*, 5S*, 8R*, 9R*, 11S*)-**1a** isomer, with a probability of 100% and mean absolute error (MAE) values of 0.24 and 3.26 for ^1^H and ^13^C NMR, respectively (Figure 4).

**Figure 4:**
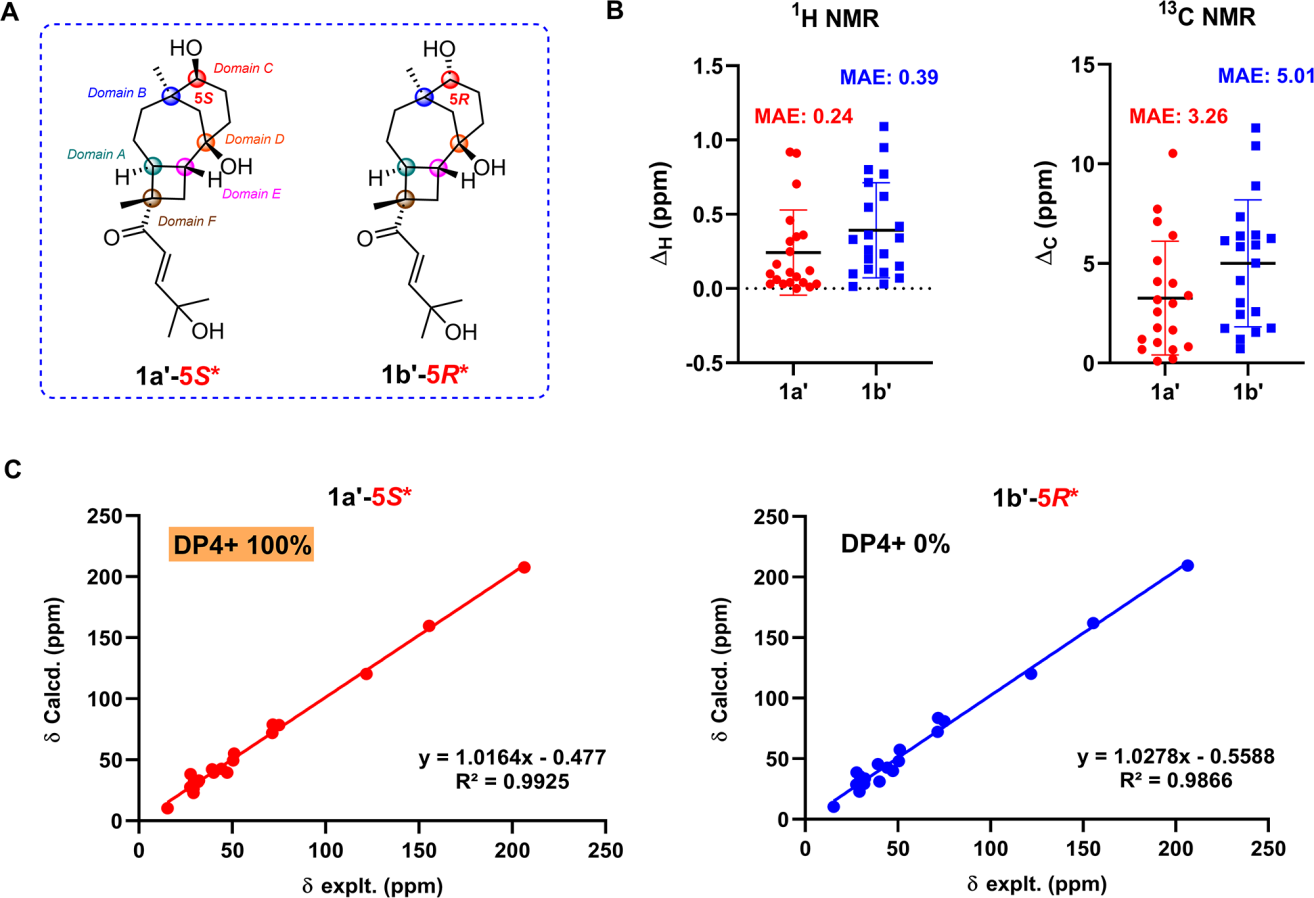
Stereochemical configuration was investigated using (GIAO) DFT-based NMR calculations for compound **1**. (**A**) Two possible diastereomeric models (**1a** and **1b**) proposed for compound **1**. (**B**) Comparison of the corrected calculated NMR chemical shifts of *versus* isomers **1a** and **1b** with the experimental shifts of compound **1**. (**C**) Linear correlations of the calculated isomers of compound **1** with the experimentally observed ^13^C NMR chemical shifts.

Compound **1** was suspected to be a racemic mixture due to its lack of optical activity and the featureless nature of its ECD spectrum. To resolve this, chiral separation was carried out, affording (+)-sclerofish A (**1a**) and (−)-sclerofish A (**1b**) in an approximate 1:1 ratio (see the Supporting Information). The absolute configurations of compounds (+)-**1a** and (−)-**1b** were assigned as 1*S*, 4*R*, 5*S*, 8*R*, 9*R*, 11*S* and 1*R*, 4*S*, 5*R*, 8*S*, 9*S*, 11*R*, respectively, by comparison of their experimental ECD spectra with those calculated using TDDFT/B3LYP/6-311G++(d,p) with the IEFPCM/methanol solvent model (Figure 5). Therefore, compounds (±)-**1** was established as a xeniaphyllane diterpenoid featuring a bicyclo­[4.3.1^4,8^ ^4,8^ ]­decane fragment comprising rings B and C.

**Figure 5:**
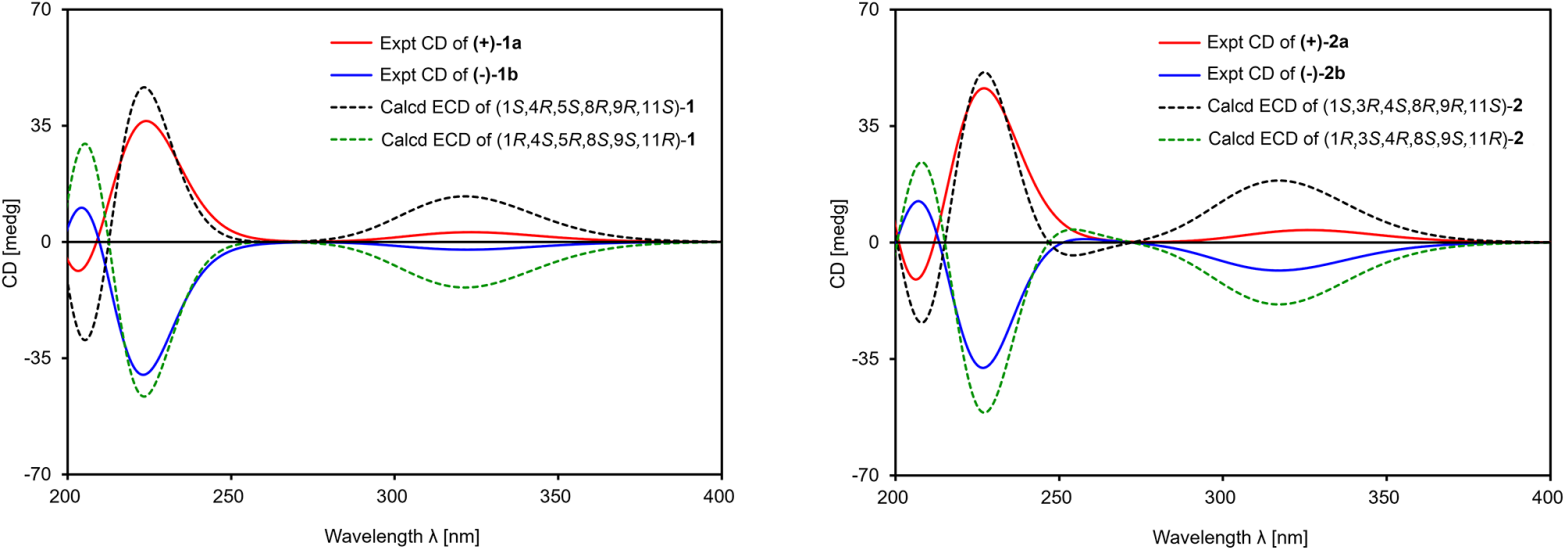
Comparison of experimental and calculated ECD spectra of compounds **1** and **2**.

Sclerofish B (**2**) was isolated as a colorless oil. Its molecular formula C_20_H_32_O_4_, as established from the HRESIMS peak at *m*/*z* 337.2382 ([M + H]^+^, calcd for C_20_H_33_O_4_, 337.2373), with five degrees of unsaturation. The NMR spectroscopic data of compound **2** (Table 2) closely resembled those of compound **1**, with the primary difference being the presence of an oxygenated methine at C-3 [δ_H_ 3.38 (1H, *m*); δ_C_ 72.8]. This structural assignment was supported by comprehensive COSY experiments, which established sequential correlations of H_2_-10/H-9/H-1/H_2_-2/H-3 and H_2_-5/H_2_-6/H_2_-7. Additionally, HMBC correlations were from H_3_-20 (δ_H_ 0.91) to C-3 (δ_C_ 72.8)/C-4 (δ_C_ 40.2)/C-5 (δ_C_ 36.7)/C-19 (δ_C_ 43.1), from H-9 to C-8 (δ_C_ 71.3), and from H_2_-19 (δ_H_ 1.50, 1.36) to C-3/C-5/C-7 (δ_C_ 33.9)/C-8 (Part A). These spectroscopic data establish compound **2** as a diterpenoid sharing the same 4/7/6-fused tricyclic framework as compound **1** (Figure 3). The relative configuration of compound **2** was determined based on NOESY experiments. Key NOE correlations between H-1 (δ_H_ 2.40)/H-3 (δ_H_ 3.38) and H-3/H_3_-20 (δ_H_ 0.91), indicated that H-1, H-3, and H_3_-20 are α-oriented. In contrast, H_3_-18 (δ_H_ 1.27)/H-9 (δ_H_ 2.40), H-9/H-19β (δ_H_ 1.36), suggested that H_3_-18 and H-9 are β-oriented, thereby supporting an R* configuration at C-8. Nevertheless, unambiguous assignment of the relative configuration of compound **2** based on NOE correlations proved challenging due to signal overlap between H-1 (δ_H_ 2.40) and H-9 (δ_H_ 2.40), as well as the presence of a secondary hydroxy group at C-3, which may influence conformational preferences. To achieve a more definitive stereochemical assignment, GIAO-NMR calculations were performed. Accordingly, eight possible diastereomers arising from stereochemical variations at C-1, C-3, and C-9-namely, (1R*, 3R*, 9R*)-**2a**, (1R*, 3R*, 9S*)-**2b**, (1R*, 3S*, 9R*)-**2c**, (1R*, 3S*, 9S*)-**2d**, (1S*, 3R*, 9R*)-**2e**, (1S*, 3R*, 9S*)-**2f**, (1S*, 3S*, 9R*)-**2g**, and (1S*, 3S*, 9S*)-**2h** were calculated. The isomer (1S*, 3R*, 9R*)-**2e** configuration showed an excellent agreement with the experimental data, achieving 100% probability by DP4+ analysis with MAE values of 0.21 and 1.44 for ^1^H and ^13^C NMR, respectively. These results support the assignment of the relative configuration of **2** as 1S*, 3R*, 4S*, 8R*, 9R*, 11S* (Figure 6, see the Supporting Information). Compound **2** was obtained as a pair of enantiomers, which were separated on a chiral HPLC column to yield (+)-**2a** and (−)-**2b**. The two enantiomers exhibited opposite specific optical rotations ([α]_D_^20^ +34.8 and −34.3, *c* 0.1, MeOH) and mirrored ECD spectra, confirming their enantiomeric relationship. Finally, the absolute configurations of (+)-**2a** and (−)-**2b** were unambiguously established as 1*S*, 3*R*, 4*S*, 8*R*, 9*R*, 11*S* and 1*R*, 3*S*, 4*R*, 8*S*, 9*S*, 11*R*, respectively, based on correlation between their experimental and calculated ECD spectra (Figure 5). Thus, the structure of compound **2** was established and designated as (±)-sclerofish B.

**Figure 6:**
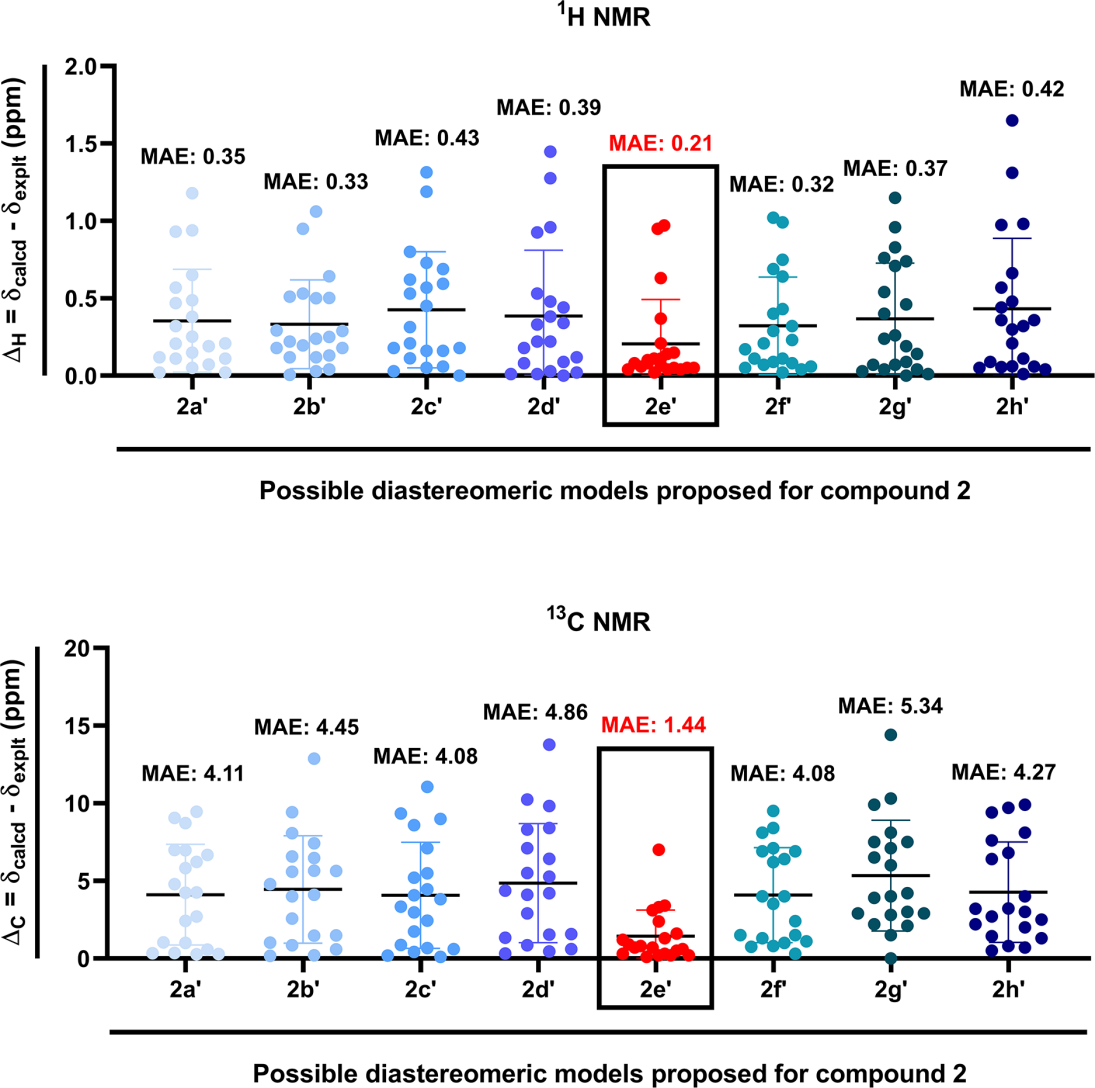
Comparison of the corrected calculated NMR chemical shifts of *versus* isomers **2a-2h** with the experimental shifts using (GIAO) DFT-based NMR calculations for compound **2**.

Structurally, (±)-sclerofish A (**1a**/**1b**) and (±)-sclerofish B (**2a**/**2b**) share a unique tricyclo­[6.3.1.0^1,9^]­dodecane framework, representing a rare structural motif in natural products. To date, only one natural product featuring this tricyclic scaffold has been reported, which was isolated from the stems and leaves of the mangrove plant *Excoecaria agallocha* L.^6^ Moreover, compounds **2a**/**2b** represent the first examples of xeniaphyllane-type diterpenoids bearing a hydroxy group at the C-3 position of the main carbon backbone. A plausible biosynthetic pathway is proposed in Scheme 1. The biosynthesis is presumed to originate from geranylgeranyl pyrophosphate (GGPP), where the departure of the pyrophosphate (-OPP) group, likely catalyzed by a terpene synthase, triggers ionization and cyclization to generate a xeniaphyllene-type macrocyclic intermediate (i–v), consistent with reported coral diterpene biosynthesis.^2a,7^ While the initial cyclization step is likely enzyme-catalyzed, subsequent transformations may proceed via nonenzymatic processes, such as acid-catalyzed alkene isomerization, air-mediated epoxidation, and subsequent acid- or base-catalyzed cyclization. Subsequent stereoselective epoxidation of the olefin, followed by intramolecular epoxide ring opening and the formation of a C-4/C-19/C-8 linkage, generates a key intermediate containing a bicyclo­[4.3.1^4,8^]­decane moiety corresponding to rings B and C, affording the 4/7/6-fused tricyclic framework. Subsequent transformations, including dehydrogenation and allylic oxidation to generate an α,β-unsaturated ketone moiety, furnish (±)-sclerofish A (**1**). Compound (±)-**2** may arise from enantiodivergent cyclization of a common xeniaphyllane precursor, potentially involving olefin isomerization and epoxidation at C-3/C-4, or via parallel enzymatic pathways operating within the soft coral. Overall, these enzymatically orchestrated cyclization, rearrangement, and oxidative transformations from a common GGPP-derived precursor rationalize the structural divergence and enantiomeric nature of (±)-sclerofish A (**1**) and (±)-sclerofish B (**2**) within the xeniaphyllane diterpenoid family.

**Scheme 1:**
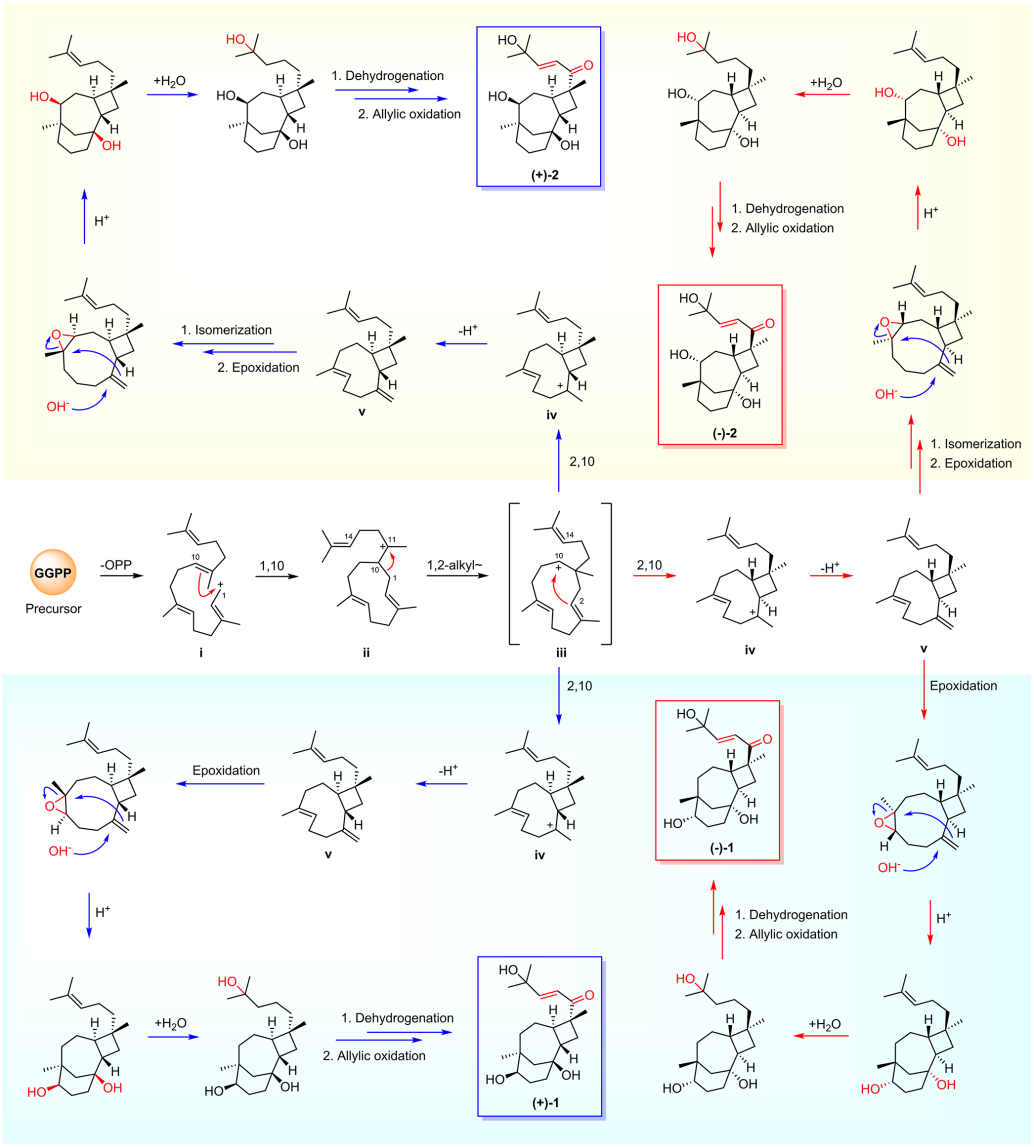
Proposed Biogenetic Pathway for Compounds (±)-1 and (±)-2

The cytotoxic activities of compounds **1** and **2** were evaluated against a panel of human cancer cell lines representing hepatocellular (Huh-7, HLE), pancreatic (PANC-1, MIA PaCa-2, KPC), colorectal (DLD-1, SW620), and lung carcinomas (PC-9), with Vero cells used as a nontumorigenic control (Table 3 and 4). Compound **1** exhibited the most pronounced cytotoxicity, particularly against hepatocellular carcinoma cells. Both enantiomers of **1** showed strong inhibition of Huh-7 cells with IC_50_ values of 5.1 ± 0.9 and 4.9 ± 0.3 μM, respectively, while neither enantiomer was active toward HLE cells (IC_50_ > 50 μM), indicating clear cell-line-dependent sensitivity within hepatocellular carcinoma models. Moderate activity was also observed against MIA PaCa-2, DLD-1, and SW620 cells (IC_50_ = 18–39 μM). Compound **2** was generally less potent than compound **1** across all tested cell lines. Its enantiomers displayed moderate inhibition of Huh-7 (IC_50_ = 16–18 μM) and MIA PaCa-2 cells (IC_50_ = 32–35 μM), with activity against colorectal cancer cells limited to SW620 (IC_50_ around 29 μM), and no significant effects on PANC-1, KPC, DLD-1, HLE, or PC-9 cells (IC_50_ > 50 μM). The enantiomers of both compounds exhibited nearly identical activities, indicating no stereochemical influence on cytotoxicity. Importantly, none of the compounds showed detectable toxicity toward Vero cells (IC_50_ > 50 μM), demonstrating selective inhibition of cancer cells over normal cells. These results highlight compound **1** as the more promising compound, particularly for hepatocellular carcinoma (SI > 9.7 in the Huh-7 model), and support further studies to clarify molecular targets and structure–activity relationships of this new xeniaphyllane-derived chemotype.

**Table 3:** Cytotoxic Activities were Evaluated Against Cell Lines for Compounds (±)-**1** and (±)-**2**

sample	IC_50_ (μM)								
	Huh-7	HLE	PANC-1	MIA PaCa-2	KPC	DLD-1	SW620	PC-9	Vero
**(+)-1**	5.1 ± 0.9	>50	>50	24.8 ± 1.5	>50	38.7 ± 1.8	22.4 ± 1.0	>50	>50
**(−)-1**	4.9 ± 0.3	>50	>50	20.2 ± 0.9	>50	35.5 ± 1.3	18.3 ± 1.2	>50	>50
**(+)-2**	18.4 ± 0.6	>50	>50	34.7 ± 1.2	>50	>50	28.9 ± 1.8	>50	>50
**(−)-2**	16.2 ± 1.5	>50	>50	32.3 ± 1.0	>50	>50	29.2 ± 1.9	>50	>50
sorafenib^a^	7.7 ± 0.7	5.8 ± 0.7	NT^e^	NT^e^	NT^e^	NT^e^	NT^e^	NT^e^	NT^e^
gemcitabine^b^	NT^e^	NT^e^	11.6 ± 0.6	7.6 ± 0.9	2.8 ± 0.4	NT^e^	NT^e^	NT^e^	NT^e^
regorafenib^c^	NT^e^	NT^e^	NT^e^	NT^e^	NT^e^	2.3 ± 0.4	2.0 ± 0.3	NT^e^	NT^e^
cisplatin^d^	NT^e^	NT^e^	NT^e^	NT^e^	NT^e^	NT^e^	NT^e^	3.2 ± 0.5	NT^e^

a Positive control for hepatocellular carcinoma (Huh-7, HLE).

b Positive control for pancreatic carcinoma (PANC-1, MIA PaCa-2, KPC).

c Positive control for colorectal carcinoma (DLD-1, SW620).

d Positive control for lung carcinoma (PC-9).

e NT: Not tested.

**Table 4:** Selectivity Index Values of (±)-**1** and (±)-**2** ^a^ ^,^ ^b^

compound	selectivity Index (SI)^a^			
	Vero/Huh-7	Vero/MIA PaCa-2	Vero/DLD-1	Vero/SW620
**(+)-1**	**> 9.7**	>2.1	>1.3	>2.2
**(−)-1**	**> 10.3**	>2.5	>1.4	>2.7
**(+)-2**	>2.7	>1.4	ND^b^	>1.7
**(−)-2**	>3.1	>1.6	ND^b^	>1.7

a SI = IC_50_ for the normal cell line (Vero)/IC_50_ for the cancer cell line.

b ND: Not determined.

## Conclusions

In summary, two pairs of enantiomeric xeniaphyllane-type diterpenoids, (±)-sclerofish A (**1**) and (±)-sclerofish B (**2**), featuring a rare 4/7/6-fused tricyclic framework, were isolated from the soft coral *Sclerophytum humesi* by molecular networking-guided isolation. A plausible biogenetic pathway, originating from geranylgeranyl pyrophosphate (GGPP) was proposed to rationalize the formation of the unusual bicyclo­[4.3.1^4,8^]­decane subunit and the resulting rare 4/7/6-fused skeleton. Compound **1a**/**1b** exhibited inhibition of Huh-7 cells with IC_50_ values of 5.1 ± 0.9 and 4.9 ± 0.3 μM, respectively, and showed selective cytotoxicity toward cancer cells (SI > 9.7), whereas compound **2a**/**2b** displayed comparatively weaker activity. These results enrich the chemical space of marine diterpenoids and underscore the potential of structurally unique coral-derived metabolites as promising scaffolds for further biological and biosynthetic investigations.

## Experimental Section

### General Experimental Procedures

The purification and isolation of compounds from the extracts were performed using a series of chromatographic techniques. Fast column chromatography (FCC) was initially conducted using a SepaBean machine 2 (China), equipped with either silica gel, to facilitate preliminary fractionation. Subsequently, high-performance liquid chromatography (HPLC) was employed to purify the target constituents. Semipreparative HPLC was performed on a Shimadzu LC-2050 system (Shimadzu, Kyoto, Japan) using a Galaksil EF-C_18_–H reverse-phase column (120 Å, 5 μm, 10 × 250 mm, China) for the initial purification and separation of compounds based on hydrophobicity. A Daicel Chiralcel OD-R column (5 μm, 4.6 × 250 mm, China) for the enantioselective resolution of racemic constituents, enabling high-resolution isolation of the bioactive compounds. Structural elucidation and characterization of the isolated compounds were conducted using a comprehensive suite of spectroscopic techniques. Nuclear magnetic resonance (NMR) spectroscopy was performed on an Agilent 600 MHz DD2 NMR spectrometer (USA), incorporating both one-dimensional (1D) and two-dimensional (2D) experiments, with methanol-*d* used as the internal lock solvent to ensure spectral reliability. Structural assignments were made with additional information from gCOSY, gHSQC, gHMBC, and gNOESY experiments. High-resolution electrospray ionization mass spectrometry (HRESIMS) data were obtained utilizing a Shimadzu LC–MS 9030 (QTOF) mass spectrometer (Japan). Infrared spectra (IR) were recorded employing an IRAffinity-1S FTIR Spectrometer (Japan). Ultraviolet (UV) spectra were measured using a U-3310 UV–vis spectrophotometer (Japan). Electronic circular dichroism (ECD) spectra were obtained with a Jasco J-715 spectropolarimeter, and optical rotation values were determined in methanol using a Jasco P-2000 polarimeter (Japan). Huh-7 (CRL-185) (hepatocellular carcinoma), MIA PaCa-2 (CRL-1420), PANC-1 (CRL-1469) (pancreatic carcinoma), DLD-1 (CRL-221), and SW620 (CCL-227) (colorectal carcinoma) cancer cell lines were acquired from the American Type Culture Collection (ATCC). The normal cell line Vero (CCL-81) (monkey kidney epithelial) was acquired from the ATCC. The HLE (hepatocellular carcinoma), KPC (pancreatic carcinoma), and PC-9 (lung carcinoma) cancer cell lines were sourced from the Bioresource Collection and Research Centre in Taiwan.

### Biological Material

Specimens of the soft coral *S. humesi* (voucher specimen No. SH-2023) were collected by scuba diving at a depth of approximately 10–15 m off the coast of Pingtung County, Taiwan, in November 2023. The specimen was taxonomically identified as *S. humesi* by Dr. Bo-Rong Peng. To preserve the chemical constituents and structural integrity, the samples were immediately frozen upon collection. A voucher specimen No. SH-2023 has been deposited and archived in the herbarium of the College of Pharmacy, Taipei Medical University (Taipei, Taiwan).

### Extraction and Isolation

The frozen dry specimen of *S. humesi* (300 g) underwent thorough extraction using ethyl acetate (EtOAc) at room temperature. The resulting extracts were evaporated under reduced pressure to obtain a brown residue (SH-EA), which weighed 18.1 g. The residue SH-EA (18.1 g) was subjected to fractionation using silica gel-FCC with a stepwise gradient elution. The separation was performed at a flow rate of 20 mL/min over a period of 180 min, utilizing a solvent system composed of *n*-hexane (Hex) and EtOAc. The elution gradient gradually transitioned from Hex/EtOAc (100:0) to (0:100) to obtained 11 fractions (fractions 1–11).

To enable the targeted isolation of diterpenoid metabolites, a molecular networking (MN) strategy was applied using MS/MS spectral data processed on the GNPS platform. In our previous study, eight molecular families (clusters A-H) were annotated as terpenoid-enriched and served primarily for global chemical profiling. In this study, an in-depth reanalysis was focused on cluster A, in which two previously uncharacterized nodes were identified based on distinct MS/MS fragmentation features.^1c^ These nodes exhibited spectral connectivity to known xeniaphyllane-type diterpenoids, including sclerohumins G and H, as well as gibberosins G and H, indicating close structural relatedness within this molecular family (Figure 1, Table 1). Notably, both unassigned nodes were predominantly localized in fraction 7, thereby guiding subsequent isolation efforts.

Accordingly, fraction 7 (512.8 mg) was selected for further purification and subjected to reversed-phase HPLC using an isocratic elution system. The mobile phase consisted of a mixture of acidic water (0.1% formic acid) and acetonitrile (CH_3_CN) in a 40:60 ratio. Photodiode array (PDA) detection was carried out at wavelengths of 203 and 254 nm, resulting in the collection of four subfractions (7A-7D). Subfraction 7C (20.8 mg) was purified by reversed-phase chromatography on an EF-C_18_–H column under isocratic conditions, employing a mobile phase composed of 32% CH_3_CN in acidified water. This procedure afforded compound **1** (2.4 mg, *t*_R_ = 36.8 min) and **2** (2.2 mg, *t*_R_ = 38.1 min). Chiral separation of compound **1** was achieved by chiral HPLC using a Daicel Chiralcel OD-R column (5 μm, 4.6 × 250 mm, China) with MeOH/H_2_O (25:75) as the mobile phase at a flow rate of 1.0 mL/min, affording (+)-**1a** (1.2 mg, *t*_R_ = 126.3 min) and **(−)-1b** (1.0 mg, *t*_R_ = 135.7 min). Similarly, compound **2** was resolved using MeOH/H_2_O (38:62) as the mobile phase, yielding **(+)-2a** (0.95 mg, *t*_R_ = 71.1 min) and (−)-**2b** (1.0 mg, *t*_R_ = 75.6 min). PDA detection was conducted at 229 nm for both compounds.

### Compound Characterization

(±)-Sclerofish A (**1**): colorless oil; [α]_D_^25^ 0 (*c* 0.1, MeOH); UV (MeOH) λ_max_ (log ε) 229 (0.65) nm; IR ν_max_ (neat) 3356, 2970, 2924, 2870, 1674, 1627, 1458, 1373, 1311, 1265, 1234, 1157 cm^–1^; HRMS (ESI) *m*/*z* [M + H]^+^ Calcd for C_20_H_33_O_4_ 337.2373; Found 337.2362. ^1^H (600 MHz, CD_3_OD) and ^13^C­{^1^H} (150 MHz, CD_3_OD) NMR data are presented in Table 2.

*(+)-Sclerofish A* (**1a**): [α]_D_^25^ +27.2 (*c* 0.1, MeOH); ECD (*c* 0.1 × 10^–3^ M, MeOH) λ_max_ (Δε) 208 (−8.74), 224 (+36.42), 323 (+2.93) nm.

*(−)-Sclerofish A* (**1b**): [α]_D_^25^ −27.0 (*c* 0.1, MeOH); ECD (*c* 0.1 × 10^–3^ M, MeOH) λ_max_ (Δε) 204 (+10.29), 223 (−40.03), 225 (−2.32) nm.

(±)-Sclerofish B (**2**): colorless oil; [α]_D_^25^ +0.2 (*c* 0.1, MeOH); UV (MeOH) λ_max_ (log ε) 229 (0.69) nm; IR ν_max_ (neat) 3332, 2978, 2870, 1635, 1450, 1373, 1319, 1273, 1211, 1157, 1095, 1054 cm^–1^; HRMS (ESI) *m*/*z* [M + H]^+^ Calcd for C_20_H_33_O_4_ 337.2373; Found 337.2382. ^1^H (600 MHz, CD_3_OD) and ^13^C­{^1^H} (150 MHz, CD_3_OD) NMR data are presented in Table 2.

*(+)-Sclerofish B* (**2a**): [α]_D_^25^ +34.8 (*c* 0.1, MeOH); ECD (*c* 0.1 × 10^–3^ M, MeOH) λ_max_ (Δε) 206 (−11.13), 227 (+46.35), 326.2 (+3.37) nm.

*(−)-Sclerofish B* (**2b**): [α]_D_^25^ −34.3 (*c* 0.1, MeOH); ECD (*c* 0.1 × 10^–3^ M, MeOH) λ_max_ (Δε) 207 (+12.41), 227 (−37.71), 317 (−8.43) nm.

### Fragment Ion Acquisition via UPLC-MS/MS Analysis

Fragmentation data (MS^2^) were acquired using a Shimadzu LCMS-9030 system following chromatographic separation on a C_18_ column of Shimadzu Shim-pack GIST (2 μm, 2.1 mm × 100 mm) at 40 °C and a flow rate of 0.4 mL/min. The mobile phases comprised methanol (M) and water (W), both containing 0.1% formic acid, with a programmed gradient arrangement: 0–7 min, 10–40% M; 7–15 min, 40–100% M; 15–20 min, 100% M. Fractions (500 ppm) were prepared in methanol, filtered (0.22 μm), and injected (5 μL) via an autosampler. MS data (*m*/*z* 100–1800) were acquired in DDA mode with collision energy ramping (35 eV) for five nontargeted precursor ions. Data processing was performed using LabSolutions (LabSolutions 5.12, Shimadzu).^1c^

### Molecular Networking Analysis (MN)

MN was performed via the Global Natural Product Social Molecular Networking (GNPS) platform (job ID: 6392eaa2535b4ba2b239c149f812acb0, accessed June 21, 2025; https://gnps.ucsd.edu/). MS/MS spectra were processed using window filtering (±50 Da), retaining the top five most intense ions per window. Network construction required at least four shared peaks and a cosine similarity score exceeding 0.70. The resulting MN, annotated based on MS^2^ fragmentation patterns, was visualized using Cytoscape 3.8.2 (NRNB, USA).^1c,8^

### NMR and ECD Calculations

NMR and ECD computational analyses were conducted on compounds **1** and **2** studied in this investigation. Initially, conformers for each stereochemical configuration were generated using the GMMX method implemented in GaussView 6.1 (USA). These conformers underwent geometry optimization and vibrational frequency analysis at the B3LYP/6-31G­(d,p) level of theory, incorporating MeOH as the solvent, using Gaussian 16 software. All conformers, confirmed as true minima, showed no imaginary frequencies. The Gibbs free energy for each conformer was computed to assess their Boltzmann populations, and those with populations below 2% were omitted from further analysis. Remaining conformers were used for NMR chemical shift calculations via Gauge-Including Atomic Orbital (GIAO) DFT at the *m*PW1PW91/6-311+G­(d,p) level with the IEFPCM model for MeOH, applying the DP4+ prediction method.^1c,9^ Unscaled chemical shifts (δ_u_) were obtained relative to tetramethylsilane (TMS), using the equation δ_u_ = σ_0_ – σ*_x_*, where σ*x* is the Boltzmann-averaged shielding tensor of all significant conformers, and σ_0_ is the TMS shielding tensor computed at the same level. The averaged chemical shifts, weighted by conformer populations, were analyzed with Excel and the DP4+ approach. For ECD calculations, the excitation energies of the lowest 30 electronic states were obtained using TDDFT/B3LYP/6-311G++(d,p) with the IEFPCM­(MeOH) solvent model in Gaussian 16.^1c,1d^ ^,^ ^9a^ The overall ECD spectra for all conformers were then generated by weighting according to their Boltzmann distributions, employing SpecDis 1.71 with a broadening parameter (σ) of 0.30 eV.^1c,1d,9a,9d^ The computed spectra subsequently were analyzed alongside the experimental CD measurements, which were recorded over a wavelength range of 200–400 nm, consisting of 301 data points, with CD values expressed in millidegrees (mdeg).^1c,10^

### Cell Viability Assay

The experimental protocols and analytical methodologies were adapted from previously published studies, incorporating minor modifications to the tested concentration ranges.^1d,11^ Upon reaching roughly 80% confluence, the cells were rinsed with phosphate-buffered saline (PBS) and detached using 0.25% trypsin-0.02% EDTA (Gibco) at 37 °C for 5 min. The detached cells were then transferred into 96-well plates at a density of 3 × 10^4^ cells per well in 100 μL of fresh medium and incubated at 37 °C with 5% CO_2_ for 24 h. Next, 100 μL of fresh medium containing the test samples was added to each well, and the cells were further incubated for an extra 72 h. Cell viability was evaluated using the Cell Counting Kit-8 (CCK-8) assay according to the manufacturer’s instructions. Absorbance was read at 450 nm with a microplate reader. Cell viability was calculated using the following formula:

$$\left(\%\right)\;Cell\;viability=\left[\left({Abs}_{sample}-{Abs}_{blank}\right)/\left({Abs}_{control}-{Abs}_{blank}\right)\right]\times 100\%$$

Cytotoxicity assays were conducted in triplicate (*n* = 3). Compounds exhibiting greater than 50% growth inhibition were subsequently evaluated for IC_50_ determination using GraphPad Prism version 8.0.2.^12^ Sorafenib, gemcitabine, and regorafenib were employed as a positive control in these assays.^1c^

### Statistical Analysis

Statistical evaluations for the in vitro studies were performed using GraphPad Prism version 8.0.2. Results are presented as the mean ± standard deviation (SD). The data were analyzed through a one-way analysis of variance (ANOVA), with subsequent Tukey’s post hoc test for multiple comparisons. A *P*-value below 0.05 was regarded as statistically significant.

## Supplementary Material



## Data Availability

The data underlying this study are available in the published article and its Supporting Information.

## Notes

Detailed experimental procedures, computational calculations, and NMR, HRESIMS, UV, and IR spectra of compounds **1** and **2** (PDF)
