# Supplementary material for: Sclerofish A and B: Two Pairs of Enantiomeric Diterpenoids Featuring a 4/7/6-Fused Tricyclic Scaffold from the Soft Coral Sclerophytum humesi
Source: J Org Chem. 2026 Jun 1;91(24):8230–40. doi: 10.1021/acs.joc.6c00512 (PMC13288667; doi:10.1021/acs.joc.6c00512)
Supplement: Supplementary file 1 [file jo6c00512_si_001.pdf]

Supporting Information

Sclerofish A and B: Two Pairs of Enantiomeric Diterpenoids Featuring a 4/7/6-Fused Tricyclic Scaffold from the Soft Coral *Sclerophytum humesi*

Phuong Vu Luu, Thuy-Tien Thi Phan, Quoc-Dung Tran Huynh, Ngoc-Thac Pham, Huong-Giang Le, Lo-Yun Chen, Huong Lien Ton-Nu, Cuong-Quoc Nguyen, Yao-An Shen, Yu-Jui Fan, Jui-Hsin Su, Bo-Rong Peng*, Kuei-Hung Lai*

***Corresponding Author**

Bo-Rong Peng - *Graduate Institute of Pharmacognosy, College of Pharmacy, Taipei Medical University, Taipei 110301, Taiwan; PhD Prosgram in Clinical Drug Development of Herbal Medicine, College of Pharmacy, Taipei Medical University, Taipei 110301, Taiwan; Graduate Institute of Healthy Industry Technology, Center for Drug Research and Development, College of Human Ecology, Chang Gung University of Science and Technology, Taoyuan 333324, Taiwan.* Email: brpeng@mail.cgust.edu.tw

Kuei-Hung Lai - *Graduate Institute of Pharmacognosy, College of Pharmacy, Taipei Medical University, Taipei 110301, Taiwan; PhD Program in Clinical Drug Development of Herbal Medicine, College of Pharmacy, Taipei Medical University, Taipei 110301, Taiwan; Traditional Herbal Medicine Research Center, Taipei Medical University Hospital, Taipei 110301, Taiwan; National Museum of Marine Biology and Aquarium, Pingtung 944401, Taiwan; PhD Program in Drug Discovery and Development Industry, College of Pharmacy, Taipei Medical University, Taipei 110301, Taiwan;* Email: kueihunglai@tmu.edu.tw

Authors

Phuong Vu Luu - *Graduate Institute of Pharmacognosy, College of Pharmacy, Taipei Medical University, Taipei 110301, Taiwan.* https://orcid.org/0009-0009-0383-1815

Thuy-Tien Thi Phan - *Graduate Institute of Biomedical Materials and Tissue Engineering, College of Biomedical Engineering, Taipei Medical University, Taipei 110301, Taiwan; Institute of Pharmaceutical Education and Research, Binh Duong University, Thu Dau Mot, Binh Duong 820000, Vietnam.*

Quoc-Dung Tran Huynh - *Institute of Pharmaceutical Education and Research, Binh Duong University, Thu Dau Mot, Binh Duong 820000, Vietnam; Institute of Biological Chemistry, Academia Sinica, Taipei 115024, Taiwan.*

Ngoc-Thac Pham - *Department of Pharmacognosy and Traditional Pharmacy, School of Pharmacy, University of Medicine and Pharmacy at Ho Chi Minh City, Ho Chi Minh City 700000, Vietnam; PhD Program in Clinical Drug Development of Herbal Medicine, College of Pharmacy, Taipei Medical University, Taipei 110301, Taiwan.*

Huong-Giang Le - *Department of Pharmacognosy and Traditional Pharmacy, School of Pharmacy, University of Medicine and Pharmacy at Ho Chi Minh City, Ho Chi Minh City 700000, Vietnam.*

Lo-Yun Chen - *PhD Program in Clinical Drug Development of Herbal Medicine, College of Pharmacy, Taipei Medical University, Taipei 110301, Taiwan.*

Huong Lien Ton-Nu - *Department of Chemistry, College of Natural Sciences, Can Tho University, Can Tho 94000, Vietnam.*

Cuong-Quoc Nguyen - *Department of Health Sciences, College of Natural Sciences, Can Tho University, Can Tho 94000, Vietnam.*

Yao-An Shen - *Department of Pathology, School of Medicine, College of Medicine, Taipei Medical University, Taipei 110301, Taiwan; Graduate Institute of Clinical Medicine, College of Medicine, Taipei Medical University, Taipei 110301, Taiwan; International Master/Ph.D. Program in Medicine, College of Medicine, Taipei Medical University, Taipei 110301, Taiwan.*

Yu-Jui Fan - *National Yang Ming Chiao Tung University, Department of Mechanical Engineering, Hsinchu 300093, Taiwan.*

Jui-Hsin Su - *National Museum of Marine Biology & Aquarium, Pingtung 94450, Taiwan; Department of Marine Biotechnology and Resources, National Sun Yat-sen University, Kaohsiung 804, Taiwan; Graduate Institute of Marine Biology, National Dong Hwa University, Pingtung 94450, Taiwan.*

Contents

1. Spectra data for compounds 1 and 2 .... S8

1.1. NMR, HRESIMS, IR, and UV spectra of sclerofish A (1)........ S8

1.2. NMR, HRESIMS, IR, and UV spectra of sclerofish B (2) S19

2. Chiral HPLC analysis S32

3. Computational details S33

3.1. Structures of isomers studied S33

3.2. Gibbs free energy of the conformers and the Boltzmann distribution S34

3.3. The cartesian coordinates of the dominant conformers of compounds 1 and 2 .... S44

3.4. DP4+ results of compounds 1 and 2........ S131

3.5. Correlation plots of compounds 1 and 2 .... S133

Table legend

Table S1. 2D NMR data for compound **1** S30

Table S2. 2D NMR data for compound **2** S31

Table S3. Gibbs free energy of the conformers and the Boltzmann distribution for **1**-5*S*.......... S34

Table S4. Gibbs free energy of the conformers and the Boltzmann distribution for **1**-5*R*. S35

Table S5. Gibbs free energy of the conformers and the Boltzmann distribution for **2**-1*R*3*R*9*R*. S36

Table S6. Gibbs free energy of the conformers and the Boltzmann distribution for **2**-1*R*3*R*9*S*. S37

Table S7. Gibbs free energy of the conformers and the Boltzmann distribution for **2**-1*R*3*S*9*R*. S38

Table S8. Gibbs free energy of the conformers and the Boltzmann distribution for **2**-1*R*3*S*9*S*. S39

Table S9. Gibbs free energy of the conformers and the Boltzmann distribution for **2**-1*S*3*R*9*R*….S40

Table S10. Gibbs free energy of the conformers and the Boltzmann distribution for **2**-1*S*3*R*9*S*……………………………………………………………………………………..S41

Table S11. Gibbs free energy of the conformers and the Boltzmann distribution for **2**-1*S*3*S*9*R*…………………………………………………………………………………......S42

Table S12. Gibbs free energy of the conformers and the Boltzmann distribution for **2**-1*S*3*S*9*S*………………………………………………………………………………………43

Table S13. The cartesian coordinates of the dominant conformers for compound **1**……………………………………………………………………………………………..44

Table S14. The cartesian coordinates of the dominant conformers for compound **2**……………………………………………………………………………………………. 63

Figure legend

Figure S1. ^{1}H NMR spectrum of sclerofish A (**1**) (600 MHz, CD_3OD). S8
Figure S2. Expanded ^{1}H NMR spectrum of sclerofish A (**1**) (600 MHz, CD_3OD). S9
Figure S3. ^{13}C NMR spectrum of sclerofish A (**1**) (150 MHz, CD_3OD). S10
Figure S4. DEPT spectrum of sclerofish A (**1**) (150 MHz, CD_3OD). S11
Figure S5. HSQC spectrum of sclerofish A (**1**) (600 and 150 MHz, CD_3OD). S12
Figure S6. HMBC spectrum of sclerofish A (**1**) (600 and 150 MHz, CD_3OD). S13
Figure S7. COSY spectrum of sclerofish A (**1**) (600 MHz, CD_3OD). S14
Figure S8. NOESY spectrum of sclerofish A (**1**) (600 MHz, CD_3OD). S15
Figure S9. HRESIMS spectrum of sclerofish A (**1**). S16
Figure S10. Infrared (IR) spectrum sclerofish A (**1**). S17
Figure S11. Ultraviolet (UV) spectrum sclerofish A (**1**). S18
Figure S12. ^{1}H NMR spectrum of sclerofish B (**2**) (600 MHz, CD_3OD). S19
Figure S13. Expanded ^{1}H NMR spectrum of sclerofish B (**2**) (600 MHz, CD_3OD). S20
Figure S14. ^{13}C NMR spectrum of sclerofish B (**2**) (150 MHz, CD_3OD). S21
Figure S15. DEPT spectrum of sclerofish B (**2**) (150 MHz, CD_3OD). S22
Figure S16. HSQC spectrum of sclerofish B (**2**) (600 and 150 MHz, CD_3OD). S23
Figure S17. HMBC spectrum of sclerofish B (**2**) (600 and 150 MHz, CD_3OD). S24
Figure S18. COSY spectrum of sclerofish B (**2**) (600 MHz, CD_3OD). S25
Figure S19. NOESY spectrum of sclerofish B (**2**) (600 MHz, CD_3OD). S26
Figure S20. HRESIMS spectrum of sclerofish B (**2**). S27
Figure S21. Infrared (IR) spectrum sclerofish B (**2**). S28
Figure S22. Ultraviolet (UV) spectrum sclerofish B (**2**). S29
Figure S23. Chiral HPLC analysis chromatography of compound **1** S32
Figure S24. Chiral HPLC analysis chromatography of compound **2** S32
Figure S25. Structures of isomers **1a′** and **1b′** of compound **1** S33
Figure S26. Structures of isomers **2a′-2h′** of compound **2**. S33
Figure S27. The cartesian coordinates of the dominant conformers for conformers **1**-5*S*. S61
Figure S28. The cartesian coordinates of the dominant conformers for conformers **1**-5*R*. S62
Figure S29. The cartesian coordinates of the dominant conformers for conformers **2**-1*R*3*R*9*R*…………………………………………………………………………………..….S123

Figure S30. The cartesian coordinates of the dominant conformers for conformers **2**-1*R*3*R*9*S*………… S124

Figure S31. The cartesian coordinates of the dominant conformers for conformers **2**-1*R*3*S*9*R*………… S125

Figure S32. The cartesian coordinates of the dominant conformers for conformers **2**-1*R*3*S*9*S*…………….. S126

Figure S33. The cartesian coordinates of the dominant conformers for conformers **2**-1*S*3*R*9*R*……………….. S127

Figure S34. The cartesian coordinates of the dominant conformers for conformers **2**-1*S*3*R*9*S*…………….. S128

Figure S35. The cartesian coordinates of the dominant conformers for conformers **2**-1*S*3*S*9*R*……………………. S129

Figure S36. The cartesian coordinates of the dominant conformers for conformers **2**-1*S*3*S*9*S*…………………. S130

Figure S37. DP4+ results obtained using experimental data of **1** *versus* isomers 1 and 2....... S131

Figure S38. DP4+ results obtained using experimental data of **2** *versus* isomers 1-8. .... S132

Figure S39. Linear correlations of the calculated isomers of **1** with the experimentally observed ^{13}C NMR chemical shifts. S133

Figure S40. Linear correlations of the calculated isomers of **2** with the experimentally observed ^{13}C NMR chemical shifts. S134

1. Spectra data for compounds 1 and 2

1.1. NMR, HRESIMS, IR, and UV spectra of sclerofish A (1)

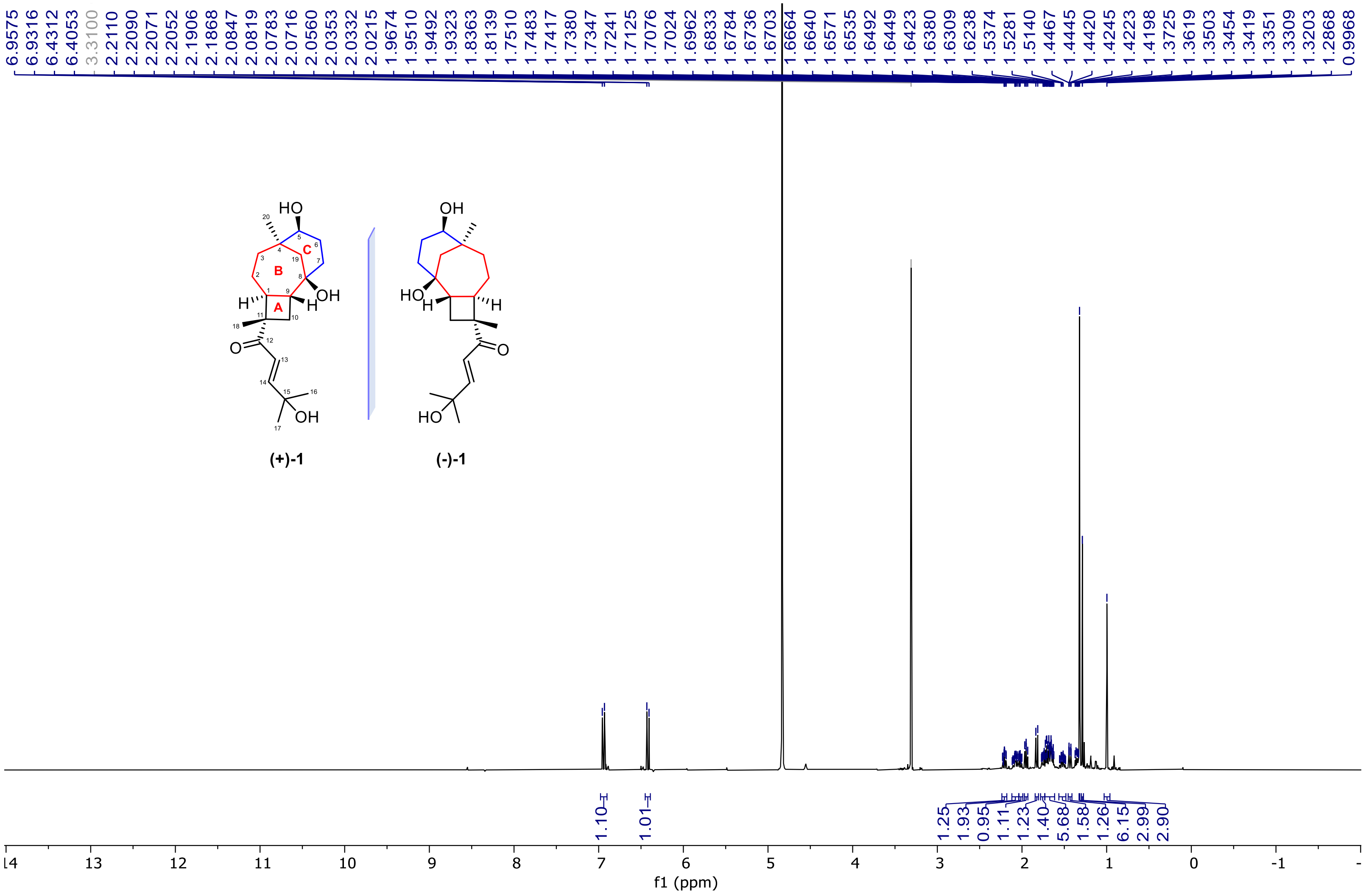

Figure S1. ^{1}H NMR spectrum of sclerofish A (**1**) (600 MHz, CD_3OD).

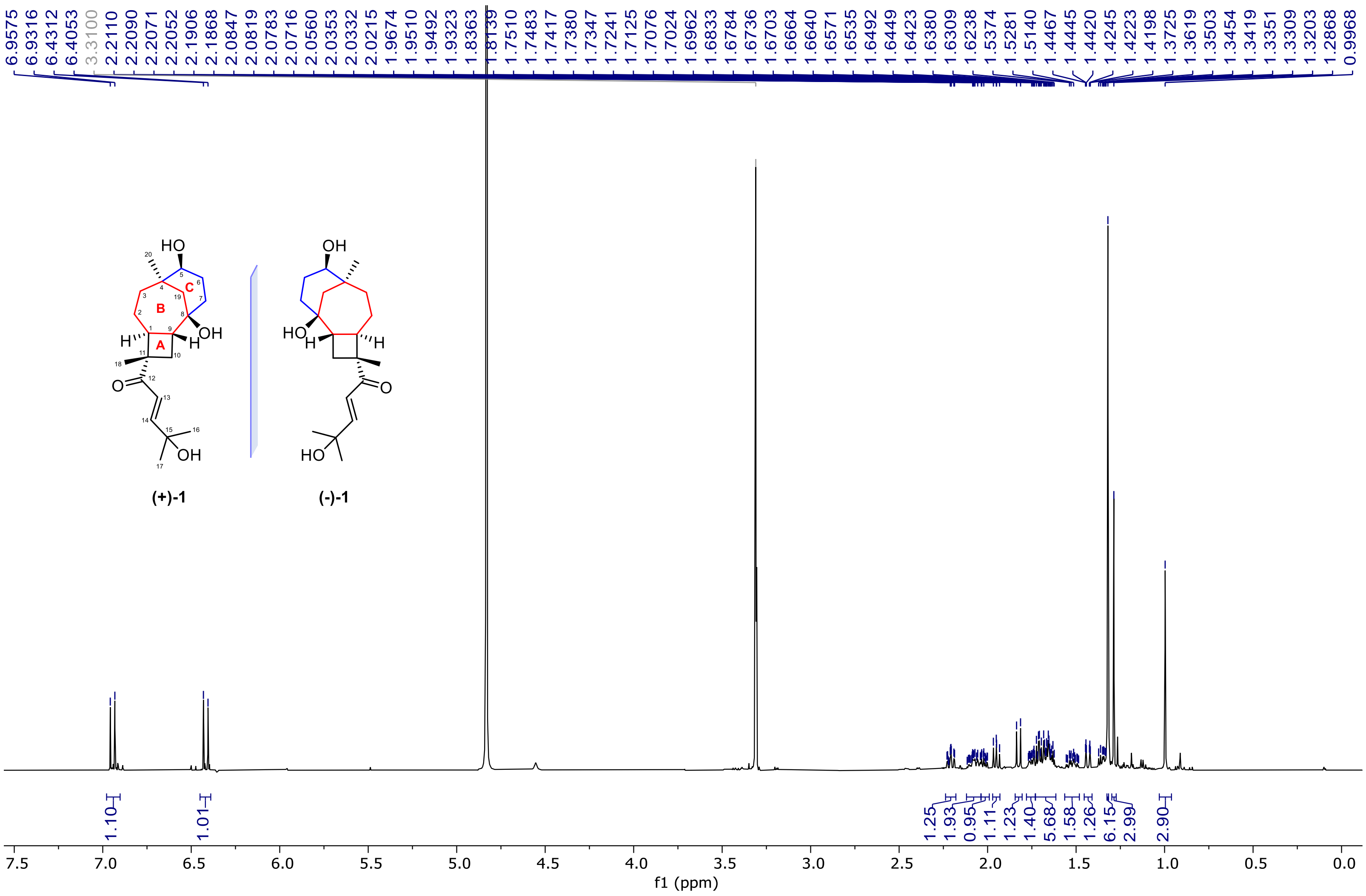

Figure S2. Expanded ^{1}H NMR spectrum of sclerofish A (**1**) (600 MHz, CD_3OD).

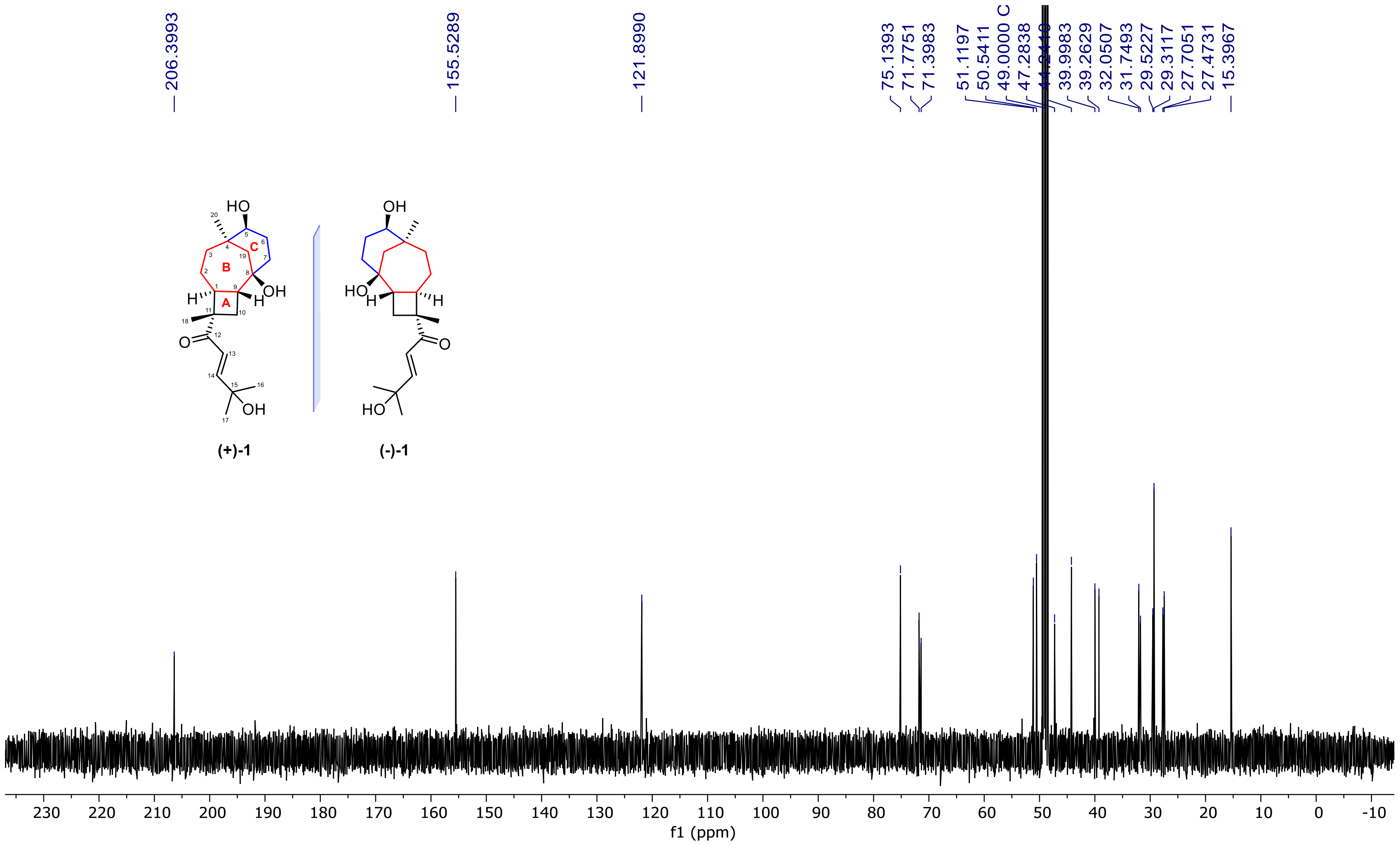

Figure S3. ^{13}C NMR spectrum of sclerofish A (**1**) (150 MHz, CD_3OD).

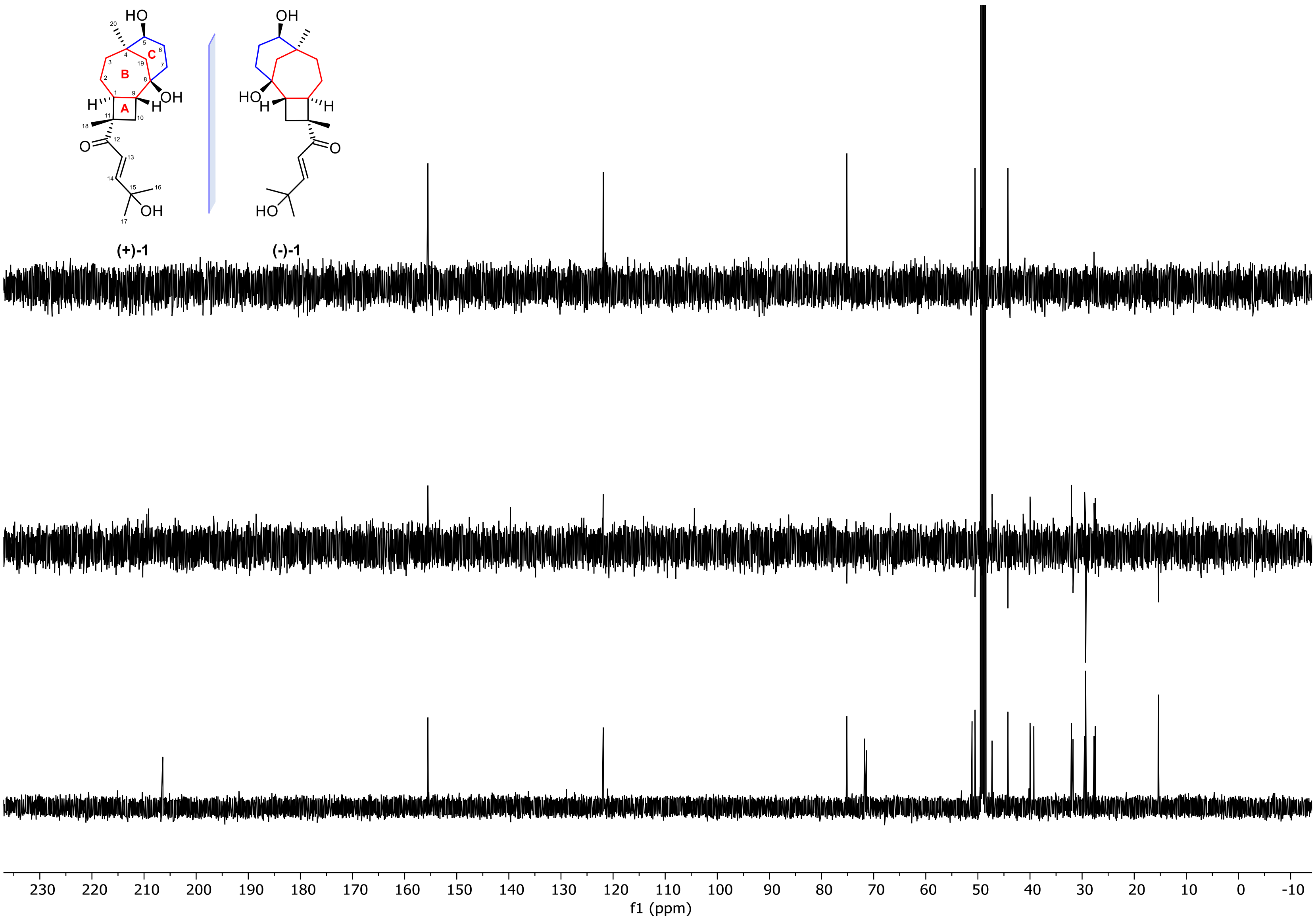

Figure S4. DEPT spectrum of sclerofish A (**1**) (150 MHz, CD_3OD).

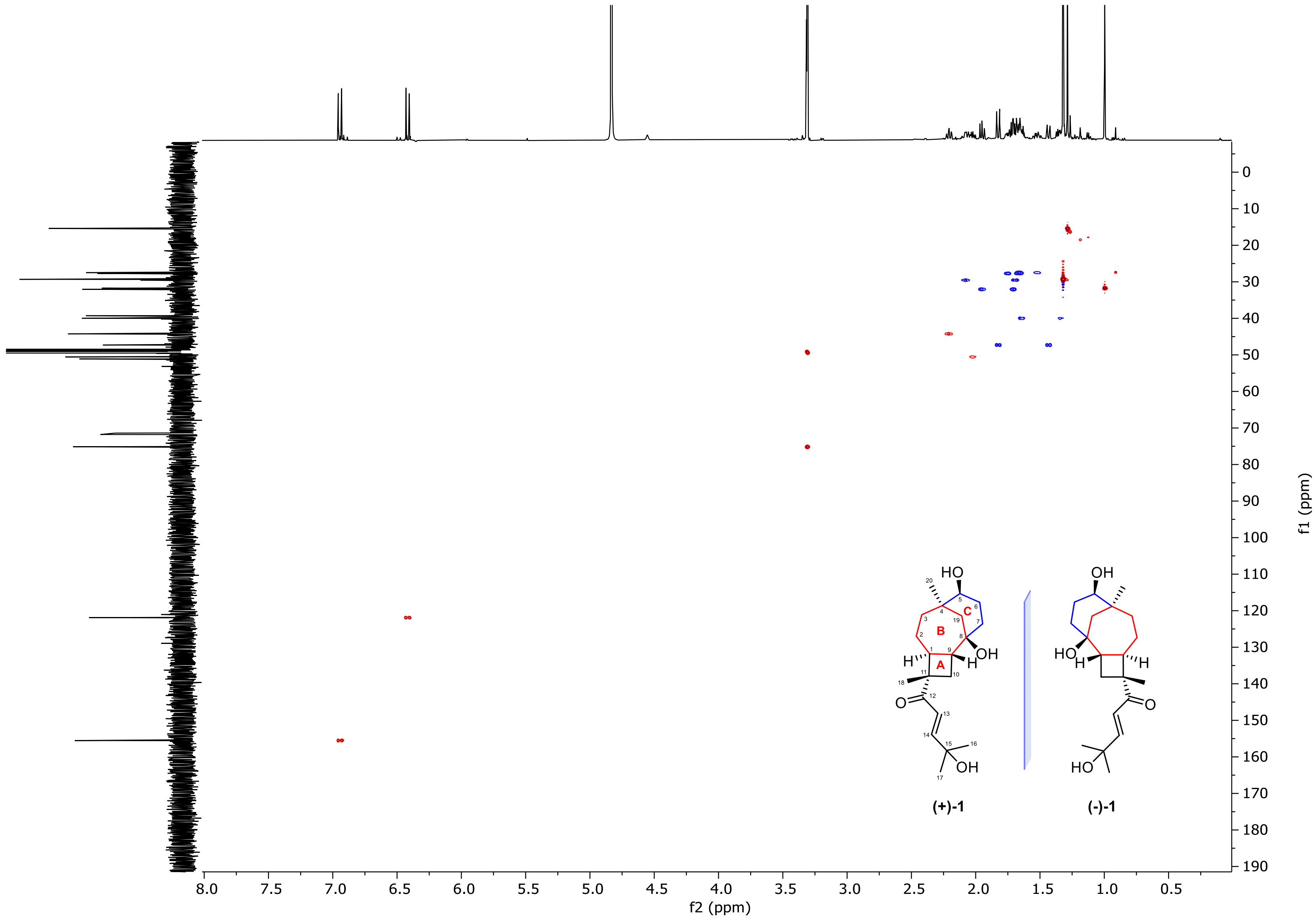

Figure S5. HSQC spectrum of sclerofish A (**1**) (600 and 150 MHz, CD_3OD).

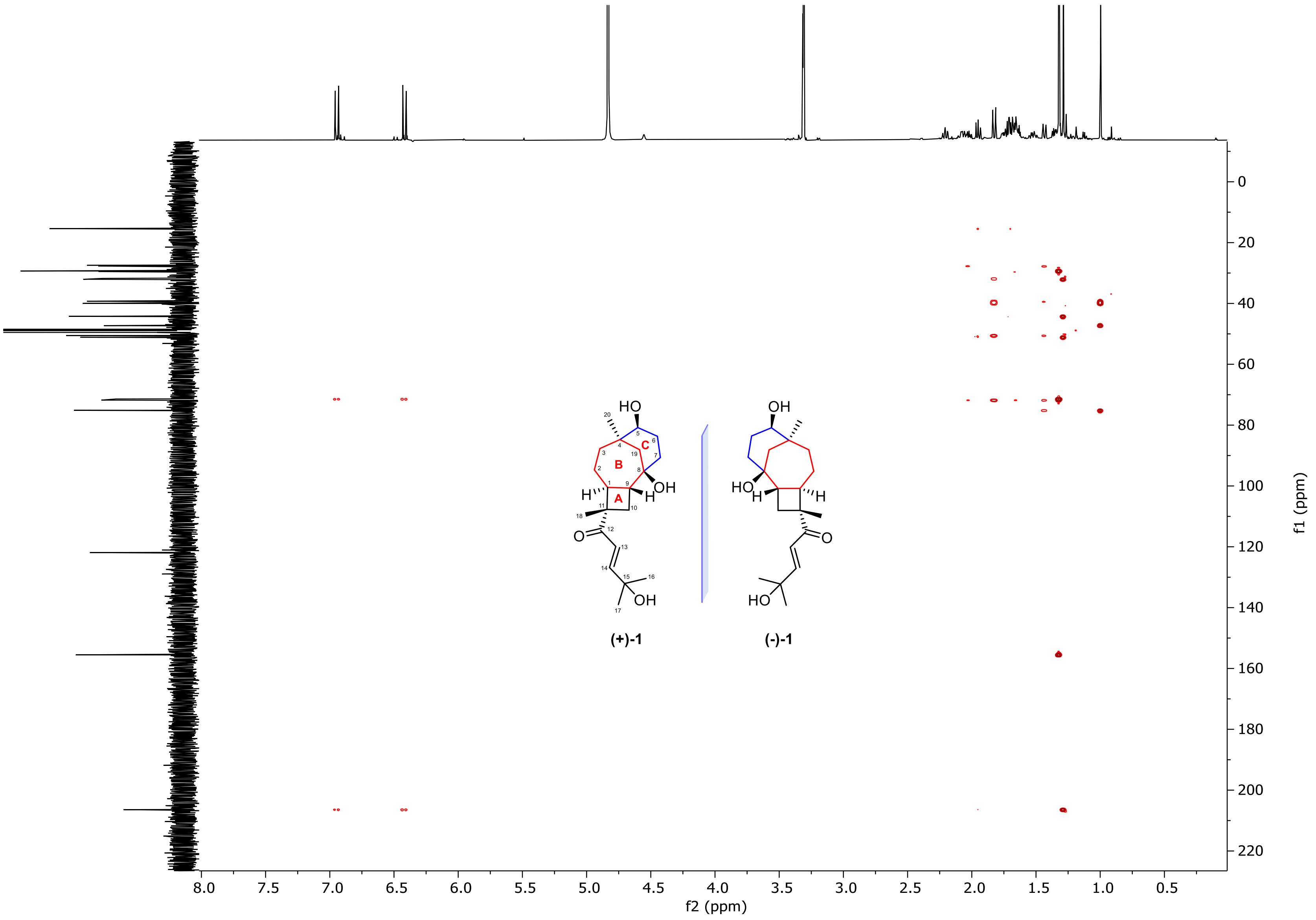

Figure S6. HMBC spectrum of sclerofish A (**1**) (600 and 150 MHz, CD_3OD).

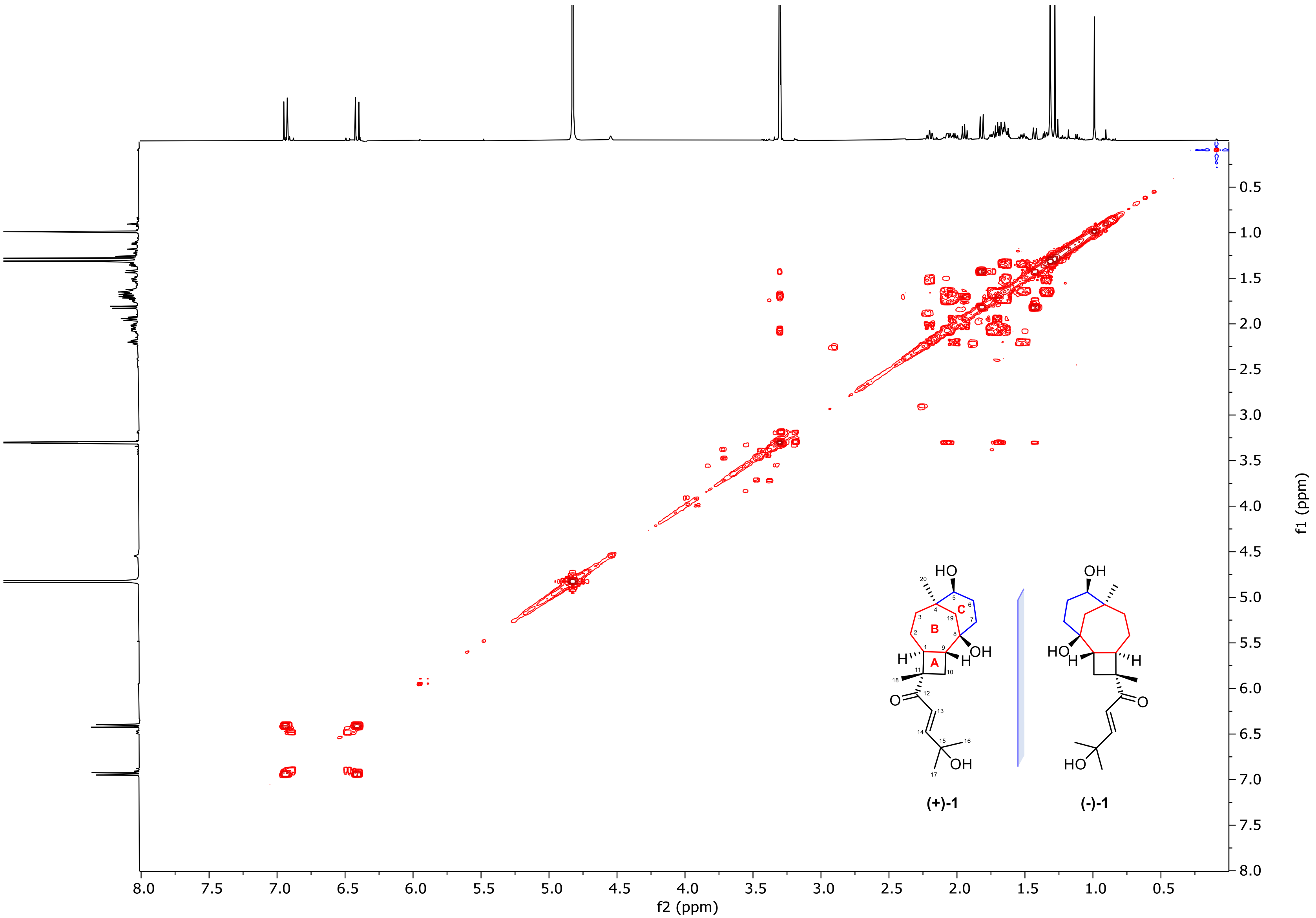

Figure S7. COSY spectrum of sclerofish A (**1**) (600 MHz, CD_3OD).

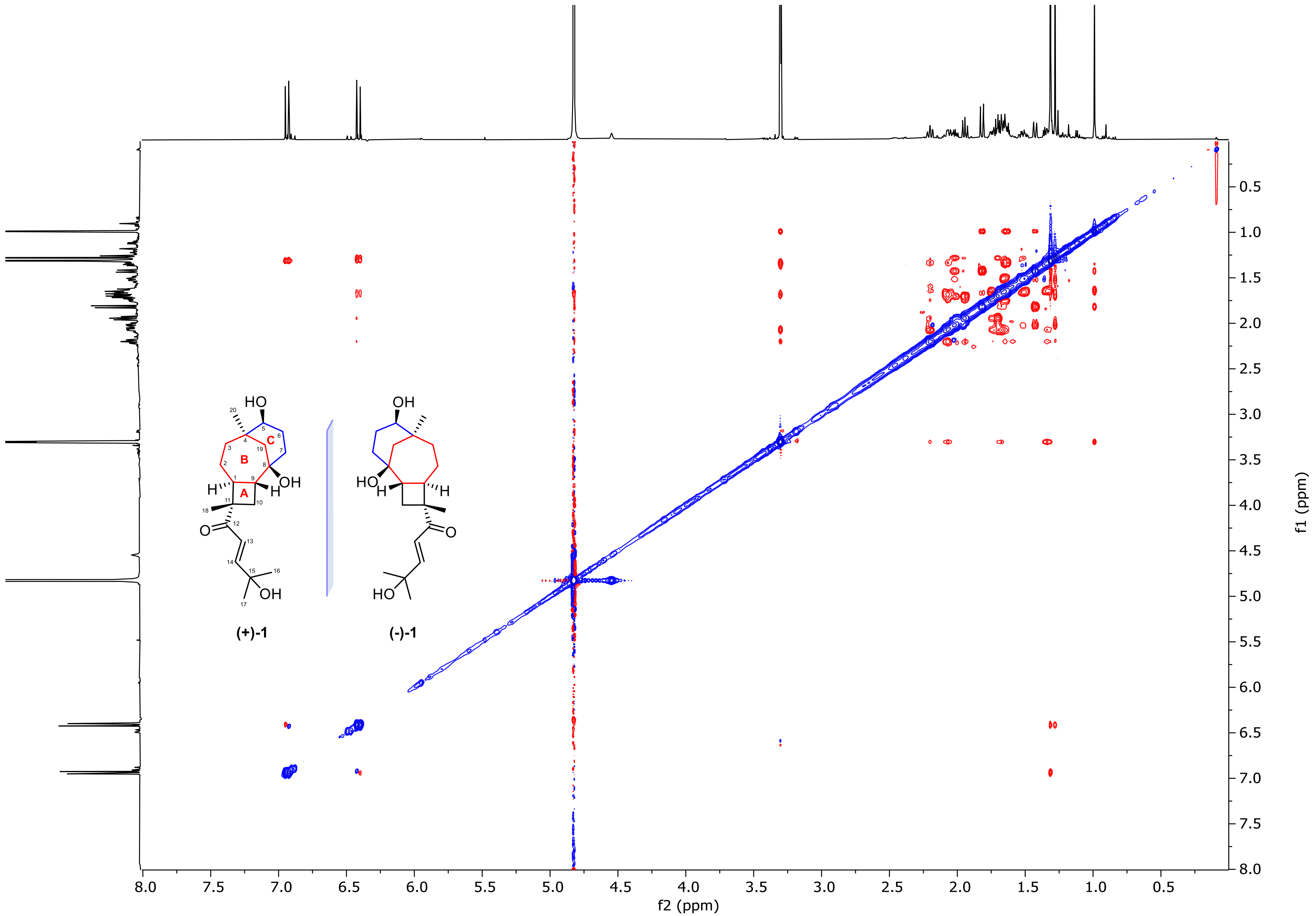

Figure S8. NOESY spectrum of sclerofish A (**1**) (600 MHz, CD_3OD).

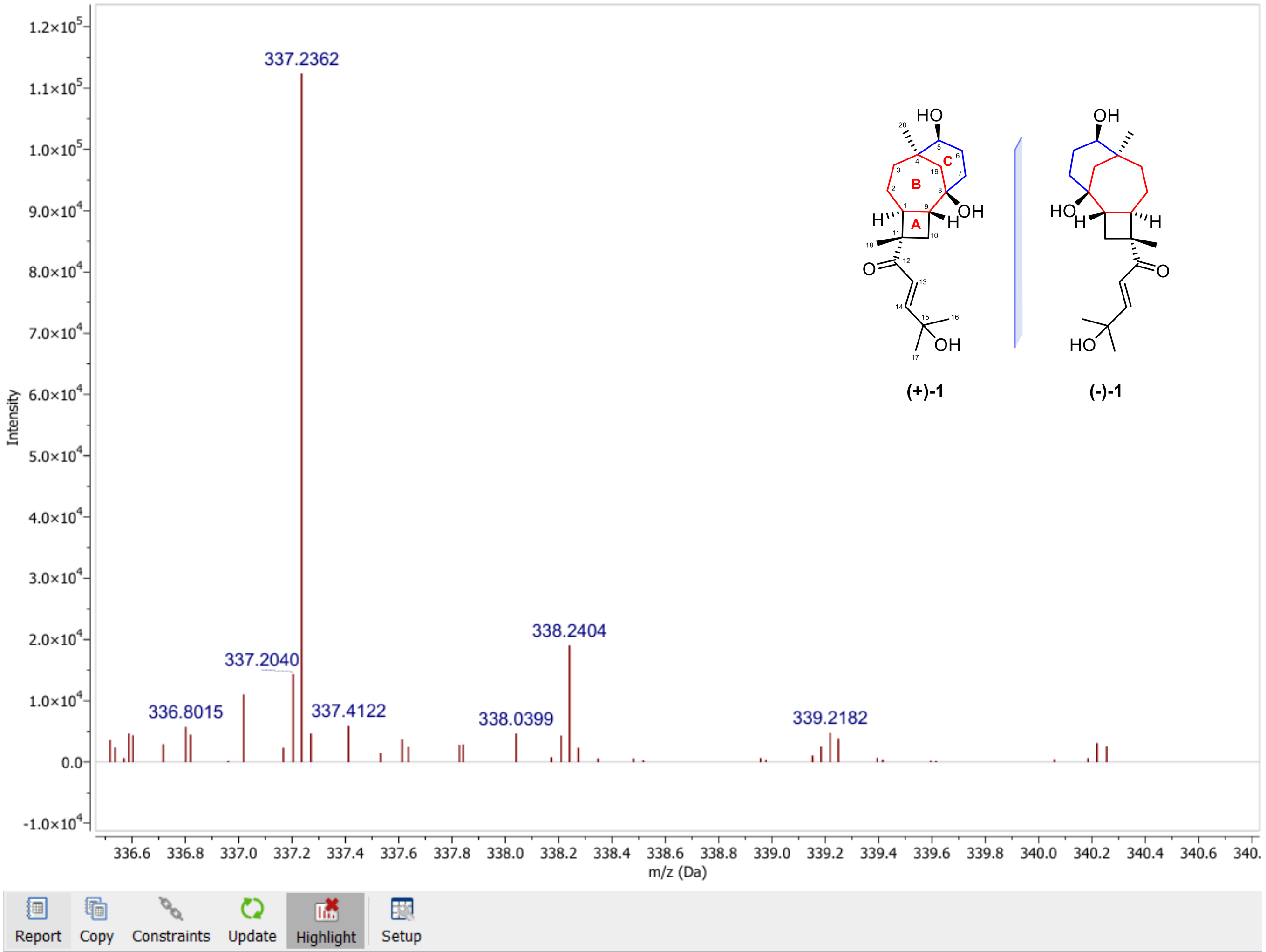

	Formula	Calculated Mass	Target Mass	Double Bond Equivalence	Absolute Error (ppm)	Error (mDa)	Error (ppm)	Fitness
1	$C_{20}H_{33}O_4$	337.23734	337.23624	4.5	3.24	-1.09	-3.24	0.993

Figure S9. HRESIMS spectrum of sclerofish A (**1**).

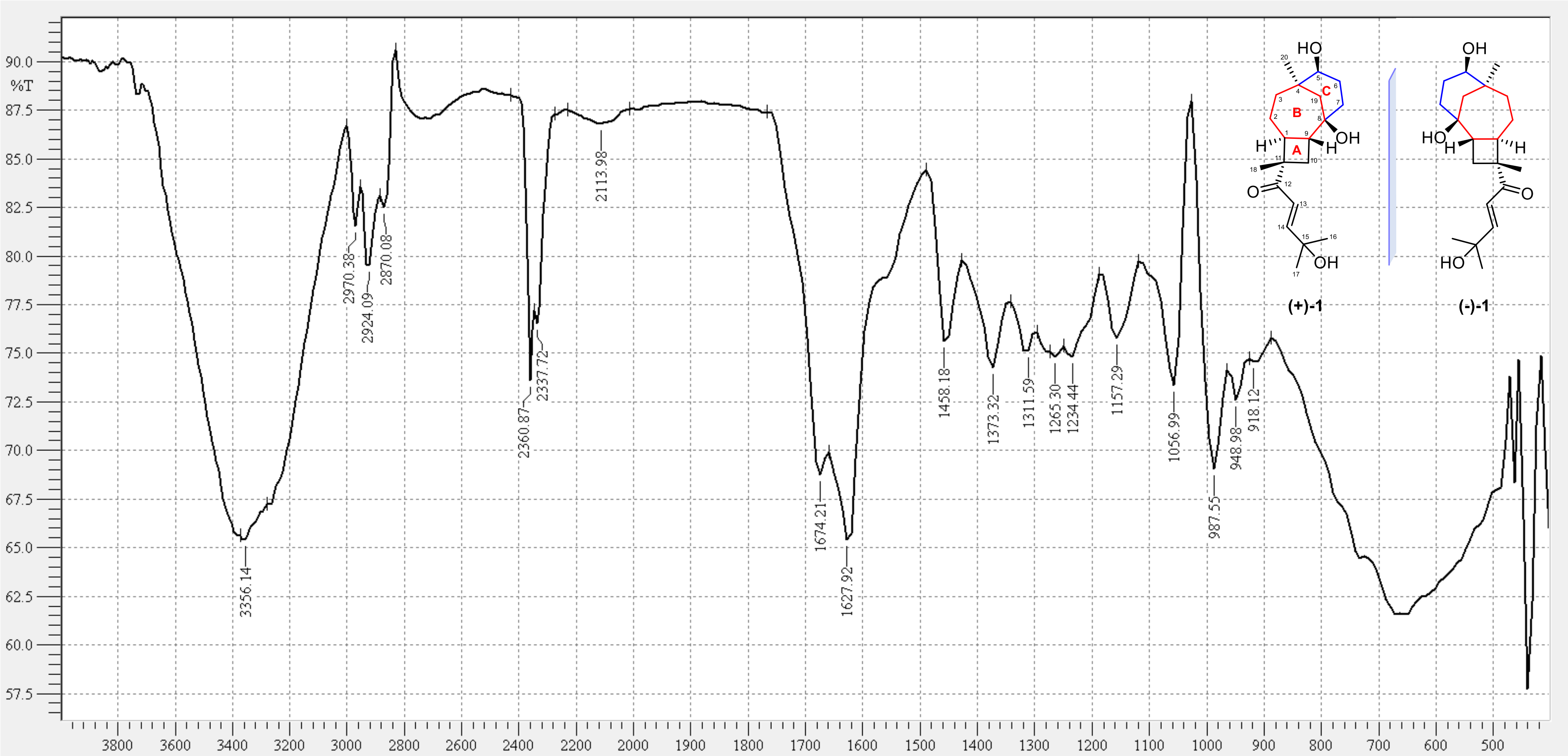

Figure S10. Infrared (IR) spectrum sclerofish A (**1**).

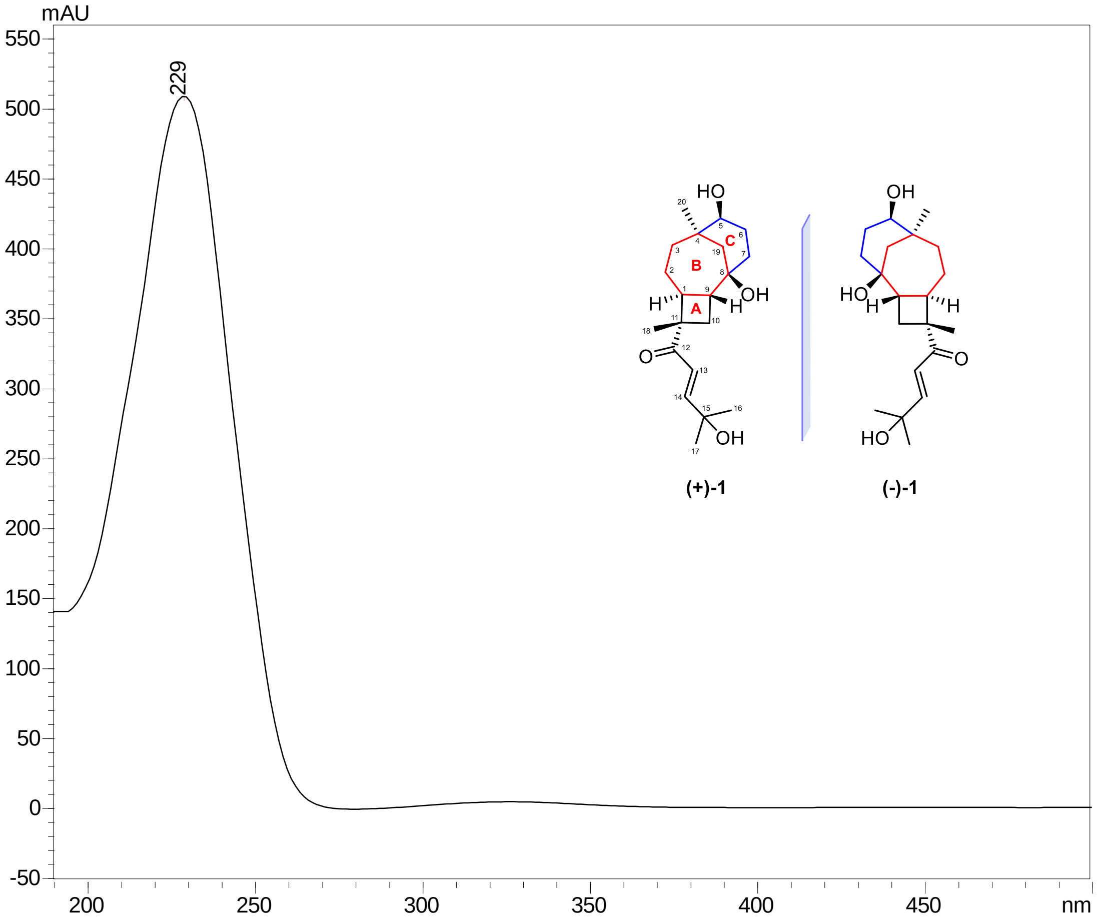

Figure S11. Ultraviolet (UV) spectrum sclerofish A (**1**).

1.2. NMR, HRESIMS, IR, and UV spectra of sclerofish B (2)

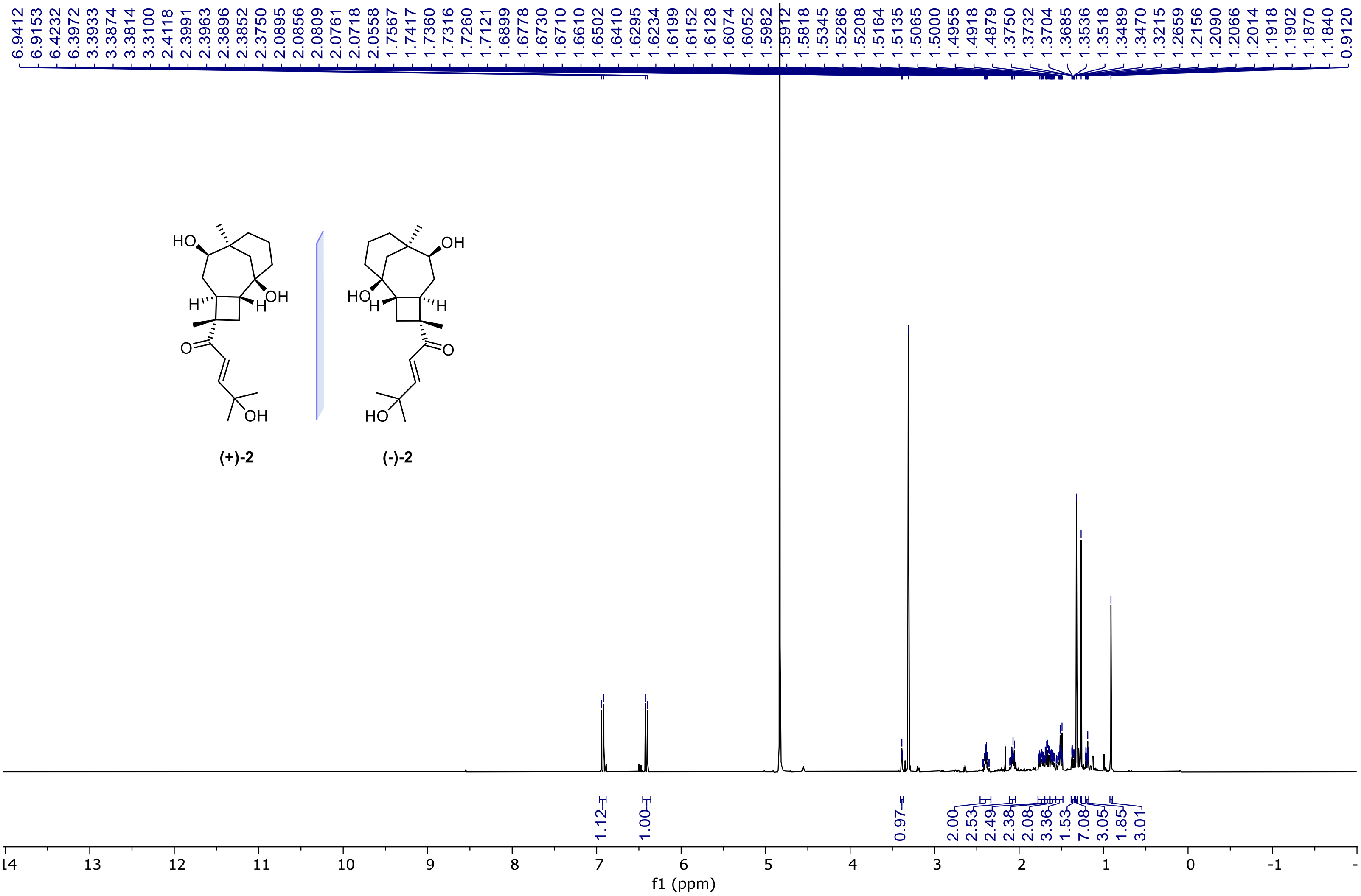

Figure S12. ^{1}H NMR spectrum of sclerofish B (**2**) (600 MHz, CD_3OD).

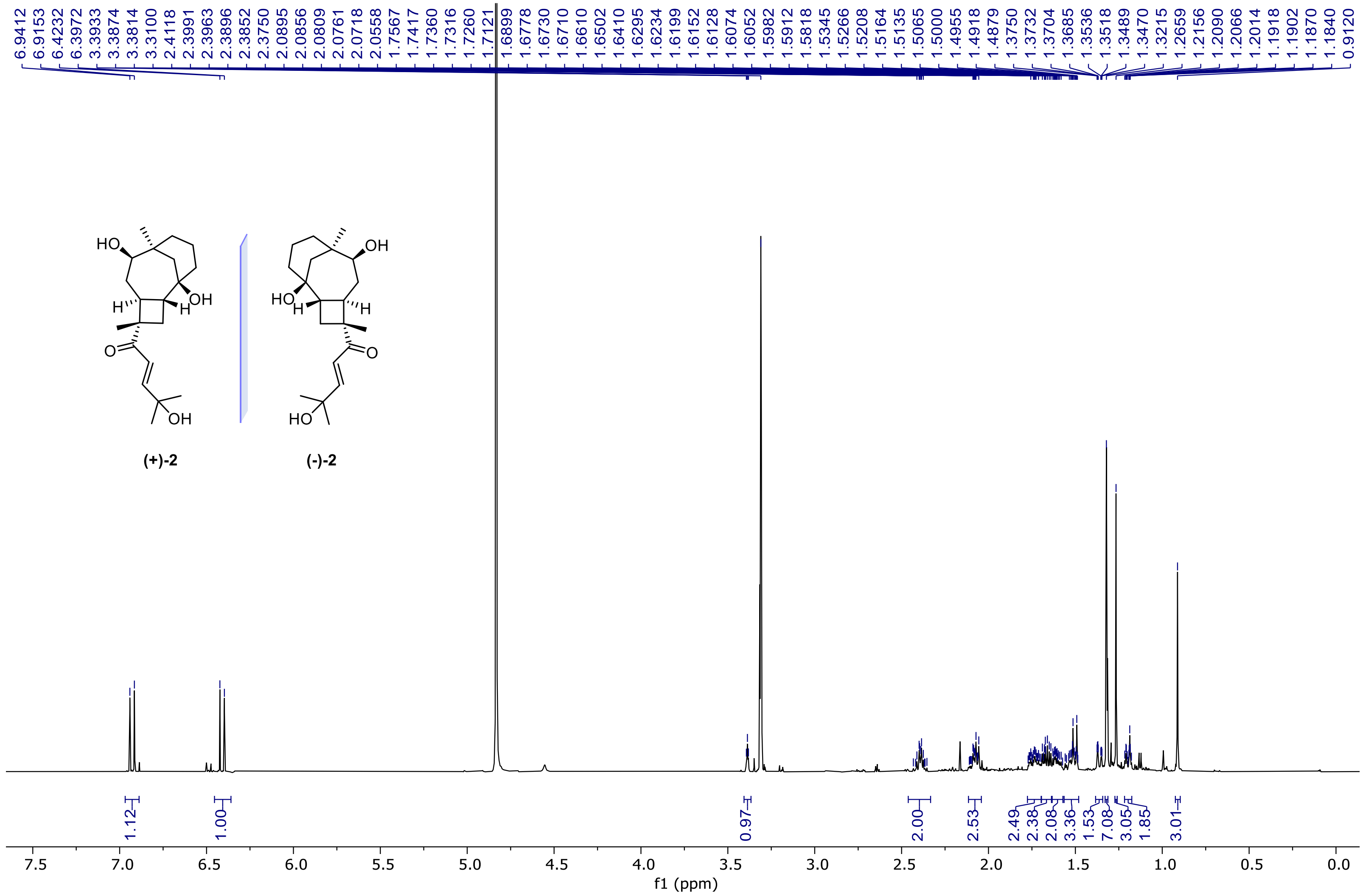

Figure S13. Expanded ^{1}H NMR spectrum of sclerofish B (**2**) (600 MHz, CD_3OD).

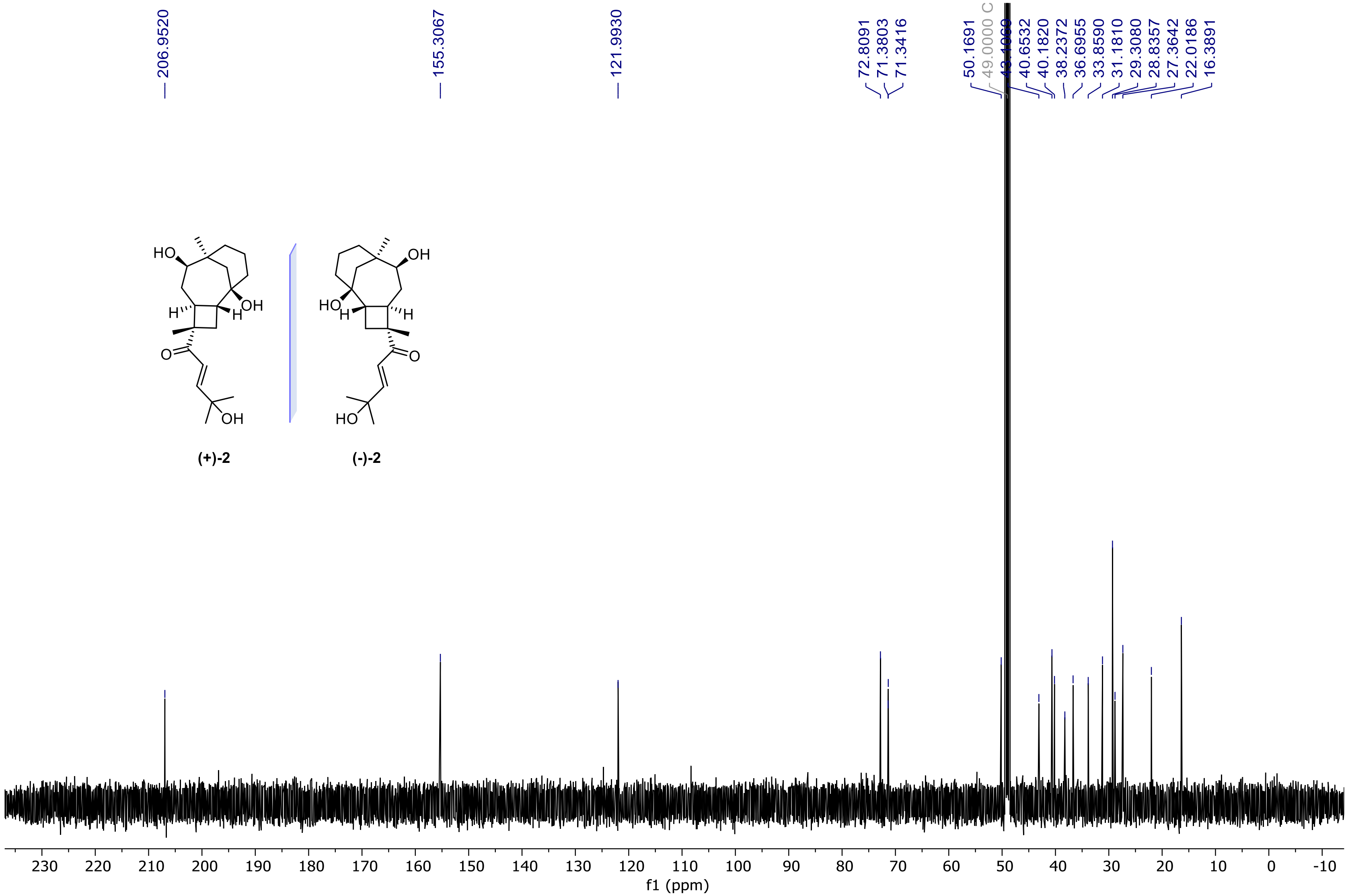

Figure S14. ^{13}C NMR spectrum of sclerofish B (**2**) (150 MHz, CD_3OD).

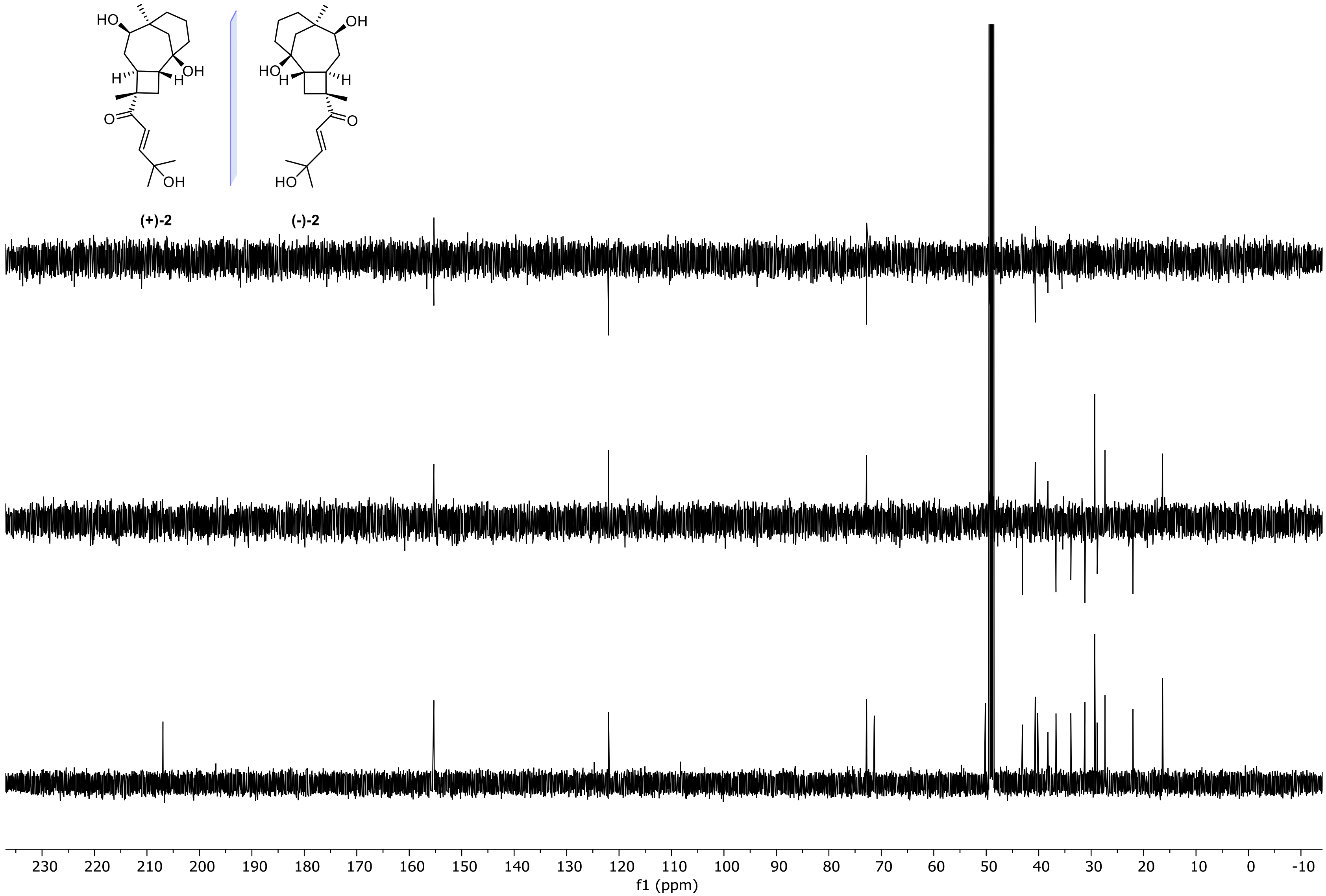

Figure S15. DEPT spectrum of sclerofish B (**2**) (150 MHz, CD_3OD).

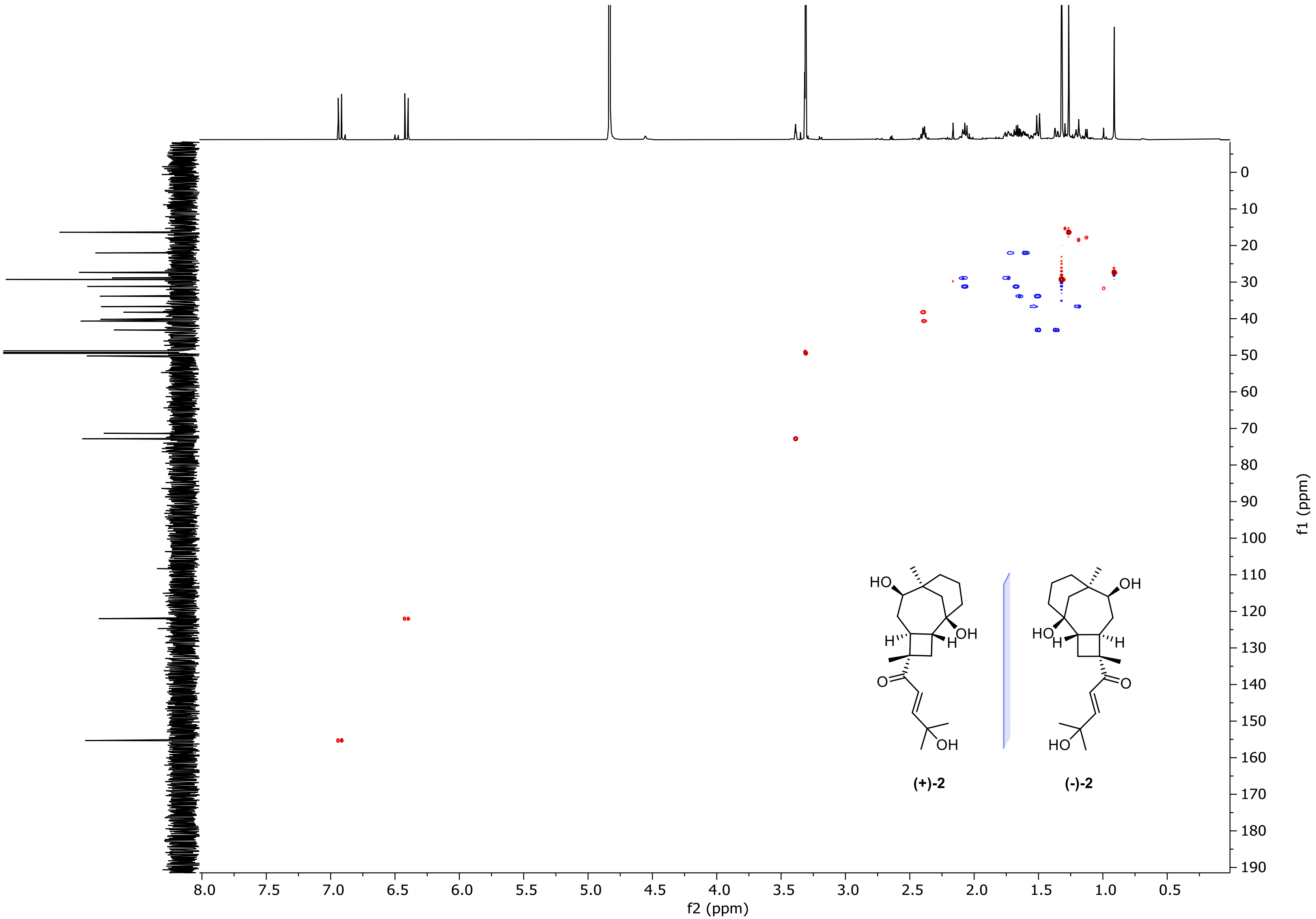

Figure S16. HSQC spectrum of sclerofish B (**2**) (600 and 150 MHz, CD_3OD).

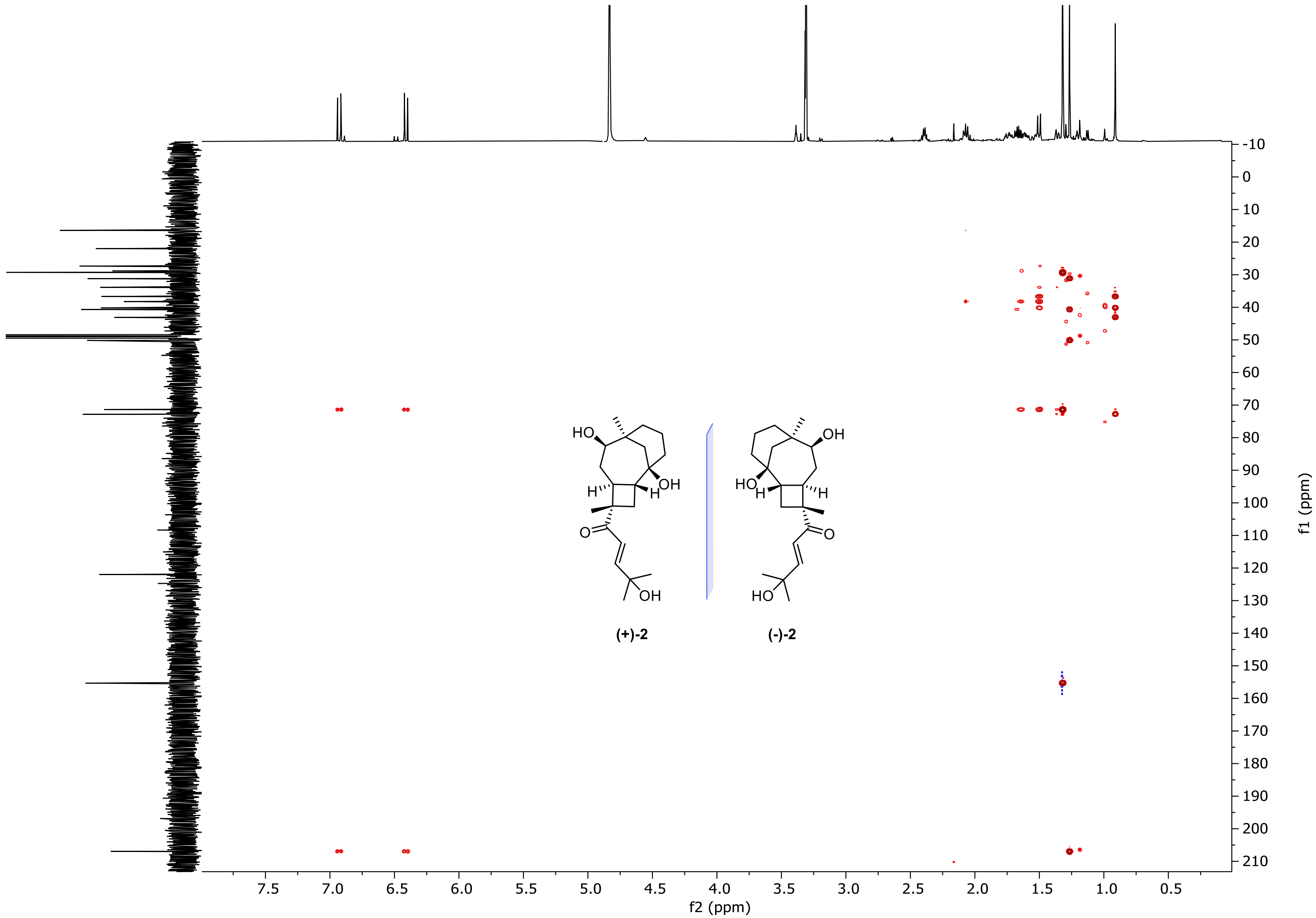

Figure S17. HMBC spectrum of sclerofish B (**2**) (600 and 150 MHz, CD_3OD).

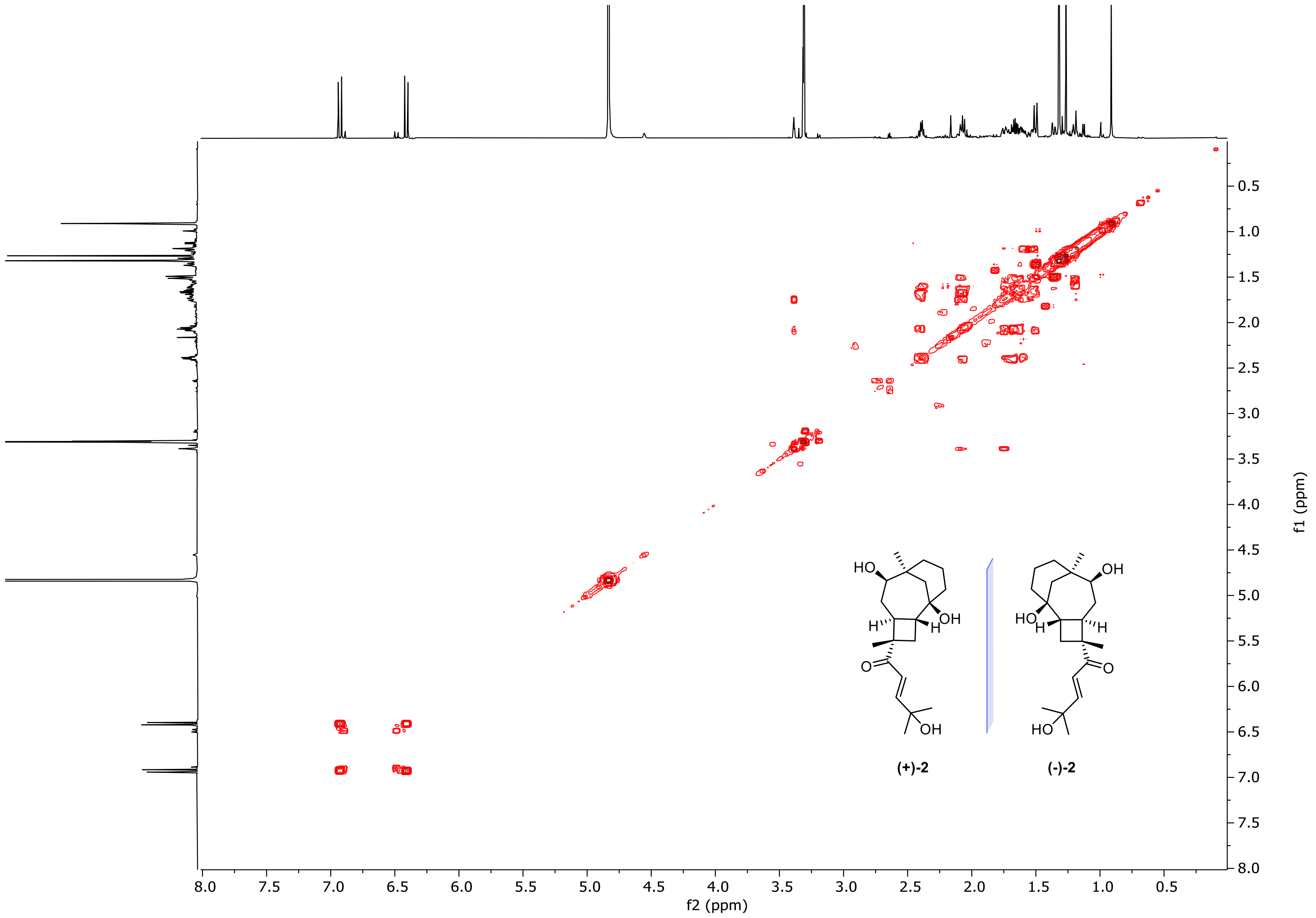

Figure S18. COSY spectrum of sclerofish B (**2**) (600 MHz, CD_3OD).

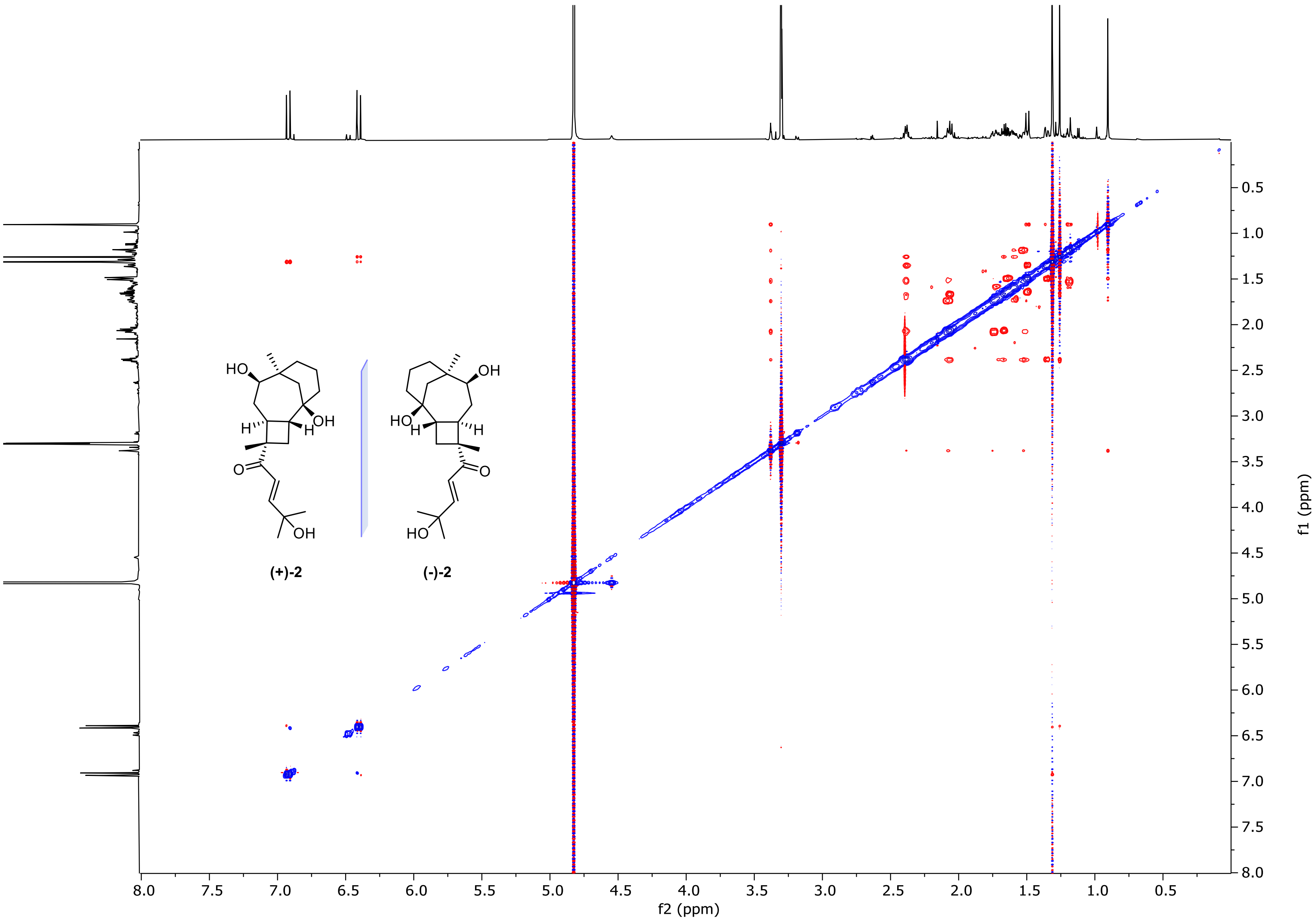

Figure S19. NOESY spectrum of sclerofish B (**2**) (600 MHz, CD_3OD).

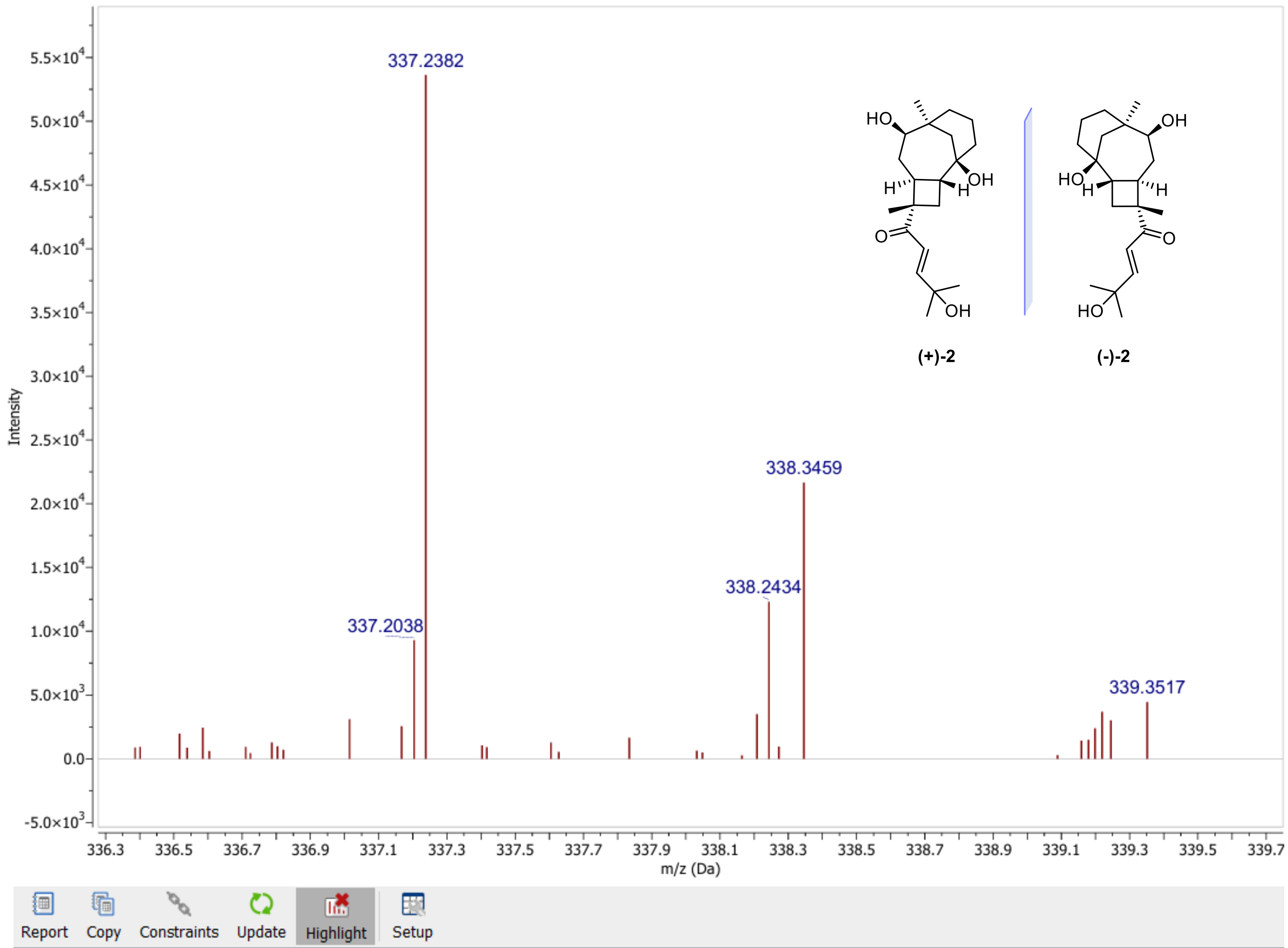

	Formula	Calculated Mass	Target Mass	Double Bond Equivalence	Absolute Error (ppm)	Error (mDa)	Error (ppm)	Fitness
1	$C_{20}H_{33}O_4$	337.23734	337.23819	4.5	2.53	0.85	2.53	0.804

Figure S20. HRESIMS spectrum of sclerofish B (**2**).

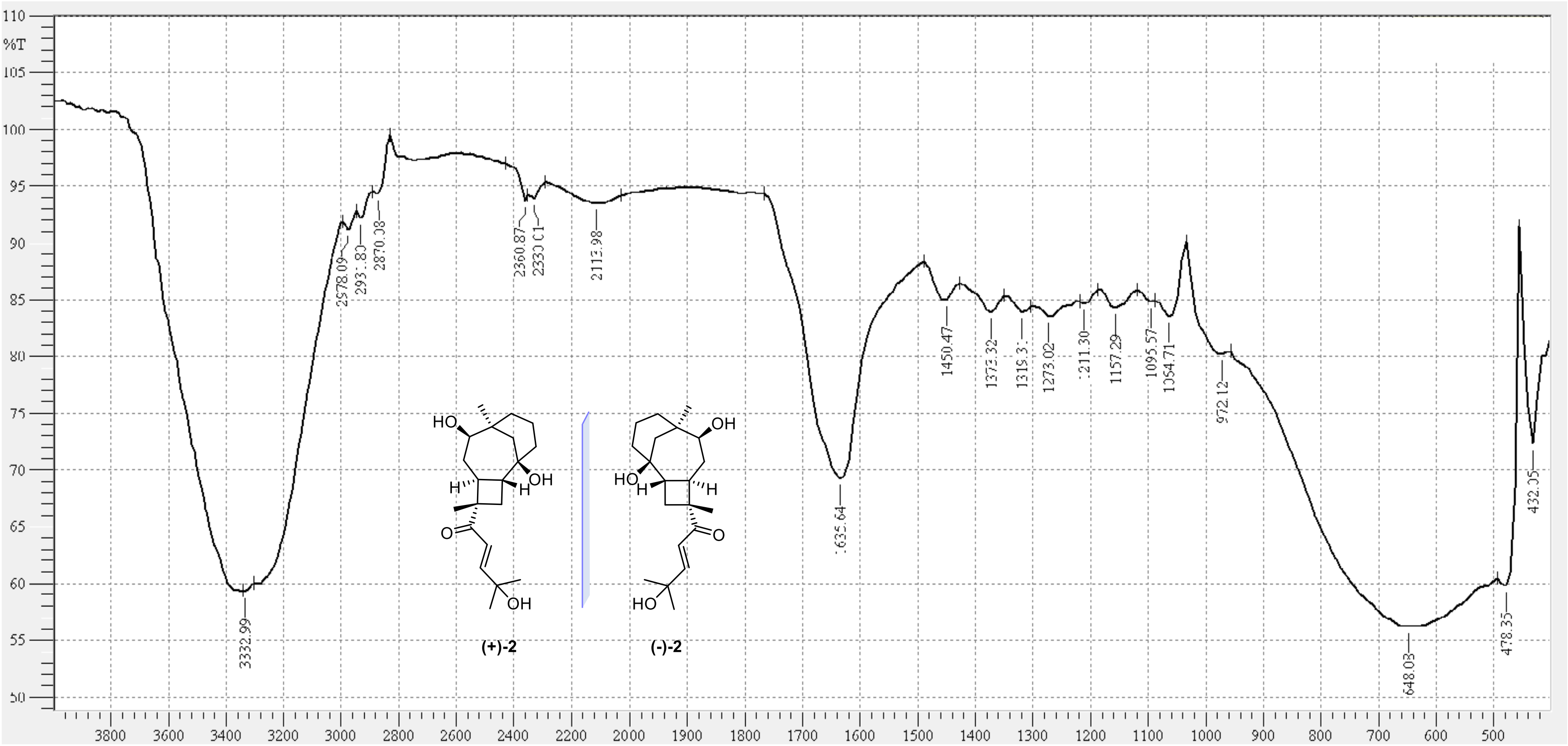

Figure S21. Infrared (IR) spectrum sclerofish B (**2**).

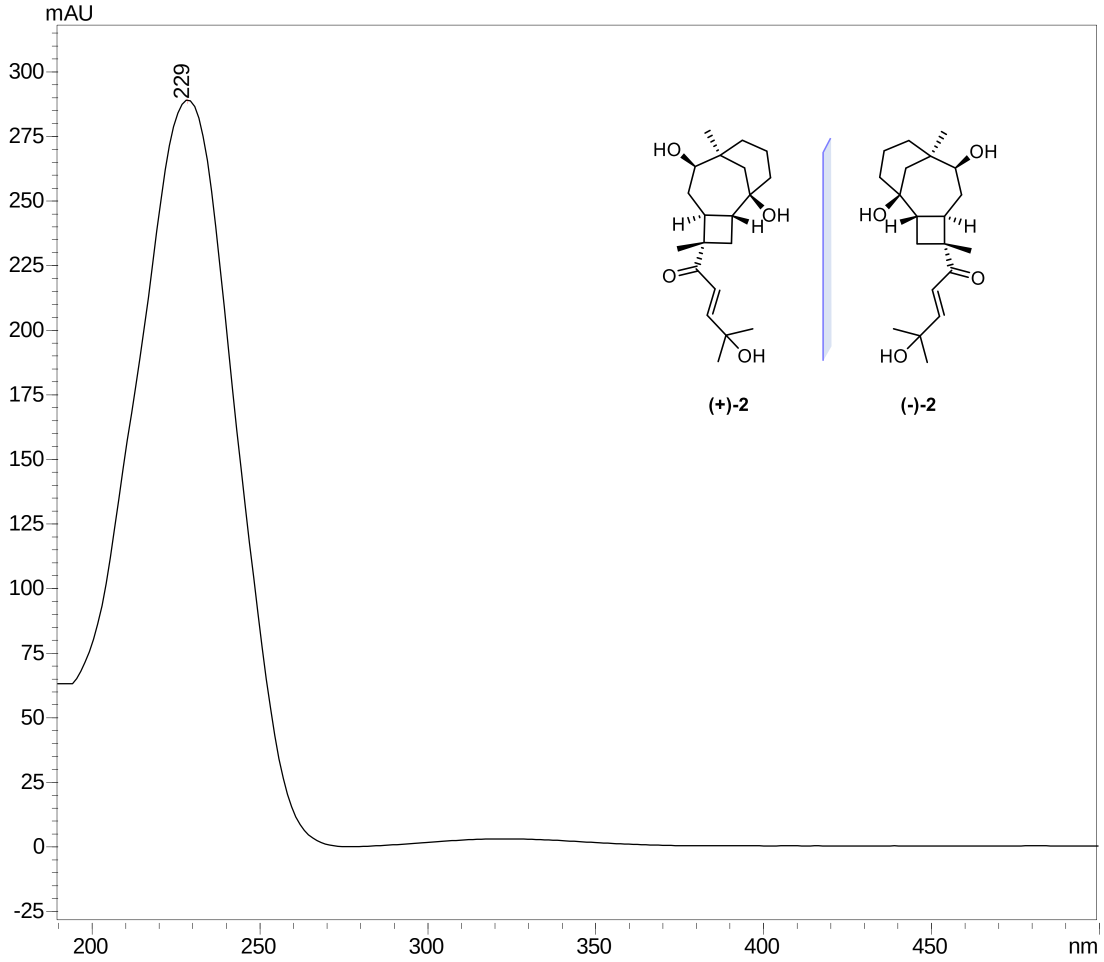

Figure S22. Ultraviolet (UV) spectrum sclerofish B (**2**).

Table S1. 2D NMR data for compound **1**

No.	**1**[a]		
	^{1}H-^{1}H COSY	^{1}H-^{13}C HMBC	NOESY
1	H-9, H_2-2	C-8, C-9, C-11, C-12, C-18	H-5
2	H-1, H_2-3	C-1, C-9	-
3α	H_2-2	C-5	H-5
3β			-
4	-	-	-
5	H_2-6	-	H-1, H_3-20
6	H-5, H_2-7	-	-
7	H_2-6	C-5, C-8, C-19	-
8	-	-	-
9	H-1, H_2-10	C-1, C-7, C-8, C-19	H_3-18, H-19β
10	H-9	C-1, C-8, C-9, C-18	-
11	-	-	-
12	-	-	-
13	H-14	C-12, C-14, C-15	-
14	H-13	C-12, C-13, C-15, C-16, C-17	-
15	-	-	-
16	-	C-14, C-15, C-17	-
17	-	C-14, C-15, C-16	-
18	-	-	-
19α	-	C-4, C-5, C-8	-
19β			H-9
20	-	C-3, C-4, C-5, C-19	-

[a]Spectra recorded in CD_3OD at 600 MHz (^{1}H NMR) and 150 MHz (^{13}C NMR).

Table S2. 2D NMR data for compound **2**

No.	**2**[a]		
	^{1}H-^{1}H COSY	^{1}H-^{13}C HMBC	NOESY
1	H-9, H_2-2	C-8, C-9, C-11, C-12, C-18	H-3
2	H-1, H-3	-	-
3	H_2-2	-	H-1, H-5α, H_3-20
4	-	-	-
5α	H_2-6	-	H-3
5β			-
6	H_2-5, H_2-7	-	-
7	H_2-6	-	-
8	-	-	-
9	H-1, H_2-10	C-1, C-8, C-10	H_3-18, H-19β
10	H-9	C-1, C-18	-
11	-	-	-
12	-	-	-
13	H-14	C-12, C-14, C-15	-
14	H-13	C-12, C-13, C-15, C-16, C-17	-
15	-	-	-
16	-	C-14, C-15, C-17	-
17	-	C-14, C-15, C-16	-
18	-	-	-
19α	-	C-3, C-4, C-7, C-8	-
19β			H-9
20	-	C-3, C-4, C-5, C-19	-

[a]Spectra recorded in CD_3OD at 600 MHz (^{1}H NMR) and 150 MHz (^{13}C NMR).

2. Chiral HPLC analysis

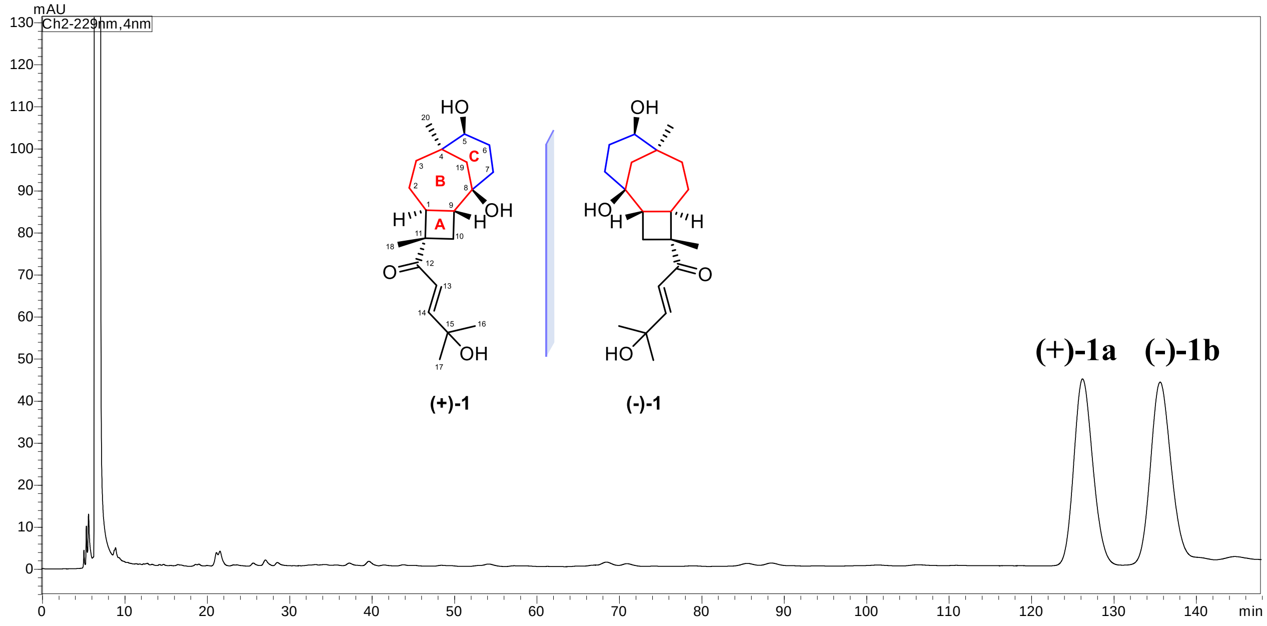

Figure S23. Chiral HPLC analysis chromatography of compound **1**

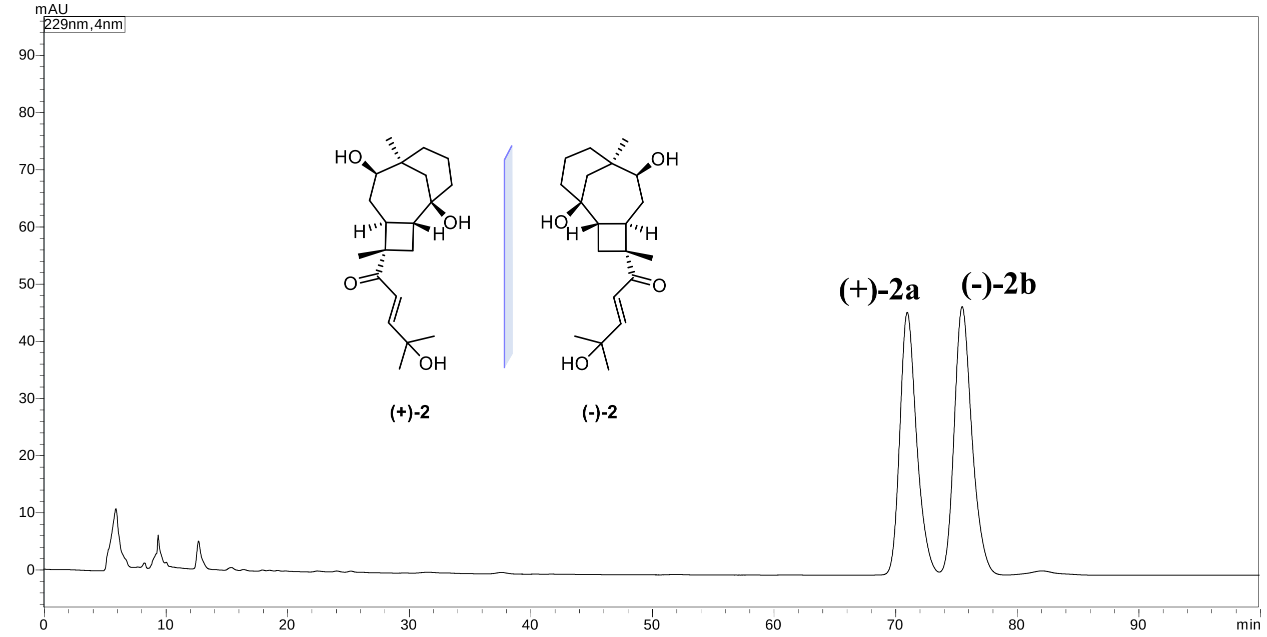

Figure S24. Chiral HPLC analysis chromatography of compound **2**

3. Computational details

3.1. Structures of isomers studied

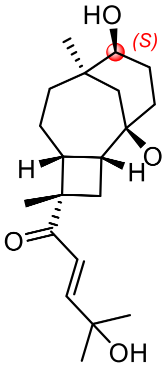

1a'

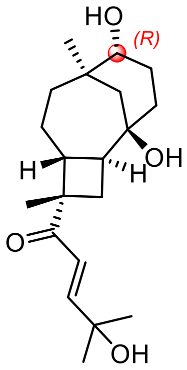

1b'

Figure S25. Structures of isomers **1a´** and **1b´** of compound **1**.

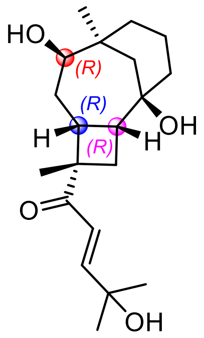

2a'

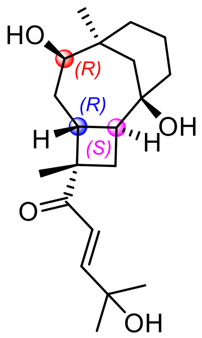

2b'

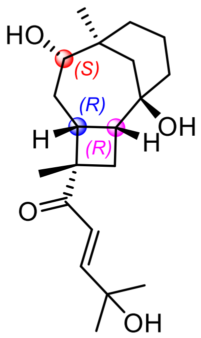

2c'

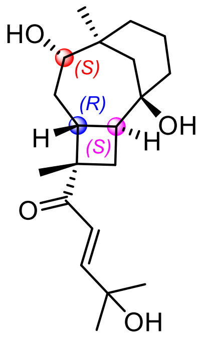

2d'

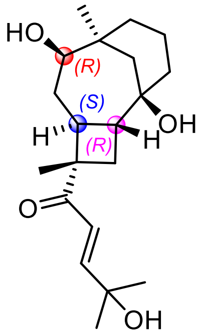

2e'

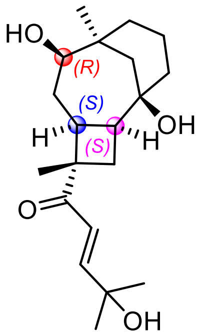

2f'

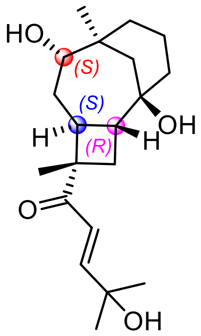

2g'

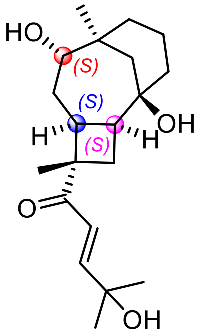

2h'

Figure S26. Structures of isomers **2a´-2h´** of compound **2**.

3.2. Gibbs free energy of the conformers and the Boltzmann distribution

Table S3. Gibbs free energy of the conformers and the Boltzmann distribution for **1**-5*S*.

Conformer	Gibbs free energy (Hartree)	Boltzmann population (%)
**1**-5*S*-conf. 1	-1081.765382	8.47
**1**-5*S*-conf. 2	-1081.765986	16.05
**1**-5*S*-conf. 3	-1081.765562	10.24
**1**-5*S*-conf. 4	-1081.765377	8.42
**1**-5*S*-conf. 5	-1081.762346	0.34
**1**-5*S*-conf. 6	-1081.76276	0.53
**1**-5*S*-conf. 7	-1081.766487	27.29
**1**-5*S*-conf. 8	-1081.766097	18.05
**1**-5*S*-conf. 9	-1081.763169	0.81
**1**-5*S*-conf. 10	-1081.762296	0.32
**1**-5*S*-conf. 11	-1081.762386	0.35
**1**-5*S*-conf. 12	-1081.763515	1.17
**1**-5*S*-conf. 13	-1081.76328	0.91
**1**-5*S*-conf. 14	-1081.762867	0.59
**1**-5*S*-conf. 15	-1081.762647	0.47
**1**-5*S*-conf. 16	-1081.762335	0.34
**1**-5*S*-conf. 17	-1081.763355	0.99
**1**-5*S*-conf. 18	-1081.763442	1.08
**1**-5*S*-conf. 18	-1081.763981	1.92
**1**-5*S*-conf. 20	-1081.763836	1.65

Table S4. Gibbs free energy of the conformers and the Boltzmann distribution for **1**-5*R*.

**Conformer**	**Gibbs free energy (Hartree)**	**Boltzmann population (%)**
**1**-5*R*-conf. 1	-1081.760442	5.69
**1**-5*R*-conf. 2	-1081.759779	2.82
**1**-5*R*-conf. 3	-1081.761178	12.41
**1**-5*R*-conf. 4	-1081.761031	10.62
**1**-5*R*-conf. 5	-1081.760818	8.48
**1**-5*R*-conf. 6	-1081.760367	5.26
**1**-5*R*-conf. 7	-1081.757898	0.38
**1**-5*R*-conf. 8	-1081.760359	5.21
**1**-5*R*-conf. 9	-1081.761326	14.52
**1**-5*R*-conf. 10	-1081.75728	0.20
**1**-5*R*-conf. 11	-1081.761636	20.16
**1**-5*R*-conf. 12	-1081.76059	6.66
**1**-5*R*-conf. 13	-1081.758634	0.84
**1**-5*R*-conf. 14	-1081.757231	0.19
**1**-5*R*-conf. 15	-1081.76053	6.25
**1**-5*R*-conf. 16	-1081.757715	0.32

Table S5. Gibbs free energy of the conformers and the Boltzmann distribution for **2**-1*R*3*R*9*R*.

**Conformer**	**Gibbs free energy (Hartree)**	**Boltzmann population (%)**
**2-**1*R*3*R*9*R*-conf. 1	-1081.796997	25.33
**2-**1*R*3*R*9*R*-conf. 2	-1081.797101	28.28
**2-**1*R*3*R*9*R*-conf. 3	-1081.7971	28.25
**2-**1*R*3*R*9*R*-conf. 4	-1081.793625	0.71
**2-**1*R*3*R*9*R*-conf. 5	-1081.793404	0.56
**2-**1*R*3*R*9*R*-conf. 6	-1081.794051	1.12
**2-**1*R*3*R*9*R*-conf. 7	-1081.794149	1.24
**2-**1*R*3*R*9*R*-conf. 8	-1081.794945	2.88
**2-**1*R*3*R*9*R*-conf. 9	-1081.794861	2.64
**2-**1*R*3*R*9*R*-conf. 10	-1081.79423	1.35
**2-**1*R*3*R*9*R*-conf. 11	-1081.79241	0.20
**2-**1*R*3*R*9*R*-conf. 12	-1081.793935	0.99
**2-**1*R*3*R*9*R*-conf. 13	-1081.794526	1.85
**2-**1*R*3*R*9*R*-conf. 14	-1081.793993	1.05
**2-**1*R*3*R*9*R*-conf. 15	-1081.795138	3.54

Table S6. Gibbs free energy of the conformers and the Boltzmann distribution for **2**-1*R*3*R*9*S*.

Conformer	Gibbs free energy (Hartree)	Boltzmann population (%)
**2**-1*R*3*R*9*S*-conf. 1	-1081.781302	11.04
**2**-1*R*3*R*9*S*-conf. 2	-1081.781328	11.35
**2**-1*R*3*R*9*S*-conf. 3	-1081.779142	1.12
**2**-1*R*3*R*9*S*-conf. 4	-1081.782008	23.33
**2**-1*R*3*R*9*S*-conf. 5	-1081.782005	23.25
**2**-1*R*3*R*9*S*-conf. 6	-1081.778041	0.35
**2**-1*R*3*R*9*S*-conf. 7	-1081.778041	0.35
**2**-1*R*3*R*9*S*-conf. 8	-1081.778857	0.83
**2**-1*R*3*R*9*S*-conf. 9	-1081.781421	12.53
**2**-1*R*3*R*9*S*-conf. 10	-1081.778967	0.93
**2**-1*R*3*R*9*S*-conf. 11	-1081.780226	3.53
**2**-1*R*3*R*9*S*-conf. 12	-1081.779233	1.23
**2**-1*R*3*R*9*S*-conf. 13	-1081.780457	4.51
**2**-1*R*3*R*9*S*-conf. 14	-1081.777866	0.29
**2**-1*R*3*R*9*S*-conf. 15	-1081.779354	1.40
**2**-1*R*3*R*9*S*-conf. 16	-1081.778852	0.82
**2**-1*R*3*R*9*S*-conf. 17	-1081.779204	1.20
**2**-1*R*3*R*9*S*-conf. 18	-1081.778388	0.50
**2**-1*R*3*R*9*S*-conf. 19	-1081.779359	1.41

Table S7. Gibbs free energy of the conformers and the Boltzmann distribution for **2**-1*R*3*S*9*R*.

Conformer	Gibbs free energy (Hartree)	Boltzmann population (%)
**2**-1*R*3*S*9*R*-conf. 1	-1081.793191	14.31
**2**-1*R*3*S*9*R*-conf. 2	-1081.792658	8.14
**2**-1*R*3*S*9*R*-conf. 3	-1081.79259	7.57
**2**-1*R*3*S*9*R*-conf. 4	-1081.789765	0.38
**2**-1*R*3*S*9*R*-conf. 5	-1081.793087	12.82
**2**-1*R*3*S*9*R*-conf. 6	-1081.790708	1.03
**2**-1*R*3*S*9*R*-conf. 7	-1081.79137	2.08
**2**-1*R*3*S*9*R*-conf. 8	-1081.793713	24.87
**2**-1*R*3*S*9*R*-conf. 9	-1081.790126	0.56
**2**-1*R*3*S*9*R*-conf. 10	-1081.790064	0.52
**2**-1*R*3*S*9*R*-conf. 11	-1081.793215	14.68
**2**-1*R*3*S*9*R*-conf. 12	-1081.793103	13.04

Table S8. Gibbs free energy of the conformers and the Boltzmann distribution for **2**-1*R*3*S*9*S*.

Conformer	Gibbs free energy (Hartree)	Boltzmann population (%)
**2-**1*R*3*S*9*S*-conf. 1	-1081.777171	5.62
**2-**1*R*3*S*9*S*-conf. 2	-1081.778329	19.16
**2-**1*R*3*S*9*S*-conf. 3	-1081.774656	0.39
**2-**1*R*3*S*9*S*-conf. 4	-1081.77693	4.36
**2-**1*R*3*S*9*S*-conf. 5	-1081.778348	19.55
**2-**1*R*3*S*9*S*-conf. 6	-1081.774781	0.45
**2-**1*R*3*S*9*S*-conf. 7	-1081.777945	12.76
**2-**1*R*3*S*9*S*-conf. 8	-1081.776299	2.23
**2-**1*R*3*S*9*S*-conf. 9	-1081.776122	1.85
**2-**1*R*3*S*9*S*-conf. 10	-1081.776165	1.94
**2-**1*R*3*S*9*S*-conf. 11	-1081.778004	13.58
**2-**1*R*3*S*9*S*-conf. 12	-1081.775076	0.61
**2-**1*R*3*S*9*S*-conf. 13	-1081.778226	17.18
**2-**1*R*3*S*9*S*-conf. 14	-1081.774431	0.31

Table S9. Gibbs free energy of the conformers and the Boltzmann distribution for **2**-1*S*3*R*9*R*.

**Conformer**	**Gibbs free energy (Hartree)**	**Boltzmann population (%)**
**2**-1*S*3*R*9*R*-conf. 1	-1081.762815	3.92
**2**-1*S*3*R*9*R*-conf. 2	-1081.763742	10.47
**2**-1*S*3*R*9*R*-conf. 3	-1081.76392	12.64
**2**-1*S*3*R*9*R*-conf. 4	-1081.760144	0.23
**2**-1*S*3*R*9*R*-conf. 5	-1081.763264	6.31
**2**-1*S*3*R*9*R*-conf. 6	-1081.760423	0.31
**2**-1*S*3*R*9*R*-conf. 7	-1081.763925	12.71
**2**-1*S*3*R*9*R*-conf. 8	-1081.765126	45.34
**2**-1*S*3*R*9*R*-conf. 9	-1081.760473	0.33
**2**-1*S*3*R*9*R*-conf. 10	-1081.760111	0.22
**2**-1*S*3*R*9*R*-conf. 11	-1081.76124	0.74
**2**-1*S*3*R*9*R*-conf. 12	-1081.760277	0.27
**2**-1*S*3*R*9*R*-conf. 13	-1081.760643	0.39
**2**-1*S*3*R*9*R*-conf. 14	-1081.76054	0.35
**2**-1*S*3*R*9*R*-conf. 15	-1081.760423	0.31
**2**-1*S*3*R*9*R*-conf. 16	-1081.761194	0.70
**2**-1*S*3*R*9*R*-conf. 17	-1081.761216	0.72
**2**-1*S*3*R*9*R*-conf. 18	-1081.762291	2.25
**2**-1*S*3*R*9*R*-conf. 19	-1081.762072	1.79

Table S10. Gibbs free energy of the conformers and the Boltzmann distribution for **2**-1*S*3*R*9*S*.

Conformer	Gibbs free energy (Hartree)	Boltzmann population (%)
**2-**1*S*3*R*9*S*-conf. 1	-1081.798042	4.69
**2-**1*S*3*R*9*S*-conf. 2	-1081.798039	4.68
**2-**1*S*3*R*9*S*-conf. 3	-1081.799231	16.52
**2-**1*S*3*R*9*S*-conf. 4	-1081.798996	12.88
**2-**1*S*3*R*9*S*-conf. 5	-1081.795739	0.41
**2-**1*S*3*R*9*S*-conf. 6	-1081.798211	5.61
**2-**1*S*3*R*9*S*-conf. 7	-1081.799292	17.63
**2-**1*S*3*R*9*S*-conf. 8	-1081.795945	0.51
**2-**1*S*3*R*9*S*-conf. 9	-1081.79573	0.41
**2-**1*S*3*R*9*S*-conf. 10	-1081.798995	12.87
**2-**1*S*3*R*9*S*-conf. 11	-1081.798211	5.61
**2-**1*S*3*R*9*S*-conf. 12	-1081.799134	14.91
**2-**1*S*3*R*9*S*-conf. 13	-1081.795477	0.31
**2-**1*S*3*R*9*S*-conf. 14	-1081.795941	0.51
**2-**1*S*3*R*9*S*-conf. 15	-1081.796083	0.59
**2-**1*S*3*R*9*S*-conf. 16	-1081.797176	1.87

Table S11. Gibbs free energy of the conformers and the Boltzmann distribution for **2**-1*S*3*S*9*R*.

**Conformer**	**Gibbs free energy (Hartree)**	**Boltzmann population (%)**
**2-**1*S*3*S*9*R*-conf. 1	-1081.757831	13.84
**2-**1*S*3*S*9*R*-conf. 2	-1081.756657	3.99
**2-**1*S*3*S*9*R*-conf. 3	-1081.756989	5.67
**2-**1*S*3*S*9*R*-conf. 4	-1081.757596	10.79
**2-**1*S*3*S*9*R*-conf. 5	-1081.757355	8.36
**2-**1*S*3*S*9*R*-conf. 6	-1081.758246	21.48
**2-**1*S*3*S*9*R*-conf. 7	-1081.754704	0.50
**2-**1*S*3*S*9*R*-conf. 8	-1081.756583	3.69
**2-**1*S*3*S*9*R*-conf. 9	-1081.754322	0.34
**2-**1*S*3*S*9*R*-conf. 10	-1081.756761	4.46
**2-**1*S*3*S*9*R*-conf. 11	-1081.755397	1.05
**2-**1*S*3*S*9*R*-conf. 12	-1081.75763	11.19
**2-**1*S*3*S*9*R*-conf. 13	-1081.75543	1.09
**2-**1*S*3*S*9*R*-conf. 14	-1081.757767	12.93
**2-**1*S*3*S*9*R*-conf. 15	-1081.75289	0.07
**2-**1*S*3*S*9*R*-conf. 16	-1081.754361	0.35
**2-**1*S*3*S*9*R*-conf. 17	-1081.753743	0.18
**2-**1*S*3*S*9*R*-conf. 18	-1081.751447	0.02

Table S12. Gibbs free energy of the conformers and the Boltzmann distribution for **2**-1*S*3*S*9*S*.

**Conformer**	**Gibbs free energy (Hartree)**	**Boltzmann population (%)**
**2**-1*S*3*S*9*S*-conf. 1	-1081.788813	0.01
**2**-1*S*3*S*9*S*-conf. 2	-1081.79536	5.28
**2**-1*S*3*S*9*S*-conf. 3	-1081.790017	0.02
**2**-1*S*3*S*9*S*-conf. 4	-1081.789393	0.01
**2**-1*S*3*S*9*S*-conf. 5	-1081.79624	13.40
**2**-1*S*3*S*9*S*-conf. 6	-1081.786276	0.00
**2**-1*S*3*S*9*S*-conf. 7	-1081.789467	0.01
**2**-1*S*3*S*9*S*-conf. 8	-1081.793131	0.50
**2**-1*S*3*S*9*S*-conf. 9	-1081.786316	0.00
**2**-1*S*3*S*9*S*-conf. 10	-1081.796396	15.80
**2**-1*S*3*S*9*S*-conf. 11	-1081.796628	20.21
**2**-1*S*3*S*9*S*-conf. 12	-1081.790678	0.04
**2**-1*S*3*S*9*S*-conf. 13	-1081.785392	0.00
**2**-1*S*3*S*9*S*-conf. 14	-1081.785365	0.00
**2**-1*S*3*S*9*S*-conf. 15	-1081.797366	44.15
**2**-1*S*3*S*9*S*-conf. 16	-1081.793295	0.59

3.3. The cartesian coordinates of the dominant conformers of compounds 1 and 2

Table S13. The cartesian coordinates of the dominant conformers for compound **1**

**1**-5*S*-conf. 1	**Coordinates (Å)**				**Coordinates (Å)**		
	**X**	**Y**	**Z**		**X**	**Y**	**Z**
O	2.383622	1.98318	-1.62652	H	-2.01179	-2.11687	1.551046
H	-1.48511	2.211207	0.99776	H	-0.90528	-2.49641	0.260388
H	-0.08373	0.161545	-0.75607	H	-1.62151	-2.79978	-1.85834
C	-0.25388	0.608909	0.226391	H	-2.80111	-3.64116	-0.83905
C	-0.504	-0.57608	1.176788	H	-3.35355	-2.63634	-2.18782
C	-1.53521	-1.69514	0.661029	H	-3.93073	-0.91372	1.841313
C	-2.60747	-2.71206	-1.38837	H	-4.76919	-2.12694	-0.48926
C	-2.67387	-1.49301	-0.44722	H	-5.40339	-0.18408	-1.41312
O	-4.43292	-1.61713	1.397485	H	-5.75494	0.155299	0.263903
C	-4.21196	-1.33464	0.022599	H	-4.19641	1.778922	-1.59219
C	-4.9334	0.017646	-0.44483	H	-4.50111	2.140825	0.109833
C	-4.11198	1.378948	-0.57608	H	-3.14033	-0.14499	-2.04794
C	-2.43233	-0.21283	-1.21675	H	-1.45119	-0.18479	-1.68416
O	-2.9018	0.489615	1.168055	H	-3.16992	1.284447	1.648777
C	-2.68342	0.963681	-0.23409	H	-0.35326	3.633512	-0.58136
C	-1.34557	1.694641	0.043286	H	-0.1547	2.302282	-1.75316
C	-0.28349	2.548521	-0.69651	H	1.685684	3.425435	1.314395
C	1.131584	2.498828	1.499487	H	1.746128	1.864615	2.145563
C	0.798438	1.798853	0.171966	H	0.227251	2.757583	2.056442
C	2.088209	1.448597	-0.55976	H	2.6319	-0.03127	1.003128
C	2.956009	0.418939	0.071574	H	4.407858	0.511608	-1.42783
C	4.110681	0.039949	-0.49266	H	5.117372	-2.16734	1.606485
O	4.503834	-1.50308	1.263335	H	7.133577	-1.09026	0.695114
C	6.427251	-0.33887	0.324832	H	6.850807	0.103597	-0.58168
C	5.060596	-0.99614	0.045218	H	6.322049	0.444007	1.080657
C	5.204235	-2.13705	-0.98224	H	5.581036	-1.76689	-1.94032
H	0.442082	-1.10983	1.338093	H	5.909893	-2.8878	-0.60957
H	-0.83026	-0.20815	2.150927	H	4.237285	-2.61919	-1.14872

**1**-5*S*-conf. 2	Coordinates (Å)				Coordinates (Å)		
	**X**	**Y**	**Z**		**X**	**Y**	**Z**
O	-1.89448	1.626898	0.729775	H	2.457367	2.578581	0.965754
H	1.081661	-2.02543	0.817146	H	3.542546	1.488034	1.781738
H	0.351613	0.784276	-0.10516	H	2.735346	3.120269	-1.2085
C	0.530003	0.066551	0.697409	H	4.358235	3.164244	-0.49977
C	1.470841	0.787115	1.678407	H	4.102243	2.407144	-2.07945
C	2.744707	1.52393	1.033866	H	4.759652	-0.37275	1.292216
C	3.663184	2.550519	-1.0853	H	5.498552	0.986624	-0.99086
C	3.403514	1.199351	-0.39018	H	5.00202	-0.74312	-2.32916
O	5.40583	0.17481	0.8162	H	5.539947	-1.60869	-0.91077
C	4.782965	0.359517	-0.44773	H	3.060411	-1.99498	-2.39581
C	4.722631	-0.99547	-1.30083	H	3.550514	-2.86597	-0.93917
C	3.386977	-1.8659	-1.35837	H	2.86786	0.234362	-2.22899
C	2.465683	0.34735	-1.21781	H	1.492071	0.811007	-1.35405
O	3.102879	-1.06586	0.781077	H	3.110898	-1.99921	1.033137
C	2.395425	-1.04654	-0.53676	H	-0.88123	-2.45376	-0.50543
C	0.973022	-1.26412	0.038861	H	-0.71556	-0.90407	-1.37979
C	-0.48996	-1.43437	-0.44935	H	-2.14297	-1.93979	1.980545
C	-1.20357	-1.3864	2.07827	H	-1.28969	-0.73186	2.952024
C	-0.90337	-0.55937	0.817106	H	-0.41195	-2.11206	2.282626
C	-2.0486	0.416864	0.577165	H	-3.40698	-1.23873	-0.00343
C	-3.34699	-0.16633	0.148103	H	-4.32411	1.678686	0.102799
C	-4.42194	0.606348	-0.05857	H	-6.58856	-1.58367	-0.95346
O	-5.71333	-1.28509	-0.67011	H	-7.13773	0.485532	-2.16375
C	-6.14119	0.807634	-1.84178	H	-6.15239	1.897902	-1.75138
C	-5.78091	0.135398	-0.50162	H	-5.41789	0.527064	-2.61204
C	-6.82075	0.488007	0.581055	H	-6.85536	1.565784	0.765612
H	0.900635	1.576253	2.185142	H	-7.81793	0.169025	0.257847
H	1.811416	0.093162	2.449252	H	-6.57794	-0.02051	1.517871

**1**-5*S*-conf. 3	Coordinates (Å)				Coordinates (Å)		
	**X**	**Y**	**Z**		**X**	**Y**	**Z**
O	2.370868	1.961042	-1.64933	H	-0.91308	-2.50007	0.245543
H	-1.47614	2.206646	1.003574	H	-2.01293	-2.12199	1.542304
H	-0.0892	0.159664	-0.76455	H	-1.63914	-2.79254	-1.87175
C	-0.25323	0.603615	0.220463	H	-2.81656	-3.63484	-0.85064
C	-0.50231	-0.5842	1.167385	H	-3.37211	-2.62305	-2.19287
C	-1.5389	-1.6984	0.651841	H	-3.92719	-0.91529	1.845945
C	-2.62276	-2.70405	-1.39702	H	-4.77892	-2.11647	-0.48612
C	-2.68168	-1.48862	-0.45072	H	-5.41216	-0.16808	-1.39877
O	-4.43291	-1.61559	1.40123	H	-5.75476	0.165449	0.28125
C	-4.21726	-1.32788	0.026607	H	-4.20022	1.792117	-1.57567
C	-4.937	0.028357	-0.43193	H	-4.49617	2.148122	0.129118
C	-4.11236	1.387872	-0.56156	H	-3.15189	-0.13301	-2.0438
C	-2.44018	-0.20604	-1.21625	H	-1.46123	-0.17875	-1.68832
O	-2.89672	0.488104	1.173497	H	-3.16181	1.281466	1.658345
C	-2.68349	0.967211	-0.22772	H	-0.34677	3.631339	-0.57561
C	-1.34238	1.69336	0.046545	H	-0.15795	2.303702	-1.75318
C	-0.28109	2.546589	-0.69513	H	1.70166	3.409784	1.309685
C	1.143944	2.485208	1.493913	H	1.757435	1.846617	2.136621
C	0.802696	1.790794	0.165617	H	0.242636	2.746613	2.054453
C	2.086283	1.436857	-0.57488	H	2.645282	-0.03045	0.993743
C	2.963311	0.413762	0.055879	H	4.418767	0.514239	-1.43883
C	4.121893	0.040092	-0.50416	H	3.777694	-2.15301	0.951934
O	4.543193	-1.62601	1.223745	H	7.072242	-1.05058	0.882656
C	6.367118	-0.30197	0.509501	H	6.835177	0.237466	-0.31806
C	5.073524	-0.9949	0.052157	H	6.150957	0.40829	1.312029
C	5.37435	-2.05318	-1.02617	H	5.819755	-1.59886	-1.91614
H	0.443225	-1.12101	1.322476	H	6.071483	-2.79503	-0.62629
H	-0.82295	-0.21927	2.144481	H	4.456498	-2.56652	-1.33298

**1**-5*S*-conf. 4	Coordinates (Å)				Coordinates (Å)		
	**X**	**Y**	**Z**		**X**	**Y**	**Z**
O	2.390868	1.999071	-1.62347	H	-0.90203	-2.49525	0.260988
H	-1.4817	2.21183	0.995923	H	-2.00787	-2.11484	1.551976
H	-0.08068	0.161954	-0.75822	H	-1.61946	-2.80069	-1.8569
C	-0.25046	0.609822	0.224069	H	-2.79862	-3.64105	-0.83629
C	-0.50059	-0.57423	1.175711	H	-3.35164	-2.63747	-2.18573
C	-1.53192	-1.6938	0.661306	H	-3.927	-0.91148	1.842165
C	-2.60519	-2.71248	-1.38657	H	-4.76648	-2.12643	-0.48714
C	-2.67114	-1.4926	-0.44647	H	-5.40092	-0.18433	-1.41234
O	-4.42938	-1.61518	1.399049	H	-5.75183	0.156483	0.26451
C	-4.209	-1.33376	0.023863	H	-4.19377	1.778391	-1.59349
C	-4.93054	0.018156	-0.44439	H	-4.49795	2.141815	0.108294
C	-4.1091	1.379306	-0.57707	H	-3.13809	-0.14567	-2.04805
C	-2.42981	-0.21301	-1.21706	H	-1.44885	-0.18545	-1.68488
O	-2.89835	0.491071	1.167413	H	-3.16736	1.285965	1.647547
C	-2.6805	0.964146	-0.23507	H	-0.35132	3.634663	-0.58342
C	-1.34254	1.695358	0.041361	H	-0.15288	2.303752	-1.75553
C	-0.28125	2.549741	-0.69876	H	1.68843	3.426518	1.312373
C	1.134932	2.499495	1.497044	H	1.750083	1.864894	2.14228
C	0.801205	1.800195	0.169168	H	0.230791	2.757152	2.054757
C	2.091762	1.454722	-0.5631	H	2.627984	-0.04076	0.985899
C	2.959463	0.419558	0.060559	H	4.41204	0.512689	-1.43704
C	4.114421	0.039066	-0.50235	H	4.704842	-0.76931	1.95657
O	4.622458	-1.4955	1.321425	H	7.157675	-1.1788	0.551684
C	6.475167	-0.41728	0.163683	H	6.846271	-0.07761	-0.80779
C	5.057223	-1.00791	0.046475	H	6.483706	0.439489	0.846254
C	5.053368	-2.23151	-0.88397	H	5.345825	-1.95137	-1.89938
H	0.445185	-1.10827	1.337869	H	5.759221	-2.9792	-0.51033
H	-0.82679	-0.20507	2.149397	H	4.05517	-2.67616	-0.91786

**1**-5*S*-conf. 7	Coordinates (Å)				Coordinates (Å)		
	**X**	**Y**	**Z**		**X**	**Y**	**Z**
O	-1.88792	1.649096	0.697402	H	2.465401	2.58791	0.941951
H	1.074374	-2.01272	0.838388	H	3.546715	1.500817	1.767611
H	0.352509	0.78985	-0.11189	H	2.743704	3.107857	-1.23749
C	0.528975	0.079727	0.697718	H	4.367149	3.152826	-0.53011
C	1.472311	0.806909	1.671428	H	4.107612	2.381802	-2.10245
C	2.748719	1.532857	1.019737	H	4.757206	-0.36807	1.295396
C	3.669624	2.536055	-1.10947	H	5.499824	0.966981	-1.00078
C	3.405695	1.192449	-0.40145	H	4.99681	-0.77375	-2.32247
O	5.405034	0.172703	0.813912	H	5.532605	-1.62756	-0.89619
C	4.782342	0.347455	-0.45149	H	3.050959	-2.0196	-2.37635
C	4.717095	-1.01539	-1.29166	H	3.53887	-2.87861	-0.9119
C	3.378537	-1.88193	-1.34035	H	2.866136	0.211624	-2.23065
C	2.464694	0.335629	-1.22048	H	1.492555	0.801131	-1.36088
O	3.097999	-1.06047	0.791586	H	3.104091	-1.99148	1.052205
C	2.3902	-1.05152	-0.52603	H	-0.89111	-2.44862	-0.47739
C	0.96737	-1.25897	0.052588	H	-0.72137	-0.909	-1.3686
C	-0.49653	-1.42995	-0.43274	H	-2.14741	-1.90571	2.005268
C	-1.20737	-1.35223	2.09551	H	-1.29151	-0.68786	2.96199
C	-0.90624	-0.54001	0.824925	H	-0.41661	-2.07665	2.307249
C	-2.04804	0.43699	0.572794	H	-3.4196	-1.22569	0.050164
C	-3.35294	-0.148	0.164653	H	-4.31486	1.700402	0.029994
C	-4.42189	0.621949	-0.08035	H	-5.77657	-1.60724	0.403614
O	-5.8466	-1.3067	-0.51425	H	-7.04824	0.223066	-2.26248
C	-6.0506	0.554006	-1.95901	H	-5.99678	1.641019	-2.06135
C	-5.78457	0.124602	-0.50725	H	-5.31209	0.103001	-2.62718
C	-6.8618	0.694087	0.435179	H	-6.86122	1.78808	0.425172
H	0.904943	1.603022	2.170422	H	-7.84793	0.342159	0.119055
H	1.810489	0.119452	2.449129	H	-6.68824	0.36621	1.465883

**1**-5*S*-conf. 8	**Coordinates (Å)**				**Coordinates (Å)**		
	**X**	**Y**	**Z**		**X**	**Y**	**Z**
O	-1.88994	1.648678	0.700827	H	3.544159	1.4983	1.77252
H	1.072774	-2.01375	0.831849	H	2.464959	2.586413	0.945491
H	0.354987	0.790788	-0.11563	H	2.74914	3.109204	-1.23257
C	0.528907	0.07921	0.693338	H	4.37075	3.15297	-0.52095
C	1.47004	0.80456	1.670462	H	4.115135	2.383988	-2.09493
C	2.748061	1.531251	1.022686	H	4.755751	-0.37013	1.301098
C	3.674618	2.537055	-1.10287	H	5.504048	0.967264	-0.99191
C	3.408607	1.192577	-0.39727	H	5.535837	-1.62759	-0.88939
O	5.404772	0.171074	0.82173	H	5.004652	-0.77215	-2.31644
C	4.785151	0.34731	-0.44496	H	3.54188	-2.87787	-0.91255
C	4.721802	-1.01467	-1.28669	H	3.058266	-2.01685	-2.37723
C	3.383058	-1.8806	-1.34016	H	2.873609	0.214263	-2.22922
C	2.46957	0.337075	-1.21993	H	1.497921	0.803049	-1.36229
O	3.097279	-1.06171	0.79206	H	3.102672	-1.9931	1.051362
C	2.392859	-1.05086	-0.52743	H	-0.88891	-2.44604	-0.49126
C	0.968504	-1.25858	0.047028	H	-0.71539	-0.9044	-1.3785
C	-0.49396	-1.42767	-0.44306	H	-2.15409	-1.90664	1.987884
C	-1.21307	-1.35549	2.082731	H	-1.29883	-0.69315	2.950581
C	-0.90695	-0.54044	0.815196	H	-0.42438	-2.08202	2.295015
C	-2.04781	0.437677	0.563398	H	-3.40771	-1.21971	-0.00355
C	-3.34891	-0.14503	0.140377	H	-4.33867	1.693673	0.105071
C	-4.43008	0.620776	-0.06003	H	-5.21637	-1.4027	-1.58074
O	-5.76957	-1.27674	-0.79606	H	-7.24165	0.565902	-2.04665
C	-6.25412	0.918466	-1.73564	H	-6.317	1.989189	-1.52007
C	-5.78903	0.124785	-0.49991	H	-5.55666	0.782971	-2.56945
C	-6.78814	0.285605	0.657101	H	-6.85092	1.328906	0.977361
H	0.90155	1.599964	2.16925	H	-7.7806	-0.04135	0.333012
H	1.806291	0.115866	2.447879	H	-6.47827	-0.32383	1.510214

**1**-5*R*-conf. 1	Coordinates (Å)				Coordinates (Å)		
	**X**	**Y**	**Z**		**X**	**Y**	**Z**
O	-2.43208	-1.8654	-1.73506	H	0.939772	2.410066	0.525579
H	1.36506	-2.35179	0.965058	H	1.980618	1.904829	1.828786
H	0.065573	-0.16341	-0.69674	H	3.488932	2.650427	-1.81664
C	0.196698	-0.6739	0.260711	H	1.732617	2.757553	-1.57953
C	0.449103	0.443372	1.291098	H	2.829724	3.578918	-0.46685
C	1.532896	1.563162	0.888403	H	4.906074	2.947027	0.543385
C	2.696043	2.670423	-1.06542	H	4.169919	1.052247	1.443736
C	2.709466	1.400706	-0.19211	H	5.334768	0.151291	-1.19947
O	5.12815	2.175515	0.005583	H	5.75654	-0.38318	0.407632
C	4.204412	1.12762	0.356295	H	4.16832	-1.83593	-1.50549
C	4.904734	-0.16263	-0.24234	H	4.408848	-2.29809	0.181571
C	4.058207	-1.48955	-0.47217	H	3.163547	0.146081	-1.87368
C	2.440772	0.183284	-1.0533	H	1.47013	0.228547	-1.54037
O	2.802382	-0.67565	1.27566	H	3.169014	-1.46271	1.700506
C	2.626676	-1.06218	-0.14399	H	0.233286	-3.64573	-0.72998
C	1.263042	-1.77847	0.039282	H	0.104063	-2.23988	-1.8215
C	0.197843	-2.5539	-0.77906	H	-1.88825	-1.98601	2.047418
C	-1.27338	-2.59699	1.379484	H	-0.39112	-2.9138	1.941756
C	-0.88609	-1.82772	0.106134	H	-1.84662	-3.49501	1.124561
C	-2.14678	-1.39919	-0.63387	H	-2.68729	0.008066	0.99618
C	-3.00119	-0.38111	0.03406	H	-4.41708	-0.35147	-1.50123
C	-4.13112	0.060322	-0.53477	H	-5.13199	2.176764	1.658726
O	-4.52938	1.513193	1.295438	H	-6.39642	-0.36704	0.952259
C	-6.45826	0.461879	0.242138	H	-7.15209	1.211417	0.63894
C	-5.06611	1.092301	0.036174	H	-6.87029	0.086682	-0.69934
C	-5.14836	2.292565	-0.92853	H	-5.84528	3.040829	-0.53498
H	-0.48671	0.994882	1.454164	H	-4.16432	2.755881	-1.03896
H	0.731234	0.004149	2.248981	H	-5.5049	1.987103	-1.91673

**1**-5*R*-conf. 2	**Coordinates (Å)**				**Coordinates (Å)**		
	**X**	**Y**	**Z**		**X**	**Y**	**Z**
O	2.434144	1.865634	-1.73459	H	-0.92535	-2.40265	0.488479
H	-1.36407	2.353973	0.961209	H	-1.961	-1.93533	1.809535
H	-0.06494	0.165658	-0.70072	H	-3.48975	-2.63868	-1.83452
C	-0.19757	0.674662	0.257339	H	-1.73454	-2.744	-1.59965
C	-0.45179	-0.44505	1.285372	H	-2.83801	-3.57586	-0.49
C	-1.52561	-1.57032	0.87254	H	-5.85382	-2.1372	0.518054
C	-2.69729	-2.66712	-1.08242	H	-4.14356	-1.0404	1.448087
C	-2.7117	-1.40431	-0.19778	H	-5.31803	-0.16811	-1.20686
O	-5.02015	-2.26812	0.046463	H	-5.75836	0.373991	0.396169
C	-4.18816	-1.13567	0.363452	H	-4.17436	1.837047	-1.5058
C	-4.89942	0.153575	-0.24755	H	-4.41549	2.294976	0.182855
C	-4.06216	1.489509	-0.47308	H	-3.1622	-0.14209	-1.87636
C	-2.44285	-0.18177	-1.05343	H	-1.47087	-0.22966	-1.53685
O	-2.80214	0.681468	1.275	H	-3.16156	1.471784	1.699918
C	-2.62862	1.064692	-0.14548	H	-0.23214	3.646807	-0.73312
C	-1.26329	1.780163	0.035641	H	-0.10208	2.240721	-1.82429
C	-0.19701	2.554963	-0.78203	H	1.885563	1.986254	2.047068
C	1.271768	2.597532	1.378429	H	0.389047	2.914803	1.939698
C	0.885695	1.82843	0.104616	H	1.845769	3.495256	1.124161
C	2.147238	1.399523	-0.63378	H	2.685061	-0.00781	0.997069
C	3.00046	0.381277	0.035407	H	4.418709	0.351119	-1.49775
C	4.131143	-0.06048	-0.53168	H	5.127105	-2.178	1.663007
O	4.526946	-1.51201	1.300098	H	6.395804	0.366314	0.956506
C	6.457444	-0.46334	0.247228	H	7.150237	-1.21313	0.645338
C	5.06488	-1.09275	0.040822	H	6.87055	-0.08943	-0.69429
C	5.14674	-2.29387	-0.92285	H	5.842841	-3.04237	-0.52828
H	0.485097	-0.99327	1.453599	H	4.162399	-2.75648	-1.03353
H	-0.74197	-0.00855	2.241928	H	5.50417	-1.98949	-1.91106

**1**-5*R*-conf. 3	Coordinates (Å)				Coordinates (Å)		
	**X**	**Y**	**Z**		**X**	**Y**	**Z**
O	-1.91255	1.58446	0.806449	H	2.467222	2.409822	1.280861
H	0.984416	-2.13665	0.774983	H	3.479047	1.230969	2.069803
H	0.341547	0.740078	0.008834	H	4.246897	2.393399	-1.69847
C	0.478514	-0.03009	0.769897	H	2.801327	3.028123	-0.88593
C	1.398606	0.605662	1.827666	H	4.369064	3.08177	-0.07662
C	2.721455	1.343904	1.285596	H	6.172868	1.527132	0.087891
C	3.736135	2.476878	-0.73628	H	4.964781	-0.17364	0.861661
C	3.434456	1.091947	-0.13109	H	4.998988	-0.65401	-2.12533
O	5.929721	0.902317	-0.60799	H	5.530386	-1.72186	-0.85115
C	4.769178	0.182068	-0.15069	H	3.084694	-1.95272	-2.35395
C	4.716932	-1.05191	-1.14469	H	3.505231	-2.91569	-0.9351
C	3.373188	-1.88896	-1.29914	H	2.923539	0.274312	-2.04918
C	2.495483	0.328713	-1.04377	H	1.543251	0.836315	-1.17258
O	3.011246	-1.20909	0.869254	H	3.117357	-2.15857	1.016392
C	2.364099	-1.1043	-0.45944	H	-0.95157	-2.43519	-0.63061
C	0.912969	-1.32783	0.042427	H	-0.72378	-0.83883	-1.40083
C	-0.53813	-1.43139	-0.49937	H	-1.42743	-0.92403	2.914846
C	-1.3269	-1.52429	2.004321	H	-0.5554	-2.27717	2.185441
C	-0.97135	-0.62704	0.807042	H	-2.27299	-2.05124	1.844745
C	-2.08851	0.386633	0.592358	H	-3.46794	-1.20844	-0.09658
C	-3.38937	-0.14607	0.107572	H	-4.32983	1.718739	0.130667
C	-4.44437	0.657376	-0.08349	H	-6.6347	-1.44528	-1.11935
O	-5.76217	-1.17916	-0.79798	H	-5.38597	0.698798	-2.6598
C	-6.11905	0.966727	-1.89427	H	-7.11571	0.681309	-2.2492
C	-5.80296	0.234784	-0.57444	H	-6.10677	2.052609	-1.76168
C	-6.8585	0.567158	0.499422	H	-7.85492	0.282134	0.143918
H	0.82691	1.378018	2.358492	H	-6.64613	0.017611	1.420339
H	1.689383	-0.14335	2.566273	H	-6.87497	1.637459	0.725502

**1**-5*R*-conf. 4	Coordinates (Å)				Coordinates (Å)		
	**X**	**Y**	**Z**		**X**	**Y**	**Z**
O	-2.43064	-1.86818	-1.7421	H	0.934873	2.408869	0.521308
H	1.361113	-2.35223	0.965428	H	1.974051	1.905421	1.826549
H	0.063384	-0.16538	-0.69989	H	3.487346	2.648278	-1.81729
C	0.193222	-0.67514	0.258088	H	1.730713	2.755317	-1.58248
C	0.443995	0.442798	1.288135	H	2.826282	3.577657	-0.46902
C	1.527894	1.562644	0.885841	H	4.901486	2.946892	0.544678
C	2.693506	2.668711	-1.0671	H	4.164354	1.052668	1.445272
C	2.706051	1.399639	-0.19285	H	5.332941	0.15007	-1.19576
O	5.124338	2.175062	0.007654	H	5.75273	-0.38318	0.412263
C	4.200321	1.12727	0.357825	H	4.167309	-1.83763	-1.50192
C	4.90173	-0.16327	-0.23897	H	4.405752	-2.29858	0.185754
C	4.055804	-1.49055	-0.469	H	3.16275	0.143877	-1.87286
C	2.438837	0.181529	-1.05351	H	1.468917	0.226215	-1.54207
O	2.797427	-0.67575	1.276616	H	3.165466	-1.46178	1.702141
C	2.623795	-1.06321	-0.14297	H	0.231832	-3.64773	-0.73035
C	1.260133	-1.77972	0.039062	H	0.104129	-2.2427	-1.82308
C	0.196399	-2.55597	-0.78031	H	-1.89322	-1.98715	2.043477
C	-1.2779	-2.59824	1.376054	H	-0.39613	-2.91467	1.939222
C	-0.88906	-1.82952	0.102751	H	-1.85077	-3.49646	1.121049
C	-2.14848	-1.40282	-0.64013	H	-2.69751	-0.00221	0.991743
C	-3.00823	-0.38687	0.025511	H	-4.40608	-0.33057	-1.52464
C	-4.13026	0.067568	-0.54894	H	-4.79711	0.710733	1.937901
O	-4.66765	1.479467	1.363548	H	-6.56675	-0.34908	0.670295
C	-6.50193	0.554084	0.053816	H	-7.177	1.308167	0.468414
C	-5.0626	1.102742	0.038923	H	-6.84042	0.298356	-0.95463
C	-4.9809	2.391529	-0.79488	H	-5.6772	3.133422	-0.3928
H	-0.49205	0.99432	1.449931	H	-3.96837	2.801993	-0.75578
H	0.725195	0.004204	2.246563	H	-5.23996	2.198843	-1.83927

**1**-5*R*-conf. 5	Coordinates (Å)				Coordinates (Å)		
	**X**	**Y**	**Z**		**X**	**Y**	**Z**
O	-2.41784	-1.84054	-1.76052	H	0.949934	2.417133	0.499857
H	1.352364	-2.34338	0.973701	H	1.98006	1.918103	1.813892
H	0.073571	-0.15931	-0.7095	H	3.517162	2.631735	-1.82514
C	0.196187	-0.66441	0.251922	H	1.75968	2.747968	-1.60152
C	0.44713	0.458035	1.276914	H	2.852367	3.57192	-0.48628
C	1.537539	1.570873	0.873105	H	4.92005	2.936818	0.544277
C	2.718978	2.660118	-1.07983	H	4.168391	1.051421	1.449737
C	2.720697	1.396089	-0.19823	H	5.346727	0.128974	-1.18015
O	5.141196	2.161674	0.011335	H	5.755927	-0.3971	0.432978
C	4.210652	1.119943	0.362134	H	4.173915	-1.8554	-1.48096
C	4.909262	-0.17703	-0.22382	H	4.40111	-2.30755	0.210629
C	4.058463	-1.50176	-0.45071	H	3.180411	0.128715	-1.86864
C	2.452345	0.174352	-1.0534	H	1.48523	0.220676	-1.54739
O	2.794501	-0.67115	1.283396	H	3.156703	-1.45631	1.715497
C	2.626709	-1.06602	-0.13488	H	0.22512	-3.64197	-0.72126
C	1.258795	-1.77519	0.043935	H	0.109526	-2.24171	-1.82154
C	0.195241	-2.55032	-0.77684	H	-1.90382	-1.95767	2.034849
C	-1.28932	-2.57509	1.372587	H	-0.41167	-2.89421	1.94066
C	-0.891	-1.81454	0.097392	H	-1.86638	-3.4711	1.119293
C	-2.14395	-1.38251	-0.65341	H	-2.69539	0.020517	0.975311
C	-3.00724	-0.36911	0.011433	H	-4.43914	-0.36145	-1.50926
C	-4.1471	0.05921	-0.54782	H	-3.77058	2.166812	1.01478
O	-4.55486	1.650699	1.251828	H	-6.20783	-0.34971	1.222945
C	-6.3979	0.403315	0.453535	H	-7.09083	1.148961	0.854498
C	-5.08362	1.087731	0.045209	H	-6.86737	-0.08444	-0.4047
C	-5.34753	2.202557	-0.98456	H	-6.03377	2.939649	-0.55793
H	-0.4875	1.014348	1.431331	H	-4.41495	2.70965	-1.2549
H	0.72177	0.023798	2.239184	H	-5.79087	1.80117	-1.90064

**1**-5*R*-conf. 6	Coordinates (Å)				Coordinates (Å)		
	**X**	**Y**	**Z**		**X**	**Y**	**Z**
O	-1.91295	1.578126	0.821426	H	2.448469	2.407633	1.274675
H	0.983196	-2.14228	0.757872	H	3.473792	1.248048	2.074157
H	0.339702	0.740106	0.014069	H	4.233546	2.404839	-1.69808
C	0.47906	-0.03548	0.769201	H	2.79267	3.031411	-0.87453
C	1.402069	0.593507	1.829089	H	4.373704	3.08039	-0.07482
C	2.717187	1.34542	1.287437	H	6.673926	0.481323	-0.37928
C	3.731464	2.485257	-0.73057	H	4.942066	-0.18177	0.867461
C	3.436864	1.097164	-0.12742	H	4.987148	-0.61638	-2.13176
O	5.880918	1.015882	-0.52015	H	5.531545	-1.7052	-0.87647
C	4.760469	0.194441	-0.13915	H	3.08569	-1.93684	-2.37129
C	4.713266	-1.03224	-1.15652	H	3.510266	-2.90811	-0.9587
C	3.374353	-1.87965	-1.31613	H	2.918409	0.288957	-2.04892
C	2.495863	0.335666	-1.04106	H	1.544298	0.846024	-1.16067
O	3.010212	-1.21999	0.858604	H	3.110424	-2.17104	0.999422
C	2.364129	-1.10242	-0.4692	H	-0.95346	-2.42987	-0.64762
C	0.912126	-1.32805	0.031332	H	-0.72697	-0.8274	-1.40548
C	-0.53976	-1.42716	-0.50912	H	-1.4239	-0.94564	2.910448
C	-1.3246	-1.5392	1.995409	H	-0.55281	-2.29332	2.169987
C	-0.97079	-0.63305	0.804369	H	-2.27087	-2.06505	1.833349
C	-2.08852	0.381883	0.598486	H	-3.46805	-1.20908	-0.0996
C	-3.38934	-0.14784	0.110289	H	-4.32931	1.717153	0.141662
C	-4.44387	0.656891	-0.07785	H	-6.63389	-1.44037	-1.12521
O	-5.7617	-1.17604	-0.80147	H	-5.38116	0.708314	-2.65561
C	-6.11524	0.974162	-1.89033	H	-7.11159	0.690956	-2.2479
C	-5.80195	0.237097	-0.57267	H	-6.10217	2.059547	-1.7538
C	-6.8591	0.566136	0.500618	H	-7.85509	0.283023	0.142411
H	0.830227	1.358328	2.370636	H	-6.64863	0.013098	1.419876
H	1.700087	-0.16147	2.558485	H	-6.8753	1.635613	0.730584

**1**-5*R*-conf. 8	**Coordinates (Å)**				**Coordinates (Å)**		
	**X**	**Y**	**Z**		**X**	**Y**	**Z**
O	2.432856	1.868593	-1.74145	H	-0.92053	-2.40134	0.484487
H	-1.36031	2.354475	0.961542	H	-1.95452	-1.93574	1.807426
H	-0.06287	0.167616	-0.70368	H	-3.48803	-2.6366	-1.83511
C	-0.19417	0.675964	0.25489	H	-1.73257	-2.74197	-1.60225
C	-0.44671	-0.44435	1.282598	H	-2.83472	-3.57458	-0.49191
C	-1.52067	-1.56967	0.870144	H	-5.84856	-2.1383	0.522705
C	-2.69475	-2.66545	-1.0839	H	-4.13819	-1.04081	1.449517
C	-2.70828	-1.40322	-0.19842	H	-5.31618	-0.16696	-1.20337
O	-5.01633	-2.26764	0.048154	H	-5.75466	0.37395	0.40052
C	-4.1841	-1.13532	0.364881	H	-4.17322	1.838591	-1.50252
C	-4.89644	0.154197	-0.24439	H	-4.41247	2.29547	0.186691
C	-4.05976	1.490464	-0.47014	H	-3.16134	-0.14003	-1.8756
C	-2.44089	-0.18008	-1.05366	H	-1.46963	-0.22739	-1.53854
O	-2.79726	0.681713	1.275844	H	-3.15907	1.470685	1.701235
C	-2.62576	1.065704	-0.14451	H	-0.2305	3.648756	-0.73352
C	-1.26041	1.78145	0.03543	H	-0.10215	2.243424	-1.82585
C	-0.19554	2.556967	-0.78326	H	1.890723	1.987489	2.04307
C	1.276398	2.59883	1.374966	H	0.394208	2.9157	1.937221
C	0.888678	1.830166	0.101235	H	1.849968	3.496758	1.120519
C	2.148994	1.403156	-0.63996	H	2.695288	0.002384	0.99265
C	3.007496	0.387003	0.026887	H	4.407625	0.330151	-1.52123
C	4.130212	-0.06784	-0.54592	H	4.793017	-0.71131	1.942043
O	4.664083	-1.47992	1.367414	H	6.564836	0.348008	0.677404
C	6.500755	-0.55507	0.060724	H	7.174946	-1.30938	0.476339
C	5.06129	-1.1033	0.043441	H	6.840966	-0.29933	-0.94714
C	4.980559	-2.39206	-0.7905	H	5.676033	-3.13414	-0.38734
H	0.490384	-0.99265	1.449497	H	3.967858	-2.80225	-0.75294
H	-0.73586	-0.00852	2.239741	H	5.241261	-2.19944	-1.83449

**1**-5*R*-conf. 9	Coordinates (Å)				Coordinates (Å)		
	**X**	**Y**	**Z**		**X**	**Y**	**Z**
O	-1.91624	1.581006	0.874859	H	2.471437	2.400401	1.297991
H	0.980555	-2.14063	0.771931	H	3.48969	1.216391	2.07075
H	0.335804	0.742698	0.033008	H	4.228137	2.40378	-1.69496
C	0.477464	-0.03351	0.787123	H	2.789705	3.034158	-0.86655
C	1.407219	0.592924	1.842013	H	4.363869	3.080376	-0.06928
C	2.725612	1.334472	1.293631	H	6.166543	1.523706	0.068072
C	3.725063	2.48091	-0.72821	H	4.96397	-0.18236	0.840114
C	3.426762	1.091945	-0.13062	H	4.972654	-0.64089	-2.15056
O	5.917632	0.903334	-0.62975	H	5.513512	-1.71866	-0.88876
C	4.760271	0.180894	-0.16795	H	3.055102	-1.9359	-2.37274
C	4.698393	-1.04571	-1.17054	H	3.486357	-2.90979	-0.96464
C	3.352471	-1.88025	-1.31996	H	2.89923	0.288857	-2.05031
C	2.479504	0.336375	-1.04106	H	1.52683	0.845927	-1.15847
O	3.009364	-1.21617	0.856374	H	3.117973	-2.16673	0.994421
C	2.351371	-1.1007	-0.4661	H	-0.96671	-2.426	-0.62107
C	0.904244	-1.32625	0.046081	H	-0.74279	-0.82404	-1.38064
C	-0.5511	-1.42372	-0.48518	H	-1.41388	-0.94167	2.939286
C	-1.32196	-1.53489	2.023305	H	-0.55136	-2.29148	2.192239
C	-0.97306	-0.62907	0.830472	H	-2.27077	-2.05799	1.866494
C	-2.08988	0.388231	0.634395	H	-3.46569	-1.19559	-0.08283
C	-3.38908	-0.13417	0.132778	H	-4.32192	1.734078	0.136311
C	-4.43846	0.672136	-0.07689	H	-5.83363	-1.57145	0.145897
O	-5.86954	-1.18513	-0.74127	H	-5.23448	0.402158	-2.692
C	-5.99265	0.803007	-2.01398	H	-6.9819	0.518051	-2.38422
C	-5.78986	0.23823	-0.59868	H	-5.92025	1.893733	-2.0141
C	-6.89685	0.736456	0.349573	H	-7.87357	0.429057	-0.03483
H	0.841036	1.361544	2.383999	H	-6.7684	0.315318	1.352599
H	1.703939	-0.16216	2.57199	H	-6.88359	1.826852	0.437986

**1**-5*R*-conf. 11	Coordinates (Å)				Coordinates (Å)		
	**X**	**Y**	**Z**		**X**	**Y**	**Z**
O	-1.90709	1.602763	0.779293	H	2.470226	2.41775	1.265652
H	0.977311	-2.12802	0.785311	H	3.477903	1.24232	2.064986
H	0.344658	0.745787	0.000198	H	4.258139	2.377773	-1.70859
C	0.477398	-0.02003	0.7663	H	2.81203	3.021407	-0.90408
C	1.396859	0.619562	1.822317	H	4.377727	3.076258	-0.0909
C	2.722377	1.351413	1.278247	H	6.177691	1.517256	0.088045
C	3.745013	2.468767	-0.74833	H	4.962644	-0.17476	0.869335
C	3.438164	1.088533	-0.13509	H	5.002784	-0.67417	-2.11447
O	5.933948	0.889299	-0.60479	H	5.528141	-1.73536	-0.83224
C	4.77043	0.175034	-0.14571	H	3.08549	-1.96903	-2.33959
C	4.717272	-1.06507	-1.13202	H	3.500012	-2.92434	-0.91387
C	3.371626	-1.8995	-1.28453	H	2.929591	0.260207	-2.04925
C	2.499308	0.322069	-1.04521	H	1.548782	0.831486	-1.17951
O	3.006229	-1.20508	0.87874	H	3.111973	-2.1538	1.030961
C	2.362655	-1.10689	-0.45221	H	-0.95656	-2.42938	-0.62293
C	0.909795	-1.32346	0.047764	H	-0.72187	-0.8383	-1.40236
C	-0.54034	-1.42605	-0.49691	H	-1.43665	-0.89448	2.912072
C	-1.33555	-1.50086	2.005703	H	-0.56656	-2.25462	2.193493
C	-0.97428	-0.61238	0.803726	H	-2.28265	-2.02646	1.847472
C	-2.08713	0.403557	0.57873	H	-3.46928	-1.1926	-0.09903
C	-3.3911	-0.12797	0.099497	H	-4.34185	1.731041	0.136053
C	-4.45189	0.66976	-0.0837	H	-5.25853	-1.26786	-1.71007
O	-5.82003	-1.16186	-0.9284	H	-5.51942	0.968548	-2.60543
C	-6.23241	1.082911	-1.78166	H	-7.22009	0.766413	-2.12895
C	-5.81229	0.225851	-0.57247	H	-6.2772	2.144334	-1.52051
C	-6.8316	0.361231	0.570139	H	-7.8246	0.073568	0.212215
H	0.826124	1.396497	2.347477	H	-6.55462	-0.29227	1.401698
H	1.684331	-0.12587	2.565778	H	-6.87592	1.390855	0.934746

**1**-5*R*-conf. 12	Coordinates (Å)				Coordinates (Å)		
	X	Y	Z		X	Y	Z
O	-2.43232	-1.86445	-1.73742	H	0.93286	2.410691	0.518018
H	1.363671	-2.35564	0.961242	H	1.972552	1.913757	1.824663
H	0.064987	-0.1657	-0.69927	H	3.439777	2.619269	-1.87436
C	0.197096	-0.67562	0.258312	H	1.718246	2.784112	-1.54725
C	0.448518	0.443119	1.288051	H	2.898564	3.570042	-0.48331
C	1.528265	1.567194	0.884881	H	5.245707	2.355361	-0.76055
C	2.696803	2.669026	-1.06961	H	4.146241	1.029851	1.448911
C	2.707895	1.402378	-0.19182	H	5.321399	0.155403	-1.21601
O	5.084594	2.24496	0.186451	H	5.765247	-0.36563	0.389283
C	4.195689	1.133795	0.368715	H	4.17332	-1.836	-1.50524
C	4.903053	-0.15217	-0.24915	H	4.418544	-2.29016	0.183742
C	4.062768	-1.48655	-0.47298	H	3.16035	0.143795	-1.87291
C	2.439993	0.182471	-1.05048	H	1.469173	0.227691	-1.53661
O	2.801467	-0.68239	1.276848	H	3.155679	-1.47476	1.702304
C	2.628264	-1.0641	-0.14354	H	0.233063	-3.64669	-0.73523
C	1.263291	-1.7808	0.036282	H	0.103777	-2.23951	-1.82508
C	0.197812	-2.55483	-0.78307	H	-1.88729	-1.98969	2.044835
C	-1.27289	-2.60011	1.375988	H	-0.39068	-2.91806	1.937661
C	-0.88577	-1.82944	0.103421	H	-1.84662	-3.49753	1.120046
C	-2.14666	-1.39974	-0.6357	H	-2.68646	0.004892	0.996786
C	-3.00043	-0.38228	0.03389	H	-4.41606	-0.34819	-1.50166
C	-4.12976	0.061443	-0.53438	H	-5.1275	2.175727	1.662625
O	-4.52651	1.511046	1.29869	H	-6.39622	-0.36571	0.950927
C	-6.45662	0.46486	0.242622	H	-7.14934	1.214592	0.640972
C	-5.06345	1.093608	0.038405	H	-6.86903	0.092402	-0.69978
C	-5.14354	2.295969	-0.92387	H	-5.8395	3.044463	-0.52906
H	-0.48852	0.99218	1.452005	H	-4.15877	2.758061	-1.03294
H	0.733055	0.005156	2.24571	H	-5.50016	1.99308	-1.91284

**1**-5*R*-conf. 15	**Coordinates (Å)**				**Coordinates (Å)**		
	**X**	**Y**	**Z**		**X**	**Y**	**Z**
O	-1.91594	1.574883	0.885808	H	2.451948	2.398805	1.290904
H	0.979061	-2.14606	0.754404	H	3.483603	1.234557	2.075338
H	0.334346	0.742535	0.036854	H	4.215832	2.414423	-1.69457
C	0.477993	-0.03878	0.785317	H	2.781489	3.036784	-0.85649
C	1.410079	0.581338	1.842519	H	4.368348	3.07941	-0.06812
C	2.720899	1.336586	1.295138	H	6.6638	0.479609	-0.40861
C	3.720862	2.488988	-0.72294	H	4.940851	-0.19033	0.847583
C	3.429505	1.097178	-0.12682	H	4.962557	-0.60373	-2.15497
O	5.870344	1.016271	-0.53855	H	5.515363	-1.70198	-0.91162
C	4.752027	0.193203	-0.15492	H	3.057788	-1.92031	-2.38929
C	4.69573	-1.02617	-1.18062	H	3.492195	-2.90224	-0.98711
C	3.354665	-1.87107	-1.33603	H	2.89544	0.302938	-2.04979
C	2.480727	0.342985	-1.03841	H	1.528802	0.855114	-1.14722
O	3.008118	-1.22682	0.846215	H	3.109883	-2.17892	0.978635
C	2.351939	-1.0989	-0.47569	H	-0.96821	-2.42051	-0.63937
C	0.903641	-1.32643	0.034433	H	-0.74504	-0.81258	-1.38654
C	-0.55224	-1.41941	-0.49614	H	-1.41134	-0.96294	2.933077
C	-1.32015	-1.54949	2.012728	H	-0.54926	-2.3071	2.175554
C	-0.97254	-0.6349	0.826303	H	-2.26899	-2.07168	1.853121
C	-2.08972	0.383597	0.638284	H	-3.46655	-1.19633	-0.08557
C	-3.38933	-0.13594	0.134785	H	-4.32147	1.73271	0.146147
C	-4.43832	0.671725	-0.07161	H	-5.83567	-1.57176	0.142837
O	-5.86996	-1.1825	-0.74311	H	-5.2331	0.410912	-2.68814
C	-5.99136	0.81	-2.00918	H	-6.98061	0.526929	-2.38084
C	-5.7897	0.24036	-0.59566	H	-5.9182	1.900673	-2.00564
C	-6.89705	0.735985	0.35352	H	-7.87367	0.430257	-0.03247
H	0.843419	1.34243	2.394627	H	-6.76942	0.311667	1.355303
H	1.713712	-0.17934	2.56358	H	-6.88332	1.826087	0.44534

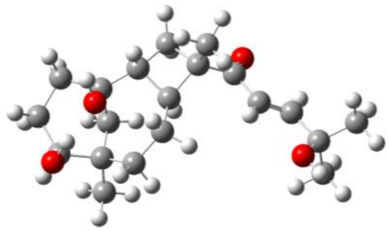

1-5*S*-conf. 1 (8.47%)

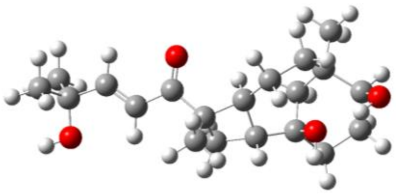

1-5*S*-conf. 2 (16.05%)

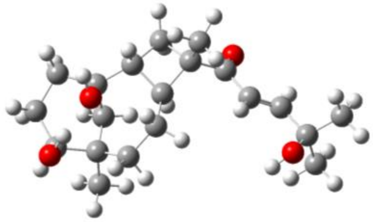

1-5*S*-conf. 3 (10.24%)

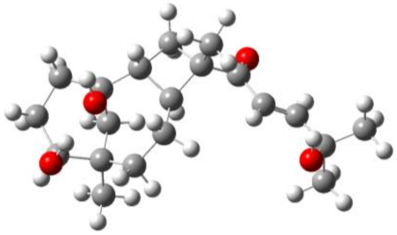

1-5*S*-conf. 4 (8.42%)

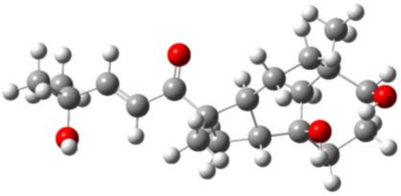

1-5*S*-conf. 7 (27.29%)

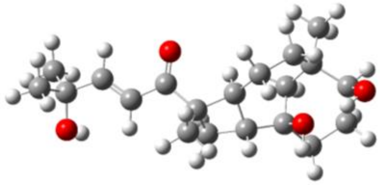

1-5*S*-conf. 8 (18.05%)

Figure S27. The cartesian coordinates of the dominant conformers for conformers **1**-5*S*.

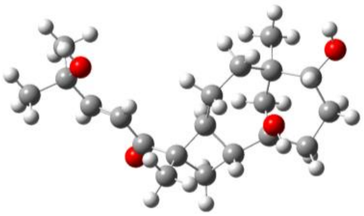

1-5*R*-conf. 1 (5.69%)

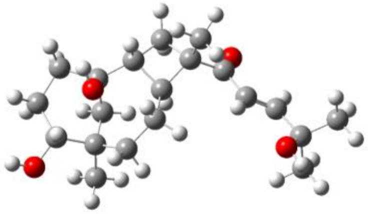

1-5*R*-conf. 2 (2.82%)

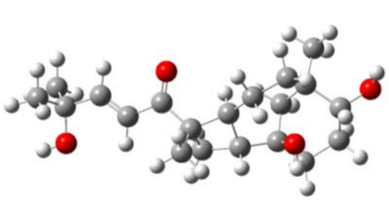

1-5*R*-conf. 3 (12.41%)

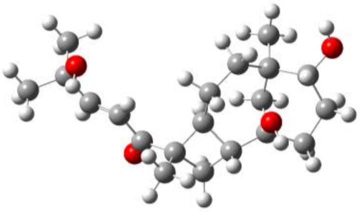

1-5*R*-conf. 4 (10.62%)

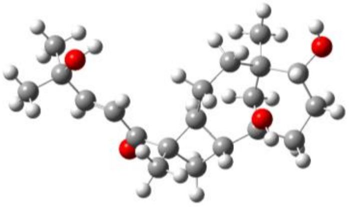

1-5*R*-conf. 5 (8.48%)

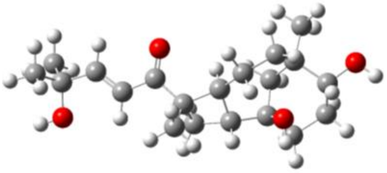

1-5*R*-conf. 6 (5.26%)

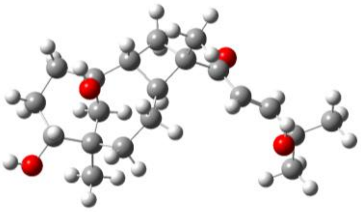

1-5*R*-conf. 8 (5.21%)

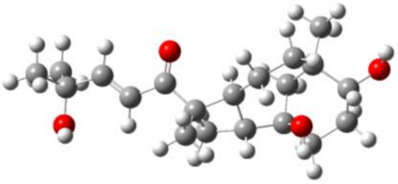

1-5*R*-conf. 9 (14.52%)

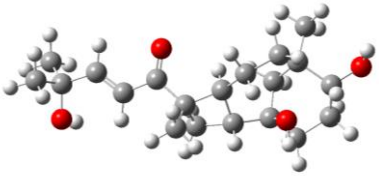

1-5*R*-conf. 11 (20.16%)

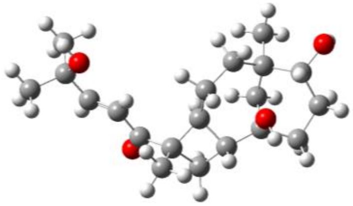

1-5*R*-conf. 12 (6.66%)

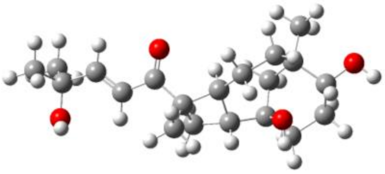

1-5*R*-conf. 15 (6.25%)

Figure S28. The cartesian coordinates of the dominant conformers for conformers **1**-5*R*.

Table S14. The cartesian coordinates of the dominant conformers for compound **2**

**2**-1*R*3*R*9*R*-conf. 1	**Coordinates (Å)**				**Coordinates (Å)**		
	**X**	**Y**	**Z**		**X**	**Y**	**Z**
O	-1.61269	1.110535	1.982398	H	0.969288	-2.63405	-0.44351
H	1.88854	2.597503	-0.9793	H	2.581001	-3.6191	1.079678
H	0.348798	1.097466	-1.95632	H	3.085437	-2.49926	2.355962
C	0.446737	0.930653	-0.88065	H	1.37771	-2.61276	1.902474
C	0.324322	-0.62986	-0.6389	H	4.641979	-2.37046	0.447607
C	1.49405	-1.70149	-0.68431	H	4.214365	-1.60616	-1.06595
C	2.401404	-2.62647	1.509118	H	5.886964	-0.21306	-0.06366
C	2.61632	-1.52638	0.455354	H	5.228273	-0.39112	1.540892
C	4.135175	-1.4994	0.01448	H	4.749615	1.822395	-0.35905
C	4.945578	-0.23214	0.494722	H	4.325848	1.716926	1.351254
C	4.254073	1.194572	0.390826	H	1.340412	-0.04002	1.372472
C	2.371438	-0.15707	1.050381	H	2.970507	-0.01314	1.954715
O	2.994274	0.363276	-1.36392	H	3.288453	1.117897	-1.89146
C	2.803002	0.903789	0.006285	H	0.66975	3.468563	0.942593
C	1.610999	1.814502	-0.263	H	0.687342	1.899569	1.767092
C	0.636016	2.385535	0.791593	H	-1.72868	3.681377	-0.31751
C	-1.18736	2.950262	-0.9281	H	-1.88325	2.539369	-1.66527
C	-0.56025	1.854314	-0.04734	H	-0.40577	3.487062	-1.47487
C	-1.64873	1.145013	0.753592	H	-2.7062	0.505965	-1.09385
C	-2.75041	0.501066	-0.01073	H	-3.7957	-0.07216	1.704698
C	-3.77983	-0.08388	0.61625	H	-5.49983	-1.12839	-1.88218
O	-4.75353	-0.67953	-1.46142	H	-7.10701	-0.57514	-0.10305
C	-6.25275	-0.06742	0.358434	H	-6.3972	-0.08294	1.442836
C	-4.94274	-0.77338	-0.04506	H	-6.241	0.972435	0.02112
C	-4.9668	-2.25399	0.385379	H	-5.05964	-2.35293	1.47097
O	1.979341	-1.92668	-1.99861	H	-5.82207	-2.76184	-0.07409
H	-0.18461	-0.80549	0.314862	H	-4.04964	-2.75523	0.064893
H	-0.38237	-0.98433	-1.39853	H	2.447965	-1.09915	-2.20318

**2-**1*R*3*R*9*R*-conf. 2	Coordinates (Å)				Coordinates (Å)		
	**X**	**Y**	**Z**		**X**	**Y**	**Z**
O	-1.61663	1.10453	1.995139	H	0.986769	-2.64027	-0.42643
H	1.869814	2.59526	-0.98498	H	2.61483	-3.61044	1.088806
H	0.336326	1.080805	-1.94997	H	3.120427	-2.48348	2.358318
C	0.439873	0.919417	-0.87402	H	1.410545	-2.60923	1.916435
C	0.328172	-0.64064	-0.62467	H	4.663653	-2.3506	0.439793
C	1.504235	-1.70536	-0.67386	H	4.221453	-1.59404	-1.07347
C	2.431702	-2.61767	1.516388	H	5.244345	-0.3641	1.522849
C	2.632742	-1.51952	0.4579	H	5.891399	-0.1869	-0.08654
C	4.148525	-1.48424	0.007094	H	4.327169	1.737541	1.332493
C	4.953836	-0.21025	0.478061	H	4.739209	1.84039	-0.38076
C	4.252554	1.211691	0.374211	H	1.353363	-0.03839	1.378683
C	2.382993	-0.14993	1.05021	H	2.986969	0.000658	1.950164
O	2.986796	0.366522	-1.36963	H	3.276716	1.120259	-1.90078
C	2.800969	0.910352	0.000044	H	0.656148	3.463754	0.941792
C	1.601476	1.812533	-0.26495	H	0.686377	1.89651	1.769136
C	0.62799	2.380312	0.792923	H	-1.74671	3.661091	-0.31211
C	-1.20463	2.930259	-0.92229	H	-1.90185	2.513092	-1.65475
C	-0.5685	1.840495	-0.04002	H	-0.42801	3.468444	-1.47468
C	-1.65167	1.130191	0.766484	H	-2.69379	0.459632	-1.07671
C	-2.75055	0.474962	0.006903	H	-3.8351	-0.03656	1.717306
C	-3.79825	-0.08153	0.629655	H	-4.00402	-1.4897	-1.6041
O	-4.7489	-0.88691	-1.46452	H	-7.07378	-0.43645	-0.35395
C	-6.22613	0.07681	0.109716	H	-6.45367	0.243681	1.165818
C	-4.95675	-0.77484	-0.05142	H	-6.09338	1.046803	-0.37683
C	-5.15675	-2.17269	0.564308	H	-5.36188	-2.10912	1.63708
O	1.981996	-1.93181	-1.99058	H	-5.99947	-2.67044	0.076122
H	-0.17347	-0.81538	0.333191	H	-4.2612	-2.78865	0.428185
H	-0.38091	-1.00269	-1.3786	H	2.444605	-1.10231	-2.20085

**2**-1*R*3*R*9*R*-conf. 3	Coordinates (Å)				Coordinates (Å)		
	**X**	**Y**	**Z**		**X**	**Y**	**Z**
O	-1.61598	1.122511	1.991187	H	0.969699	-2.63651	-0.4247
H	1.878816	2.594065	-0.98787	H	2.59127	-3.6128	1.093811
H	0.338616	1.086108	-1.95281	H	3.100697	-2.4868	2.362668
C	0.440507	0.925157	-0.87664	H	1.390775	-2.60485	1.918695
C	0.320988	-0.63408	-0.62593	H	4.646988	-2.36363	0.445839
C	1.491996	-1.70441	-0.67247	H	4.210225	-1.6068	-1.06885
C	2.412399	-2.61865	1.520005	H	5.236285	-0.3787	1.527046
C	2.620051	-1.52273	0.460479	H	5.885739	-0.20644	-0.08191
C	4.13655	-1.49531	0.011484	H	4.32918	1.726995	1.333523
C	4.947523	-0.22466	0.481802	H	4.743695	1.826059	-0.3794
C	4.253166	1.200523	0.375679	H	1.346756	-0.03445	1.377994
C	2.376233	-0.15129	1.050931	H	2.979851	-0.00259	1.951455
O	2.985652	0.359519	-1.3688	H	3.278014	1.111766	-1.90073
C	2.800619	0.90573	0.000078	H	0.667115	3.470371	0.936641
C	1.605816	1.81336	-0.26736	H	0.689409	1.90392	1.765737
C	0.634062	2.386886	0.788962	H	-1.73441	3.677375	-0.31935
C	-1.19404	2.944066	-0.92812	H	-1.89104	2.529881	-1.66244
C	-0.56434	1.851738	-0.04436	H	-0.41386	3.478948	-1.47868
C	-1.65133	1.147398	0.762508	H	-2.70545	0.494852	-1.08025
C	-2.75398	0.497274	0.003799	H	-3.79741	-0.08222	1.717968
C	-3.78271	-0.09114	0.628867	H	-5.0255	0.28774	-1.68689
O	-4.88113	-0.64594	-1.47486	H	-7.10953	-0.71109	-0.0087
C	-6.27082	-0.20415	0.476974	H	-6.36072	-0.33776	1.559049
C	-4.94384	-0.78217	-0.05008	H	-6.33961	0.868017	0.263107
C	-4.8633	-2.29319	0.220736	H	-4.87387	-2.49938	1.294217
O	1.970548	-1.93474	-1.98826	H	-5.71925	-2.79471	-0.24052
H	-0.18265	-0.80538	0.331462	H	-3.94386	-2.7037	-0.20516
H	-0.38908	-0.99344	-1.38012	H	2.437188	-1.10766	-2.19899

**2**-1*R*3*R*9*R*-conf. 8	Coordinates (Å)				Coordinates (Å)		
	**X**	**Y**	**Z**		**X**	**Y**	**Z**
O	1.63217	1.141693	-1.96748	H	-0.98465	-2.64121	0.411
H	-1.88464	2.587858	1.002796	H	-2.60331	-3.60506	-1.11829
H	-0.35285	1.069975	1.965544	H	-3.1062	-2.47054	-2.38213
C	-0.44993	0.916433	0.887855	H	-1.39799	-2.59488	-1.93343
C	-0.33262	-0.64132	0.62715	H	-4.65853	-2.35514	-0.47023
C	-1.50602	-1.70966	0.662795	H	-4.22545	-1.60865	1.050606
C	-2.42091	-2.60883	-1.53807	H	-5.23986	-0.36243	-1.5412
C	-2.62975	-1.51873	-0.47279	H	-5.89427	-0.19841	0.066591
C	-4.14772	-1.49065	-0.02877	H	-4.32856	1.739993	-1.33198
C	-4.95424	-0.21535	-0.4941	H	-4.74881	1.8298	0.379977
C	-4.25707	1.207607	-0.37708	H	-1.3501	-0.02776	-1.37712
C	-2.38091	-0.1443	-1.05416	H	-2.98108	0.011184	-1.9558
O	-2.99701	0.353079	1.366452	H	-3.29398	1.101442	1.901275
C	-2.8065	0.907277	0.001702	H	-0.66367	3.472542	-0.91225
C	-1.61067	1.810745	0.278849	H	-0.6851	1.910696	-1.74973
C	-0.63324	2.3883	-0.77009	H	1.72887	3.670473	0.356023
C	1.18776	2.932984	0.958936	H	1.883906	2.51448	1.691794
C	0.560093	1.846565	0.066108	H	0.406242	3.463519	1.511708
C	1.651682	1.148401	-0.73894	H	2.657268	0.452754	1.105606
C	2.744488	0.480888	0.024313	H	3.827904	-0.0066	-1.68921
C	3.795596	-0.06671	-0.60186	H	4.332814	-2.56832	-0.52044
O	5.127572	-2.04099	-0.6875	H	5.682411	-1.62745	1.873006
C	4.820912	-1.05505	1.51933	H	4.770837	-0.12285	2.089562
C	4.971734	-0.79039	0.017864	H	3.914312	-1.63222	1.7298
C	6.259313	-0.0082	-0.27654	H	6.246986	0.962845	0.225068
O	-1.98917	-1.94699	1.975568	H	7.122201	-0.57886	0.080157
H	0.174174	-0.80755	-0.32959	H	6.372901	0.154096	-1.35213
H	0.373397	-1.00747	1.381969	H	-2.45566	-1.12065	2.18964

**2**-1*R*3*R*9*R*-conf. 9	Coordinates (Å)				Coordinates (Å)		
	**X**	**Y**	**Z**		**X**	**Y**	**Z**
O	1.627248	1.124208	-1.985	H	-0.96859	-2.63382	0.450585
H	-1.89294	2.599066	0.966495	H	-2.584	-3.62762	-1.06316
H	-0.35327	1.102534	1.950212	H	-3.09292	-2.51399	-2.34307
C	-0.45043	0.932858	0.874946	H	-1.38389	-2.62385	-1.8937
C	-0.32593	-0.62796	0.636787	H	-4.64453	-2.37811	-0.43163
C	-1.49419	-1.7012	0.689222	H	-4.21381	-1.60742	1.077755
C	-2.4065	-2.63678	-1.49753	H	-5.2361	-0.40402	-1.53113
C	-2.61975	-1.53223	-0.44811	H	-5.89007	-0.21962	0.074646
C	-4.13749	-1.50485	-0.00329	H	-4.33467	1.705522	-1.35338
C	-4.9504	-0.24031	-0.48652	H	-4.75401	1.818275	0.35747
C	-4.25988	1.187496	-0.39086	H	-1.34798	-0.04868	-1.37529
C	-2.37795	-0.16529	-1.04973	H	-2.9796	-0.02581	-1.95301
O	-2.99448	0.364599	1.363939	H	-3.29284	1.119793	1.888308
C	-2.8076	0.899588	-0.00899	H	-0.67758	3.461238	-0.96234
C	-1.61582	1.812666	0.253861	H	-0.6922	1.887166	-1.77672
C	-0.6419	2.379356	-0.80426	H	1.715333	3.688891	0.300803
C	1.177777	2.9581	0.914955	H	1.875929	2.553905	1.653894
C	0.554676	1.855609	0.038859	H	0.394057	3.493418	1.459939
C	1.648135	1.149519	-0.7567	H	2.664323	0.496071	1.097498
C	2.743947	0.497202	0.015284	H	3.81297	-0.03693	-1.69315
C	3.786321	-0.0738	-0.6046	H	6.19978	0.729417	-0.28691
O	6.166878	-0.20116	-0.55251	H	5.892647	-2.72669	-0.04671
C	4.983788	-2.24199	-0.4161	H	4.114819	-2.77319	-0.01937
C	4.972515	-0.77172	0.025245	H	4.971783	-2.31572	-1.50723
C	5.026897	-0.65626	1.55185	H	4.165324	-1.13824	2.023101
O	-1.97519	-1.92162	2.005745	H	5.934842	-1.14221	1.918456
H	0.180645	-0.80573	-0.31796	H	5.046252	0.391992	1.868072
H	0.382688	-0.97965	1.395991	H	-2.44524	-1.09436	2.208148

**2**-1*R*3*R*9*R*-conf. 15	Coordinates (Å)				Coordinates (Å)		
	**X**	**Y**	**Z**		**X**	**Y**	**Z**
O	-1.85486	-0.96014	1.973269	H	2.155274	1.259199	2.51143
H	1.55304	-2.4145	-1.31175	H	3.161422	3.268644	1.596754
H	1.213417	-2.38402	1.032004	H	2.684762	3.564231	-0.08334
C	0.827185	-1.48177	0.550868	H	1.474536	3.050919	1.103101
C	1.072861	-0.2834	1.552019	H	4.79092	2.286911	-0.01212
C	2.413197	0.54735	1.71773	H	4.85549	0.600763	0.447181
C	2.515254	2.916185	0.784234	H	4.205477	1.893224	-2.23705
C	2.815687	1.446309	0.443879	H	5.400066	0.673325	-1.88376
C	4.283382	1.322101	-0.13369	H	2.863645	0.253876	-3.1366
C	4.367822	0.969449	-1.67141	H	3.934061	-1.02996	-2.56771
C	3.384042	-0.13495	-2.25428	H	0.880744	0.978438	-0.39591
C	1.928326	0.963363	-0.68182	H	1.998377	1.630092	-1.54666
O	3.35247	-1.08039	-0.14556	H	3.582831	-1.92939	-0.54641
C	2.418251	-0.44308	-1.10913	H	-0.59381	-1.71876	-2.24586
C	1.25015	-1.41492	-0.97763	H	-0.45128	-0.10228	-1.53165
C	-0.22059	-1.1601	-1.38227	H	-1.9881	-3.33035	-0.53731
C	-1.0439	-3.17327	-0.00399	H	-1.15629	-3.55461	1.015538
C	-0.659	-1.67863	0.02574	H	-0.27229	-3.76208	-0.50915
C	-1.79923	-0.96697	0.744832	H	-2.79582	-0.45136	-1.1712
C	-2.8692	-0.36262	-0.09224	H	-3.96553	0.3249	1.546888
C	-3.91864	0.262004	0.460299	H	-4.10341	1.417201	-1.9209
O	-4.8548	0.842955	-1.71323	H	-6.07357	2.810913	-0.38561
C	-5.23953	2.35722	0.157339	H	-5.44657	2.41763	1.22989
C	-5.06337	0.895736	-0.29694	H	-4.33383	2.939445	-0.04433
C	-6.34741	0.089783	-0.04185	H	-6.57752	0.048142	1.025934
O	3.465298	-0.2158	2.289407	H	-7.18576	0.562084	-0.56238
H	0.279553	0.459533	1.429089	H	-6.23225	-0.93138	-0.4149
H	0.891688	-0.70346	2.547014	H	3.694087	-0.83897	1.5785

**2**-1*R*3*R*9*S*-conf. 1	Coordinates (Å)				Coordinates (Å)		
	**X**	**Y**	**Z**		**X**	**Y**	**Z**
O	1.962131	2.020678	-1.51469	H	-1.4905	-2.49761	1.517566
H	-0.57007	1.032075	-1.53764	H	-3.04745	-3.78533	0.212406
H	-1.0549	1.168577	1.467528	H	-3.1948	-3.2462	-1.4685
C	-0.4573	0.7668	0.647774	H	-1.596	-3.38534	-0.72055
C	-0.38223	-0.74732	0.957308	H	-4.8869	-2.1213	-0.06698
C	-1.77794	-1.4805	1.227549	H	-4.49008	-0.836	1.049008
C	-2.6381	-3.09726	-0.5364	H	-5.73094	0.203546	-0.69745
C	-2.74372	-1.63849	-0.05679	H	-5.00148	-0.76105	-1.95423
C	-4.27066	-1.22229	0.055424	H	-4.29736	1.988759	-1.20014
C	-4.7724	-0.19336	-1.04605	H	-3.67954	1.03255	-2.55
C	-3.83458	1.031133	-1.46587	H	-1.14784	-0.85212	-1.27107
C	-2.21022	-0.69705	-1.10325	H	-2.68193	-0.89141	-2.07073
O	-3.04517	0.949071	0.684427	H	-3.26689	1.886432	0.765613
C	-2.53587	0.784377	-0.69309	H	-0.11465	3.354116	-1.06498
C	-1.10661	1.362299	-0.64368	H	-1.05003	3.301403	0.462116
C	-0.4581	2.716839	-0.24889	H	1.771282	3.398365	1.620142
C	1.182403	2.49506	1.814916	H	1.818865	1.793982	2.363593
C	0.644758	1.895582	0.508986	H	0.348064	2.770572	2.467607
C	1.804711	1.505096	-0.40945	H	2.534566	0.010795	1.065003
C	2.737035	0.464428	0.101727	H	3.976347	0.552736	-1.57714
C	3.802449	0.077868	-0.61308	H	5.062787	-2.14949	1.321328
O	4.414456	-1.47789	1.068062	H	5.539209	-2.86633	-0.98184
C	4.796953	-2.10751	-1.25335	H	5.043613	-1.73694	-2.25271
C	4.805561	-0.96994	-0.21242	H	3.811552	-2.57974	-1.28783
C	6.204261	-0.32757	-0.11852	H	6.507887	0.110166	-1.0741
O	-2.40579	-0.9539	2.387111	H	6.209852	0.456255	0.643578
H	0.170664	-0.87248	1.897089	H	6.945022	-1.087	0.155491
H	0.178087	-1.30775	0.200176	H	-2.76395	-0.09719	2.091325

**2**-1*R*3*R*9*S*-conf. 2	Coordinates (Å)				Coordinates (Å)		
	**X**	**Y**	**Z**		**X**	**Y**	**Z**
O	-1.54101	-0.93123	1.299506	H	3.353145	-2.35558	1.251872
H	0.200443	1.114764	0.407966	H	4.997444	-1.12198	2.500301
H	1.212002	-1.06133	-1.45699	H	4.489552	0.51783	2.937571
C	0.710024	-0.81841	-0.51866	H	3.365276	-0.83706	3.12633
C	1.446012	-1.69429	0.522885	H	5.701849	0.546325	0.782788
C	3.039749	-1.59763	0.524503	H	4.991397	-0.02594	-0.70844
C	4.153409	-0.42254	2.486126	H	5.165841	2.337093	-0.95346
C	3.651273	-0.1977	1.048673	H	4.728858	2.662324	0.703528
C	4.773409	0.534164	0.198915	H	2.982458	2.825892	-1.65209
C	4.463919	2.049246	-0.16455	H	2.615872	3.27854	0.015149
C	2.988248	2.465302	-0.61687	H	1.648799	0.403975	1.565336
C	2.514859	0.789743	1.034362	H	2.793624	1.703341	1.567394
O	2.83187	0.347176	-1.47826	H	2.578482	0.738867	-2.325
C	2.17426	1.178949	-0.44942	H	-1.17788	1.446041	-1.56888
C	0.694563	0.745844	-0.49537	H	-0.02951	0.554106	-2.60225
C	-0.41025	0.667991	-1.58226	H	-2.21347	-1.61382	-2.4163
C	-1.23398	-1.83499	-1.98101	H	-1.2984	-2.81438	-1.49387
C	-0.782	-0.75685	-0.98589	H	-0.51241	-1.91112	-2.80044
C	-1.81123	-0.63202	0.138173	H	-3.34571	0.129143	-1.27926
C	-3.16491	-0.14672	-0.24635	H	-3.93352	-0.32833	1.687025
C	-4.14775	-0.04363	0.658195	H	-6.53989	1.073904	-1.16438
O	-5.64034	0.759074	-0.99949	H	-7.57173	-0.34755	0.567578
C	-6.5453	-0.69147	0.736604	H	-6.45978	-0.99372	1.784608
C	-5.54979	0.434935	0.392519	H	-6.36111	-1.56407	0.104286
C	-5.83053	1.689744	1.243588	H	-5.71955	1.481223	2.311792
O	3.577761	-2.05891	-0.70764	H	-5.1418	2.493489	0.970027
H	1.239237	-2.74477	0.279803	H	-6.85631	2.034673	1.071977
H	1.066861	-1.53941	1.536599	H	3.399678	-1.32933	-1.3285

**2-**1*R*3*R*9*S*-conf. 4	Coordinates (Å)				Coordinates (Å)		
	**X**	**Y**	**Z**		**X**	**Y**	**Z**
O	1.957486	2.023089	-1.52437	H	-1.48159	-2.50218	1.50573
H	-0.57444	1.038174	-1.54037	H	-3.04463	-3.78509	0.202686
H	-1.04703	1.164199	1.467252	H	-3.19887	-3.23971	-1.47557
C	-0.45263	0.7655	0.643718	H	-1.59701	-3.38178	-0.73478
C	-0.37591	-0.74969	0.947408	H	-4.88504	-2.12015	-0.06264
C	-1.77051	-1.48404	1.220879	H	-4.48354	-0.8391	1.05657
C	-2.6383	-3.09429	-0.54524	H	-5.73176	0.207256	-0.68046
C	-2.74186	-1.6373	-0.05979	H	-5.00803	-0.75275	-1.94406
C	-4.26833	-1.22158	0.060597	H	-4.30033	1.994171	-1.18322
C	-4.77485	-0.18849	-1.03479	H	-3.68844	1.042761	-2.53908
C	-3.83878	1.037444	-1.45432	H	-1.15121	-0.84659	-1.27807
C	-2.21285	-0.69208	-1.10512	H	-2.68869	-0.88294	-2.07127
O	-3.03964	0.947519	0.692181	H	-3.26663	1.883348	0.776314
C	-2.53666	0.787869	-0.68818	H	-0.11685	3.358559	-1.06181
C	-1.10712	1.365484	-0.64304	H	-1.04626	3.300947	0.468699
C	-0.45706	2.718677	-0.24641	H	1.779205	3.394186	1.617879
C	1.189743	2.490992	1.811339	H	1.826123	1.787954	2.357599
C	0.648657	1.895368	0.504957	H	0.356829	2.765725	2.466096
C	1.805167	1.507872	-0.41863	H	2.543403	0.018393	1.054523
C	2.743081	0.469366	0.088223	H	3.967004	0.535252	-1.60259
C	3.802187	0.072469	-0.63041	H	4.732908	-0.71546	1.727301
O	4.555225	-1.44792	1.119432	H	5.402205	-2.96342	-0.83628
C	4.656854	-2.21265	-1.11502	H	4.803035	-1.94806	-2.16554
C	4.805108	-0.97849	-0.21083	H	3.660167	-2.64687	-0.99929
C	6.229814	-0.40043	-0.30681	H	6.459543	-0.07561	-1.3259
O	-2.39324	-0.96191	2.385224	H	6.34318	0.464356	0.355985
H	0.180808	-0.87832	1.884457	H	6.955158	-1.16379	-0.01115
H	0.181116	-1.30729	0.185742	H	-2.75351	-0.10441	2.094342

**2-**1*R*3*R*9*S*-conf. 5	Coordinates (Å)				Coordinates (Å)		
	**X**	**Y**	**Z**		**X**	**Y**	**Z**
O	1.955175	2.019009	-1.5273	H	-1.48521	-2.50832	1.495957
H	-0.57952	1.040683	-1.54039	H	-3.0538	-3.78387	0.192709
H	-1.04405	1.157541	1.468898	H	-3.21156	-3.23251	-1.48325
C	-0.45247	0.760692	0.642478	H	-1.60799	-3.37995	-0.74731
C	-0.37785	-0.75572	0.940948	H	-4.89196	-2.11475	-0.06207
C	-1.77309	-1.48866	1.215445	H	-4.4852	-0.83822	1.060396
C	-2.64826	-3.09124	-0.55395	H	-5.73613	0.21614	-0.66986
C	-2.74793	-1.63573	-0.06327	H	-5.01723	-0.74077	-1.93857
C	-4.27332	-1.21771	0.062573	H	-4.30278	2.002298	-1.16992
C	-4.7808	-0.18002	-1.02797	H	-3.69618	1.054627	-2.53076
C	-3.84365	1.045733	-1.44562	H	-1.15907	-0.84366	-1.28316
C	-2.21997	-0.68787	-1.10674	H	-2.69873	-0.87453	-2.07225
O	-3.0389	0.946796	0.698433	H	-3.26434	1.882641	0.786496
C	-2.53995	0.791193	-0.68383	H	-0.11595	3.358393	-1.05562
C	-1.1092	1.366062	-0.64058	H	-1.04176	3.298074	0.476921
C	-0.45547	2.716747	-0.24132	H	1.785717	3.382553	1.619679
C	1.196186	2.47913	1.811749	H	1.834104	1.773537	2.352942
C	0.650482	1.889077	0.504811	H	0.365697	2.751923	2.470402
C	1.804035	1.501706	-0.42238	H	2.534719	-0.00068	1.040959
C	2.739686	0.459164	0.079936	H	4.000464	0.573064	-1.58144
C	3.815147	0.084004	-0.62599	H	3.611324	-2.13572	0.806336
O	4.414404	-1.63021	0.9991	H	5.707644	-2.7622	-1.04238
C	4.978975	-2.00347	-1.34177	H	5.327433	-1.52866	-2.26375
C	4.813721	-0.97228	-0.20932	H	4.02669	-2.50008	-1.55733
C	6.158441	-0.30159	0.114476	H	6.534739	0.256019	-0.74717
O	-2.39165	-0.96959	2.383211	H	6.04295	0.388676	0.954339
H	0.180943	-0.8884	1.876256	H	6.893636	-1.06524	0.385135
H	0.17597	-1.31177	0.175691	H	-2.75116	-0.11037	2.096447

**2-**1*R*3*R*9*S*-conf. 9	Coordinates (Å)				Coordinates (Å)		
	**X**	**Y**	**Z**		**X**	**Y**	**Z**
O	-1.53735	-0.92881	1.310691	H	3.356199	-2.35437	1.25174
H	0.201198	1.11402	0.411927	H	5.003054	-1.11854	2.495528
H	1.207697	-1.06345	-1.45384	H	4.495535	0.521646	2.93181
C	0.708258	-0.81993	-0.51432	H	3.372168	-0.83339	3.124788
C	1.44716	-1.69446	0.526393	H	5.703276	0.547222	0.773649
C	3.041002	-1.59722	0.524342	H	4.988666	-0.02687	-0.71502
C	4.158741	-0.41944	2.482306	H	5.163033	2.336399	-0.96242
C	3.653338	-0.19654	1.04572	H	4.7285	2.662913	0.694952
C	4.77327	0.534294	0.192266	H	2.977659	2.822558	-1.65814
C	4.462566	2.048837	-0.17211	H	2.614389	3.277642	0.008941
C	2.985869	2.46343	-0.62231	H	1.651905	0.405049	1.566204
C	2.516589	0.790546	1.032768	H	2.796177	1.704972	1.563932
O	2.827562	0.343054	-1.47895	H	2.585663	0.741216	-2.32592
C	2.172535	1.177203	-0.45083	H	-1.18239	1.44358	-1.56189
C	0.692722	0.744258	-0.49252	H	-0.03672	0.550196	-2.59699
C	-0.41491	0.665349	-1.57626	H	-2.22201	-1.6184	-2.40132
C	-1.24058	-1.83829	-1.96966	H	-1.30237	-2.81721	-1.48131
C	-0.78512	-0.75903	-0.97738	H	-0.52235	-1.91466	-2.79194
C	-1.81034	-0.63235	0.149691	H	-3.34608	0.127134	-1.26541
C	-3.16563	-0.14669	-0.2307	H	-3.94053	-0.34285	1.69775
C	-4.15215	-0.04844	0.670559	H	-5.16355	1.705281	-1.04828
O	-5.69581	0.901482	-0.95871	H	-7.55879	-0.38419	0.353146
C	-6.53744	-0.73782	0.522476	H	-6.48228	-1.18282	1.519428
C	-5.55614	0.437896	0.389595	H	-6.30469	-1.50823	-0.2175
C	-5.91965	1.565471	1.374278	H	-5.86032	1.222514	2.41155
O	3.576295	-2.0594	-0.70862	H	-5.23731	2.414985	1.26016
H	1.240284	-2.74523	0.284767	H	-6.93933	1.908713	1.177459
H	1.07022	-1.53863	1.540796	H	3.396885	-1.33033	-1.32973

**2-**1*R*3*R*9*S*-conf. 11	**Coordinates (Å)**				**Coordinates (Å)**		
	**X**	**Y**	**Z**		**X**	**Y**	**Z**
O	1.963202	2.026451	-1.49913	H	-1.49662	-2.50979	1.500121
H	-0.57429	1.037794	-1.53303	H	-3.05473	-3.78856	0.187386
H	-1.06005	1.156382	1.472853	H	-3.20165	-3.23893	-1.49013
C	-0.4621	0.759644	0.651001	H	-1.60299	-3.38375	-0.7431
C	-0.38772	-0.75652	0.951055	H	-4.8931	-2.12188	-0.08212
C	-1.78399	-1.49079	1.216741	H	-4.49601	-0.84399	1.042203
C	-2.6449	-3.09614	-0.55713	H	-5.73571	0.207696	-0.69742
C	-2.74974	-1.64027	-0.06852	H	-5.00719	-0.74947	-1.9604
C	-4.27648	-1.22399	0.046217	H	-4.30116	1.994988	-1.18952
C	-4.77755	-0.18776	-1.04867	H	-3.68368	1.046703	-2.54495
C	-3.8389	1.038675	-1.46091	H	-1.15336	-0.84749	-1.27813
C	-2.21562	-0.69276	-1.10918	H	-2.68744	-0.88085	-2.07781
O	-3.0486	0.942568	0.688847	H	-3.28013	1.877237	0.773342
C	-2.54028	0.786364	-0.68972	H	-0.11807	3.357255	-1.04614
C	-1.11061	1.363124	-0.63712	H	-1.05464	3.295666	0.479731
C	-0.46223	2.715192	-0.23412	H	1.76267	3.38861	1.640994
C	1.17553	2.483121	1.830399	H	1.81266	1.78094	2.377073
C	0.639896	1.890087	0.520397	H	0.33977	2.753347	2.483345
C	1.800841	1.505602	-0.39822	H	2.508458	0.007955	1.069571
C	2.734277	0.459641	0.10942	H	3.982201	0.574005	-1.55677
C	3.806951	0.082055	-0.60059	H	6.205064	0.374148	0.254107
O	6.143138	-0.34412	-0.39234	H	5.51406	-2.20912	1.389021
C	4.700583	-1.50679	1.189384	H	3.751207	-2.03353	1.323201
C	4.844864	-0.95646	-0.233	H	4.751099	-0.7023	1.930659
C	4.813506	-2.08955	-1.26806	H	3.863931	-2.62912	-1.2248
O	-2.41111	-0.97144	2.379788	H	4.94454	-1.69071	-2.27796
H	0.164554	-0.88822	1.890391	H	5.627958	-2.79164	-1.06511
H	0.171627	-1.31278	0.19004	H	-2.77014	-0.11314	2.089766

**2-**1*R*3*R*9*S*-conf. 13	Coordinates (Å)				Coordinates (Å)		
	**X**	**Y**	**Z**		**X**	**Y**	**Z**
O	1.96072	2.030322	-1.47564	H	-1.50649	-2.53	1.461918
H	-0.57433	1.059076	-1.51834	H	-3.0704	-3.78363	0.131656
H	-1.05775	1.13465	1.489392	H	-3.21656	-3.20856	-1.53737
C	-0.46171	0.748342	0.661215	H	-1.6179	-3.37019	-0.79382
C	-0.39207	-0.77234	0.938488	H	-4.90304	-2.10675	-0.11127
C	-1.79067	-1.50592	1.194067	H	-4.50069	-0.84666	1.031119
C	-2.65865	-3.08166	-0.60278	H	-5.73809	0.234131	-0.69224
C	-2.75795	-1.63287	-0.09244	H	-5.01308	-0.70662	-1.96954
C	-4.28318	-1.21299	0.029599	H	-4.29777	2.023777	-1.15792
C	-4.78134	-0.15917	-1.0497	H	-3.68426	1.093981	-2.52797
C	-3.83885	1.070199	-1.44407	H	-1.15966	-0.82757	-1.29122
C	-2.22128	-0.67181	-1.11923	H	-2.69439	-0.84375	-2.09022
O	-3.0477	0.939401	0.703522	H	-3.27193	1.87438	0.803282
C	-2.54065	0.801991	-0.67755	H	-0.10966	3.369031	-0.99819
C	-1.10895	1.372955	-0.6174	H	-1.04548	3.289073	0.52726
C	-0.45559	2.716692	-0.19517	H	1.772897	3.35448	1.68946
C	1.181237	2.449252	1.86554	H	1.814273	1.736654	2.403394
C	0.644056	1.877068	0.546946	H	0.34598	2.714418	2.521195
C	1.803	1.501518	-0.37784	H	2.517182	-0.00818	1.074849
C	2.739437	0.45252	0.118174	H	3.97938	0.582008	-1.55246
C	3.811991	0.085187	-0.5974	H	4.110814	-2.2869	-1.51111
O	4.963386	-1.84236	-1.39636	H	6.982712	-1.07231	0.032241
C	6.218805	-0.30307	-0.11645	H	6.236928	0.380848	0.735985
C	4.84227	-0.96853	-0.25268	H	6.465382	0.258534	-1.02196
C	4.503481	-1.78948	0.995537	H	4.468106	-1.16346	1.89196
O	-2.41512	-1.00199	2.365293	H	3.534224	-2.28785	0.889495
H	0.160383	-0.91995	1.875309	H	5.269098	-2.55579	1.142109
H	0.164991	-1.31892	0.168768	H	-2.77151	-0.13825	2.088471

**2**-1*R*3*S*9*R*-conf. 1	Coordinates (Å)				Coordinates (Å)		
	**X**	**Y**	**Z**		**X**	**Y**	**Z**
O	1.499796	-1.09958	1.999494	H	-2.05523	1.434378	-1.83506
H	-1.94901	-2.67391	-0.99025	H	-2.57601	3.611959	0.908173
H	-0.36968	-1.23948	-1.98661	H	-3.06417	2.489807	2.182243
C	-0.48862	-1.02136	-0.92217	H	-1.35956	2.61372	1.710312
C	-0.36402	0.54704	-0.76064	H	-4.64653	2.376301	0.269783
C	-1.5489	1.57567	-0.88075	H	-4.23077	1.537912	-1.216
C	-2.3861	2.620362	1.331333	H	-5.92226	0.222217	-0.15954
C	-2.62506	1.501973	0.303738	H	-5.25446	0.44204	1.435657
C	-4.15016	1.482878	-0.12991	H	-4.81678	-1.83836	-0.39441
C	-4.97804	0.244554	0.394232	H	-4.38776	-1.6851	1.311055
C	-4.3079	-1.19535	0.333883	H	-3.00515	0.047368	1.851935
C	-2.40466	0.148472	0.942903	H	-1.3753	0.028547	1.270529
O	-3.02443	-0.4359	-1.44423	H	-3.36679	-1.19363	-1.93793
C	-2.8542	-0.93866	-0.06799	H	-0.75421	-3.50245	0.965974
C	-1.6712	-1.87426	-0.29281	H	-0.78905	-1.91752	1.758167
C	-0.71718	-2.4226	0.793659	H	1.868796	-2.64178	-1.58862
C	1.154766	-3.03235	-0.85758	H	0.39059	-3.59051	-1.40761
C	0.497681	-1.91204	-0.03075	H	1.682397	-3.74041	-0.20902
C	1.561423	-1.17097	0.773692	H	2.642198	-0.56452	-1.06952
C	2.676919	-0.54292	0.014906	H	3.728076	0.026295	1.728097
C	3.712769	0.033711	0.639018	H	3.959609	1.369072	-1.63215
O	4.702275	0.771371	-1.46207	H	7.006483	0.346081	-0.29823
C	6.148514	-0.15	0.165209	H	6.356907	-0.28694	1.229531
C	4.885861	0.701495	-0.04282	H	6.019808	-1.13323	-0.29515
C	5.0822	2.116506	0.533512	H	5.270529	2.08433	1.610766
O	-0.85843	2.840697	-0.91145	H	5.934544	2.596027	0.04378
H	0.135515	0.787588	0.184602	H	4.191619	2.73163	0.365268
H	0.342301	0.8659	-1.53481	H	-1.50502	3.510982	-1.16899

**2**-1*R*3*S*9*R*-conf. 2	Coordinates (Å)				Coordinates (Å)		
	**X**	**Y**	**Z**		**X**	**Y**	**Z**
O	1.495936	-1.10983	1.985809	H	-2.04984	1.433599	-1.83815
H	-1.96881	-2.67327	-0.9871	H	-2.54118	3.619795	0.903685
H	-0.38308	-1.2523	-1.99248	H	-3.03124	2.503459	2.182254
C	-0.49606	-1.03122	-0.92798	H	-1.32813	2.614109	1.701593
C	-0.35954	0.536603	-0.77014	H	-4.62387	2.39818	0.278076
C	-1.53781	1.573135	-0.88663	H	-4.22176	1.554298	-1.20836
C	-2.35648	2.627609	1.327735	H	-5.91732	0.252655	-0.14111
C	-2.60861	1.50924	0.303283	H	-5.23987	0.470295	1.450299
C	-4.136	1.50051	-0.12259	H	-4.82793	-1.81623	-0.37811
C	-4.97015	0.269069	0.407823	H	-4.38923	-1.66326	1.324978
C	-4.31075	-1.17574	0.346566	H	-1.36473	0.028449	1.266345
C	-2.39481	0.155292	0.943701	H	-2.99154	0.060071	1.855865
O	-3.03094	-0.42866	-1.43926	H	-3.37985	-1.18522	-1.93013
C	-2.85723	-0.93026	-0.06316	H	-0.7711	-3.50753	0.964803
C	-1.68199	-1.87462	-0.29209	H	-0.7913	-1.92128	1.755019
C	-0.72719	-2.42814	0.79093	H	1.846189	-2.67012	-1.60217
C	1.133408	-3.05382	-0.86639	H	0.362694	-3.60841	-1.41095
C	0.488029	-1.92727	-0.03909	H	1.659614	-3.76386	-0.21877
C	1.55879	-1.19002	0.760195	H	2.658874	-0.62054	-1.08529
C	2.678538	-0.57522	-0.00242	H	3.68475	0.063288	1.713271
C	3.693609	0.033577	0.625071	H	5.470619	0.982283	-1.87182
O	4.715839	0.547701	-1.45119	H	7.036365	0.506495	-0.03466
C	6.172655	0.015046	0.42696	H	6.291497	0.075153	1.512896
C	4.870728	0.69936	-0.03555	H	6.171673	-1.03777	0.132292
C	4.880085	2.196228	0.335273	H	4.945192	2.339123	1.418
O	-0.83918	2.833534	-0.92308	H	5.745179	2.688671	-0.12256
H	0.146004	0.775253	0.172295	H	3.969749	2.680431	-0.02794
H	0.345808	0.848887	-1.54775	H	-1.48198	3.507086	-1.18155

**2-**1*R*3*S*9*R*-conf. 3	Coordinates (Å)				Coordinates (Å)		
	**X**	**Y**	**Z**		**X**	**Y**	**Z**
O	1.480657	-1.10885	1.98452	H	-2.07038	1.440682	-1.83946
H	-1.98492	-2.67559	-0.98945	H	-2.50803	3.619757	0.901108
H	-0.38511	-1.27582	-2.00041	H	-3.00154	2.503236	2.187516
C	-0.49867	-1.04338	-0.93841	H	-1.30088	2.608768	1.70037
C	-0.36411	0.528988	-0.79714	H	-4.60395	2.414859	0.293623
C	-1.54669	1.577715	-0.89475	H	-4.22008	1.56553	-1.19427
C	-2.33087	2.630571	1.330174	H	-5.9117	0.275654	-0.10463
C	-2.59285	1.516879	0.302109	H	-5.21236	0.489158	1.477727
C	-4.12439	1.514044	-0.10964	H	-4.83438	-1.79763	-0.35858
C	-4.95725	0.287238	0.431702	H	-4.37694	-1.64912	1.339703
C	-4.30593	-1.16067	0.361145	H	-2.9633	0.068183	1.855668
C	-2.3768	0.160405	0.936845	H	-1.3437	0.029964	1.248057
O	-3.0371	-0.426	-1.43951	H	-3.40273	-1.17967	-1.92262
C	-2.85507	-0.92445	-0.06302	H	-0.78121	-3.51471	0.95609
C	-1.68853	-1.8779	-0.29744	H	-0.80004	-1.93083	1.75074
C	-0.73522	-2.43493	0.785276	H	1.848802	-2.68179	-1.59616
C	1.133425	-3.0639	-0.86204	H	0.365713	-3.62149	-1.40777
C	0.482439	-1.9353	-0.04181	H	1.657915	-3.77083	-0.20961
C	1.54855	-1.19348	0.759493	H	2.654978	-0.62701	-1.08315
C	2.670404	-0.57898	-0.00032	H	3.669342	0.064535	1.71773
C	3.682206	0.032767	0.629628	H	5.466193	0.981123	-1.86246
O	4.708046	0.550234	-1.4441	H	7.026315	0.50518	-0.02351
C	6.161489	0.013329	0.435623	H	6.278355	0.071472	1.521849
C	4.860719	0.699166	-0.02793	H	6.160431	-1.03898	0.139106
C	4.870383	2.195399	0.345422	H	4.934412	2.336434	1.428437
O	-0.93869	2.882902	-0.89095	H	5.736135	2.688207	-0.11075
H	0.16205	0.779352	0.130294	H	3.960658	2.680796	-0.01782
H	0.329299	0.825287	-1.59481	H	-0.4521	2.967856	-1.72171

**2**-1*R*3*S*9*R*-conf. 5	Coordinates (Å)				Coordinates (Å)		
	**X**	**Y**	**Z**		**X**	**Y**	**Z**
O	1.497616	-1.11182	1.996128	H	-2.04373	1.437733	-1.8326
H	-1.95736	-2.67191	-0.99197	H	-2.5557	3.614931	0.912987
H	-0.37192	-1.24314	-1.98804	H	-3.04896	2.493518	2.185702
C	-0.48927	-1.02627	-0.92316	H	-1.34374	2.610381	1.713934
C	-0.35697	0.541133	-0.75855	H	-4.6314	2.389771	0.272333
C	-1.53693	1.575621	-0.87802	H	-4.21905	1.551159	-1.21431
C	-2.37021	2.621986	1.334963	H	-5.91715	0.242194	-0.15986
C	-2.6141	1.505874	0.306033	H	-5.24877	0.457086	1.435789
C	-4.1391	1.494484	-0.12825	H	-4.82105	-1.82309	-0.39703
C	-4.973	0.259509	0.394214	H	-4.39254	-1.67407	1.308938
C	-4.30959	-1.18346	0.332429	H	-3.00169	0.051462	1.852519
C	-2.40031	0.150762	0.943876	H	-1.37169	0.025831	1.271791
O	-3.02213	-0.42805	-1.44435	H	-3.3659	-1.18439	-1.93923
C	-2.85451	-0.93313	-0.06825	H	-0.76631	-3.50697	0.964245
C	-1.67577	-1.87393	-0.29414	H	-0.79272	-1.92178	1.756224
C	-0.72397	-2.42731	0.791793	H	1.855282	-2.66142	-1.59547
C	1.141941	-3.0473	-0.86135	H	0.37301	-3.60194	-1.40825
C	0.493154	-1.92293	-0.03332	H	1.667917	-3.75772	-0.214
C	1.561384	-1.18752	0.770687	H	2.660596	-0.61225	-1.07152
C	2.682913	-0.56926	0.012508	H	3.684799	0.083086	1.725227
C	3.696432	0.045727	0.636691	H	4.994162	-0.43335	-1.62854
O	4.846664	0.50896	-1.4615	H	7.038248	0.638	0.053567
C	6.187536	0.153369	0.541162	H	6.251645	0.335185	1.618033
C	4.874224	0.707408	-0.04288	H	6.259989	-0.9273	0.37703
C	4.788258	2.228703	0.159778	H	4.774352	2.481431	1.223241
O	-0.84056	2.837195	-0.90713	H	5.654938	2.709988	-0.30304
H	0.142798	0.7774	0.187595	H	3.878991	2.619949	-0.30429
H	0.351815	0.857963	-1.5312	H	-1.48427	3.511161	-1.16227

**2-**1*R*3*S*9*R*-conf. 7	Coordinates (Å)				Coordinates (Å)		
	**X**	**Y**	**Z**		**X**	**Y**	**Z**
O	-1.50409	1.118902	1.974315	H	2.059934	-1.44204	-1.83039
H	1.970411	2.670086	-0.99754	H	2.552086	-3.6139	0.922277
H	0.389409	1.241699	-2.00019	H	3.038536	-2.49069	2.196052
C	0.500829	1.025568	-0.93457	H	1.336178	-2.60685	1.7142
C	0.366937	-0.54179	-0.76957	H	4.633048	-2.39113	0.293176
C	1.54693	-1.57741	-0.87881	H	4.230968	-1.5549	-1.19761
C	2.364915	-2.62009	1.341435	H	5.922857	-0.24513	-0.13432
C	2.616116	-1.50599	0.312117	H	5.24385	-0.45673	1.457252
C	4.143928	-1.49627	-0.1122	H	4.829735	1.820583	-0.38221
C	4.97507	-0.26086	0.413539	H	4.389501	1.674612	1.320954
C	4.312989	1.182361	0.344784	H	2.994195	-0.04888	1.858471
C	2.398889	-0.14951	0.945998	H	1.368165	-0.02334	1.266896
O	3.035877	0.424606	-1.439	H	3.389923	1.177526	-1.93179
C	2.860413	0.93226	-0.06521	H	0.767631	3.509585	0.949528
C	1.683972	1.873721	-0.29989	H	0.789897	1.926416	1.74561
C	0.726305	2.429557	0.779552	H	-1.84342	2.660301	-1.61959
C	-1.13223	3.046506	-0.88341	H	-0.36114	3.599723	-1.42866
C	-0.48627	1.92359	-0.05109	H	-1.66004	3.758145	-0.23898
C	-1.55793	1.189562	0.748769	H	-2.62384	0.599457	-1.09912
C	-2.67472	0.563923	-0.01579	H	-3.6974	-0.03142	1.700836
C	-3.7024	-0.02528	0.611369	H	-4.2191	-2.52137	0.365998
O	-5.01024	-2.01743	0.606534	H	-5.67837	-1.42315	-1.89168
C	-4.80656	-0.86997	-1.53267	H	-4.78567	0.101611	-2.03499
C	-4.89648	-0.71676	-0.01105	H	-3.90614	-1.42237	-1.82126
C	-6.17784	0.025358	0.392971	H	-6.19523	1.031376	-0.0341
O	0.849319	-2.83839	-0.90964	H	-7.04973	-0.52723	0.029804
H	-0.13999	-0.77712	0.173048	H	-6.24808	0.105359	1.481491
H	-0.33572	-0.85932	-1.54762	H	1.493511	-3.51335	-1.16093

**2**-1*R*3*S*9*R*-conf. 8	Coordinates (Å)				Coordinates (Å)		
	**X**	**Y**	**Z**		**X**	**Y**	**Z**
O	1.483862	-1.08978	1.986169	H	-2.06969	1.42847	-1.84296
H	-1.97449	-2.67654	-0.98191	H	-2.49688	3.622252	0.867061
H	-0.38266	-1.26775	-1.99427	H	-3.04094	2.53914	2.160126
C	-0.49665	-1.03808	-0.93179	H	-1.33401	2.596596	1.727519
C	-0.36497	0.53443	-0.792	H	-4.62239	2.402943	0.281234
C	-1.55056	1.57566	-0.90074	H	-4.22916	1.553461	-1.20367
C	-2.35071	2.635605	1.315339	H	-5.91837	0.256092	-0.11912
C	-2.60784	1.511999	0.297809	H	-5.22786	0.477154	1.466163
C	-4.13734	1.503519	-0.11856	H	-4.83039	-1.81284	-0.36167
C	-4.96673	0.273723	0.421931	H	-4.38012	-1.65722	1.337951
C	-4.30794	-1.17129	0.358288	H	-1.35489	0.034222	1.254223
C	-2.38762	0.160053	0.93927	H	-2.9765	0.065894	1.856526
O	-3.03624	-0.43265	-1.43831	H	-3.39862	-1.18737	-1.92205
C	-2.85662	-0.9287	-0.06129	H	-0.77288	-3.50619	0.969375
C	-1.68385	-1.87615	-0.29065	H	-0.79429	-1.91891	1.757312
C	-0.72917	-2.42709	0.793977	H	1.849436	-2.67557	-1.59286
C	1.136522	-3.05715	-0.85613	H	0.367937	-3.61643	-1.39887
C	0.486844	-1.92803	-0.03583	H	1.663459	-3.76249	-0.20398
C	1.553381	-1.18293	0.761793	H	2.659247	-0.62456	-1.0837
C	2.67606	-0.5738	-0.00102	H	3.679952	0.069606	1.714301
C	3.691035	0.035527	0.626245	H	5.47447	0.971446	-1.87085
O	4.715636	0.544166	-1.45003	H	7.035938	0.492715	-0.0332
C	6.169933	0.005384	0.428496	H	6.288915	0.065367	1.514412
C	4.871297	0.695586	-0.03453	H	6.164047	-1.04751	0.134197
C	4.88769	2.192558	0.335752	H	4.953234	2.335593	1.418437
O	-0.95545	2.875162	-1.0403	H	5.755147	2.680723	-0.1221
H	0.156195	0.775568	0.143677	H	3.979875	2.681123	-0.02804
H	0.327474	0.842506	-1.58276	H	-0.41027	3.042289	-0.25894

**2-**1*R*3*S*9*R*-conf. 11	Coordinates (Å)				Coordinates (Å)		
	**X**	**Y**	**Z**		**X**	**Y**	**Z**
O	1.481294	-1.08225	1.997059	H	-2.08166	1.440029	-1.8364
H	-1.96001	-2.67743	-0.99163	H	-2.5571	3.609804	0.90469
H	-0.37061	-1.25956	-1.99386	H	-3.04389	2.486193	2.187434
C	-0.49084	-1.03032	-0.93193	H	-1.34236	2.61108	1.708012
C	-0.37207	0.542988	-0.78695	H	-4.63602	2.385127	0.284658
C	-1.56441	1.580541	-0.88873	H	-4.23585	1.542149	-1.20253
C	-2.37089	2.622075	1.333261	H	-5.92019	0.233593	-0.12411
C	-2.61616	1.507256	0.302387	H	-5.23171	0.451711	1.462381
C	-4.14541	1.489803	-0.1175	H	-4.82111	-1.82869	-0.37468
C	-4.96887	0.253894	0.417497	H	-4.37369	-1.67777	1.325994
C	-4.30275	-1.18734	0.348457	H	-2.98029	0.052472	1.851779
C	-2.38999	0.151993	0.936174	H	-1.35725	0.031304	1.2526
O	-3.03148	-0.43715	-1.44435	H	-3.39025	-1.19236	-1.93016
C	-2.85216	-0.93597	-0.06792	H	-0.75763	-3.50618	0.959541
C	-1.67509	-1.87754	-0.29737	H	-0.79473	-1.92275	1.754517
C	-0.72119	-2.42601	0.789207	H	1.873338	-2.64674	-1.58358
C	1.158762	-3.03633	-0.85255	H	0.398023	-3.59982	-1.40192
C	0.494771	-1.91478	-0.03298	H	1.687221	-3.7395	-0.19931
C	1.551954	-1.16546	0.77249	H	2.645116	-0.57231	-1.06796
C	2.672507	-0.54289	0.016488	H	3.71301	0.036976	1.732577
C	3.7049	0.036927	0.643391	H	3.970003	1.356029	-1.63584
O	4.709983	0.757876	-1.45611	H	7.005506	0.338116	-0.27288
C	6.143554	-0.15349	0.187919	H	6.344135	-0.28315	1.254643
C	4.883601	0.698168	-0.03512	H	6.016749	-1.13981	-0.26633
C	5.078029	2.117064	0.532286	H	5.25926	2.092336	1.610934
O	-0.96833	2.891365	-0.87987	H	5.934222	2.591938	0.044742
H	0.146837	0.796587	0.143777	H	4.189425	2.732237	0.353964
H	0.322049	0.848005	-1.58081	H	-0.4919	2.988141	-1.71516

**2-**1*R*3*S*9*R*-conf. 12	Coordinates (Å)				Coordinates (Å)		
	X	Y	Z		X	Y	Z
O	1.481706	-1.10632	1.995089	H	-2.06382	1.445684	-1.83346
H	-1.97053	-2.6745	-0.99431	H	-2.52884	3.613841	0.910944
H	-0.37276	-1.26416	-1.99476	H	-3.02533	2.491256	2.190835
C	-0.49125	-1.0367	-0.93225	H	-1.32216	2.605652	1.714446
C	-0.36181	0.535269	-0.7832	H	-4.21848	1.560134	-1.20201
C	-1.54692	1.580955	-0.88474	H	-4.61505	2.403605	0.285866
C	-2.34995	2.624124	1.338051	H	-5.91262	0.261009	-0.12749
C	-2.60092	1.512657	0.304895	H	-5.22468	0.472415	1.460152
C	-4.12971	1.505697	-0.11694	H	-4.82664	-1.80804	-0.37963
C	-4.96185	0.27438	0.415302	H	-4.38029	-1.66238	1.321812
C	-4.305	-1.17107	0.345036	H	-2.97645	0.057986	1.851666
C	-2.38437	0.155011	0.936936	H	-1.35283	0.027188	1.254402
O	-3.0271	-0.42636	-1.44516	H	-3.38947	-1.1788	-1.93261
C	-2.85234	-0.92843	-0.0693	H	-0.77459	-3.5127	0.957128
C	-1.68099	-1.8771	-0.29909	H	-0.80056	-1.92926	1.752541
C	-0.73074	-2.43273	0.787089	H	1.858122	-2.67122	-1.58949
C	1.142748	-3.05561	-0.85654	H	0.376669	-3.61354	-1.40414
C	0.488579	-1.92951	-0.03525	H	1.667604	-3.76281	-0.20471
C	1.55172	-1.1887	0.770413	H	2.659173	-0.62027	-1.06919
C	2.676254	-0.57261	0.014757	H	3.669671	0.087704	1.729358
C	3.686292	0.045953	0.641053	H	4.994457	-0.4378	-1.61867
O	4.84369	0.504446	-1.45424	H	7.030151	0.637684	0.068623
C	6.177796	0.153742	0.554011	H	6.237896	0.337744	1.63075
C	4.866469	0.706147	-0.03588	H	6.251227	-0.92722	0.392341
C	4.779703	2.227895	0.163067	H	4.762051	2.482984	1.225903
O	-0.94261	2.88781	-0.87215	H	5.647951	2.708213	-0.29779
H	0.157237	0.78315	0.148936	H	3.872063	2.618269	-0.30493
H	0.336009	0.836956	-1.575	H	-0.45294	2.97834	-1.70051

**2-**1*R*3*S*9*S*-conf. 1	**Coordinates (Å)**				**Coordinates (Å)**		
	**X**	**Y**	**Z**		**X**	**Y**	**Z**
O	-1.92538	-2.1874	-1.35189	H	2.363919	0.82415	1.918701
H	0.620748	-1.24866	-1.41618	H	1.650407	3.202951	-0.9651
H	1.071855	-1.14395	1.589119	H	3.10382	3.695	-0.0911
C	0.480272	-0.80005	0.739082	H	3.249386	2.958637	-1.69085
C	0.430692	0.736745	0.930666	H	4.510164	0.789902	1.058713
C	1.842203	1.414718	1.161381	H	4.944057	1.98883	-0.14848
C	2.687852	2.926986	-0.75051	H	5.777122	-0.38128	-0.57624
C	2.788792	1.522375	-0.13177	H	5.065989	0.473728	-1.91925
C	4.314414	1.108936	0.034983	H	4.340723	-2.19765	-0.94027
C	4.823793	-0.01226	-0.96783	H	3.741274	-1.35816	-2.37218
C	3.882921	-1.26415	-1.29005	H	2.750791	0.601965	-2.0711
C	2.265508	0.493116	-1.09707	H	1.20658	0.640807	-1.29312
O	3.050533	-0.98857	0.836404	H	3.366508	-1.89337	0.964505
C	2.577734	-0.94933	-0.55208	H	0.122692	-3.51596	-0.75315
C	1.139089	-1.50846	-0.48862	H	1.038929	-3.34231	0.777552
C	0.464022	-2.8151	0.010134	H	-1.83626	-1.64156	2.499906
C	-1.20463	-2.39629	2.02117	H	-0.38418	-2.62957	2.70707
C	-0.63788	-1.91731	0.677899	H	-1.8066	-3.30307	1.893971
C	-1.77728	-1.58778	-0.28846	H	-2.5087	0.028462	1.050356
C	-2.70335	-0.4982	0.123285	H	-3.92183	-0.70652	-1.56041
C	-3.75515	-0.15898	-0.63432	H	-5.02507	2.216169	1.109233
O	-4.38311	1.517898	0.919921	H	-5.4494	2.770291	-1.24716
C	-4.71088	1.986152	-1.44814	H	-4.94172	1.548035	-2.4237
C	-4.75369	0.925155	-0.32992	H	-3.71945	2.444428	-1.4955
C	-6.16099	0.304428	-0.22071	H	-6.44925	-0.19713	-1.14931
O	1.52399	2.702377	1.714954	H	-6.19064	-0.42351	0.594431
H	-0.1249	0.945968	1.852926	H	-6.89934	1.08777	-0.01686
H	-0.10604	1.257011	0.128389	H	2.349555	3.089925	2.035204

**2**-1*R*3*S*9*S*-conf. 2	Coordinates (Å)				Coordinates (Å)		
	**X**	**Y**	**Z**		**X**	**Y**	**Z**
O	-1.92499	-2.19909	-1.36248	H	2.34879	0.831788	1.91842
H	0.62836	-1.25543	-1.42053	H	1.651823	3.19876	-0.97957
H	1.061593	-1.13984	1.587026	H	3.098888	3.695142	-0.09745
C	0.474411	-0.79974	0.732493	H	3.255886	2.953029	-1.69343
C	0.422175	0.73768	0.918333	H	4.500122	0.795366	1.072018
C	1.831269	1.418502	1.155248	H	4.940853	1.990433	-0.13651
C	2.687994	2.924385	-0.75684	H	5.778706	-0.38033	-0.55077
C	2.785971	1.522136	-0.13227	H	5.075321	0.469267	-1.90126
C	4.310803	1.110625	0.045922	H	4.34631	-2.19937	-0.91767
C	4.827559	-0.01356	-0.94968	H	3.755133	-1.36544	-2.35608
C	3.889858	-1.26745	-1.27344	H	2.760931	0.594691	-2.06845
C	2.269673	0.488881	-1.09711	H	1.211882	0.634943	-1.30047
O	3.04311	-0.98509	0.846729	H	3.367421	-1.88644	0.978097
C	2.579678	-0.95138	-0.54497	H	0.127568	-3.52096	-0.7525
C	1.141095	-1.51173	-0.48883	H	1.03536	-3.34162	0.782474
C	0.464189	-2.81734	0.010341	H	-1.85146	-1.63766	2.483415
C	-1.21573	-2.39318	2.011259	H	-0.39885	-2.62221	2.702779
C	-0.64198	-1.91852	0.669339	H	-1.81543	-3.30142	1.884031
C	-1.77708	-1.59337	-0.30296	H	-2.49596	0.038934	1.019409
C	-2.70095	-0.49779	0.099074	H	-3.95259	-0.73493	-1.55645
C	-3.76611	-0.16907	-0.64453	H	-3.53718	2.157072	0.59864
O	-4.35139	1.684563	0.82557	H	-5.61514	2.657719	-1.3134
C	-4.89565	1.866465	-1.54248	H	-5.245	1.320631	-2.42391
C	-4.75217	0.932259	-0.32598	H	-3.93502	2.329874	-1.79215
C	-6.10834	0.310041	0.043701	H	-6.48822	-0.31274	-0.77042
O	1.507047	2.707844	1.701259	H	-6.00743	-0.30873	0.939333
H	-0.1391	0.94968	1.836575	H	-6.83382	1.103991	0.244391
H	-0.10968	1.254599	0.110515	H	2.329604	3.098389	2.025542

**2**-1*R*3*S*9*S*-conf. 4	Coordinates (Å)				Coordinates (Å)		
	**X**	**Y**	**Z**		**X**	**Y**	**Z**
O	-1.92388	-2.18352	-1.35736	H	2.375373	0.82723	1.913461
H	0.622418	-1.24647	-1.41773	H	1.661658	3.204814	-0.97823
H	1.069092	-1.14407	1.588046	H	3.107785	3.689704	-0.08755
C	0.477971	-0.79929	0.737743	H	3.263288	2.952459	-1.69333
C	0.433973	0.738358	0.929548	H	4.510212	0.789323	1.06225
C	1.855605	1.419938	1.156994	H	4.947032	1.989121	-0.14262
C	2.697584	2.928279	-0.7552	H	5.778018	-0.38067	-0.57395
C	2.79174	1.525336	-0.12983	H	5.066916	0.475179	-1.91626
C	4.316605	1.109585	0.03898	H	4.341329	-2.19662	-0.93947
C	4.824682	-0.01093	-0.96491	H	3.741214	-1.3555	-2.36989
C	3.883331	-1.26254	-1.28774	H	2.75246	0.603934	-2.06652
C	2.267105	0.494518	-1.09286	H	1.208402	0.642954	-1.28911
O	3.049049	-0.99159	0.839367	H	3.377927	-1.89256	0.96149
C	2.577991	-0.94878	-0.54948	H	0.123269	-3.51457	-0.75699
C	1.138863	-1.50716	-0.48934	H	1.037966	-3.34181	0.77474
C	0.463425	-2.81421	0.007322	H	-1.83923	-1.64525	2.496745
C	-1.20652	-2.39876	2.017475	H	-0.38618	-2.63199	2.703552
C	-0.63956	-1.91798	0.675001	H	-1.80753	-3.30603	1.889173
C	-1.77801	-1.58717	-0.29178	H	-2.51342	0.023878	1.051127
C	-2.70567	-0.49956	0.121747	H	-3.92053	-0.70214	-1.56527
C	-3.75599	-0.15797	-0.63681	H	-5.0311	2.209673	1.113245
O	-4.38724	1.513713	0.921898	H	-5.45092	2.77242	-1.24276
C	-4.71165	1.989361	-1.44513	H	-4.94071	1.554289	-2.42246
C	-4.75557	0.924687	-0.33047	H	-3.7204	2.44834	-1.48949
C	-6.1627	0.302926	-0.22536	H	-6.44932	-0.1957	-1.15604
O	1.647986	2.746728	1.664864	H	-6.19322	-0.42772	0.587322
H	-0.12465	0.938996	1.854682	H	-6.90174	1.085246	-0.02006
H	-0.10555	1.264238	0.13311	H	1.233615	2.654865	2.533235

**2**-1*R*3*S*9*S*-conf. 5	**Coordinates (Å)**				**Coordinates (Å)**		
	**X**	**Y**	**Z**		**X**	**Y**	**Z**
O	-1.9307	-2.20783	-1.35478	H	2.351312	0.822458	1.919969
H	0.620453	-1.25221	-1.42054	H	1.637023	3.202529	-0.96252
H	1.065987	-1.14823	1.5855	H	3.087512	3.697457	-0.08531
C	0.474563	-0.80538	0.73495	H	3.237984	2.963233	-1.68558
C	0.42034	0.731113	0.927895	H	4.499283	0.795147	1.064457
C	1.829575	1.41254	1.162279	H	4.932349	1.996464	-0.14065
C	2.674793	2.929067	-0.74632	H	5.772769	-0.37092	-0.56916
C	2.778365	1.524088	-0.12886	H	5.061991	0.483503	-1.91271
C	4.304745	1.114668	0.040641	H	4.342155	-2.19093	-0.93833
C	4.81923	-0.00407	-0.96227	H	3.742967	-1.35137	-2.37012
C	3.882376	-1.25817	-1.28766	H	2.746763	0.605486	-2.06916
C	2.259827	0.494341	-1.09624	H	1.200903	0.639306	-1.29435
O	3.04438	-0.98728	0.837572	H	3.373546	-1.88778	0.962412
C	2.574879	-0.94786	-0.5519	H	0.127968	-3.52193	-0.75886
C	1.137679	-1.51112	-0.49202	H	1.04156	-3.34561	0.773091
C	0.465963	-2.82015	0.005047	H	-1.84262	-1.65422	2.491094
C	-1.20771	-2.40711	2.013672	H	-0.38784	-2.63746	2.701194
C	-0.63975	-1.92617	0.671469	H	-1.80642	-3.31586	1.885363
C	-1.77948	-1.60178	-0.29588	H	-2.50448	0.021785	1.035148
C	-2.70446	-0.50845	0.109989	H	-3.91818	-0.70159	-1.57869
C	-3.752	-0.15892	-0.64895	H	-4.69218	0.84074	1.624385
O	-4.49433	1.515812	0.958976	H	-5.29746	2.874581	-1.12842
C	-4.56162	2.091269	-1.33312	H	-4.70043	1.740727	-2.35925
C	-4.74015	0.940386	-0.32988	H	-3.55918	2.518038	-1.24209
C	-6.17403	0.380755	-0.39205	H	-6.40073	-0.02339	-1.38306
O	1.506654	2.698591	1.716673	H	-6.30867	-0.42409	0.338831
H	-0.13756	0.938019	1.849306	H	-6.88825	1.178195	-0.16794
H	-0.11593	1.250925	0.125016	H	2.330485	3.088344	2.038694

**2-**1*R*3*S*9*S*-conf. 7	**Coordinates (Å)**				**Coordinates (Å)**		
	**X**	**Y**	**Z**		**X**	**Y**	**Z**
O	-1.92633	-2.19794	-1.36434	H	2.363299	0.832306	1.912395
H	0.628258	-1.25279	-1.42136	H	1.663391	3.200558	-0.99105
H	1.059414	-1.13972	1.586244	H	3.103973	3.689167	-0.09338
C	0.472238	-0.79811	0.731993	H	3.269251	2.94705	-1.69595
C	0.427002	0.74022	0.918956	H	4.502213	0.793209	1.072766
C	1.847356	1.422845	1.151705	H	4.944865	1.989185	-0.13374
C	2.698193	2.925406	-0.76103	H	5.778978	-0.38168	-0.55296
C	2.789745	1.524514	-0.13074	H	5.074602	0.469481	-1.90177
C	4.313857	1.109984	0.047434	H	4.344965	-2.19945	-0.92041
C	4.827586	-0.01356	-0.9501	H	3.751826	-1.36308	-2.35629
C	3.888357	-1.26657	-1.27377	H	2.760806	0.596828	-2.06457
C	2.270498	0.490335	-1.09309	H	1.212781	0.637715	-1.29543
O	3.042154	-0.9894	0.8482	H	3.380944	-1.88638	0.972282
C	2.579026	-0.95116	-0.54338	H	0.12647	-3.51913	-0.75435
C	1.1398	-1.50998	-0.48922	H	1.033889	-3.33995	0.780922
C	0.462611	-2.81567	0.008876	H	-1.85529	-1.63857	2.481028
C	-1.21829	-2.39328	2.009204	H	-0.40175	-2.62173	2.70133
C	-0.64401	-1.91771	0.66788	H	-1.817	-3.30208	1.881315
C	-1.77866	-1.59231	-0.30474	H	-2.49718	0.041167	1.016478
C	-2.70261	-0.49664	0.096873	H	-3.9561	-0.73626	-1.55693
C	-3.76871	-0.16921	-0.64593	H	-3.53789	2.160809	0.589118
O	-4.35073	1.688161	0.820659	H	-5.62241	2.654141	-1.31683
C	-4.90297	1.86287	-1.546	H	-5.25463	1.314314	-2.42481
C	-4.75478	0.932152	-0.32744	H	-3.94355	2.326415	-1.8
C	-6.1092	0.309889	0.048445	H	-6.49117	-0.3155	-0.76269
O	1.636588	2.75088	1.654857	H	-6.00486	-0.30631	0.945453
H	-0.13615	0.94369	1.840771	H	-6.83472	1.103778	0.24923
H	-0.1079	1.263814	0.117767	H	1.224895	2.661159	2.524718

**2-**1*R*3*S*9*S*-conf. 8	Coordinates (Å)				Coordinates (Å)		
	**X**	**Y**	**Z**		**X**	**Y**	**Z**
O	1.499074	-1.30303	-0.82743	H	-3.3319	-1.17903	1.28043
H	-0.20406	0.955141	-0.73041	H	-3.4284	-1.81185	-2.47654
H	-1.18265	-0.33714	1.834589	H	-5.0511	-1.81619	-1.77855
C	-0.7003	-0.48152	0.865971	H	-4.51498	-0.44221	-2.76401
C	-1.47981	-1.66839	0.246714	H	-4.95472	0.37407	0.835301
C	-3.05952	-1.50244	0.272566	H	-5.71433	0.360624	-0.73723
C	-4.19619	-1.17957	-2.01868	H	-5.13877	2.672177	0.143509
C	-3.66498	-0.45737	-0.7678	H	-4.7093	2.322458	-1.50676
C	-4.76419	0.552885	-0.22484	H	-2.91982	3.358623	0.59236
C	-4.44329	2.090652	-0.47117	H	-2.58908	3.131825	-1.12558
C	-2.9515	2.627596	-0.22533	H	-2.81213	1.101906	-1.97184
C	-2.525	0.452701	-1.13965	H	-1.67416	-0.11532	-1.50674
O	-2.7094	0.985578	1.400224	H	-3.38721	1.627584	1.636248
C	-2.15064	1.364259	0.101883	H	1.234222	1.97491	0.95522
C	-0.67244	0.961781	0.257792	H	0.091443	1.555506	2.263174
C	0.457463	1.273498	1.271765	H	1.316463	-2.00461	2.475649
C	1.271314	-0.91299	2.561421	H	0.56965	-0.66492	3.363908
C	0.802722	-0.27731	1.244527	H	2.262954	-0.5613	2.863003
C	1.802738	-0.60018	0.134555	H	3.381534	0.605567	1.132898
C	3.172498	-0.03568	0.283979	H	3.893347	-0.95163	-1.44893
C	4.135473	-0.30416	-0.60776	H	6.588603	1.359328	0.621209
O	5.677518	1.035623	0.595857	H	7.555732	-0.62764	-0.45841
C	6.520812	-0.98739	-0.46811	H	6.407658	-1.66424	-1.32017
C	5.550525	0.20718	-0.56554	H	6.337649	-1.54877	0.451947
C	5.831622	1.035091	-1.8357	H	5.689442	0.437806	-2.74124
O	-3.66128	-2.78244	0.015233	H	5.164085	1.899859	-1.87807
H	-1.27651	-2.55171	0.869417	H	6.867321	1.3926	-1.82679
H	-1.14025	-1.9266	-0.76018	H	-3.40193	-3.36469	0.741874

**2**-1*R*3*S*9*S*-conf. 11	Coordinates (Å)				Coordinates (Å)		
	**X**	**Y**	**Z**		**X**	**Y**	**Z**
O	-1.92327	-2.18235	-1.3616	H	2.374155	0.828548	1.920718
H	0.622127	-1.24343	-1.41734	H	1.664234	3.19989	-0.99635
H	1.068987	-1.15315	1.589019	H	3.087001	3.690771	-0.05699
C	0.478634	-0.80503	0.739735	H	3.279287	2.977221	-1.66675
C	0.43329	0.731644	0.940903	H	4.513571	0.789811	1.062027
C	1.852017	1.4168	1.167573	H	4.948137	1.989996	-0.14266
C	2.69743	2.930853	-0.74026	H	5.779385	-0.37878	-0.5783
C	2.793249	1.523358	-0.12731	H	5.064511	0.477908	-1.91815
C	4.318275	1.110021	0.038982	H	4.340646	-2.19426	-0.93826
C	4.824935	-0.00923	-0.96664	H	3.740992	-1.35477	-2.36991
C	3.883189	-1.26052	-1.28788	H	2.747808	0.605971	-2.06843
C	2.265901	0.497071	-1.09278	H	1.20634	0.645985	-1.2852
O	3.048339	-0.9862	0.838773	H	3.372739	-1.88813	0.965336
C	2.577821	-0.94639	-0.54999	H	0.125087	-3.51365	-0.76626
C	1.139439	-1.50692	-0.49019	H	1.038829	-3.34707	0.766719
C	0.464613	-2.81645	0.001167	H	-1.84032	-1.65674	2.493092
C	-1.20723	-2.40868	2.011908	H	-0.38751	-2.64416	2.697912
C	-0.63915	-1.92305	0.671667	H	-1.80803	-3.31552	1.879777
C	-1.77693	-1.58858	-0.29465	H	-2.51015	0.020652	1.051654
C	-2.70337	-0.50084	0.121342	H	-3.91937	-0.7002	-1.56518
C	-3.75387	-0.15733	-0.63613	H	-5.02423	2.21111	1.116197
O	-4.38203	1.513761	0.924304	H	-5.44999	2.773269	-1.23795
C	-4.71109	1.990291	-1.44196	H	-4.94208	1.555505	-2.41896
C	-4.75278	0.925275	-0.32757	H	-3.71995	2.44936	-1.48824
C	-6.15969	0.303341	-0.22002	H	-6.44795	-0.19524	-1.15022
O	1.64766	2.686636	1.800607	H	-6.18857	-0.42737	0.592656
H	-0.11379	0.935767	1.869393	H	-6.8984	1.085563	-0.01325
H	-0.11311	1.253272	0.143885	H	1.115809	3.233195	1.205932

**2-**1*R*3*S*9*S*-conf. 13	Coordinates (Å)				Coordinates (Å)		
	**X**	**Y**	**Z**		**X**	**Y**	**Z**
O	1.496607	-1.31502	-0.80357	H	-3.34502	-1.16594	1.302547
H	-0.19976	0.949777	-0.73722	H	-3.40727	-1.8304	-2.46388
H	-1.18413	-0.29984	1.843949	H	-5.02703	-1.86983	-1.74228
C	-0.69874	-0.46453	0.880129	H	-4.53059	-0.50292	-2.75441
C	-1.48285	-1.66393	0.28938	H	-4.96535	0.400704	0.815639
C	-3.06312	-1.50055	0.304985	H	-5.7162	0.354535	-0.77012
C	-4.18819	-1.21604	-1.99707	H	-5.13308	2.669835	0.119127
C	-3.66869	-0.46412	-0.75996	H	-4.71752	2.320151	-1.53756
C	-4.77322	0.555234	-0.24585	H	-2.9345	3.360966	0.549824
C	-4.44544	2.087261	-0.50234	H	-2.58919	3.123004	-1.16329
C	-2.95832	2.62253	-0.2617	H	-2.80249	1.075119	-1.98243
C	-2.52418	0.436041	-1.13955	H	-1.67167	-0.13955	-1.49138
O	-2.7863	0.984668	1.347583	H	-2.69748	1.763122	1.913414
C	-2.15609	1.36247	0.079006	H	1.234709	1.992079	0.930073
C	-0.6726	0.966343	0.249304	H	0.100757	1.589969	2.249185
C	0.459831	1.29427	1.258473	H	1.319713	-1.96681	2.507304
C	1.274484	-0.87419	2.578256	H	0.573089	-0.61556	3.377627
C	0.804642	-0.25718	1.252987	H	2.266215	-0.51801	2.874157
C	1.802867	-0.59702	0.146377	H	3.384015	0.621995	1.12392
C	3.172972	-0.03116	0.284636	H	3.89023	-0.9723	-1.43631
C	4.134165	-0.31297	-0.60496	H	6.590124	1.366738	0.596005
O	5.679524	1.041183	0.577998	H	7.554314	-0.63757	-0.45865
C	6.519017	-0.99628	-0.46139	H	6.403283	-1.6841	-1.30427
C	5.54964	0.197893	-0.5723	H	6.337298	-1.54536	0.466354
C	5.828773	1.009088	-1.85362	H	5.684327	0.400344	-2.75111
O	-3.658	-2.80185	0.196473	H	5.161842	1.873794	-1.90578
H	-1.29491	-2.53435	0.930471	H	6.864778	1.365835	-1.85139
H	-1.13496	-1.94161	-0.71158	H	-3.35791	-3.19482	-0.63465

**2**-1*S*3*R*9*R*-conf. 1	Coordinates (Å)				Coordinates (Å)		
	**X**	**Y**	**Z**		**X**	**Y**	**Z**
O	2.28644	2.145418	-1.51625	H	-1.13831	-2.59166	0.295018
H	-1.58091	2.122497	1.122465	H	-3.39944	-2.55425	-2.32598
H	-0.16234	0.235932	-0.78962	H	-1.68991	-2.75041	-1.90582
C	-0.33833	0.601187	0.224295	H	-2.93432	-3.60587	-0.97925
C	-0.56734	-0.6489	1.088363	H	-4.91192	-2.10265	-0.56926
C	-1.7057	-1.72716	0.653465	H	-4.45431	-1.44584	0.979656
C	-2.70048	-2.66366	-1.48919	H	-5.43234	-0.08372	-1.54262
C	-2.81083	-1.4752	-0.51708	H	-5.91399	0.184753	0.110442
C	-4.33513	-1.30023	-0.09217	H	-4.58823	2.11883	0.209084
C	-5.03666	0.054804	-0.53108	H	-4.24828	1.891296	-1.50759
C	-4.19293	1.407049	-0.52651	H	-1.52203	-0.14531	-1.6615
C	-2.5145	-0.16999	-1.2215	H	-3.19615	-0.04837	-2.06817
O	-3.00096	0.408486	1.181132	H	-3.3737	1.144394	1.684198
C	-2.77359	0.959983	-0.18498	H	-0.46903	3.676374	-0.34736
C	-1.4395	1.684517	0.130604	H	-0.26001	2.441192	-1.61833
C	-0.38882	2.604169	-0.54565	H	0.127535	2.606533	2.21457
C	1.032975	2.400672	1.637721	H	1.577353	3.344337	1.523578
C	0.703828	1.802139	0.260741	H	1.655676	1.724784	2.231664
C	1.995849	1.523042	-0.49679	H	2.551251	-0.079	0.937026
C	2.873437	0.45357	0.049173	H	4.330867	0.696675	-1.427
C	4.036321	0.140906	-0.53837	H	5.061234	-2.2327	1.360869
O	4.437227	-1.55288	1.070988	H	7.064402	-1.03382	0.589283
C	6.347619	-0.266	0.277604	H	6.775323	0.267391	-0.57647
C	5.000065	-0.92239	-0.08509	H	6.213451	0.440788	1.100753
C	5.183745	-1.95858	-1.21207	H	5.568907	-1.49294	-2.1241
O	-2.31701	-2.19621	1.852419	H	5.898092	-2.72789	-0.89842
H	0.366272	-1.22377	1.140562	H	4.229773	-2.44107	-1.44089
H	-0.81239	-0.35644	2.111601	H	-2.79825	-1.41807	2.176368

**2**-1*S*3*R*9*R*-conf. 2	Coordinates (Å)				Coordinates (Å)		
	**X**	**Y**	**Z**		**X**	**Y**	**Z**
O	-1.81058	-1.72808	0.212324	H	2.800755	-2.65762	0.183093
H	1.117426	1.488181	-1.59612	H	4.213987	-1.12766	2.94954
H	0.40996	-0.57643	0.525778	H	2.915104	-2.20606	2.413222
C	0.59111	-0.3119	-0.51647	H	4.568491	-2.44892	1.825044
C	1.540457	-1.39185	-1.05801	H	5.647638	-0.23763	1.292968
C	2.94591	-1.68338	-0.29412	H	5.240375	-0.11629	-0.39824
C	3.825796	-1.6872	2.090979	H	4.955484	1.883753	1.859538
C	3.551718	-0.7526	0.898993	H	5.568156	2.125975	0.246228
C	4.864634	0.096624	0.600505	H	3.538587	3.107073	-0.40565
C	4.747841	1.666326	0.806616	H	3.018104	2.983234	1.276015
C	3.387943	2.40383	0.422966	H	1.594473	-0.09822	1.58798
C	2.546434	0.315116	1.269385	H	2.910894	0.876301	2.134692
O	3.158334	0.718564	-1.13997	H	3.225958	1.462438	-1.753
C	2.440531	1.267681	0.045331	H	-0.8609	2.455654	-0.62405
C	1.01273	1.17556	-0.55361	H	-0.6828	1.49143	0.870171
C	-0.45609	1.532878	-0.19981	H	-0.35963	0.859447	-2.9343
C	-1.14786	0.305392	-2.41799	H	-2.09138	0.831991	-2.59239
C	-0.85003	0.163724	-0.91638	H	-1.2234	-0.68138	-2.887
C	-1.98566	-0.60147	-0.24757	H	-3.37898	1.097154	-0.55794
C	-3.30222	0.085265	-0.17497	H	-4.26094	-1.53569	0.727642
C	-4.37469	-0.51973	0.353164	H	-6.59401	1.773732	0.028389
O	-5.70511	1.40055	-0.04968	H	-7.15916	0.530551	2.07665
C	-6.14979	0.117112	1.97278	H	-6.14791	-0.88266	2.417159
C	-5.75221	0.072304	0.483519	H	-5.45451	0.750016	2.530655
C	-6.75156	-0.78229	-0.32188	H	-6.76985	-1.81681	0.03354
O	3.925851	-1.94153	-1.29754	H	-7.76183	-0.37136	-0.21712
H	1.00518	-2.34949	-1.06494	H	-6.48197	-0.78033	-1.38137
H	1.809287	-1.17424	-2.09436	H	4.029384	-1.08061	-1.73347

**2**-1*S*3*R*9*R*-conf. 3	Coordinates (Å)				Coordinates (Å)		
	**X**	**Y**	**Z**		**X**	**Y**	**Z**
O	2.277163	2.134853	-1.53525	H	-1.13987	-2.59244	0.280402
H	-1.57247	2.118655	1.128136	H	-3.41353	-2.54278	-2.3293
H	-0.16541	0.237102	-0.7971	H	-1.7023	-2.74261	-1.91809
C	-0.33629	0.598948	0.218917	H	-2.94336	-3.59987	-0.98864
C	-0.56364	-0.65365	1.079652	H	-4.91715	-2.09542	-0.56417
C	-1.70487	-1.729	0.645179	H	-4.45158	-1.44498	0.985057
C	-2.71075	-2.65612	-1.49627	H	-5.43877	-0.07214	-1.52785
C	-2.81499	-1.47108	-0.51931	H	-5.91294	0.190775	0.128288
C	-4.33706	-1.29559	-0.08675	H	-4.58384	2.12251	0.22869
C	-5.03858	0.062024	-0.5175	H	-4.25211	1.901232	-1.49039
C	-4.19294	1.413074	-0.51149	H	-1.52939	-0.13874	-1.66459
C	-2.5199	-0.16367	-1.22015	H	-3.20514	-0.03789	-2.06329
O	-2.99436	0.406223	1.186815	H	-3.36451	1.140368	1.694367
C	-2.77271	0.962722	-0.1782	H	-0.46491	3.676324	-0.34125
C	-1.4362	1.684243	0.134012	H	-0.26325	2.445226	-1.61742
C	-0.38721	2.604738	-0.54367	H	0.141046	2.598569	2.213261
C	1.04389	2.391985	1.632676	H	1.590333	3.334532	1.519429
C	0.707884	1.798614	0.25522	H	1.66672	1.712891	2.222804
C	1.994899	1.518592	-0.51018	H	2.56114	-0.07625	0.926287
C	2.879643	0.453219	0.033868	H	4.343454	0.704514	-1.43429
C	4.046477	0.144756	-0.5483	H	3.715647	-2.17509	0.693183
O	4.473005	-1.6691	1.021883	H	6.997083	-1.02999	0.767631
C	6.285184	-0.25943	0.457237	H	6.754616	0.361477	-0.3103
C	5.007225	-0.92431	-0.07903	H	6.049353	0.369462	1.319849
C	5.335832	-1.87341	-1.24705	H	5.78355	-1.33229	-2.08586
O	-2.31102	-2.20258	1.844842	H	6.039881	-2.63925	-0.90931
H	0.369491	-1.22997	1.126592	H	4.429225	-2.36902	-1.61088
H	-0.80427	-0.36435	2.104792	H	-2.79036	-1.42562	2.174405

**2-**1*S*3*R*9*R*-conf. 5	Coordinates (Å)				Coordinates (Å)		
	**X**	**Y**	**Z**		**X**	**Y**	**Z**
O	2.284536	2.153404	-1.52295	H	-1.12291	-2.58661	0.301933
H	-1.58133	2.127659	1.117712	H	-3.38331	-2.56345	-2.31983
H	-0.15629	0.240541	-0.78882	H	-1.67334	-2.7531	-1.89849
C	-0.33359	0.608051	0.223996	H	-2.91539	-3.61013	-0.97023
C	-0.55927	-0.64027	1.091477	H	-4.898	-2.11207	-0.56505
C	-1.69359	-1.72341	0.658217	H	-4.44332	-1.44965	0.982353
C	-2.68435	-2.66851	-1.4825	H	-5.42399	-0.09749	-1.54428
C	-2.79894	-1.47792	-0.51346	H	-5.90775	0.173907	0.107682
C	-4.324	-1.30657	-0.0898	H	-4.5882	2.112492	0.202081
C	-5.02954	0.045015	-0.53283	H	-4.24637	1.881412	-1.51372
C	-4.19014	1.399971	-0.53132	H	-1.51362	-0.14688	-1.66056
C	-2.50627	-0.17358	-1.22104	H	-3.18772	-0.0563	-2.06847
O	-2.99609	0.409791	1.179872	H	-3.37386	1.145085	1.680072
C	-2.76964	0.958287	-0.18761	H	-0.47347	3.681389	-0.35565
C	-1.43803	1.687773	0.126983	H	-0.26048	2.443679	-1.62349
C	-0.39006	2.608955	-0.55128	H	0.125936	2.621324	2.208076
C	1.032273	2.416009	1.632503	H	1.574269	3.360709	1.515816
C	0.704988	1.812452	0.257292	H	1.656027	1.743637	2.229343
C	1.997082	1.535156	-0.50051	H	2.565625	-0.04777	0.947802
C	2.880666	0.47111	0.04798	H	4.309014	0.666703	-1.46244
C	4.029548	0.13451	-0.55401	H	4.669781	-0.80828	1.84242
O	4.581086	-1.49835	1.169003	H	7.099092	-1.12197	0.369662
C	6.404947	-0.34418	0.038811	H	6.754678	0.052277	-0.91899
C	4.988142	-0.93624	-0.0842	H	6.422398	0.47269	0.768488
C	4.972545	-2.10524	-1.08222	H	5.245086	-1.76672	-2.08524
O	-2.30392	-2.19197	1.857842	H	5.688535	-2.86877	-0.76399
H	0.376022	-1.21217	1.146279	H	3.975921	-2.55284	-1.12289
H	-0.80637	-0.34559	2.113565	H	-2.78836	-1.41497	2.179724

2-1*S*3*R*9*R*-conf. 7	Coordinates (Å)				Coordinates (Å)		
	X	Y	Z		X	Y	Z
O	-1.81283	-1.75508	0.229982	H	2.810738	-2.64577	0.285844
H	1.113027	1.418506	-1.65915	H	4.193462	-1.00376	3.002992
H	0.403397	-0.569	0.534251	H	2.905837	-2.11052	2.498184
C	0.590128	-0.34295	-0.51607	H	4.565141	-2.36346	1.931177
C	1.550174	-1.43622	-1.00976	H	5.633086	-0.16601	1.324504
C	2.952493	-1.68896	-0.22633	H	5.237513	-0.11125	-0.37299
C	3.815341	-1.59767	2.163236	H	4.922768	1.970373	1.805663
C	3.543653	-0.71017	0.93524	H	5.545431	2.155965	0.188684
C	4.853008	0.136417	0.614348	H	3.513396	3.097901	-0.51366
C	4.724203	1.71201	0.760277	H	2.982241	3.033413	1.167916
C	3.362101	2.425067	0.339821	H	1.577281	-0.04394	1.58602
C	2.528723	0.363696	1.25858	H	2.88334	0.959389	2.104679
O	3.154855	0.680468	-1.16017	H	3.229665	1.403513	-1.79678
C	2.425229	1.268877	-0.00124	H	-0.878	2.40966	-0.73732
C	1.002575	1.144887	-0.60632	H	-0.70472	1.503128	0.793079
C	-0.47073	1.505869	-0.27613	H	-0.35273	0.732487	-2.98257
C	-1.14018	0.191877	-2.45114	H	-2.08643	0.704685	-2.65068
C	-0.85116	0.108535	-0.94341	H	-1.2054	-0.81221	-2.88342
C	-1.98642	-0.63956	-0.2558	H	-3.3774	1.049237	-0.61629
C	-3.30224	0.050797	-0.19627	H	-4.25548	-1.51739	0.799886
C	-4.37039	-0.52022	0.37675	H	-5.72937	1.196709	-1.12496
O	-5.81053	1.366593	-0.17513	H	-7.03428	0.833574	2.075474
C	-6.02976	0.411511	1.97704	H	-5.97107	-0.49609	2.583506
C	-5.74218	0.104074	0.49861	H	-5.3051	1.135173	2.359693
C	-6.80138	-0.85712	-0.07315	H	-6.79663	-1.81428	0.456648
O	3.940914	-1.97735	-1.21301	H	-7.79387	-0.40811	0.024781
H	1.021316	-2.39704	-0.9834	H	-6.61192	-1.05754	-1.13324
H	1.824491	-1.25649	-2.0519	H	4.041846	-1.13244	-1.67977

**2**-1*S*3*R*9*R*-conf. 8	Coordinates (Å)				Coordinates (Å)		
	**X**	**Y**	**Z**		**X**	**Y**	**Z**
O	-1.80769	-1.73812	0.236895	H	2.807893	-2.6544	0.209316
H	1.109106	1.467495	-1.60951	H	4.221504	-1.09194	2.957286
H	0.411736	-0.57733	0.53466	H	2.925397	-2.18012	2.43412
C	0.590097	-0.32297	-0.51059	H	4.578624	-2.42326	1.845598
C	1.541911	-1.40535	-1.04286	H	5.649533	-0.21434	1.288973
C	2.949474	-1.68478	-0.27823	H	5.238774	-0.11149	-0.40264
C	3.833768	-1.66145	2.1051	H	4.951644	1.910498	1.835246
C	3.554556	-0.73994	0.904158	H	5.560648	2.138232	0.218428
C	4.864211	0.110353	0.594605	H	3.526658	3.106354	-0.43957
C	4.742828	1.681709	0.784972	H	3.00968	2.99772	1.244198
C	3.379888	2.410968	0.396353	H	1.59646	-0.08488	1.589929
C	2.546526	0.328263	1.265442	H	2.910655	0.899379	2.124369
O	3.152948	0.709004	-1.14898	H	3.219342	1.447346	-1.7688
C	2.435573	1.267971	0.031914	H	-0.87028	2.439196	-0.64359
C	1.007146	1.165313	-0.56367	H	-0.68646	1.490605	0.86006
C	-0.46197	1.521952	-0.21077	H	-0.369	0.821021	-2.9385
C	-1.15455	0.26985	-2.41514	H	-2.09999	0.791864	-2.59315
C	-0.85315	0.144424	-0.91281	H	-1.22801	-0.72188	-2.87383
C	-1.98511	-0.61675	-0.23381	H	-3.37803	1.078577	-0.55425
C	-3.30316	0.068328	-0.16337	H	-4.27284	-1.55046	0.730961
C	-4.38078	-0.53203	0.359838	H	-5.23496	1.954384	0.711446
O	-5.76123	1.454892	0.070206	H	-7.24903	0.402901	2.019884
C	-6.24945	-0.03309	1.936294	H	-6.29523	-1.07737	2.259123
C	-5.7571	0.082494	0.481259	H	-5.57777	0.501013	2.61712
C	-6.72085	-0.63546	-0.47715	H	-6.76465	-1.7057	-0.25879
O	3.928479	-1.95056	-1.28051	H	-7.72525	-0.21522	-0.37016
H	1.009726	-2.36472	-1.03937	H	-6.39238	-0.50283	-1.51143
H	1.808368	-1.19721	-2.08173	H	4.028886	-1.09401	-1.72566

**2**-1*S*3*R*9*R*-conf. 18	Coordinates (Å)				Coordinates (Å)		
	**X**	**Y**	**Z**		**X**	**Y**	**Z**
O	-1.81317	-1.73957	0.094896	H	2.804198	-2.66112	0.042543
H	1.124748	1.571137	-1.52006	H	4.195319	-1.28131	2.897947
H	0.408247	-0.60421	0.485099	H	2.902434	-2.33109	2.294676
C	0.594386	-0.28399	-0.54052	H	4.560387	-2.53922	1.706196
C	1.548247	-1.33176	-1.13458	H	5.639092	-0.30074	1.301782
C	2.949983	-1.66198	-0.37977	H	5.243782	-0.08993	-0.38338
C	3.814379	-1.79412	2.007545	H	4.941343	1.786849	1.97538
C	3.54682	-0.79658	0.866079	H	5.562951	2.115364	0.380911
C	4.860506	0.069065	0.622564	H	3.535917	3.128247	-0.22897
C	4.740125	1.625527	0.911209	H	3.005466	2.913128	1.440243
C	3.381365	2.381003	0.559529	H	1.584729	-0.18154	1.57809
C	2.538115	0.248813	1.287578	H	2.897017	0.762913	2.18387
O	3.163473	0.781712	-1.09312	H	3.24168	1.559303	-1.66133
C	2.438149	1.265625	0.115579	H	-0.86036	2.48433	-0.51028
C	1.014084	1.203941	-0.49624	H	-0.68958	1.443403	0.932602
C	-0.45704	1.540949	-0.13247	H	-0.34539	1.013756	-2.89762
C	-1.13513	0.431002	-2.41654	H	-2.07884	0.964084	-2.56942
C	-0.84512	0.210931	-0.92268	H	-1.20504	-0.52974	-2.93743
C	-1.98271	-0.58919	-0.30106	H	-3.35732	1.126364	-0.52837
C	-3.30101	0.096106	-0.19019	H	-4.25064	-1.56502	0.637002
C	-4.3722	-0.53078	0.31641	H	-6.50297	-0.97518	-1.03294
O	-6.687	-0.93738	-0.08305	H	-7.03124	1.692668	-0.00907
C	-5.98722	1.395592	-0.1381	H	-5.35383	2.156581	0.327061
C	-5.77137	0.017656	0.495805	H	-5.7649	1.376147	-1.21019
C	-6.11672	0.039418	1.991423	H	-5.47807	0.746323	2.527245
O	3.935953	-1.863	-1.39024	H	-7.16108	0.339744	2.120095
H	1.014886	-2.28844	-1.19669	H	-5.98609	-0.95373	2.430451
H	1.82235	-1.05737	-2.15592	H	4.04081	-0.97907	-1.77703

**2**-1*S*3*R*9*S*-conf. 1	Coordinates (Å)				Coordinates (Å)		
	**X**	**Y**	**Z**		**X**	**Y**	**Z**
O	-2.44761	-2.59381	-0.33997	H	1.830749	2.84031	0.545966
H	1.128695	-1.54556	-2.0478	H	4.035245	3.47225	-0.24769
H	-0.50202	-0.00424	-1.33112	H	4.539827	2.545664	-1.67027
C	0.169106	-0.22486	-0.49341	H	2.887195	3.172638	-1.56308
C	0.579096	1.211921	0.047085	H	5.352081	1.454875	0.385439
C	1.971016	1.754733	0.607225	H	4.293089	0.611618	1.486657
C	3.705723	2.729215	-0.98342	H	5.693901	-1.05844	0.48765
C	3.263944	1.429367	-0.28884	H	5.609592	-0.33382	-1.09541
C	4.504107	0.760528	0.428767	H	4.109054	-1.96143	-1.70595
C	5.003766	-0.58647	-0.219	H	3.959616	-2.55452	-0.05632
C	3.917123	-1.64813	-0.6746	H	3.577959	0.240913	-2.0662
C	2.809934	0.402415	-1.30316	H	1.925427	0.735394	-1.84649
O	2.436585	-0.78822	0.909011	H	2.736139	-1.62098	1.293814
C	2.56799	-0.93864	-0.54889	H	-0.13901	-3.15825	-0.70833
C	1.170498	-1.36835	-0.96783	H	0.972757	-2.92003	0.648942
C	0.390301	-2.40607	-0.12054	H	-0.88222	-1.99745	2.451887
C	-0.5299	-1.09111	1.94653	H	-1.17492	-0.26419	2.260454
C	-0.55548	-1.29908	0.424446	H	0.484043	-0.87685	2.283436
C	-2.01657	-1.49837	0.011726	H	-2.45805	0.652138	0.362437
C	-2.89474	-0.29757	0.073795	H	-4.6081	-1.34388	-0.5017
C	-4.2	-0.37557	-0.21667	H	-5.10436	2.672871	0.210459
O	-4.46898	1.943817	0.194182	H	-5.85486	0.364067	1.842345
C	-6.28812	0.459746	0.843194	H	-7.02049	1.274678	0.855941
C	-5.18372	0.763377	-0.18913	H	-6.81834	-0.46601	0.600719
C	-5.78872	0.94813	-1.59533	H	-6.52537	1.759066	-1.5809
O	2.129468	1.535249	2.001713	H	-5.00444	1.201468	-2.31364
H	0.330016	1.903895	-0.76607	H	-6.29478	0.03951	-1.93496
H	-0.12728	1.458773	0.84856	H	2.280978	0.577932	2.060035

**2**-1*S*3*R*9*S*-conf. 2	Coordinates (Å)				Coordinates (Å)		
	**X**	**Y**	**Z**		**X**	**Y**	**Z**
O	-2.4475	-2.59371	-0.34056	H	1.830622	2.840406	0.545489
H	1.128968	-1.54593	-2.04761	H	4.03543	3.472174	-0.24821
H	-0.50186	-0.00457	-1.33131	H	4.539776	2.545319	-1.67069
C	0.169212	-0.225	-0.4935	H	2.887245	3.172558	-1.56348
C	0.579098	1.211871	0.046788	H	5.351963	1.455069	0.385584
C	1.970945	1.754839	0.606913	H	4.292808	0.611923	1.486746
C	3.705748	2.729082	-0.98381	H	5.693955	-1.05816	0.488146
C	3.263907	1.429357	-0.28899	H	5.609674	-0.33384	-1.09504
C	4.504017	0.760689	0.428875	H	4.109292	-1.96171	-1.70535
C	5.003809	-0.58637	-0.21861	H	3.959764	-2.55442	-0.05557
C	3.91728	-1.64818	-0.67408	H	3.578086	0.240499	-2.0661
C	2.810017	0.402179	-1.30316	H	1.925524	0.734996	-1.8466
O	2.436465	-0.78801	0.909182	H	2.735503	-1.62085	1.294198
C	2.568053	-0.93875	-0.5486	H	-0.13888	-3.15842	-0.70815
C	1.170646	-1.36856	-0.96766	H	0.972675	-2.92007	0.649271
C	0.390336	-2.40619	-0.12036	H	-0.88312	-1.99694	2.45195
C	-0.53005	-1.09096	1.946457	H	-1.17448	-0.26348	2.2601
C	-0.55551	-1.29914	0.424417	H	0.484001	-0.87742	2.283501
C	-2.01652	-1.49835	0.011478	H	-2.45805	0.652132	0.362319
C	-2.89472	-0.29757	0.073634	H	-4.60809	-1.34387	-0.50187
C	-4.19999	-0.37558	-0.21678	H	-5.10426	2.672887	0.210392
O	-4.46887	1.943847	0.193933	H	-6.818	-0.46602	0.601566
C	-6.28775	0.459811	0.843708	H	-5.85416	0.36432	1.842732
C	-5.18372	0.763353	-0.18902	H	-7.02018	1.274694	0.85656
C	-5.78924	0.947958	-1.59503	H	-6.52601	1.758778	-1.58039
O	2.129337	1.535588	2.001463	H	-5.00524	1.201371	-2.31362
H	0.33003	1.903669	-0.76651	H	-6.29528	0.039247	-1.93443
H	-0.12734	1.458854	0.848158	H	2.280711	0.578252	2.059975

**2-**1*S*3*R*9*S*-conf. 3	**Coordinates (Å)**				**Coordinates (Å)**		
	**X**	**Y**	**Z**		**X**	**Y**	**Z**
O	-2.44384	-2.60366	-0.36315	H	1.81242	2.834747	0.565871
H	1.120716	-1.53667	-2.05387	H	4.011459	3.481249	-0.23177
H	-0.51418	-0.00749	-1.32115	H	4.514798	2.565584	-1.66185
C	0.160757	-0.2309	-0.48727	H	2.859918	3.184787	-1.54482
C	0.566244	1.20411	0.061206	H	4.287677	0.611056	1.48401
C	1.957687	1.749436	0.619867	H	5.339151	1.465614	0.384378
C	3.682447	2.741347	-0.97086	H	5.599189	-0.31277	-1.10841
C	3.248818	1.435328	-0.28279	H	5.692092	-1.04676	0.46985
C	4.494315	0.76739	0.426326	H	3.962257	-2.54689	-0.07744
C	4.997517	-0.57342	-0.2315	H	4.10357	-1.943	-1.72388
C	3.913842	-1.6369	-0.68997	H	3.561813	0.259288	-2.0686
C	2.795757	0.412794	-1.30196	H	1.908016	0.745374	-1.84024
O	2.43452	-0.79367	0.903941	H	2.742293	-1.62617	1.282742
C	2.562149	-0.93404	-0.55535	H	-0.13591	-3.16402	-0.72211
C	1.16523	-1.36697	-0.97283	H	0.979205	-2.93062	0.633533
C	0.392115	-2.41396	-0.13055	H	-0.87259	-2.03064	2.448915
C	-0.52615	-1.11896	1.949151	H	-1.17258	-0.29706	2.27305
C	-0.55644	-1.31508	0.425709	H	0.488384	-0.90336	2.283357
C	-2.01691	-1.51471	0.011652	H	-2.46989	0.620003	0.429297
C	-2.90107	-0.31889	0.096141	H	-4.59573	-1.33097	-0.58542
C	-4.19624	-0.37792	-0.2409	H	-4.39664	1.745898	1.323583
O	-4.57744	1.943975	0.393082	H	-6.9186	-0.49504	0.150762
C	-6.42243	0.366827	0.606775	H	-6.15785	0.097954	1.635139
C	-5.17046	0.777718	-0.19102	H	-7.12848	1.201658	0.635675
C	-5.5503	1.185376	-1.62362	H	-6.27867	2.001185	-1.59226
O	2.1219	1.521846	2.012381	H	-4.66428	1.525565	-2.16626
H	0.311123	1.900046	-0.74664	H	-5.99077	0.344396	-2.16559
H	-0.13836	1.44269	0.866747	H	2.278013	0.564888	2.064083

**2-**1*S*3*R*9*S*-conf. 4	**Coordinates (Å)**				**Coordinates (Å)**		
	**X**	**Y**	**Z**		**X**	**Y**	**Z**
O	-2.44016	-2.59706	-0.37221	H	1.81746	2.840993	0.536467
H	1.131603	-1.55481	-2.04345	H	4.021327	3.476271	-0.25693
H	-0.50438	-0.01614	-1.33359	H	4.530083	2.545796	-1.67547
C	0.166113	-0.2325	-0.49422	H	2.875623	3.1686	-1.5725
C	0.571007	1.207065	0.042604	H	5.342945	1.464696	0.385319
C	1.960922	1.756073	0.60201	H	4.284751	0.622932	1.488437
C	3.694716	2.729603	-0.99024	H	5.691004	-1.04744	0.498042
C	3.255778	1.431085	-0.29142	H	5.607641	-0.32907	-1.08793
C	4.496782	0.768266	0.430249	H	4.112101	-1.96246	-1.69505
C	5.000951	-0.5799	-0.21161	H	3.961796	-2.5502	-0.04362
C	3.917827	-1.64608	-0.66508	H	3.57553	0.237033	-2.06398
C	2.806018	0.39923	-1.3026	H	1.921409	0.727917	-1.84842
O	2.432146	-0.78471	0.913181	H	2.735334	-1.61442	1.301724
C	2.566676	-0.9398	-0.54394	H	-0.13363	-3.16729	-0.70221
C	1.171143	-1.37471	-0.9639	H	0.975173	-2.92235	0.656627
C	0.39252	-2.41233	-0.11526	H	-0.88711	-2.00209	2.453684
C	-0.53489	-1.09634	1.947188	H	-1.18039	-0.2694	2.259808
C	-0.55733	-1.30697	0.425704	H	0.478475	-0.88105	2.28515
C	-2.01554	-1.50768	0.003919	H	-2.46951	0.629752	0.406561
C	-2.9005	-0.31193	0.081161	H	-4.60559	-1.34336	-0.5418
C	-4.20163	-0.38099	-0.22984	H	-3.94665	2.235486	-0.59319
O	-4.53014	2.005152	0.144319	H	-6.73178	-0.4114	0.789319
C	-6.19879	0.531135	0.939723	H	-5.69089	0.494902	1.907196
C	-5.1797	0.771481	-0.18556	H	-6.92786	1.346707	0.955387
C	-5.89131	0.898232	-1.54596	H	-6.60552	1.725854	-1.50989
O	2.118289	1.542251	1.997369	H	-5.16873	1.095293	-2.34544
H	0.320294	1.895812	-0.77281	H	-6.43145	-0.01883	-1.79907
H	-0.13652	1.453993	0.843094	H	2.271734	0.585507	2.059703

**2-**1*S*3*R*9*S*-conf. 6	Coordinates (Å)				Coordinates (Å)		
	**X**	**Y**	**Z**		**X**	**Y**	**Z**
O	-2.06887	-1.65919	1.229755	H	3.48151	-2.59786	-0.17066
H	0.818064	1.233426	1.992363	H	5.74832	-1.90233	0.347537
H	0.139613	-1.01965	1.687997	H	5.807005	-0.61586	1.563507
C	0.560924	-0.63526	0.756073	H	4.709389	-1.98849	1.779694
C	1.614629	-1.74676	0.338072	H	5.790615	0.355329	-0.70241
C	3.036139	-1.61604	-0.37035	H	4.371406	0.355591	-1.7176
C	5.138366	-1.30593	1.036214	H	4.784478	2.645471	-1.13765
C	4.041748	-0.54116	0.274666	H	5.203457	2.263113	0.510535
C	4.705875	0.51781	-0.69395	H	3.13084	3.009123	1.160113
C	4.48993	2.02506	-0.28515	H	2.575688	3.156966	-0.50269
C	3.049876	2.46991	0.210763	H	3.834005	0.937067	1.836875
C	3.202579	0.285852	1.224166	H	2.656986	-0.3396	1.930614
O	2.10752	0.732875	-1.0264	H	1.937321	1.527886	-1.54621
C	2.250782	1.17559	0.369794	H	-1.17613	1.762796	0.697984
C	0.867674	0.91337	0.946092	H	-0.22045	1.855279	-0.78773
C	-0.39518	1.26495	0.115914	H	-1.43301	-0.2023	-2.19685
C	-0.63292	-0.68799	-1.62865	H	-0.76552	-1.7709	-1.72645
C	-0.66441	-0.25742	-0.15291	H	0.321637	-0.41937	-2.07936
C	-2.00133	-0.73335	0.424875	H	-3.10378	0.791645	-0.75801
C	-3.22048	-0.02838	-0.05811	H	-4.52294	-1.21281	1.064818
C	-4.44149	-0.38711	0.35988	H	-6.27692	1.702341	-1.23966
O	-5.43837	1.296479	-0.9796	H	-5.8102	1.610186	1.646446
C	-6.43666	0.836681	1.194277	H	-7.39522	1.284679	0.90984
C	-5.74212	0.249379	-0.05084	H	-6.63483	0.063927	1.942946
C	-6.63824	-0.80827	-0.72599	H	-7.59581	-0.35819	-1.01087
O	2.952252	-1.59027	-1.78888	H	-6.15327	-1.19584	-1.6259
H	1.827858	-2.29977	1.260831	H	-6.84627	-1.64454	-0.0519
H	1.074793	-2.46236	-0.29332	H	2.586932	-0.70974	-1.97294

**2-**1*S*3*R*9*S*-conf. 7	Coordinates (Å)				Coordinates (Å)		
	**X**	**Y**	**Z**		**X**	**Y**	**Z**
O	-2.44403	-2.60383	-0.36241	H	1.812878	2.834613	0.566501
H	1.120301	-1.53648	-2.05377	H	4.011707	3.481196	-0.23164
H	-0.51406	-0.00702	-1.32089	H	4.514692	2.565718	-1.66195
C	0.16076	-0.23071	-0.48698	H	2.859859	3.184939	-1.54447
C	0.566396	1.204051	0.061996	H	5.33942	1.465405	0.384032
C	1.958085	1.749298	0.620284	H	4.28816	0.610647	1.483713
C	3.682499	2.741392	-0.97075	H	5.692376	-1.04697	0.46879
C	3.248962	1.43528	-0.28274	H	5.598938	-0.31265	-1.10928
C	4.494577	0.767183	0.426007	H	4.103229	-1.94291	-1.72449
C	4.997554	-0.57348	-0.23222	H	3.962281	-2.54694	-0.07809
C	3.913749	-1.63688	-0.69052	H	3.56148	0.259558	-2.06881
C	2.79561	0.412927	-1.30195	H	1.90776	0.745667	-1.83996
O	2.434729	-0.79383	0.903824	H	2.743362	-1.62607	1.282507
C	2.562092	-0.93401	-0.55552	H	-0.13582	-3.16408	-0.72177
C	1.16507	-1.36683	-0.97273	H	0.97923	-2.93021	0.633864
C	0.392067	-2.41381	-0.13033	H	-0.87327	-2.0307	2.448747
C	-0.52664	-1.11897	1.949211	H	-1.1731	-0.29706	2.27305
C	-0.5566	-1.31494	0.425732	H	0.487854	-0.90352	2.28365
C	-2.01704	-1.51467	0.011665	H	-2.46958	0.620468	0.427414
C	-2.90106	-0.31872	0.095476	H	-4.5963	-1.33136	-0.58386
C	-4.19647	-0.37799	-0.24059	H	-4.39509	1.748365	1.321046
O	-4.57674	1.944883	0.390385	H	-6.91823	-0.49447	0.154712
C	-6.42154	0.368111	0.608805	H	-6.15574	0.100814	1.637271
C	-5.17056	0.777777	-0.19119	H	-7.12758	1.202966	0.63726
C	-5.55225	1.183308	-1.6239	H	-6.28052	1.999211	-1.59282
O	2.122619	1.521483	2.012716	H	-4.66692	1.522628	-2.16821
H	0.31086	1.900358	-0.74539	H	-5.99346	0.341543	-2.16405
H	-0.13784	1.442219	0.867976	H	2.278871	0.564528	2.064152

**2-**1*S*3*R*9*S*-conf. 10	Coordinates (Å)				Coordinates (Å)		
	**X**	**Y**	**Z**		**X**	**Y**	**Z**
O	-2.4403	-2.59715	-0.37157	H	1.817508	2.840865	0.536639
H	1.131717	-1.55484	-2.04353	H	4.021572	3.476182	-0.25665
H	-0.50421	-0.01622	-1.33383	H	4.530196	2.545728	-1.67525
C	0.166226	-0.23248	-0.49437	H	2.875797	3.168694	-1.57221
C	0.57108	1.20705	0.042452	H	5.342831	1.464717	0.385956
C	1.960901	1.755938	0.602075	H	4.284565	0.622394	1.488548
C	3.694859	2.729582	-0.99	H	5.691651	-1.04716	0.497278
C	3.255789	1.431032	-0.29125	H	5.607272	-0.32841	-1.08843
C	4.496748	0.768163	0.430451	H	4.112254	-1.96261	-1.69497
C	5.001066	-0.57966	-0.21188	H	3.962088	-2.54997	-0.04338
C	3.918025	-1.64599	-0.66507	H	3.575495	0.237155	-2.064
C	2.806045	0.399276	-1.30256	H	1.921374	0.727916	-1.84828
O	2.432132	-0.78473	0.913123	H	2.736156	-1.61404	1.301827
C	2.566732	-0.93983	-0.5439	H	-0.13357	-3.16732	-0.70239
C	1.171242	-1.37473	-0.96398	H	0.975031	-2.92223	0.656623
C	0.392471	-2.41232	-0.11541	H	-0.88713	-2.00194	2.45347
C	-0.53488	-1.09622	1.946979	H	-1.18033	-0.2692	2.259502
C	-0.55738	-1.30691	0.425424	H	0.478537	-0.88098	2.284824
C	-2.01561	-1.5076	0.004073	H	-2.46952	0.629996	0.405961
C	-2.90054	-0.31183	0.081027	H	-4.60575	-1.3435	-0.54121
C	-4.20174	-0.38102	-0.22966	H	-3.94705	2.235583	-0.59386
O	-4.53015	2.005209	0.14394	H	-6.73162	-0.41125	0.789859
C	-6.19875	0.531411	0.939871	H	-5.69073	0.495595	1.907296
C	-5.17978	0.771481	-0.1856	H	-6.92793	1.346886	0.955312
C	-5.89153	0.8979	-1.54595	H	-6.6057	1.725554	-1.51002
O	2.11813	1.541969	1.99745	H	-5.16906	1.094692	-2.34559
H	0.3206	1.895781	-0.77306	H	-6.43175	-0.01921	-1.79873
H	-0.13663	1.454063	0.842737	H	2.271897	0.585248	2.0596

**2**-1*S*3*R*9*S*-conf. 11	**Coordinates (Å)**				**Coordinates (Å)**		
	**X**	**Y**	**Z**		**X**	**Y**	**Z**
O	2.068779	-1.65878	-1.22996	H	-3.48118	-2.598	0.170839
H	-0.81845	1.233534	-1.99258	H	-5.74817	-1.90274	-0.34723
H	-0.1396	-1.01946	-1.68808	H	-5.80708	-0.61625	-1.56317
C	-0.56095	-0.63504	-0.75618	H	-4.70937	-1.98878	-1.77948
C	-1.61445	-1.74667	-0.33808	H	-5.79058	0.354793	0.702908
C	-3.03592	-1.61611	0.370448	H	-4.37112	0.35545	1.717741
C	-5.13834	-1.30626	-1.03595	H	-4.78518	2.645277	1.137352
C	-4.04172	-0.54139	-0.27452	H	-5.20373	2.262376	-0.51079
C	-4.70588	0.517527	0.694171	H	-3.13121	3.009102	-1.15995
C	-4.49028	2.024697	0.285089	H	-2.57618	3.156782	0.502911
C	-3.05027	2.469774	-0.21066	H	-3.83421	0.936823	-1.83682
C	-3.20272	0.285692	-1.22411	H	-2.65709	-0.33973	-1.93054
O	-2.10743	0.732853	1.026305	H	-1.93785	1.527899	1.546262
C	-2.25095	1.175559	-0.36978	H	1.175864	1.763165	-0.69847
C	-0.86788	0.913523	-0.94629	H	0.220276	1.855752	0.787313
C	0.395015	1.265325	-0.11627	H	1.433567	-0.20198	2.196431
C	0.633133	-0.68742	1.628503	H	0.765346	-1.77037	1.726326
C	0.664397	-0.25699	0.152721	H	-0.3212	-0.41839	2.079467
C	2.00131	-0.73299	-0.42503	H	3.103871	0.791978	0.757766
C	3.220513	-0.02813	0.057955	H	4.522903	-1.21293	-1.06469
C	4.441504	-0.38715	-0.35985	H	6.277066	1.702543	1.239254
O	5.438547	1.296197	0.979842	H	6.634813	0.063808	-1.94287
C	6.436766	0.836513	-1.19412	H	5.810359	1.610132	-1.64617
C	5.7422	0.249157	0.050966	H	7.395402	1.284362	-0.9097
C	6.638239	-0.80859	0.726033	H	7.59583	-0.35859	1.010971
O	-2.95193	-1.59027	1.788978	H	6.153237	-1.19618	1.625918
H	-1.82765	-2.29981	-1.26077	H	6.84623	-1.64485	0.051909
H	-1.07447	-2.46213	0.29333	H	-2.5865	-0.70976	1.972933

**2-**1*S*3*R*9*S*-conf. 12	**Coordinates (Å)**				**Coordinates (Å)**		
	**X**	**Y**	**Z**		**X**	**Y**	**Z**
O	2.066273	-1.67476	-1.22966	H	-3.48568	-2.59524	0.162775
H	-0.81286	1.234773	-1.99059	H	-5.75078	-1.89168	-0.35197
H	-0.14011	-1.02086	-1.69283	H	-5.8067	-0.60112	-1.56373
C	-0.56025	-0.63809	-0.7597	H	-4.71315	-1.97614	-1.78516
C	-1.61688	-1.74798	-0.34497	H	-4.36581	0.356101	1.719323
C	-3.03735	-1.61531	0.365055	H	-5.78588	0.362674	0.70544
C	-5.13965	-1.29478	-1.03916	H	-5.19418	2.272089	-0.50306
C	-4.04033	-0.53558	-0.27598	H	-4.77373	2.648963	1.145978
C	-4.70073	0.522284	0.696452	H	-2.56346	3.15581	0.512066
C	-4.48104	2.030032	0.291759	H	-3.11986	3.015029	-1.15091
C	-3.03999	2.472455	-0.2034	H	-3.8294	0.946832	-1.83383
C	-3.19946	0.291976	-1.22349	H	-2.65614	-0.33288	-1.93222
O	-2.10124	0.729122	1.027692	H	-1.93258	1.52255	1.550419
C	-2.24459	1.176424	-0.36705	H	1.183863	1.754242	-0.69662
C	-0.86265	0.911965	-0.94521	H	0.229482	1.846475	0.790053
C	0.401844	1.257595	-0.11496	H	1.434965	-0.21815	2.194524
C	0.633443	-0.70019	1.625158	H	0.762561	-1.78371	1.720438
C	0.666154	-0.26619	0.150297	H	-0.32021	-0.4294	2.076396
C	2.001025	-0.74681	-0.42759	H	3.105141	0.776486	0.753235
C	3.223201	-0.0442	0.052544	H	4.527235	-1.21166	-1.08583
C	4.444996	-0.393	-0.37189	H	5.238737	0.789832	1.865765
O	5.532333	1.248387	1.065108	H	6.513947	0.343436	-1.98776
C	6.344044	1.01619	-1.14307	H	5.667902	1.81393	-1.46203
C	5.74268	0.258105	0.051199	H	7.300396	1.460809	-0.85213
C	6.719845	-0.81808	0.560928	H	7.661289	-0.34691	0.85755
O	-2.95215	-1.59396	1.783561	H	6.302943	-1.34133	1.428336
H	-1.83242	-2.297	-1.26957	H	6.929955	-1.5629	-0.21236
H	-1.07862	-2.46746	0.283343	H	-2.58456	-0.71486	1.969881

**2-**1*S*3*S*9*R*-conf. 1	**Coordinates (Å)**				**Coordinates (Å)**		
	**X**	**Y**	**Z**		**X**	**Y**	**Z**
O	2.163311	2.093933	-1.65994	H	-2.27253	-1.76538	1.801035
H	-1.64619	2.246125	1.061362	H	-1.76436	-2.84631	-1.67917
H	-0.25244	0.237136	-0.74134	H	-3.46767	-2.55752	-2.08421
C	-0.4074	0.671107	0.248208	H	-3.05662	-3.63916	-0.76122
C	-0.58689	-0.52599	1.197693	H	-4.92031	-2.17644	-0.18166
C	-1.70728	-1.64399	0.870507	H	-4.37813	-1.36136	1.274334
C	-2.76327	-2.70631	-1.25877	H	-5.95679	0.12176	0.328642
C	-2.82876	-1.49533	-0.30897	H	-5.51194	-0.25004	-1.31479
C	-4.33519	-1.32307	0.185715	H	-4.68409	2.093461	0.261133
C	-5.09323	-0.02669	-0.32769	H	-4.40315	1.765524	-1.45077
C	-4.29297	1.348789	-0.44343	H	-3.30835	-0.16927	-1.92074
C	-2.59279	-0.22073	-1.09526	H	-1.61866	-0.20076	-1.57462
O	-3.00716	0.492703	1.268952	H	-3.36299	1.252266	1.748763
C	-2.84797	0.965748	-0.12493	H	-0.57611	3.695796	-0.54059
C	-1.52031	1.736996	0.101622	H	-0.3846	2.373176	-1.72342
C	-0.49191	2.61252	-0.6627	H	1.618912	1.952091	2.126229
C	0.978046	2.578067	1.497796	H	0.082931	2.818548	2.077229
C	0.624541	1.878438	0.175474	H	1.511494	3.514872	1.303606
C	1.901394	1.55202	-0.58846	H	2.503707	0.074612	0.954112
C	2.801721	0.533633	0.016794	H	4.230725	0.668775	-1.5003
C	3.95525	0.180424	-0.56638	H	3.65847	-2.03173	0.86782
O	4.4243	-1.50194	1.133269	H	6.940592	-0.8923	0.758519
C	6.220669	-0.14754	0.406503	H	6.668802	0.407816	-0.42156
C	4.927884	-0.84991	-0.03859	H	6.009386	0.549913	1.221473
C	5.223236	-1.89092	-1.1351	H	4.30635	-2.41081	-1.43359
O	-0.9067	-2.82578	0.670823	H	5.648904	-1.4202	-2.02622
H	0.348459	-1.0958	1.225166	H	5.935161	-2.6297	-0.75608
H	-0.77885	-0.17093	2.212648	H	-1.49884	-3.58913	0.683008

**2-**1*S*3*S*9*R*-conf. 2	Coordinates (Å)				Coordinates (Å)		
	**X**	**Y**	**Z**		**X**	**Y**	**Z**
O	-2.17991	-2.11906	-1.62406	H	2.330932	1.69238	1.795269
H	1.642055	-2.23189	1.080504	H	1.758806	2.811121	-1.71469
H	0.244102	-0.25407	-0.75045	H	3.468542	2.54778	-2.10627
C	0.402434	-0.66742	0.247304	H	3.011243	3.63217	-0.78112
C	0.582009	0.553081	1.169128	H	4.93329	2.163812	-0.23289
C	1.752896	1.646506	0.868179	H	4.408275	1.362406	1.236869
C	2.757827	2.696214	-1.28536	H	5.963236	-0.13668	0.284821
C	2.835459	1.49458	-0.32552	H	5.500943	0.223047	-1.35627
C	4.349241	1.316602	0.149574	H	4.680271	-2.10228	0.249247
C	5.092609	0.011068	-0.36235	H	4.380903	-1.78684	-1.46183
C	4.284109	-1.36153	-0.45668	H	3.293688	0.155333	-1.92899
C	2.586018	0.213325	-1.09753	H	1.608345	0.196774	-1.56887
O	3.016149	-0.49339	1.264919	H	3.36949	-1.25382	1.745177
C	2.843849	-0.9707	-0.1256	H	0.56671	-3.70361	-0.49516
C	1.514082	-1.73592	0.114356	H	0.370202	-2.39856	-1.69666
C	0.482055	-2.62224	-0.633	H	-1.61499	-1.91941	2.155598
C	-0.97687	-2.5548	1.533902	H	-0.07893	-2.78606	2.112762
C	-0.63071	-1.87571	0.199085	H	-1.51093	-3.49471	1.357009
C	-1.91213	-1.56063	-0.56217	H	-2.50861	-0.06217	0.963729
C	-2.80756	-0.53347	0.03419	H	-4.2277	-0.6738	-1.49137
C	-3.95613	-0.18142	-0.55907	H	-5.03379	2.051418	1.477065
O	-4.40501	1.388569	1.159608	H	-7.0178	0.920732	0.552577
C	-6.29215	0.174524	0.210404	H	-6.69156	-0.29336	-0.6943
C	-4.93172	0.849264	-0.0581	H	-6.18744	-0.5911	0.983763
C	-5.07561	1.968019	-1.10945	H	-4.11346	2.461799	-1.26947
O	1.106865	2.919957	0.680659	H	-5.43098	1.574044	-2.06623
H	-0.34661	1.136623	1.135561	H	-5.79858	2.714723	-0.76266
H	0.721494	0.222593	2.202356	H	0.605851	3.096806	1.487994

**2-**1*S*3*S*9*R*-conf. 3	Coordinates (Å)				Coordinates (Å)		
	**X**	**Y**	**Z**		**X**	**Y**	**Z**
O	2.175813	2.112093	-1.63602	H	-2.33176	-1.69173	1.793677
H	-1.63457	2.229772	1.083323	H	-1.76835	-2.80806	-1.71882
H	-0.24646	0.251835	-0.75497	H	-3.47822	-2.54087	-2.1068
C	-0.40074	0.664258	0.243782	H	-3.02058	-3.6278	-0.78381
C	-0.58115	-0.55662	1.164896	H	-4.93895	-2.15583	-0.23034
C	-1.75546	-1.64687	0.865598	H	-4.40928	-1.3583	1.239849
C	-2.76633	-2.69172	-1.28737	H	-5.96252	0.146219	0.294088
C	-2.8398	-1.49115	-0.32589	H	-5.50514	-0.2119	-1.34872
C	-4.35229	-1.31061	0.152525	H	-4.67542	2.109099	0.258227
C	-5.09387	-0.00248	-0.35549	H	-4.38052	1.795465	-1.45393
C	-4.2825	1.36848	-0.44963	H	-3.2993	-0.14869	-1.92618
C	-2.58958	-0.20931	-1.09668	H	-1.6132	-0.19402	-1.57076
O	-3.01263	0.494857	1.267845	H	-3.36504	1.254707	1.749712
C	-2.84244	0.973859	-0.12231	H	-0.56025	3.702329	-0.49219
C	-1.51029	1.735618	0.115798	H	-0.37041	2.399564	-1.69717
C	-0.47855	2.621103	-0.6327	H	1.62592	1.907134	2.147848
C	0.987675	2.545647	1.529433	H	0.092163	2.777987	2.111566
C	0.635092	1.870427	0.19437	H	1.523748	3.48444	1.352915
C	1.912412	1.554418	-0.57303	H	2.51123	0.05062	0.945745
C	2.811683	0.526476	0.017619	H	4.246857	0.691258	-1.49064
C	3.968236	0.185605	-0.56694	H	3.669893	-2.05844	0.8179
O	4.430166	-1.52987	1.101143	H	6.94759	-0.90398	0.757664
C	6.227382	-0.15455	0.416272	H	6.67938	0.420018	-0.39645
C	4.94059	-0.85207	-0.05318	H	6.007433	0.524654	1.244246
C	5.248053	-1.86937	-1.16838	H	4.335221	-2.38545	-1.48531
O	-1.11276	-2.92191	0.676614	H	5.679673	-1.37922	-2.04604
H	0.345555	-1.14318	1.128859	H	5.958961	-2.61387	-0.79876
H	-0.71709	-0.22676	2.198777	H	-0.62008	-3.10526	1.487613

**2-**1*S*3*S*9*R*-conf. 4	**Coordinates (Å)**				**Coordinates (Å)**		
	**X**	**Y**	**Z**		**X**	**Y**	**Z**
O	-2.16959	-2.09998	-1.64378	H	2.283933	1.758378	1.802439
H	1.651044	-2.24507	1.061594	H	1.759217	2.848665	-1.67389
H	0.251173	-0.23688	-0.73722	H	3.46016	2.559152	-2.08884
C	0.409629	-0.67012	0.252081	H	3.057028	3.640275	-0.76299
C	0.591177	0.52794	1.200211	H	4.922816	2.176456	-0.19194
C	1.714346	1.643614	0.873824	H	4.386187	1.361335	1.266117
C	2.760471	2.707827	-1.25936	H	5.960173	-0.1225	0.314541
C	2.830421	1.496224	-0.31072	H	5.509921	0.250101	-1.32723
C	4.338856	1.323241	0.177656	H	4.686582	-2.09358	0.250516
C	5.094443	0.026611	-0.33879	H	4.399609	-1.76473	-1.46025
C	4.293178	-1.34846	-0.4523	H	3.303852	0.171041	-1.92501
C	2.591244	0.222164	-1.09693	H	1.615408	0.202914	-1.57275
O	3.013943	-0.49303	1.265268	H	3.367872	-1.25427	1.743813
C	2.849433	-0.96506	-0.1284	H	0.576599	-3.69494	-0.53708
C	1.522308	-1.73586	0.102252	H	0.380663	-2.37213	-1.71898
C	0.49161	-2.61163	-0.65866	H	-1.60995	-1.95173	2.137059
C	-0.9703	-2.57733	1.50705	H	-0.07313	-2.81671	2.083811
C	-0.62215	-1.87771	0.183261	H	-1.50338	-3.51481	1.31493
C	-1.903	-1.55347	-0.57538	H	-2.50164	-0.07278	0.96685
C	-2.79989	-0.53429	0.03221	H	-4.21936	-0.66039	-1.49523
C	-3.94908	-0.17802	-0.55734	H	-5.03311	2.028283	1.504162
O	-4.40479	1.367606	1.181344	H	-7.01494	0.907322	0.559451
C	-6.28803	0.166202	0.208995	H	-6.68444	-0.28894	-0.70351
C	-4.92697	0.845092	-0.04545	H	-6.18559	-0.61027	0.97178
C	-5.06764	1.978026	-1.08203	H	-4.10542	2.475201	-1.23064
O	0.917675	2.829399	0.683376	H	-5.4179	1.596742	-2.04581
H	-0.34324	1.099193	1.226453	H	-5.79336	2.718902	-0.72854
H	0.782041	0.17353	2.215719	H	1.512844	3.590342	0.697871

**2-**1*S*3*S*9*R*-conf. 5	Coordinates (Å)				Coordinates (Å)		
	**X**	**Y**	**Z**		**X**	**Y**	**Z**
O	-2.19208	-2.13354	-1.6038	H	2.359922	1.649206	1.798364
H	1.638288	-2.22181	1.087367	H	1.759	2.784882	-1.74127
H	0.242679	-0.25639	-0.75679	H	3.472129	2.573683	-2.09688
C	0.40169	-0.65997	0.24493	H	2.9693	3.63286	-0.76714
C	0.578317	0.568105	1.158047	H	4.945649	2.15112	-0.26137
C	1.770714	1.642	0.883271	H	4.430234	1.359164	1.216531
C	2.750584	2.693852	-1.28151	H	5.968018	-0.15396	0.26412
C	2.843929	1.48737	-0.33081	H	5.502362	0.203127	-1.37662
C	4.361544	1.307897	0.129832	H	4.678066	-2.11445	0.236264
C	5.096177	-0.0037	-0.38077	H	4.370496	-1.80028	-1.47357
C	4.280902	-1.37321	-0.46853	H	3.291326	0.146325	-1.93752
C	2.587322	0.208086	-1.10301	H	1.607947	0.194079	-1.57084
O	3.025647	-0.49774	1.258734	H	3.377602	-1.25953	1.737824
C	2.843912	-0.97587	-0.13025	H	0.556963	-3.70281	-0.47386
C	1.510352	-1.73294	0.11776	H	0.361006	-2.40719	-1.68546
C	0.474578	-2.62242	-0.6202	H	-1.61649	-1.88985	2.165536
C	-0.9801	-2.53246	1.549444	H	-0.08212	-2.76005	2.129682
C	-0.63425	-1.86614	0.208056	H	-1.51622	-3.47284	1.381594
C	-1.9175	-1.55775	-0.55297	H	-2.49717	-0.02358	0.943808
C	-2.80762	-0.51867	0.030492	H	-4.25103	-0.70633	-1.46772
C	-3.96732	-0.18787	-0.55336	H	-5.01977	2.09984	1.434266
O	-4.38881	1.437563	1.119858	H	-7.01015	0.91717	0.605046
C	-6.28547	0.168152	0.266937	H	-6.7046	-0.33441	-0.60977
C	-4.94163	0.849036	-0.06289	H	-6.14933	-0.5696	1.062091
C	-5.12888	1.9274	-1.14897	H	-4.17794	2.426035	-1.35468
O	1.167335	2.946537	0.88002	H	-5.50673	1.494897	-2.0802
H	-0.34182	1.163634	1.11609	H	-5.85057	2.678199	-0.80852
H	0.693408	0.24821	2.197655	H	0.579209	3.002653	0.114442

**2-**1*S*3*S*9*R*-conf. 6	**Coordinates (Å)**				**Coordinates (Å)**		
	**X**	**Y**	**Z**		**X**	**Y**	**Z**
O	-1.75105	1.691609	0.098142	H	3.727341	1.214917	1.485798
H	1.117889	-1.80744	1.414782	H	2.976396	2.430178	-2.00003
H	0.457274	0.554224	-0.38341	H	4.257493	1.398728	-2.63424
C	0.624617	0.145317	0.61315	H	4.625443	2.596883	-1.37991
C	1.554337	1.156742	1.308608	H	5.719792	0.345762	-1.05587
C	3.024915	1.487656	0.70048	H	5.228957	-0.05683	0.579476
C	3.880385	1.874529	-1.7224	H	5.611686	-2.15145	-0.43438
C	3.598023	0.799028	-0.65877	H	5.023334	-1.64941	-1.99585
C	4.907269	-0.09231	-0.46149	H	3.575184	-3.22623	0.021106
C	4.798191	-1.60712	-0.92486	H	3.086481	-2.84007	-1.63062
C	3.433761	-2.40139	-0.68857	H	2.990087	-0.62525	-2.13595
C	2.602439	-0.20712	-1.20271	H	1.657838	0.250542	-1.47813
O	3.157868	-0.98417	1.112242	H	3.2147	-1.81289	1.605987
C	2.472328	-1.34207	-0.14982	H	-0.85763	-2.58785	0.285202
C	1.031346	-1.33632	0.432049	H	-0.64877	-1.41174	-1.04425
C	-0.43693	-1.61694	0.009844	H	-1.21754	0.172597	2.992896
C	-1.14596	-0.73204	2.37975	H	-0.37099	-1.36832	2.814896
C	-0.82598	-0.36736	0.920797	H	-2.09757	-1.26697	2.459893
C	-1.94346	0.507051	0.365768	H	-3.35602	-1.20411	0.371392
C	-3.26388	-0.14427	0.159684	H	-4.19113	1.612181	-0.48518
C	-4.32051	0.550119	-0.28359	H	-6.55972	-1.74449	-0.39509
O	-5.67152	-1.39709	-0.23408	H	-7.07454	-0.16413	-2.21397
C	-6.06348	0.211637	-2.02167	H	-6.04081	1.271449	-2.29235
C	-5.69881	0.002029	-0.53811	H	-5.36232	-0.32891	-2.66306
C	-6.71026	0.719884	0.378149	H	-6.46285	0.540142	1.427733
O	3.145534	2.919495	0.647288	H	-6.7158	1.799122	0.198932
H	1.031803	2.120258	1.331495	H	-7.72043	0.340928	0.186895
H	1.718359	0.867384	2.351167	H	2.49931	3.24976	0.008236

**2-**1*S*3*S*9*R*-conf. 8	Coordinates (Å)				Coordinates (Å)		
	**X**	**Y**	**Z**		**X**	**Y**	**Z**
O	-2.18547	-2.13273	-1.62274	H	2.326695	1.690029	1.795938
H	1.63846	-2.23232	1.078903	H	1.757374	2.811867	-1.71356
H	0.241248	-0.25497	-0.7532	H	3.467339	2.548561	-2.10421
C	0.399024	-0.6684	0.244607	H	3.009308	3.631988	-0.77852
C	0.578051	0.551708	1.167182	H	4.930768	2.163158	-0.23007
C	1.749527	1.645261	0.868278	H	4.404678	1.360821	1.238771
C	2.756096	2.696473	-1.28367	H	5.960229	-0.13773	0.286592
C	2.832967	1.494144	-0.32466	H	5.498807	0.223085	-1.3545
C	4.346386	1.315745	0.151403	H	4.677044	-2.10319	0.249141
C	5.089953	0.010514	-0.36093	H	4.378742	-1.78669	-1.46189
C	4.281389	-1.36196	-0.45656	H	3.291917	0.155765	-1.92871
C	2.583827	0.213409	-1.09759	H	1.606413	0.197354	-1.56948
O	3.012202	-0.49475	1.264765	H	3.367463	-1.25472	1.74434
C	2.840981	-0.9712	-0.12617	H	0.56506	-3.70488	-0.49714
C	1.511115	-1.73665	0.112557	H	0.368859	-2.4002	-1.69904
C	0.480092	-2.6236	-0.63525	H	-1.61888	-1.92031	2.15209
C	-0.98013	-2.55587	1.531109	H	-0.08238	-2.78604	2.11063
C	-0.63342	-1.87729	0.196017	H	-1.51349	-3.49621	1.354522
C	-1.91534	-1.56674	-0.56586	H	-2.50719	-0.05767	0.94968
C	-2.81183	-0.53606	0.024252	H	-4.22914	-0.67161	-1.50324
C	-3.95935	-0.18038	-0.56918	H	-4.61662	0.653871	1.863873
O	-4.5307	1.372332	1.220492	H	-7.04289	1.007845	0.397846
C	-6.34078	0.250968	0.036654	H	-6.68451	-0.10795	-0.93797
C	-4.92908	0.86047	-0.0569	H	-6.35274	-0.59608	0.731242
C	-4.92122	2.07088	-1.00439	H	-3.92871	2.528971	-1.02232
O	1.103863	2.918892	0.681426	H	-5.18682	1.772457	-2.02194
H	-0.35008	1.135967	1.133003	H	-5.64559	2.813647	-0.65726
H	0.716337	0.220565	2.20036	H	0.603537	3.096057	1.489118

**2-**1*S*3*S*9*R*-conf. 10	Coordinates (Å)				Coordinates (Å)		
	**X**	**Y**	**Z**		**X**	**Y**	**Z**
O	-1.75343	1.699552	0.137955	H	3.738573	1.250319	1.470786
H	1.119161	-1.81059	1.420635	H	2.984625	2.447746	-1.98471
H	0.457592	0.560028	-0.36722	H	4.218978	1.366693	-2.65864
C	0.62583	0.144601	0.626453	H	4.654172	2.563109	-1.42555
C	1.566127	1.14609	1.321619	H	5.713619	0.346126	-1.06003
C	3.021875	1.493068	0.681584	H	5.222233	-0.05556	0.575696
C	3.877138	1.863601	-1.74355	H	5.607446	-2.14981	-0.44162
C	3.592951	0.801556	-0.66546	H	5.012669	-1.64757	-2.00053
C	4.901189	-0.09144	-0.46525	H	3.572373	-3.22395	0.023399
C	4.79165	-1.60575	-0.92865	H	3.078373	-2.84036	-1.62738
C	3.428623	-2.40018	-0.68711	H	2.97824	-0.6254	-2.13499
C	2.594099	-0.20645	-1.20088	H	1.648611	0.251421	-1.47286
O	3.158391	-0.98048	1.112358	H	3.217015	-1.80824	1.607513
C	2.468723	-1.34048	-0.14684	H	-0.86078	-2.58555	0.293889
C	1.030028	-1.33664	0.439362	H	-0.65419	-1.40539	-1.03229
C	-0.43969	-1.61421	0.020541	H	-1.2104	0.166377	3.011224
C	-1.1422	-0.73632	2.394839	H	-0.36779	-1.37605	2.825957
C	-0.8248	-0.36716	0.936418	H	-2.09497	-1.26927	2.475013
C	-1.94284	0.510857	0.388533	H	-3.35096	-1.2038	0.373326
C	-3.2601	-0.14244	0.168703	H	-4.18636	1.614665	-0.47587
C	-4.31465	0.55131	-0.28046	H	-6.54657	-1.74875	-0.42202
O	-5.66164	-1.39908	-0.24846	H	-7.04486	-0.16652	-2.24294
C	-6.03675	0.210835	-2.03859	H	-6.01278	1.270929	-2.30804
C	-5.68874	0.000367	-0.55113	H	-5.32723	-0.32787	-2.6723
C	-6.71243	0.714602	0.354248	H	-6.47661	0.534417	1.406428
O	3.064498	2.917075	0.465441	H	-6.71873	1.794008	0.176087
H	1.039612	2.107491	1.355878	H	-7.71936	0.333193	0.151127
H	1.748316	0.836874	2.35548	H	2.881111	3.329741	1.319913

**2-**1*S*3*S*9*R*-conf. 12	Coordinates (Å)				Coordinates (Å)		
	**X**	**Y**	**Z**		**X**	**Y**	**Z**
O	-2.17677	-2.11419	-1.64082	H	2.282113	1.756191	1.802873
H	1.646867	-2.24491	1.060286	H	1.760742	2.849315	-1.67313
H	0.249174	-0.23628	-0.73991	H	3.461765	2.558805	-2.08707
C	0.406777	-0.66982	0.249395	H	3.058467	3.639281	-0.76072
C	0.588937	0.527476	1.198463	H	4.922827	2.173703	-0.18961
C	1.713215	1.642607	0.873695	H	4.384631	1.358284	1.267692
C	2.761593	2.70743	-1.258	H	5.958084	-0.12632	0.316255
C	2.83	1.495159	-0.31011	H	5.509025	0.247445	-1.32558
C	4.33796	1.320767	0.179179	H	4.682927	-2.09639	0.250552
C	5.092836	0.023816	-0.33748	H	4.3972	-1.76646	-1.46018
C	4.290541	-1.35057	-0.45211	H	3.303321	0.170384	-1.92482
C	2.590289	0.221718	-1.09713	H	1.614724	0.203555	-1.57354
O	3.01074	-0.49488	1.265051	H	3.366746	-1.25559	1.742891
C	2.846935	-0.96615	-0.12894	H	0.573349	-3.69528	-0.5383
C	1.519123	-1.73615	0.100612	H	0.378695	-2.37307	-1.72103
C	0.488826	-2.61203	-0.66049	H	-1.61469	-1.94894	2.133422
C	-0.97457	-2.57548	1.504699	H	-0.0779	-2.81398	2.082514
C	-0.62514	-1.87719	0.180359	H	-1.50748	-3.51315	1.313201
C	-1.90644	-1.5575	-0.57904	H	-2.49621	-0.05918	0.947801
C	-2.80314	-0.53256	0.020559	H	-4.22543	-0.66418	-1.50245
C	-3.95423	-0.17756	-0.56639	H	-4.6054	0.637651	1.873663
O	-4.527	1.361134	1.234967	H	-7.03995	0.99026	0.417774
C	-6.33549	0.238977	0.049571	H	-6.68064	-0.11534	-0.92625
C	-4.92685	0.855637	-0.04435	H	-6.34134	-0.61251	0.738791
C	-4.92752	2.072057	-0.98416	H	-3.93723	2.534917	-1.00206
O	0.917466	2.82897	0.683517	H	-5.19479	1.778817	-2.0028
H	-0.34471	1.099962	1.224929	H	-5.65433	2.80921	-0.63023
H	0.779027	0.171942	2.213713	H	1.512985	3.589612	0.699455

**2-**1*S*3*S*9*R*-conf. 14	Coordinates (Å)				Coordinates (Å)		
	**X**	**Y**	**Z**		**X**	**Y**	**Z**
O	-2.17012	-2.09185	-1.64124	H	2.356696	1.653789	1.794082
H	1.62712	-2.21906	1.092842	H	1.782154	2.776642	-1.75493
H	0.249742	-0.25527	-0.76622	H	3.496986	2.559284	-2.09747
C	0.40106	-0.65526	0.238089	H	2.987926	3.625295	-0.7756
C	0.575628	0.576034	1.146938	H	4.956924	2.138859	-0.25122
C	1.773385	1.644882	0.875276	H	4.429565	1.354114	1.2263
C	2.770185	2.684788	-1.28763	H	5.968423	-0.16745	0.288701
C	2.853489	1.481826	-0.3315	H	5.513437	0.184741	-1.35607
C	4.367547	1.299058	0.139394	H	4.671731	-2.12363	0.261359
C	5.10083	-0.01687	-0.3618	H	4.37601	-1.81558	-1.45164
C	4.281554	-1.384	-0.44899	H	3.306145	0.132981	-1.93019
C	2.597252	0.200418	-1.1003	H	1.62078	0.187951	-1.57424
O	3.018007	-0.49771	1.266858	H	3.367111	-1.25783	1.750677
C	2.843825	-0.98061	-0.12138	H	0.548772	-3.70033	-0.47116
C	1.506432	-1.73265	0.121127	H	0.365196	-2.40701	-1.68731
C	0.47168	-2.62003	-0.62097	H	-1.62816	-1.87485	2.154231
C	-0.99454	-2.52118	1.539365	H	-0.09975	-2.75465	2.122097
C	-0.63956	-1.85822	0.198841	H	-1.53614	-3.45819	1.370354
C	-1.91435	-1.54133	-0.57265	H	-2.52657	-0.04781	0.951658
C	-2.82043	-0.52015	0.019637	H	-4.24887	-0.68573	-1.49484
C	-3.97683	-0.18308	-0.56752	H	-3.70106	2.054299	0.83568
O	-4.45883	1.517653	1.110182	H	-6.97	0.87916	0.747527
C	-6.24339	0.13654	0.404855	H	-6.68724	-0.43472	-0.41464
C	-4.95814	0.845226	-0.05198	H	-6.02411	-0.54735	1.229139
C	-5.26544	1.86844	-1.16183	H	-4.35405	2.392435	-1.46985
O	1.174212	2.951338	0.863082	H	-5.68889	1.381771	-2.04539
H	-0.34232	1.174413	1.095612	H	-5.98309	2.605924	-0.79121
H	0.682169	0.260852	2.188871	H	0.596111	3.008366	0.089987

**2-**1*S*3*S*9*S*-conf. 2	Coordinates (Å)				Coordinates (Å)		
	**X**	**Y**	**Z**		**X**	**Y**	**Z**
O	-2.45798	-2.64894	-0.2806	H	2.105402	1.120971	1.655338
H	1.171301	-1.65578	-1.99525	H	4.555247	2.390936	-1.59246
H	-0.46762	-0.09796	-1.32167	H	2.915032	3.060101	-1.50696
C	0.191993	-0.31037	-0.47253	H	4.066276	3.370978	-0.20503
C	0.599618	1.123867	0.058001	H	4.242142	0.500819	1.594148
C	1.968327	1.592856	0.684124	H	5.343858	1.354492	0.52833
C	3.72167	2.61429	-0.91693	H	5.644762	-0.43987	-0.94194
C	3.267718	1.32712	-0.20952	H	5.682088	-1.15738	0.645975
C	4.493218	0.661302	0.544519	H	3.972488	-2.66175	0.05384
C	5.012672	-0.68932	-0.08309	H	4.159973	-2.06787	-1.59291
C	3.940061	-1.7561	-0.56638	H	3.638493	0.125117	-1.96455
C	2.847125	0.287853	-1.22625	H	1.979043	0.614676	-1.79941
O	2.431336	-0.88114	0.979596	H	2.706932	-1.71862	1.372235
C	2.58691	-1.04795	-0.46551	H	-0.14907	-3.23405	-0.66488
C	1.193999	-1.46842	-0.91613	H	0.950859	-3.00893	0.704786
C	0.385964	-2.48854	-0.07354	H	-0.89321	-2.01954	2.495312
C	-0.51285	-1.13328	1.974492	H	-1.13098	-0.2815	2.276402
C	-0.54864	-1.36409	0.455153	H	0.511382	-0.94836	2.29861
C	-2.01215	-1.55249	0.049688	H	-2.42817	0.608636	0.364962
C	-2.87619	-0.34034	0.091866	H	-4.60196	-1.37446	-0.46837
C	-4.18213	-0.40686	-0.19834	H	-5.04991	2.658448	0.182361
O	-4.42396	1.921109	0.180299	H	-5.83591	0.38089	1.843837
C	-6.26391	0.468644	0.841678	H	-6.98606	1.292755	0.840622
C	-5.15174	0.74451	-0.19003	H	-6.80472	-0.45358	0.609307
C	-5.74898	0.917803	-1.60102	H	-6.47532	1.738147	-1.60063
O	1.729872	2.999117	0.896741	H	-4.95877	1.15137	-2.31952
H	-0.14715	1.399174	0.811664	H	-6.26526	0.01112	-1.93026
H	0.42745	1.823593	-0.76926	H	2.41114	3.313498	1.505542

**2**-1*S*3*S*9*S*-conf. 5	Coordinates (Å)				Coordinates (Å)		
	**X**	**Y**	**Z**		**X**	**Y**	**Z**
O	-2.45766	-2.64594	-0.29745	H	2.11525	1.109898	1.656566
H	1.174047	-1.64894	-1.99746	H	4.585707	2.403268	-1.55757
H	-0.46605	-0.09263	-1.32323	H	2.943944	3.043313	-1.54017
C	0.191322	-0.3081	-0.47313	H	4.035932	3.378832	-0.18404
C	0.603275	1.124929	0.063865	H	4.24605	0.496933	1.598535
C	1.979179	1.592373	0.694378	H	5.349119	1.35236	0.536597
C	3.731464	2.616028	-0.90608	H	5.646442	-0.43843	-0.94146
C	3.273501	1.327466	-0.20322	H	5.685113	-1.15982	0.644636
C	4.498115	0.659791	0.549921	H	3.973644	-2.66146	0.051354
C	5.015226	-0.68939	-0.0824	H	4.160082	-2.06421	-1.59424
C	3.941132	-1.75453	-0.5669	H	3.639838	0.128917	-1.96095
C	2.849851	0.291263	-1.22128	H	1.981837	0.620155	-1.79343
O	2.431406	-0.88448	0.981474	H	2.710373	-1.72177	1.372007
C	2.587912	-1.04641	-0.4639	H	-0.14853	-3.23072	-0.67443
C	1.194964	-1.46469	-0.91778	H	0.948479	-3.00943	0.698395
C	0.385184	-2.48704	-0.0797	H	-0.89989	-2.02517	2.487634
C	-0.51724	-1.13787	1.970294	H	-1.13449	-0.28611	2.273974
C	-0.5508	-1.36424	0.450286	H	0.506749	-0.95601	2.296959
C	-2.01341	-1.55129	0.040772	H	-2.43174	0.607682	0.368039
C	-2.87849	-0.34014	0.088864	H	-4.60286	-1.37291	-0.4779
C	-4.18425	-0.40639	-0.20222	H	-5.05657	2.654832	0.200023
O	-4.42807	1.919784	0.186241	H	-5.83352	0.370433	1.847335
C	-6.2643	0.461945	0.846714	H	-6.98763	1.285038	0.85106
C	-5.15533	0.743635	-0.18683	H	-6.80453	-0.45998	0.611855
C	-5.75658	0.9221	-1.59548	H	-6.48402	1.741416	-1.58957
O	1.816945	2.977144	1.037844	H	-4.9686	1.159902	-2.31505
H	-0.1407	1.400798	0.82002	H	-6.27243	0.0161	-1.92728
H	0.424715	1.821091	-0.76804	H	1.618907	3.467722	0.227888

**2-**1*S*3*S*9*S*-conf. 10	**Coordinates (Å)**				**Coordinates (Å)**		
	**X**	**Y**	**Z**		**X**	**Y**	**Z**
O	-2.4581	-2.66291	-0.27876	H	2.100051	1.111438	1.661693
H	1.163929	-1.64943	-2.00032	H	4.533992	2.407459	-1.58783
H	-0.47775	-0.10075	-1.31396	H	2.891611	3.07003	-1.49307
C	0.184909	-0.31531	-0.46775	H	4.046328	3.378484	-0.19365
C	0.589012	1.117464	0.069287	H	4.238703	0.499839	1.590315
C	1.958044	1.588024	0.693486	H	5.33374	1.362837	0.525205
C	3.701995	2.62419	-0.90822	H	5.681479	-1.14832	0.628874
C	3.255302	1.331694	-0.2059	H	5.636181	-0.42289	-0.95522
C	4.48567	0.666505	0.540658	H	3.975321	-2.65594	0.034741
C	5.007916	-0.67901	-0.09554	H	4.155163	-2.05296	-1.60951
C	3.937557	-1.74723	-0.58069	H	3.624684	0.14	-1.96824
C	2.835151	0.296103	-1.22657	H	1.964092	0.622714	-1.79532
O	2.430241	-0.886	0.974632	H	2.715517	-1.72237	1.362679
C	2.58221	-1.04458	-0.47181	H	0.956512	-3.01694	0.69356
C	1.189432	-1.4677	-0.92034	H	-0.14655	-3.23956	-0.67406
C	0.387456	-2.49499	-0.08061	H	-1.12986	-0.30573	2.285863
C	-0.50987	-1.15373	1.97709	H	-0.88529	-2.04416	2.494288
C	-0.54938	-1.37645	0.456581	H	0.514788	-0.96683	2.298638
C	-2.01307	-1.56734	0.054107	H	-2.43057	0.589124	0.386925
C	-2.88005	-0.35676	0.101177	H	-4.59953	-1.37367	-0.50654
C	-4.18154	-0.41205	-0.21114	H	-4.31386	1.79705	1.242417
O	-4.51757	1.945654	0.30757	H	-6.89107	-0.47381	0.265144
C	-6.3729	0.405606	0.658894	H	-6.08141	0.189931	1.692582
C	-5.14024	0.757395	-0.19547	H	-7.06917	1.249125	0.662027
C	-5.55707	1.09303	-1.63643	H	-6.27483	1.918719	-1.62813
O	1.715041	2.992175	0.914423	H	-4.68339	1.392539	-2.22143
H	0.411568	1.82103	-0.75355	H	-6.02258	0.230189	-2.11999
H	-0.15625	1.386031	0.826918	H	2.398277	3.306334	1.521137

**2**-1*S*3*S*9*S*-conf. 11	Coordinates (Å)				Coordinates (Å)		
	**X**	**Y**	**Z**		**X**	**Y**	**Z**
O	-2.45229	-2.65441	-0.30798	H	2.090709	1.130401	1.649624
H	1.181859	-1.66789	-1.99173	H	4.55497	2.387287	-1.59219
H	-0.46435	-0.11157	-1.33369	H	2.91309	3.053742	-1.51927
C	0.191751	-0.31783	-0.48026	H	4.056775	3.373923	-0.21284
C	0.592995	1.120111	0.044806	H	4.228544	0.513786	1.602765
C	1.95751	1.59616	0.674916	H	5.334066	1.36452	0.538599
C	3.717325	2.612717	-0.92242	H	5.646701	-0.43652	-0.92118
C	3.262108	1.328476	-0.21049	H	5.677812	-1.14577	0.67058
C	4.484976	0.669428	0.553707	H	3.974569	-2.65719	0.07793
C	5.010935	-0.68311	-0.06423	H	4.169413	-2.07184	-1.57097
C	3.943378	-1.75499	-0.54735	H	3.644692	0.118109	-1.95727
C	2.849184	0.282902	-1.2239	H	1.983347	0.604774	-1.80328
O	2.423954	-0.87531	0.985709	H	2.70355	-1.7082	1.385217
C	2.58805	-1.04952	-0.45747	H	-0.14202	-3.24264	-0.66127
C	1.198587	-1.47563	-0.9134	H	0.950877	-3.0095	0.712945
C	0.388632	-2.49376	-0.07031	H	-0.90378	-2.01653	2.490648
C	-0.52222	-1.13199	1.967833	H	-1.14232	-0.27971	2.264125
C	-0.55076	-1.36919	0.449389	H	0.500378	-0.94463	2.295523
C	-2.01112	-1.56042	0.03506	H	-2.43268	0.595923	0.366019
C	-2.88025	-0.35132	0.081756	H	-4.60884	-1.38295	-0.4723
C	-4.18755	-0.41499	-0.20352	H	-3.90449	2.172474	-0.69184
O	-4.47585	1.990047	0.068315	H	-5.5962	0.580443	1.935642
C	-6.13512	0.57788	0.984426	H	-6.85338	1.403196	0.984665
C	-5.15027	0.751121	-0.18293	H	-6.68426	-0.3632	0.896255
C	-5.90438	0.82381	-1.52421	H	-6.60571	1.662913	-1.505
O	1.714015	3.002961	0.877744	H	-5.20635	0.971502	-2.35538
H	-0.15799	1.397452	0.793579	H	-6.46508	-0.09608	-1.71512
H	0.423128	1.815006	-0.787	H	2.391804	3.323305	1.487322

**2-**1*S*3*S*9*S*-conf. 15	Coordinates (Å)				Coordinates (Å)		
	**X**	**Y**	**Z**		**X**	**Y**	**Z**
O	-2.45717	-2.66006	-0.29888	H	2.107995	1.101453	1.662007
H	1.168357	-1.64379	-2.00258	H	4.565675	2.41928	-1.55199
H	-0.47583	-0.09691	-1.31728	H	2.921434	3.052555	-1.52818
C	0.184353	-0.3138	-0.46973	H	4.015112	3.386537	-0.17296
C	0.592179	1.118199	0.072918	H	4.240802	0.497278	1.596096
C	1.967789	1.58802	0.702471	H	5.338036	1.361684	0.535385
C	3.712042	2.625681	-0.89765	H	5.638603	-0.42127	-0.95146
C	3.260768	1.332234	-0.19951	H	5.683771	-1.14969	0.63124
C	4.489717	0.665819	0.547631	H	3.976741	-2.65511	0.035162
C	5.010398	-0.67848	-0.09205	H	4.156848	-2.04966	-1.6081
C	3.939218	-1.74546	-0.57885	H	3.627414	0.143056	-1.9635
C	2.838583	0.299041	-1.22124	H	1.967996	0.627236	-1.78988
O	2.429554	-0.8885	0.977081	H	2.715838	-1.72535	1.363238
C	2.583482	-1.04313	-0.46939	H	0.95396	-3.01775	0.687367
C	1.191125	-1.46461	-0.92211	H	-0.14537	-3.23678	-0.68404
C	0.387023	-2.49395	-0.08703	H	-1.13376	-0.30992	2.281693
C	-0.51469	-1.15813	1.971734	H	-0.89267	-2.04929	2.485782
C	-0.5515	-1.37706	0.450651	H	0.509639	-0.97419	2.296042
C	-2.01412	-1.5667	0.043586	H	-2.43463	0.586756	0.39194
C	-2.88245	-0.35744	0.098105	H	-4.60031	-1.37145	-0.5192
C	-4.18359	-0.41176	-0.21587	H	-4.31907	1.783394	1.257238
O	-4.52228	1.940722	0.323703	H	-6.08628	0.171103	1.690881
C	-6.37674	0.395853	0.658835	H	-7.07389	1.23858	0.668744
C	-5.14346	0.756579	-0.19091	H	-6.89346	-0.4805	0.256474
C	-5.55901	1.105083	-1.62916	H	-6.27747	1.930062	-1.61399
O	1.800776	2.970341	1.052927	H	-4.68499	1.410669	-2.21052
H	-0.151	1.387875	0.832127	H	-6.02332	0.246368	-2.12113
H	0.40895	1.817456	-0.75528	H	1.601055	3.464373	0.245501

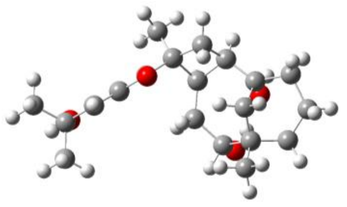

2-1*R*3*R*9*R*-conf. 1 (25.33%)

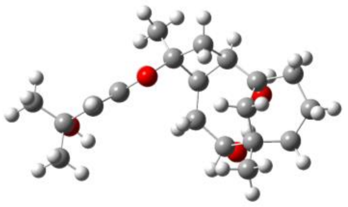

2-1*R*3*R*9*R*-conf. 2 (28.28%)

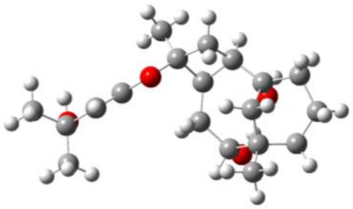

2-1*R*3*R*9*R*-conf. 3 (28.25%)

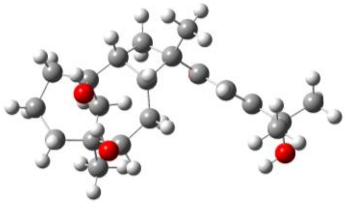

2-1*R*3*R*9*R*-conf. 8 (2.28%)

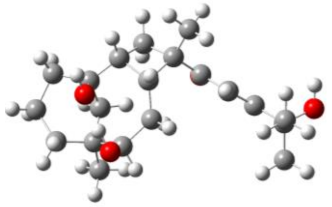

2-1*R*3*R*9*R*-conf. 9 (2.64%)

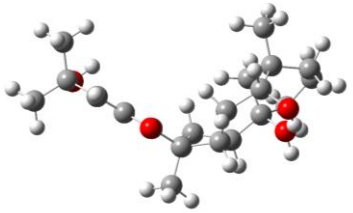

2-1*R*3*R*9*R*-conf. 15 (3.54%)

Figure S29. The cartesian coordinates of the dominant conformers for conformers **2**-1*R*3*R*9*R*.

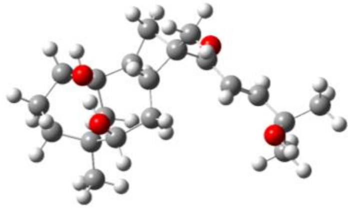

2-1*R*3*R*9*S*-conf. 1 (11.04%)

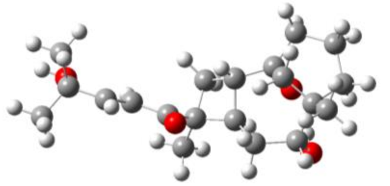

2-1*R*3*R*9*S*-conf. 2 (11.35%)

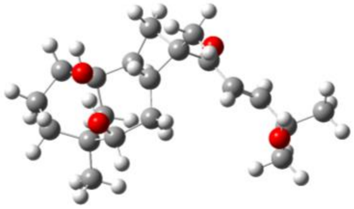

2-1*R*3*R*9*S*-conf. 4 (23.33%)

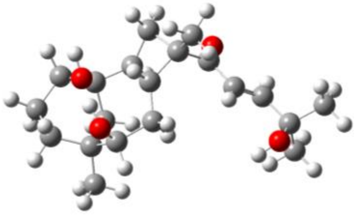

2-1*R*3*R*9*S*-conf. 5 (23.25%)

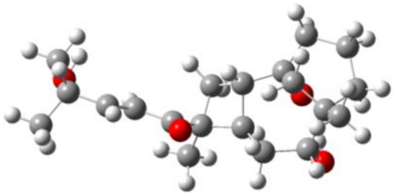

2-1*R*3*R*9*S*-conf. 9 (12.53%)

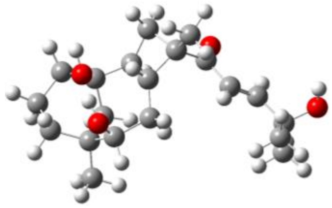

2-1*R*3*R*9*S*-conf. 11 (3.53%)

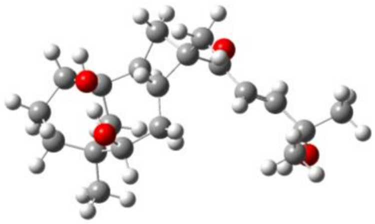

2-1*R*3*R*9*S*-conf. 13 (4.51%)

Figure S30. The cartesian coordinates of the dominant conformers for conformers **2**-1*R*3*R*9*S*.

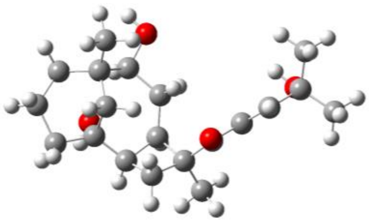

2-1*R*3*S*9*R*-conf. 1 (14.31%)

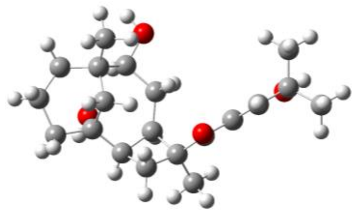

2-1*R*3*R*9*S*-conf. 2 (8.14%)

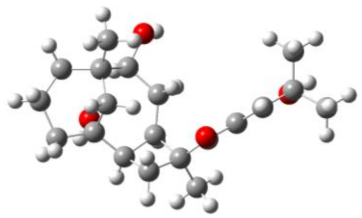

2-1*R*3*R*9*S*-conf. 3 (7.57%)

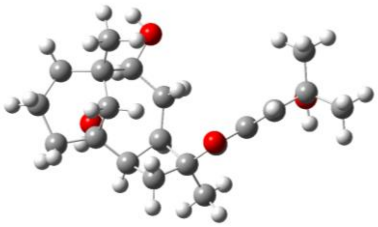

2-1*R*3*R*9*S*-conf. 5 (12.82%)

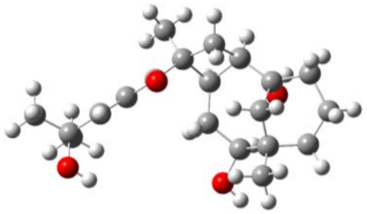

2-1*R*3*R*9*S*-conf. 7 (2.08%)

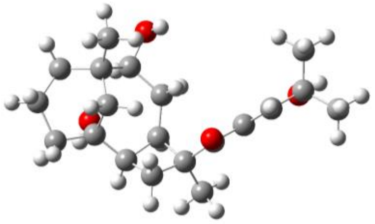

2-1*R*3*R*9*S*-conf. 8 (24.87%)

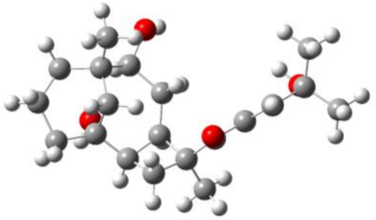

2-1*R*3*R*9*S*-conf. 11 (14.68%)

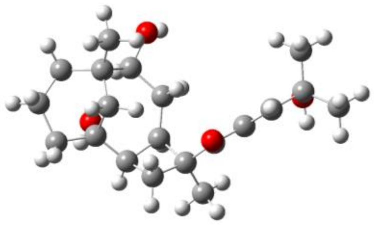

2-1*R*3*R*9*S*-conf. 12 (13.04%)

Figure S31. The cartesian coordinates of the dominant conformers for conformers **2**-1*R*3*S*9*R*.

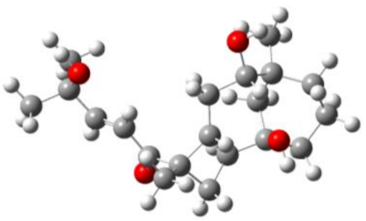

2-1*R*3*S*9*S*-conf. 1 (5.62%)

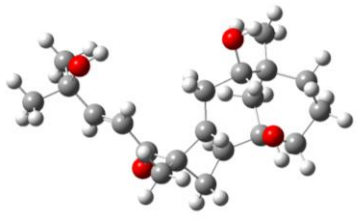

2-1*R*3*S*9*S*-conf. 2 (19.16%)

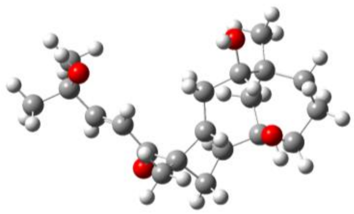

2-1*R*3*S*9*S*-conf. 4 (4.36%)

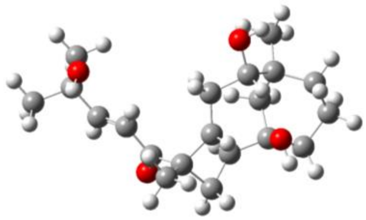

2-1*R*3*S*9*S*-conf. 5 (19.55%)

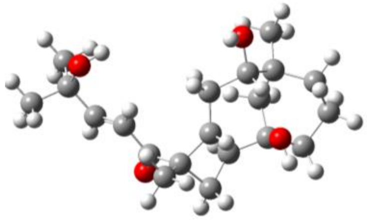

2-1*R*3*S*9*S*-conf. 7 (12.76%)

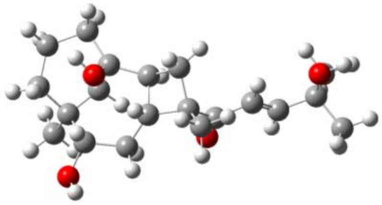

2-1*R*3*S*9*S*-conf. 8 (2.23%)

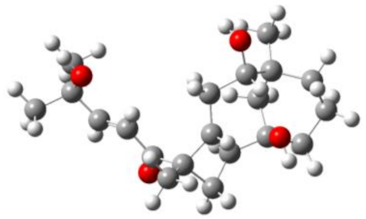

2-1*R*3*S*9*S*-conf. 11 (13.58%)

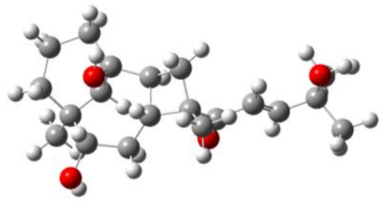

2-1*R*3*S*9*S*-conf. 13 (17.18%)

Figure S32. The cartesian coordinates of the dominant conformers for conformers **2**-1*R*3*S*9*S*.

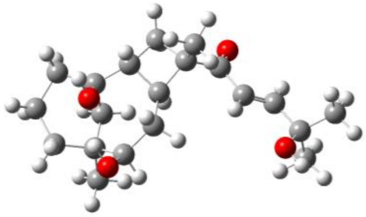

2-1*S*3*R*9*R*-conf. 1 (3.92%)

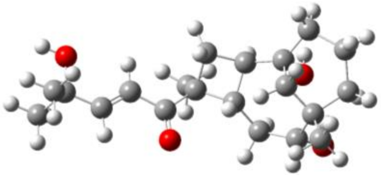

2-1*S*3*R*9*R*-conf. 2 (10.47%)

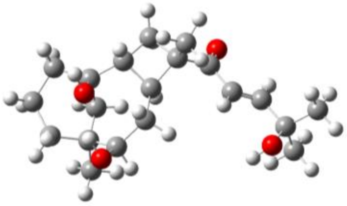

2-1*S*3*R*9*R*-conf. 3 (12.64%)

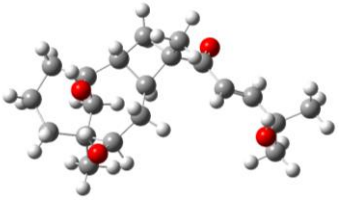

2-1*S*3*R*9*R*-conf. 5 (6.31%)

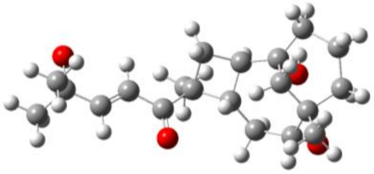

2-1*S*3*R*9*R*-conf. 7 (12.71%)

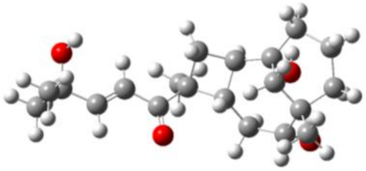

2-1*S*3*R*9*R*-conf. 8 (45.34%)

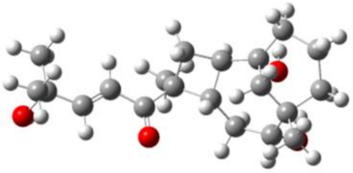

2-1*S*3*R*9*R*-conf. 18 (2.25%)

Figure S33. The cartesian coordinates of the dominant conformers for conformers **2**-1*S*3*R*9*R*.

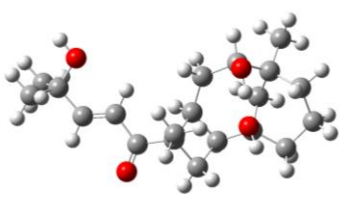

2-1*S*3*R*9*S*-conf. 1 (4.69%)

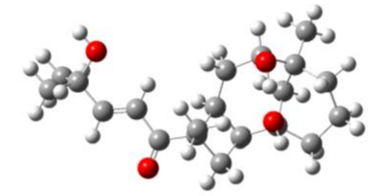

2-1*S*3*R*9*S*-conf. 2 (4.68%)

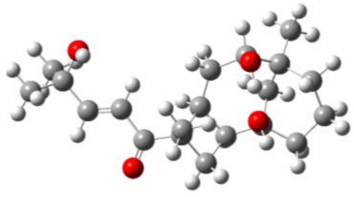

2-1*S*3*R*9*S*-conf. 3 (16.52%)

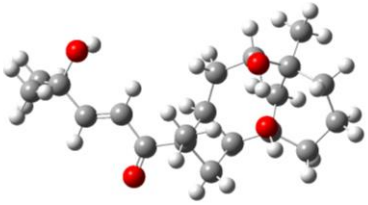

2-1*S*3*R*9*S*-conf. 4 (12.88%)

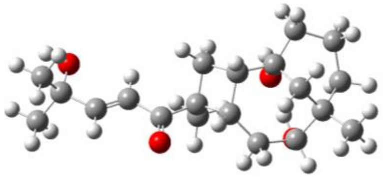

2-1*S*3*R*9*S*-conf. 6 (5.61%)

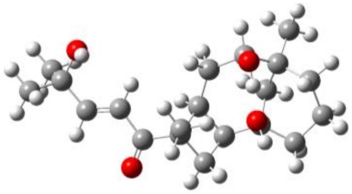

2-1*S*3*R*9*S*-conf. 7 (17.63%)

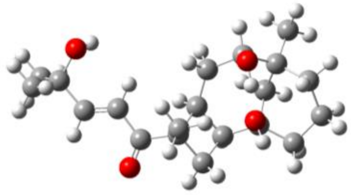

2-1*S*3*R*9*S*-conf. 10 (12.87%)

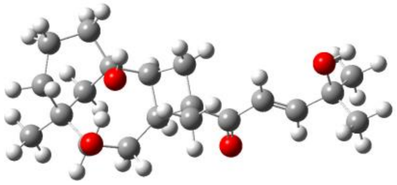

2-1*S*3*R*9*S*-conf. 11 (5.61%)

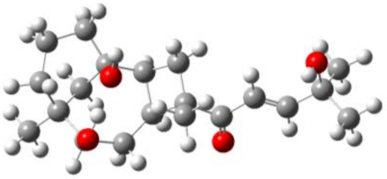

2-1*S*3*R*9*S*-conf. 12 (14.91%)

Figure S34. The cartesian coordinates of the dominant conformers for conformers **2**-1*S*3*R*9*S*.

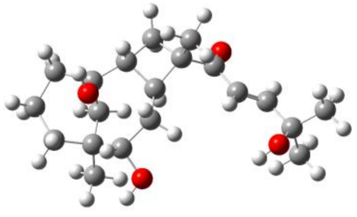

2-1*S*3*S*9*R*-conf. 1 (13.84%)

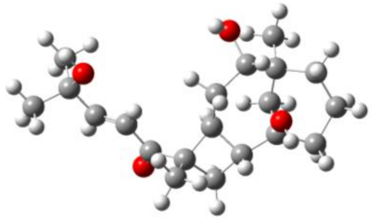

2-1*S*3*S*9*R*-conf. 2 (3.99%)

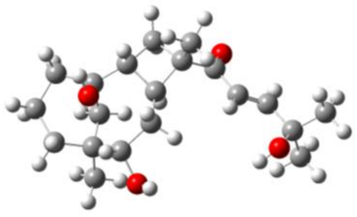

2-1*S*3*S*9*R*-conf. 3 (5.67%)

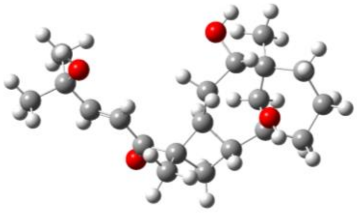

2-1*S*3*S*9*R*-conf. 4 (10.79%)

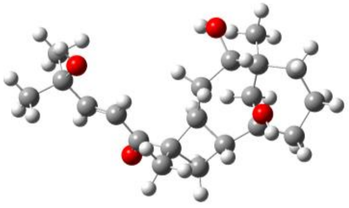

2-1*S*3*S*9*R*-conf. 5 (8.36%)

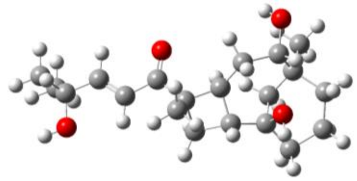

2-1*S*3*S*9*R*-conf. 6 (21.48%)

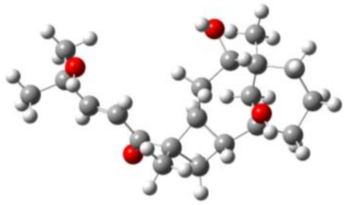

2-1*S*3*S*9*R*-conf. 8 (3.69%)

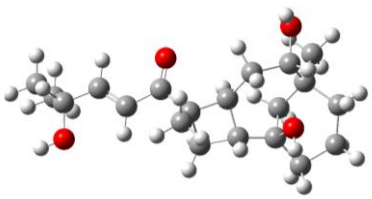

2-1*S*3*S*9*R*-conf. 10 (4.46%)

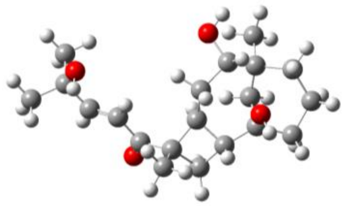

2-1*S*3*S*9*R*-conf. 12 (11.19%)

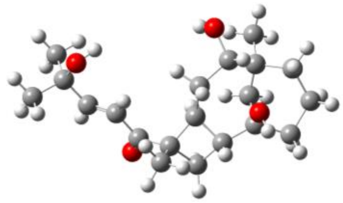

2-1*S*3*S*9*R*-conf. 14 (12.93%)

Figure S35. The cartesian coordinates of the dominant conformers for conformers **2**-1*S*3*S*9*R*.

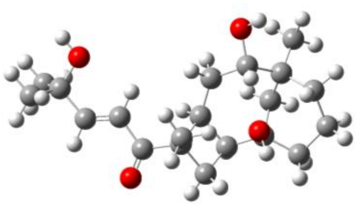

2-1*S*3*S*9*S*-conf. 2 (2.58%)

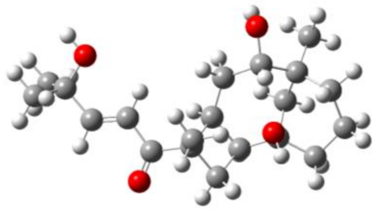

2-1*S*3*S*9*S*-conf. 5 (13.40%)

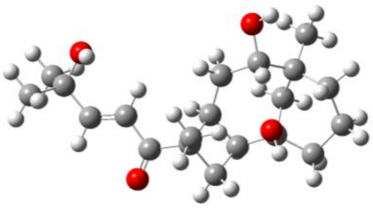

2-1*S*3*S*9*S*-conf. 10 (15.80%)

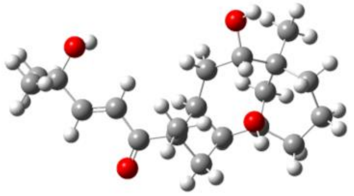

2-1*S*3*S*9*S*-conf. 11 (20.21%)

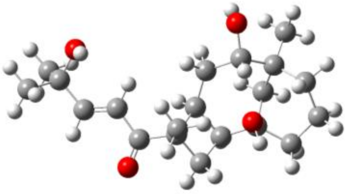

2-1*S*3*S*9*S*-conf. 15 (44.15%)

Figure S36. The cartesian coordinates of the dominant conformers for conformers **2**-1*S*3*S*9*S*.

3.4. DP4+ results of compounds 1 and 2

Functional		Solvent?		Basis Set		Type of Data	
mPW1PW91		PCM		6-311+G(d,p)		Unscaled Shifts	
		DP4+	100.00%	0.00%	-	-	-
Nuclei	sp2?	Experimental	Isomer 1	Isomer 2	Isomer 3	Isomer 4	Isomer 5
C		44.3	42.5	42.6			
C		27.5	27.7	28.7			
C		40.1	39.4	31.2			
C		39.3	42.3	45.4			
C		75.1	78.5	80.9			
C		29.5	32.1	35.4			
C		27.7	38.2	38.6			
C		71.8	78.9	83.6			
C		50.5	49.5	47.9			
C		32.1	32.9	33.6			
C		51.1	55.1	57.4			
C	x	206.4	207.60	209.43			
C	x	121.9	120.24	120.15			
C	x	155.5	159.59	161.88			
C		71.4	72.07	72.12			
C		29.3	26.12	22.87			
C		29.3	22.90	25.15			
C		15.4	10.26	10.38			
C		47.3	39.57	39.96			
C		31.8	31.89	29.36			
H		2.21	2.91	3.01			
H		1.66	1.82	1.89			
H		1.52	1.98	2.07			
H		1.34	1.34	1.33			
H		1.64	1.96	2.35			
H		3.31	3.35	3.57			
H		1.69	1.72	2.46			
H		2.08	2.19	1.74			
H		1.66	1.78	2.08			
H		1.75	1.72	1.78			
H		2.03	2.28	2.36			
H		1.7	1.76	1.85			
H		1.96	1.93	1.85			
H		6.42	6.77	6.78			
H		6.94	7.86	7.89			
H		1.32	1.33	1.42			
H		1.32	1.4	1.52			
H		1.29	1.39	1.42			
H		1.83	1.47	1.21			
H		1.43	2.34	2.52			
H		1	1.04	1.07			

Functional	Solvent?		Basis Set		Type of Data	
mPW1PW91	PCM		6-311+G(d,p)		Unscaled Shifts	
	Isomer 1	Isomer 2	Isomer 3	Isomer 4	Isomer 5	Isomer 6
sDP4+ (H data)	100.00%	0.00%	-	-	-	-
sDP4+ (C data)	100.00%	0.00%	-	-	-	-
sDP4+ (all data)	100.00%	0.00%	-	-	-	-
uDP4+ (H data)	100.00%	0.00%	-	-	-	-
uDP4+ (C data)	100.00%	0.00%	-	-	-	-
uDP4+ (all data)	100.00%	0.00%	-	-	-	-
DP4+ (H data)	100.00%	0.00%	-	-	-	-
DP4+ (C data)	100.00%	0.00%	-	-	-	-
DP4+ (all data)	100.00%	0.00%	-	-	-	-

Figure S37. DP4+ results obtained using experimental data of **1** *versus* isomers 1 and 2.

Functional	Solvent?	Basis Set	Type of Data
mPW1PW91	PCM	6-311+G(d,p)	Unscaled Shifts

		DP4+	0.00%	0.00%	0.00%	0.00%	100.00%	0.00%	0.00%	0.00%
Nuclei	sp2?	Experimental	Isomer 1	Isomer 2	Isomer 3	Isomer 4	Isomer 5	Isomer 6	Isomer 7	Isomer 8
C		40.7	47.4	46.3	49.7	50.5	41.9	44.23	42.8	43.9
C		28.8	37.5	33.6	39.9	34.1	30.1	36.9	43.2	38.5
C		72.8	81.9	80.9	81.4	83.0	79.8	82.3	83.1	82.7
C		40.2	39.9	39.3	39.6	39.3	40.1	39.9	47.7	39.5
C		36.7	29.7	31.0	38.4	38.0	37.2	29.6	44.8	38.9
C		20	19.7	21.0	19.3	21.6	20.2	18.5	22.2	18.56
C		33.9	36.6	37.9	36.9	38.3	34.8	34.9	38.1	35.9
C		71.3	80.8	78.7	76.8	75.5	74.4	77.7	78.4	74.3
C		38.2	43.0	51.1	43.4	52.0	41.5	45.1	48.1	45.8
C		31.2	24.2	29.7	24.1	29.7	31.9	22.8	34.1	23.1
C		50.2	51.2	56.8	51.1	57.3	50.5	49.45	57.7	50.7
C	x	207	207.36	207.22	207.42	207.45	207.21	209.4	209.9	209.5
C	x	122	121.70	122.18	121.81	122.33	122.31	120.7	120.5	120.7
C	x	155.3	161.12	160.90	157.75	160.80	156.9	162.2	158.3	162.1
C		71.4	71.97	71.99	71.28	72.00	72.1	72.2	71.4	72.2
C		29.3	23.07	22.82	25.48	22.87	25.9	25.3	25.3	25.3
C		29.3	25.05	25.19	25.22	25.19	26.91	23.1	25.4	22.9
C		16.4	20.66	18.97	20.86	19.30	17.01	14.9	9.9	13.7
C		43.1	40.68	52.53	39.75	51.51	42.9	47.1	40.3	46.32
C		27.4	26.39	25.92	18.06	19.09	28.2	26.3	21.4	17.99
H		2.39	2.88	3.03	3.01	3.32	2.54	3.08	3.15	3.052
H		1.74	1.53	1.94	1.15	1.92	1.79	2.49	1.67	2.31
H		2.09	2.02	1.80	1.98	1.71	2.05	2.17	2.92	1.73
H		3.38	3.63	3.89	4.69	4.83	3.49	3.78	4.34	5.03
H		1.19	1.08	1.20	1.50	2.47	1.21	1.14	1.65	2.5
H		1.55	2.49	2.61	2.35	1.47	2.18	2.57	2.29	1.59
H		1.6	1.98	1.78	1.66	1.72	1.64	1.77	1.79	1.92
H		1.72	1.74	1.97	1.88	1.94	1.79	2.01	1.96	1.73
H		1.65	1.97	1.78	1.49	1.66	1.69	1.88	1.65	1.56
H		1.51	1.56	2.01	1.96	1.99	1.56	1.57	2.05	1.87
H		2.39	2.96	2.15	2.92	2.05	2.44	2.71	2.35	2.69
H		1.68	2.86	1.39	2.87	1.35	1.79	1.61	1.77	2.654
H		2.08	1.43	2.61	1.39	2.52	2.02	2.72	2.01	1.6
H		6.41	6.88	6.913	6.98	6.94	6.78	6.84	6.81	6.85
H		6.93	7.86	7.88	7.66	7.89	7.88	7.92	7.64	7.91
H		1.32	1.43	1.44	1.32	1.23	1.53	1.53	1.28	1.53
H		1.32	1.17	1.54	1.29	1.54	1.42	1.43	1.29	1.43
H		1.27	1.08	1.23	1.09	1.26	1.41	1.31	1.41	1.331
H		1.5	1.71	1.32	1.71	1.48	2.47	1.52	1.24	1.445
H		1.36	1.24	1.48	1.18	1.39	1.28	1.27	2.51	1.3
H		0.91	0.89	0.88	0.86	0.91	0.99	1.02	0.92	0.86

Functional	Solvent?	Basis Set	Type of Data
mPW1PW91	PCM	6-311+G(d,p)	Unscaled Shifts

	Isomer 1	Isomer 2	Isomer 3	Isomer 4	Isomer 5	Isomer 6	Isomer 7	Isomer 8
sDP4+ (H data)	0.00%	0.09%	0.00%	0.00%	99.42%	0.49%	0.00%	0.00%
sDP4+ (C data)	0.00%	0.00%	0.00%	0.00%	100.00%	0.00%	0.00%	0.00%
sDP4+ (all data)	0.00%	0.00%	0.00%	0.00%	100.00%	0.00%	0.00%	0.00%
uDP4+ (H data)	0.00%	0.00%	0.00%	0.00%	100.00%	0.00%	0.00%	0.00%
uDP4+ (C data)	0.00%	0.00%	0.00%	0.00%	100.00%	0.00%	0.00%	0.00%
uDP4+ (all data)	0.00%	0.00%	0.00%	0.00%	100.00%	0.00%	0.00%	0.00%
DP4+ (H data)	0.00%	0.00%	0.00%	0.00%	100.00%	0.00%	0.00%	0.00%
DP4+ (C data)	0.00%	0.00%	0.00%	0.00%	100.00%	0.00%	0.00%	0.00%
DP4+ (all data)	0.00%	0.00%	0.00%	0.00%	100.00%	0.00%	0.00%	0.00%

Figure S38. DP4+ results obtained using experimental data of **2** *versus* isomers 1-8.

3.5. Correlation plots of compounds 1 and 2

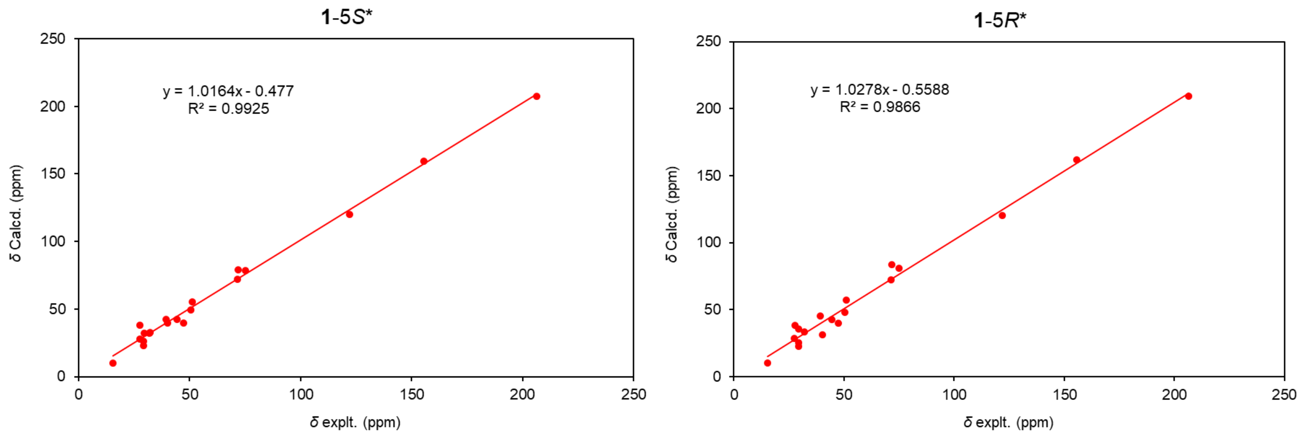

Figure S39. Linear correlations of the calculated isomers of **1** with the experimentally observed ^{13}C NMR chemical shifts.

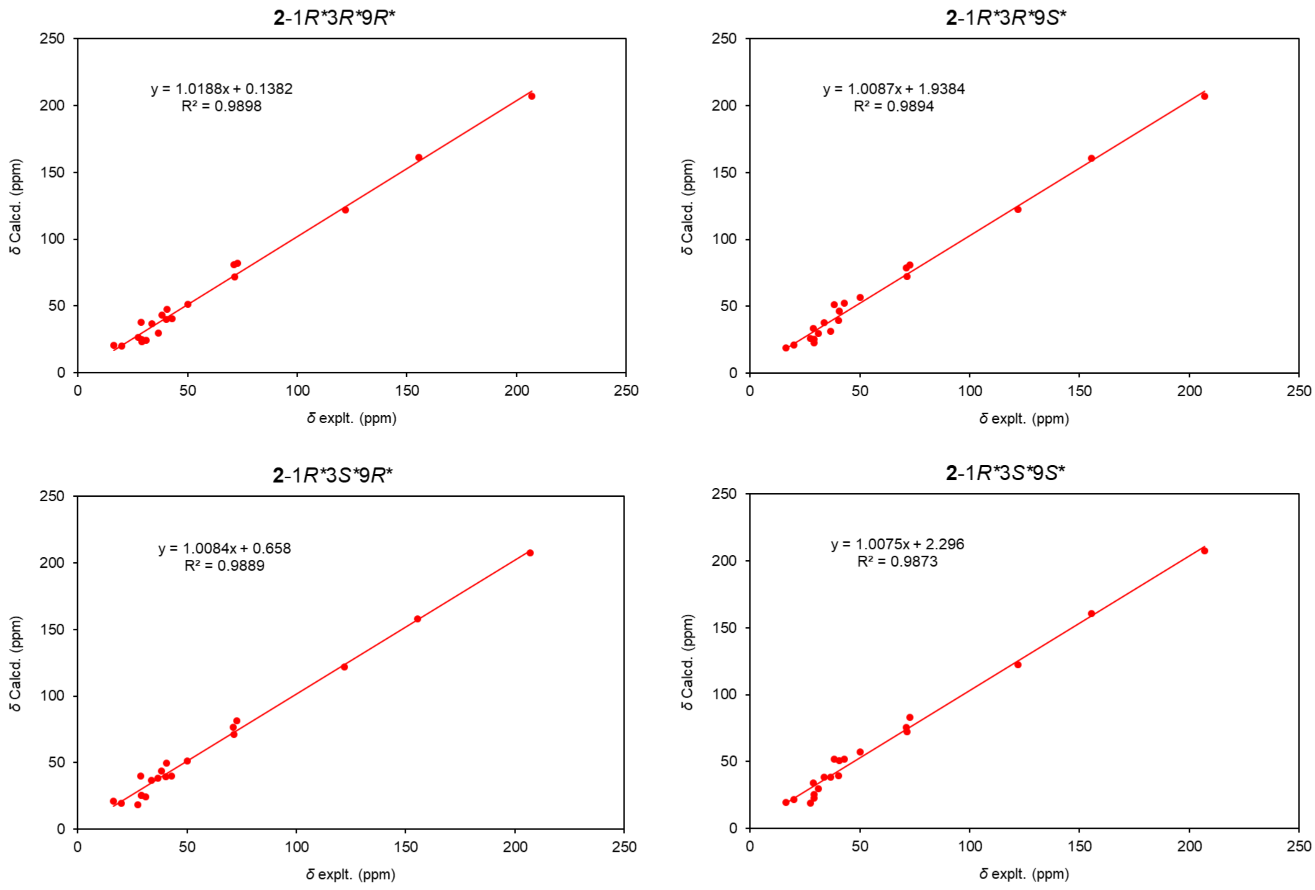

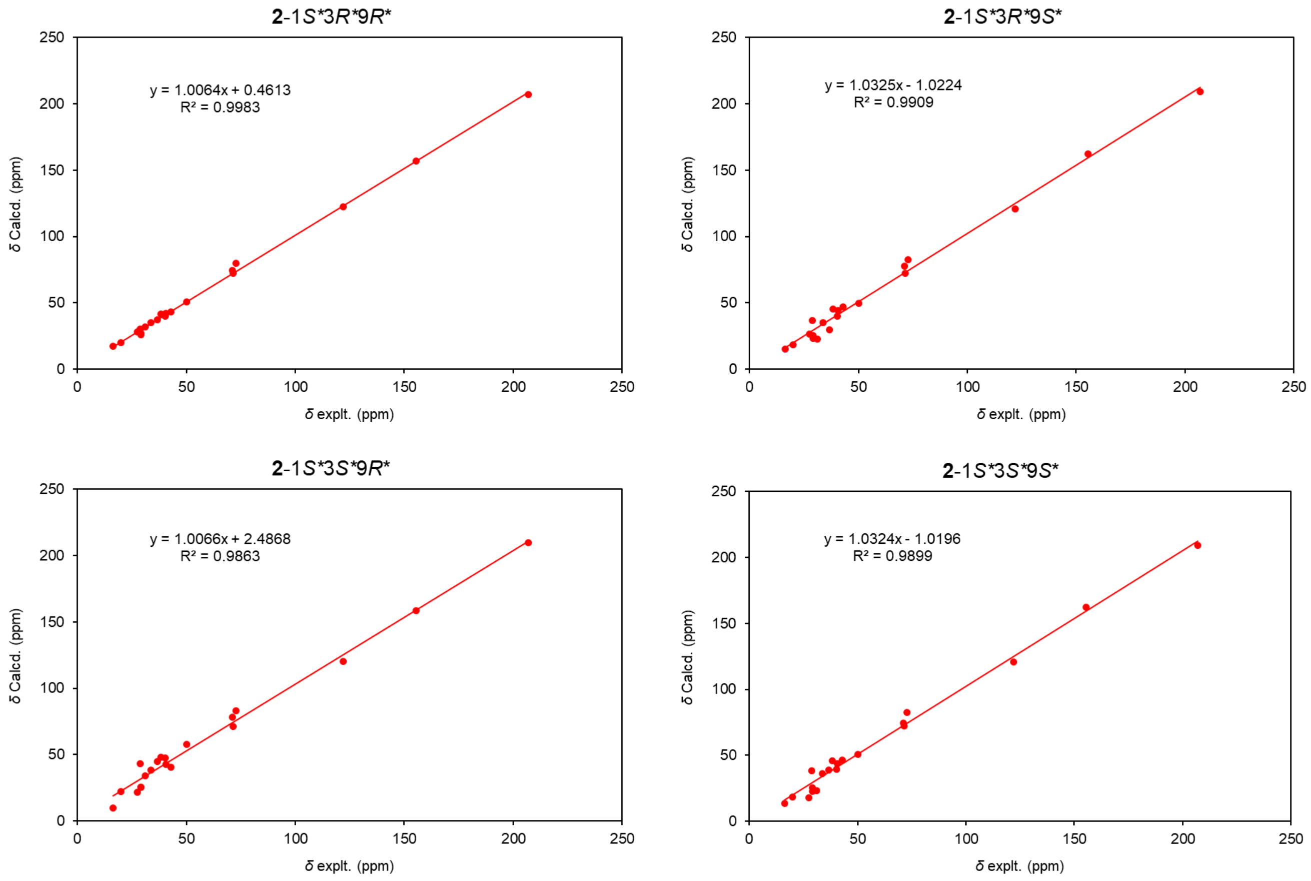

Figure S40. Linear correlations of the calculated isomers of **2** with the experimentally observed ^{13}C NMR chemical shifts.
